# BAP31 Regulates Wnt Signaling to Modulate Cell Migration in Lung Cancer

**DOI:** 10.3389/fonc.2022.859195

**Published:** 2022-03-10

**Authors:** Tianye Li, Zhenzhen Hao, Zihan Tang, Chunting Li, Linglin Cheng, Tao Wang, Xiaojin Zhu, Yunhao He, Yongye Huang, Bing Wang

**Affiliations:** College of Life and Health Sciences, Northeastern University, Shenyang, China

**Keywords:** BAP31, β-catenin, cancer, autophagy, migration

## Abstract

B-cell receptor-associated protein 31 (BAP31) has been shown to overexpress in a wide range type of cancers. The present study aims to investigate the role of BAP31 on migration in lung cancer. Results showed that the migration of BAP31 knockdown cells was weaken than the control cells. Applying TGFβ to treat BAP31 knockdown cells could reduce cell migration. The enhancement on proliferation by TGFβ treatment was downregulated after BAP31 knockdown. The cell death and G0/G1 phase arrest was increased in the cells with TGFβ and BAP31 siRNA treatment when compared with TGFβ treatment alone. Gene expression analysis showed that Bax/Bcl2, MLKL and LC3 was upregulated in the cells with combinatorial treatment of TGFβ and BAP31 siRNA. In addition, BAP31 was shown to regulate multiple signaling pathways, especially for Wnt signaling. It found that BAP31 knockdown cells treated with TGFβ decreased β-catenin cytosolic expression and nuclear localization. Wnt signaling activator BIO could restore the downregulation of proliferation by BAP31 knockdown. This finding suggested that BAP31 regulated cancer cell migration is possibly involved with cell death mechanisms and Wnt signaling.

## Introduction

B-cell receptor-associated protein 31 (BAP31, or BCAP31), a polytopic integral membrane protein of the endoplasmic reticulum, has been primarily shown to regulate apoptosis by forming complex with Bcl2, Bcl-XL and procaspase-8 (1–3). We also have found that BAP31 promoted the retrotranslocation from the ER and degradation of its client protein DeltaF508 mutant of CFTR (4). In recent years, BAP31 has been recognized as a cancer/testis antigen (5). In cervical cancer, BAP31 depletion has been shown to cell invasion and migration by modulating cytoskeleton assemblage (5). In hepatocellular cancer, BAP31 enhancing cell proliferation is found to be associated with serpin family E member 2 (SERPINE2) stabilization, and administration of anti-BAP31 antibody could significantly prevent *in vivo* tumor growth (6). In ovarian cancer, BAP31 modulates migration and invasion *via* epithelial-mesenchymal transition (EMT) process (7). In our previous study, cell death was induced *via* regulating p27 proteasome degradation when applying a BAP31 intrabody in gastric cancer (8). Generally, BAP31 functions in the cancer development *via* multiple molecular mechanisms, including cell death signaling pathways. However, the function on cell death regulation of BAP31 in cancer cell migration has not yet been fully identified.

Impaired cell death mode is considered as one of the cancer hallmarks. Therefore, cell death induction is involved in almost all of the non-surgical strategies to eliminate cancer cells. The apoptosis, autophagy and necroptosis are the common programmed cell death mechanism that critical for cellular homeostasis (9). Activation of the anti-apoptotic system would contribute to cancer cell to maintain survival, establish therapeutic resistance and acquire recurrence (10). Exploiting the mechanisms underlying apoptosis has provided a novel opportunity to enhance the therapeutic sensitivity and precision. Targeting anti-apoptotic Bcl-2 family members, p53, and the caspases et al. are recognized as new targeted therapies (11). In addition, crosstalk between autophagy and apoptosis is also important for cancer development. Autophagy, a “self-feeding” physiological phenomenon, is a cellular catabolic degradation mechanism under nutrient starvation or metabolic stress (12, 13). Autophagy is critical for inhibiting or clearing pathogens in cells. Therefore, autophagy is involved in tumorigenesis and chemotherapy/radiotherapy induced cell death mechanism. BAP31 interacting with STX17 has been shown to inhibit autophagy (14), further confirming that cell death mechanisms might act a key role in the BAP31 participated cancer development and therapy.

Cell migration, an essential process for many biological invents, the abnormality of which is characteristic of cancer cells (15). The epithelial–mesenchymal transition (EMT) process, a fundamental event for cancer growth and distant metastatic spread, is also responsible for the poor response to cancer treatment (16, 17). Transcription program switching in EMT is controlled by a number of signaling pathways, including transforming growth factor β (TGF-β) and Wnt/β−catenin signaling pathways (18). The Wnt/β−catenin signaling pathway has potential function in tumorigenesis, metastasis and recurrence (19, 20). The role of BAP31 in migration and invasion has been confirmed in cervical cancer (5) and ovarian cancer (7). Therefore, the present study tried to further uncover the underlying mechanism of BAP31 in lung cancer migration, especially for cell death and Wnt/β−catenin signaling pathway.

## Materials and Methods

### Cell Culture, Transfection, BAP31 Knockdown and Overexpression Stable Cell Lines Construction

The human lung cancer cell line A549 was cultured in RPMI 1640 medium (Gibco, Shanghai, China), and bladder cancer cell line UC3 was cultured in DMEM medium (Gibco, Shanghai, China). All cells were cultured in a 37℃ humidified incubator with 5% CO_2_ and the culture medium containing 10% fetal bovine serum (FBS) (Gibco, Shanghai, China), 1% penicillin–streptomycin (Gibco, Shanghai, China), 1% L-glutamine (Gibco, Shanghai, China) and 1% non-essential amino acid (NEAA) (Gibco, Shanghai, China) in addition to the basic culture medium.

To transient knockdown BAP31 in cancer cells, siRNA 5′-CCUCCAAUGAAGCCUUUAATT-3′ and 5′-UUAAAGGCUUCAUUGGAGGTT-3′ were used this study. As a control for this study, a non-silencing negative siRNA sequence obtained from GenePharma (Shanghai, China) was used. When cells growing into 50% confluence, siRNA was transfected into cells using Lipofectamine 3000 reagent (Invitrogen, Shanghai, China) according to the manufacture’s guidelines. A final concentration of the siRNA was 100 nM. After being incubated with BAP31 siRNA for 24 hours, cells were re-transfected for another 48 hours. The knockdown cells were harvested after 72 hours.

For transient overexpression studies, a pcDNA3.1(+)-BAP31-Flag plasmid was used, and the empty vector—pcDNA3.1(+) was used as control. When the cells growing into 80% confluence, the cells were transfected using Lipofectamine 3000 reagent (Invitrogen, Shanghai, China) and harvested after 48 hours for subsequent experiments.

For BAP31 stable knockdown cell lines construction, UC3 cells were seeded into a 6-well plate at a density of 20×10^4^ cells/well. Cells were transfected with BAP31 shRNA (Novobio Scientific Co., Ltd., Shanghai, China) plasmid using Lipofectamine 3000 reagent (Invitrogen, Shanghai, China) according to the manufacture’s guidelines. After 48 hours, blasticidin was added to the medium every three days to screen successfully transfected cells. After 20 days treatment, cells were digested and seeded into 96-well plates at a density of 1 cell/well to grow into a monoclonal cell mass. Then, the expression of BAP31 gene was detected to select the positive cell clone with extremely low BAP31 expression. The positive clone cell masses were collected and stored in liquid nitrogen for subsequent experiments.

### Chemicals and Antibodies

TGFβ1 was purchased from Peprotech. Chloroquine (CQ) and magnesium chloride (MgCl_2_) were purchased from Sigma-Aldrich. 3-methyladenine (3-MA), 6-Bromoindirubin-3´-oxine (BIO), XAV939 and Z-VAD-FMK were purchased from Selleck. All the stock solutions were diluted in culture medium prior to experimentation. Unless otherwise specified, all other chemicals were purchased from Sigma-Aldrich.

### Proliferation Ability Detection

The proliferation of UC3 BAP31 knockdown cells was detected using Methyl Thiazolyl Tetrazolium (MTT) assay, and the proliferation of BAP31 siRNA knockdown cells was examined using Cell Counting Kit-8 (CCK-8) assay. In addition, the proliferation was also further determined using soft agar cloning formation assay.

For MTT assay, UC3 BAP31 knockdown cell lines were seeded (2×10^3^ cells/well) into 96-well plates with five replicates. After 24 hours, 10 μl of MTT solution mixed with 100 μl culture medium was added to each well and the cells were incubated for 4 hours at 37°C. Then, the MTT solution was removed, and 100 μl of DMSO was added to dissolve the formazan crystals. Absorbance (OD) was measured using a microplate reader at 570 nm wavelength.

For CCK-8 assay, cancer cells were seeded into 96-well plates with a density 2×10^3^ cells/well and transfected with BAP31 siRNA or negative siRNA control. After 48 hours transfection, cells were treated another 24 hours with different concentrations of culture medium containing chemicals (TGFβ1, BIO, XAV939, MgCl_2_ or Z-VAD-FMK). Finally, cells in each well were added 10 μl of CCK-8 solution mixed with 100 μl culture medium and incubated for 1.5 hours at 37°C. The absorbance of the samples was measured at a wavelength of 450 nm.

Soft agar cloning formation assay was also used to detect the viability of BAP31 knockdown cells. Briefly, 1.5 ml culture medium with 0.6% agar was used as the bottom gel and coated into 6-well plates for each well. After solidification, 1×10^3^ BAP31 knockdown cells were suspended in 1 ml medium with 0.35% agar and poured onto the bottom gel, and the cells were cultured for another 20 days in incubator to form colonies. In the meantime, 150 μl culture medium was added to these plates every 2 days to prevent the cells from drying. Finally, the cells were washed with PBS, fixed with 4% paraformaldehyde for 30 min, and stained with 0.1% crystal violet solution for 15 min. The stained colonies were counted and photographed for further analysis. Each group had three replicates.

### Flow Cytometry Assay

Flow cytometry was used to detect the cell apoptosis and cell cycle of BAP31 knockdown or overexpress cells. In brief, 30×10^4^ cells were seeded to 6-cm dishes and transfected for 48 hours and treated with different chemicals for another 24 hours. The treated cells were incubated with propidium iodide (PI) (BD Biosciences, New York, USA) or an annexin V-FITC/PI apoptosis detection kit (Meilunbio, Dalian, China) for cell cycle and apoptosis detection according to the manufacture’s guidelines. The cells were harvested and analyzed using a flow cytometer (BD LSRFortessa, BD Biosciences, New York, USA).

### Western Blotting Analysis

Cells were washed with PBS and lysed in RIPA buffer (Beyotime, Shanghai, China) containing 1 mM phenylmethanesulfonyl fluoride (PMSF) (Beyotime, Shanghai, China). The extracted protein concentration was measured using a bicinchoninic acid (BCA) protein assay kit (Beyotime, Shanghai, China). Protein samples (20 μg) were separated on 12% sodium dodecyl sulphate polyacrylamide gel electrophoresis (SDS-PAGE) and transferred to polyvinylidene difluoride (PVDF) membranes. After transferring, the membranes were blocked in 5% non-fat milk solution for 1 hour at room temperature, and incubated with specific primary antibodies overnight at 4°C. Then, the membranes were incubated with the appropriate HRP-conjugated secondary antibody at room temperature for 1 hour, and visualized using an ECL detection system. The sources and dilutions of the antibodies used in this study were listed in **Supplementary Table S1**.

### Quantitative Real-Time Polymerase Chain Reaction (qRT-PCR)

Total RNA of the BAP31 knockdown cells was isolated using TRIzol reagent (Tiangen Biotech, Beijing, China) and reverse-transcribed into cDNA using the All-in-One cDNA synthesis SuperMix kit (Bimake, Shanghai, China) according to the manufacturer’s instructions. Quantitative RT-PCR was conducted using 2×SYBR Green qPCR Master Mix (Bimake, Shanghai, China) following the manufacturer’s protocol on a CFX96 real-time PCR detection system and GAPDH was used as a reference gene. The relative expression of the target genes was calculated using the 2^-ΔΔCt^ method.

### Determination of Cell Migration Ability

Transwell assay was performed by a 24-well transwell chamber (8 µm pore size, Greiner Bio-one). Treated cells (2×10^4^ cells/well) were suspended in 100 μl serum-free culture medium and seeded into the upper chamber, and 600 μl medium containing 2.5% FBS was added to the lower chamber of each well. After incubation 24 hours at 37°C, the chambers were washed thrice with PBS, fixed in methanol for 30 min, and stained with 0.1% crystal violet for 15 min at room temperature. Then, the chambers were washed with PBS and five fields were randomly selected and observed under a microscope.

### Immunofluorescence

Immunofluorescence assay was used to detect target protein localization and expression. Briefly, lung cancer A549 cells were seeded at 2×10^4^ cells per well into a 24-well plate and transfected with siRNA and/or TGFβ1 treatment. After that, the cells were fixed with 4% paraformaldehyde for 30 min, permeabilized with 0.2% Triton X-100 for 30 min, and blocked in 1% bovine serum albumin (BSA) in PBS for 30 min at room temperature. Then, the fixed cells were incubated with primary antibodies at 4°C overnight, rinsed thrice with 0.2% Tween-20 in PBS and incubated with the appropriate fluorescence‐conjugated secondary antibody at 4°C overnight in the dark. The sources and dilutions of the antibodies used for immunofluorescence were listed in **Supplementary Table S1**. Finally, the nuclei were stained with Hoechst 33342 for 5 min. A fluorescence microscope was used to observe cells and collect pictures.

### Nuclear and Cytoplasmic Proteins Isolation

To determine β-catenin distribution in BAP31 knockdown cells, cytoplasmic and nuclear proteins were isolated using the Nuclear and Cytoplasmic Protein Extraction Kit (Beyotime, Shanghai, China) according to the manufacturer’s instructions. The concentration of the extracted protein was determined by BCA protein assay kit (Beyotime, Shanghai, China) and the protein samples were detected by Western blot analysis. GAPDH and H3 were served as reference genes for cytoplasm and nucleus, respectively.

### Statistical Analysis

All of the experiments were performed in triplicate unless otherwise noted. The statistical analyses were conducted using SPSS 22 and GraphPad prism 6 software. The data are presented as the means ± the experimental standard error of the mean (SEM). Survival curves were generated using the Kaplan-Meier method and log-rank tests. One-way analysis of variance (ANOVA) was used to determine the statistically significant differences for multiple comparisons. Chi-square analysis was used to test the difference on proliferation or survival rate. *p*-value <0.05 was considered significant.

## Results

### High Expression of BAP31 Predicted Poor Prognosis in Lung Cancer

**Figure 1:**
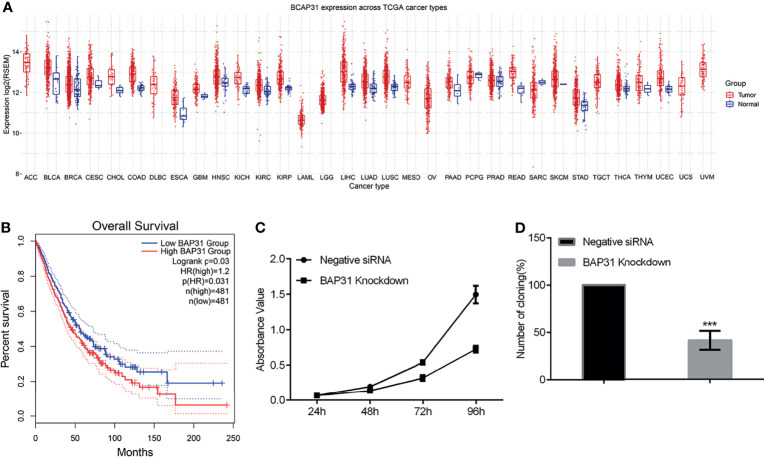
The survival of lung cancer. **(A)** BAP31 expression in different types of cancer datasets [The Cancer Genome Atlas (TCGA), Taylor). **(B)** High levels of BAP31 predicted shorter overall survivals in lung cancer. **(C)** The survival of lung cancer cells A549 treated with BAP31 siRNA was examined by the CCK-8 assay. **(D)** The colony formation of A549 cells with BAP31 knockdown using siRNA. ***p < 0.001 versus control.

To determine the effect of BAP31 on cancers, we first investigated BAP31 expression in different types of cancer datasets [The Cancer Genome Atlas (TCGA), Taylor]. As shown in **Figure 1A**, the expression of BAP31 was increased in several types of cancers compared to normal tissues, including lung adenocarcinoma (LUAD), lung squamous cell carcinoma (LUSC) and bladder urothelial carcinoma (BLCA). Kaplan-Meier analyses based on patient information revealed that high BAP31 expression predicted shorter overall survivals (**Figure 1B**). The above results possible suggest that BAP31 expression is increased in lung cancer tissues and associated with poor prognosis in lung cancer patients.

### Downregulation of BAP31 Decreased the Proliferation of Cancer Cells

Considering the potential role of BAP31 in cancer development, we firstly uncovered the impact of modifying BAP31 expression on cell proliferation. We constructed BAP31 over-expression plasmid using pcDNA3.1(+) as backbone vector. Transient expression of BAP31 over-expression plasmid was conducted in lung cancer cells A549, and CCK-8 assay was performed in the cells received transfection for 24, 48, 72 and 96 hours (**Figure S1**). Results indicated that there was no significant difference on the absorbance value of cells among BAP31 over-expression group, pcDNA3.1(+) empty vector transfection group, and blank control group. To further determine the role of BAP31 on cell proliferation, siRNA was applied to knockdown the expression of BAP31 in A549 cells. As shown in **Figure 1C**, the cell proliferation was down-regulated after decreasing the expression of BAP31, especially at 96 hours. The colony formation assay was then performed in A549 cells, and results also found that the number of cell colony in BAP31 knockdown group was significantly lower than the control group (**Figure 1D**). To further confirmed that BAP31 downregulation impaired the cancer cell proliferation, we constructed two stable BAP31 knockdown cell lines using shRNA in bladder cancer cells UC3 named shBAP31-4 and shBAP31-15. Western blot analysis confirmed that the expression of BAP31 was extremely low in the BAP31 shRNA knockdown cells (**Figure S2C**). Results of MTT assay showed that the proliferation rate of BAP31 shRNA knockdown cells was lower than the control cells (**Figure S2A**). The colony formation rate in both shBAP31-4 and shBAP31-15 cells was also significantly lower than the control cells (**Figure S2B**). Generally, it seems that downregulation of BAP31 could decrease the proliferation of cancer cells.

### Decreasing the Expression of BAP31 Impaired Cell Cycle Progression in Cancer Cells

**Figure 2:**
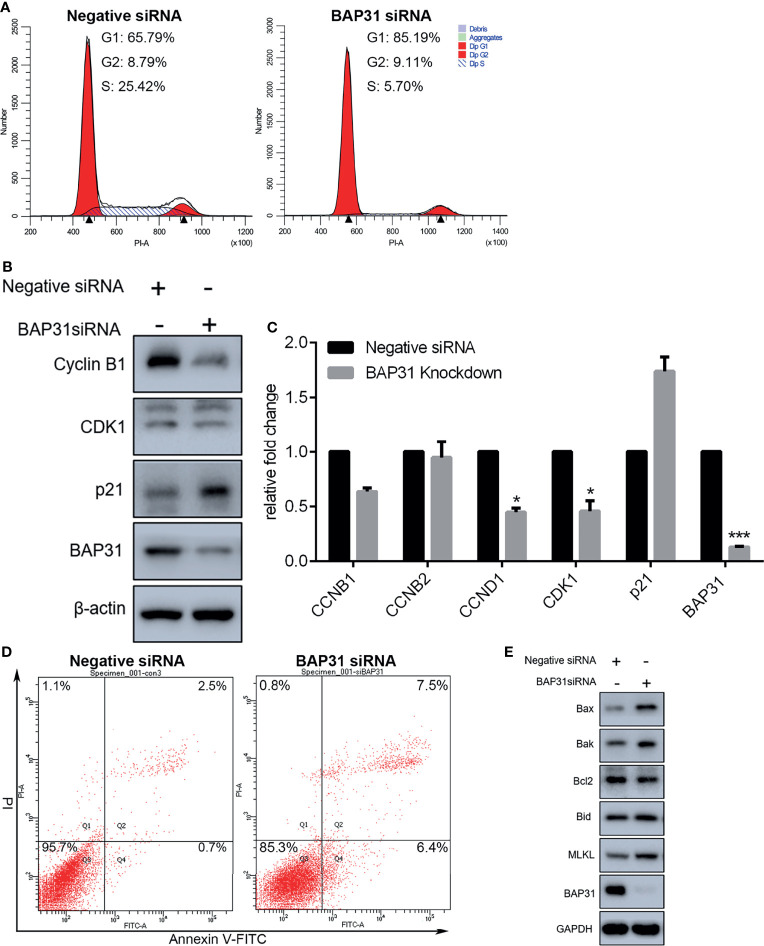
Cell cycle distribution and apoptosis induction analysis in A549 lung cancer cells with BAP31 siRNA knockdown. **(A)** Cell cycle distribution was determined by PI staining using flow cytometry. **(B)** Expression of cell cycle associated genes as evaluated by Western blot analysis. **(C)** Expression of cell cycle associated genes as examined by qPCR (mean ± SEM of duplicate experiments). *p < 0.05 versus control, and ***p < 0.001 versus control. **(D)** Apoptosis in lung cancer cells was determined by annexin V-FITC/PI staining using flow cytometry. **(E)** Expression of apoptosis genes as evaluated by Western blot analysis.

Proliferation is closely connected with regulation on cell cycle distribution. The PI staining-based flow cytometry was used to examine the cell cycle progression in the control of BAP31. Results indicated that the proportion of cells in G0/G1 phase was increased after BAP31 knockdown by siRNA in A549 cells (**Figure 2A**). In addition, the proportion of cells in S phase was remarkedly decreased by BAP31 knockdown. To further uncover the role of BAP31 in cell cycle distribution, the expression of cyclin B1, cyclin B2, cyclin D1, CDK1, p21 and BAP31 was determined by Western blot and/or qRT-PCR (**Figures 2B, C**). Importantly, it found that the expression of BAP31 at both mRNA and protein level was decreased by siRNA. The expression of cyclin D1 and CDK1 was downregulated by BAP31 knockdown as revealed by qRT-PCR. The expression of cyclin B1 at the protein level was showed to be decreased by BAP31 knockdown. Furthermore, p21 expression was found to be upregulated by BAP31 knockdown as determined by Western blot. In sum, BAP31 knockdown induing cell cycle arrest is possibly through regulating a number of cell cycle kinases.

### BAP31 Knockdown Activated Several Cell Death Mechanisms in Cancer Cells

Cell proliferation inhibition is always accomplished with cell death activation. Apoptosis, necroptosis, and autophagy are the common cell death pathways. To determine the occurrence of cell death, annexin V-FITC/PI staining assay was performed and assessed by flow cytometry. As shown in **Figure S1B**, transient expression of BAP31 did not alter the viable cell proportion in lung cancer cells A549. In BAP31 knockdown A549 cells using siRNA technique, many more cells were subjected to death than the control (**Figure 2D**). Results of Western blot confirmed that the expression of BAP31 in A549 cells were decreased by siRNA (**Figure 2E**). Furthermore, the expression of pro-apoptotic protein Bax, Bak and Bid was enhanced by BAP31 knockdown, and the expression of anti-apoptotic protein Bcl2 was decreased by BAP31 knockdown, indicating that apoptosis was evoked in A549 cells after downregulation of BAP31 expression. As an important marker gene in necroptosis, MLKL expression was also promoted in the A549 cells received BAP31 siRNA. To further confirm such findings, the occurrence of cell death was also examined in bladder cancers cells. The expression of some necroptosis (RIPK1, MLKL, and PARP-1) and apoptosis (HRK, FAS, TNFRSF10A, and TNFRSF10B) related genes were accessed using qRT-PCR. Results showed that the expression of PARP-1, TNFRSF10A, and TNFRSF10B was increased in shBAP31-4 cells, and the expression of MLKL, PARP-1 and TNFRSF10A was increased in shBAP31-15 cells (**Figures S2D, E**). To further investigate the impact of BAP31 on the cell death of UC3 cells, annexin V-FITC/PI staining-based flow cytometry was applied. As shown in **Figure S2F**, the cell death proportion was increased after the expression of BAP31 being knockdown by siRNA. In addition, a well-known pan caspase inhibitor Z-VAD-FMK was applied to treat A549 cells in this study, and it confirmed that enhancement on cell death of BAP31 knockdown could be largely prevent by Z-VAD-FMK treatment (**Figure S3A**). Results of CCK-8 assay also demonstrated that the survival rate in BAP31 knockdown cells was reversed by Z-VAD-FMK treatment (**Figure S3B**). Our previous studies have found that MgCl_2_ treatment could inhibit cancer cell survival; therefore, the present study applied MgCl_2_ as a control. It found that MgCl_2_ could subject more BAP31 knockdown cells into death. Generally, these results conveyed a message that downregulation of BAP31 could induce cell death in cancer cells.

**Figure 3:**
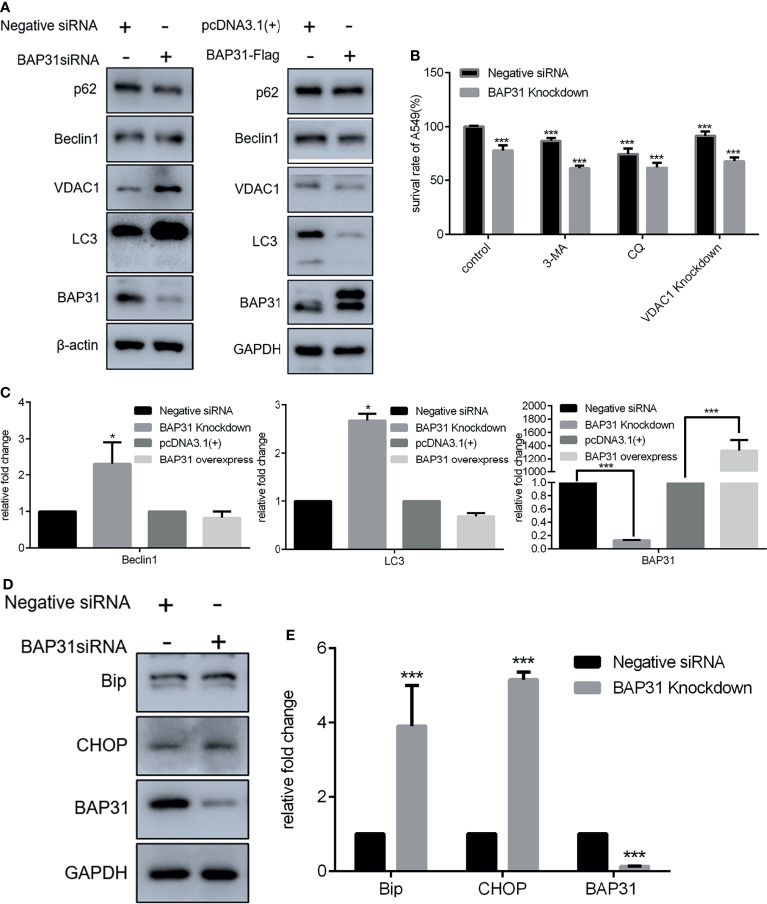
Expression of genes related to homeostasis in lung cancer cells after altering BAP31 expressing. **(A)** Expression of autophagy associated genes as assessed by Western blot in the cells transfected with BAP31 siRNA, or BAP31 overexpressing plasmid (pcDNA3.1(+)-BAP31-Flag). **(B)** The survival of lung cancer cells was examined by the CCK-8 assay. The BAP31 siRNA knockdown cells received combinatorial treatment with 3-Methyladenine (3-MA), Chloroquine (CQ) or VDAC1 siRNA. **(C)** Expression of autophagy associated genes was determined by qPCR (mean ± SEM of duplicate experiments). **(D)** Expression of ER stress related genes as assessed by Western blot. **(E)** Expression of ER stress associated genes was determined by qPCR (mean ± SEM of duplicate experiments). *p < 0.05 versus control and ***p < 0.001 versus control.

Autophagy is defined as a conserved intracellular self-degradation system that is critical for cellular renovation and homeostasis in response to stress conditions. Excessive autophagy leads to a form of programmed cell death named as autophagic cell death. Therefore, the regulatory role of BAP31 in autophagy induction was investigated in A549 cells in this study. The expression of autophagy related genes was firstly examined. Results of qRT-PCR revealed that Beclin 1 and LC3 was enhanced in the cells knockdown with BAP31 siRNA (**Figure 3**). The expression of SQSTM1/p62 was downregulated by BAP31 siRNA. However, over-expression of BAP31 in A549 cells did not largely change SQSTM1/p62 expression. In addition, BAP31 knockdown promoted the expression of LC3, and BAP31 overexpression downregulated the expression of LC3, indicating that BAP31 possibly exerted a negative role in autophagy occurrence. To further ascertain the relationship between BAP31 and autophagy, two autophagy inhibitors 3-Methyladenine (3-MA) and Chloroquine (CQ) were used to treat BAP31 knockdown cells. As shown in **Figure 3B**, both 3-MA and CQ could further enforce the inhibitory effect of BAP31 knockdown on cell proliferation. Furthermore, the expression of VDAC1, which is critical for mitophagy, was increased in the BAP31 knockdown cells, and its expression was decreased in the BAP31 overexpressing cells. It found that VDAC1 knockdown could also enhance the inhibitory effect of BAP31 knockdown on cell proliferation. ER stress is considered as a potent trigger for autophagy. The expression of Bip and CHOP was shown to be enhanced in the BAP31 knockdown cells at both mRNA and protein level. These results further validated that BAP31 worked as a stabilizer for autophagy induction.

### BAP31 Participated in the Regulation on Migration and Stemness of Lung Cancer

**Figure 4:**
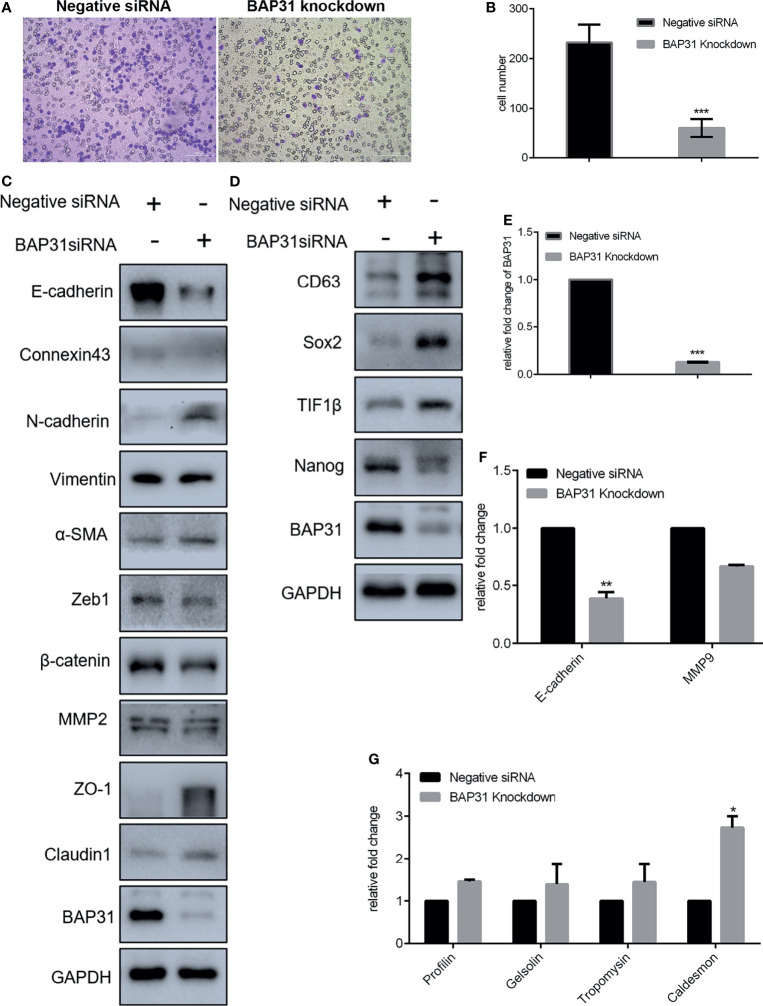
Migration of A549 lung cancer cells that transfected with BAP31 siRNA. **(A)** Cell migration was evaluated by the trans-well assay. Cells that had undergone migration were stained with 0.1% crystal violet. **(B)** Statistics on the average migrated cell number in trans-well assay. **(C, D)** Protein expression as determined by Western blot analysis. **(E–G)** mRNA expression as examined by qPCR (mean ± SEM of duplicate experiments). *p < 0.05 versus control, **p < 0.01 versus control and ***p < 0.001 versus control.

Considering the high expression of BAP31 in lung cancer tissues, it urged us to determine its role in cancer cells migration and stemness. As revealed by trans-well assay, the migrated cells in BAP31 knockdown group were significantly less than the control group (**Figures 4A, B**). Expression profile of EMT related genes was then examined. Results of both qRT-PCR and Western blot analysis revealed that the expression of E-cadherin was decreased after BAP31 knockdown (**Figures 4C, E, F**). The expression of Connexin 43 was also downregulated in the BAP31 knockdown cells. The expression of N-cadherin, α-SMA and Claudin 1 was promoted in the cell knockdown by BAP31 siRNA. Thus, it seems that the EMT process was evoked in the BAP31 knockdown cells though that the migrated cells become less. Certainly, there still a sum of EMT associated genes expression indicated that BAP31 knockdown could prevent EMT process. The expression of ZO-1 and Claudin 1 was increased, and the expression of Zeb l was decreased in the BAP31 knockdown cells. To investigate the stemness of cancer cells treated with BAP31 siRNA, the expression of CD63, Sox2, TIF1β and Nanog was then analyzed. As shown in **Figure 4D**, the expression of CD63, Sox2 and TIF1β was upregulated by BAP31 knockdown. However, the expression of Nanog was downregulated in the BAP31 knockdown cells. We also determined the expression of cytoskeleton genes Profilin, Gelsolin, Tropomysin and Caldesmon. As shown in **Figure 4G**, the expression of Caldesmon was promoted in the cells treated with BAP31 siRNA. These results possibly convey a message that BAP31 participated in the migration and stemness of lung cancer cells, but its actual role should be further identified.

### BAP31 Knockdown Enhanced TGFβ Induced Cell Death of Lung Cancer Cells

**Figure 5:**
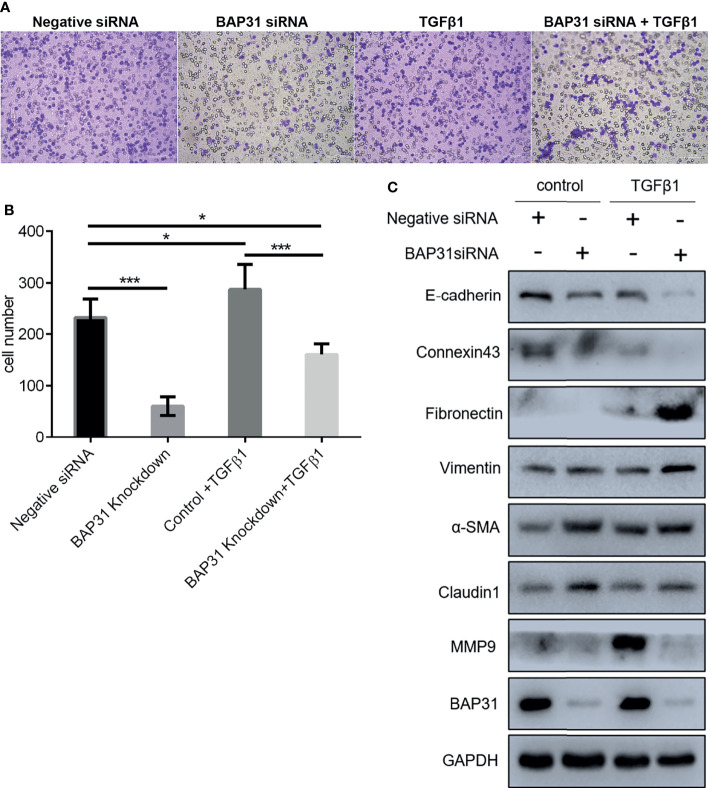
Migration of A549 lung cancer cells with combinatorial treatment of BAP31 siRNA and TGFβ. **(A)** Cell migration was examined by the trans-well assay. Cells that had undergone migration were stained with 0.1% crystal violet. **(B)** Statistics on the average migrated cell number in trans-well assay (mean ± SEM of duplicate experiments). *p < 0.05 versus control and ***p < 0.001 versus control. **(C)** Protein expression as determined by Western blot analysis.

To exploit the actual role of BAP31 in cancer cell metastasis, the EMT process in A549 cells was induced *via* TGFβ treatment. As shown in **Figures 5A, B**, the TGFβ induced cell migration was reduced by BAP31 knockdown. However, the expression of both E-cadherin and Connexin 43 were also further downregulated in the TGFβ treated cells in combination with BAP31 knockdown (**Figure 5C**). The expression of Fibronectin, Vimentin, α-SMA and Claudin 1 in the cells with TGFβ and BAP31 siRNA treatment was higher than the cells with TGFβ treatment alone, possibly suggesting that BAP31 knockdown could activate the induction of TGFβ induced EMT process. The expression of Nanog was also further downregulated, CD63 and TIF1β was upregulated in the cells with TGFβ and BAP31 siRNA treatment (**Figure 7C**). It urged us to find out the molecular mechanism underlying BAP31 knockdown cells with potential high ability of migration but showing actual low migration proportion.

**Figure 6:**
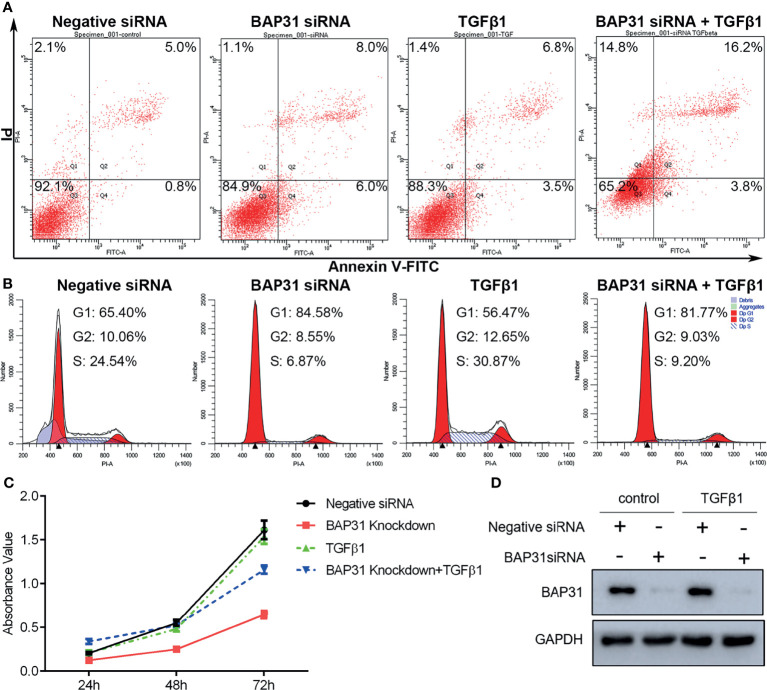
The survival of A549 lung cancer cells with BAP31 siRNA knockdown and TGFβ treatment. **(A)** Apoptosis was determined by annexin V-FITC/PI staining using flow cytometry. **(B)** Cell cycle distribution was examined by PI staining using flow cytometry. **(C)** The cell survival was examined by the CCK-8 assay. **(D)** Expression of BAP31 as evaluated by Western blot analysis.

**Figure 7:**
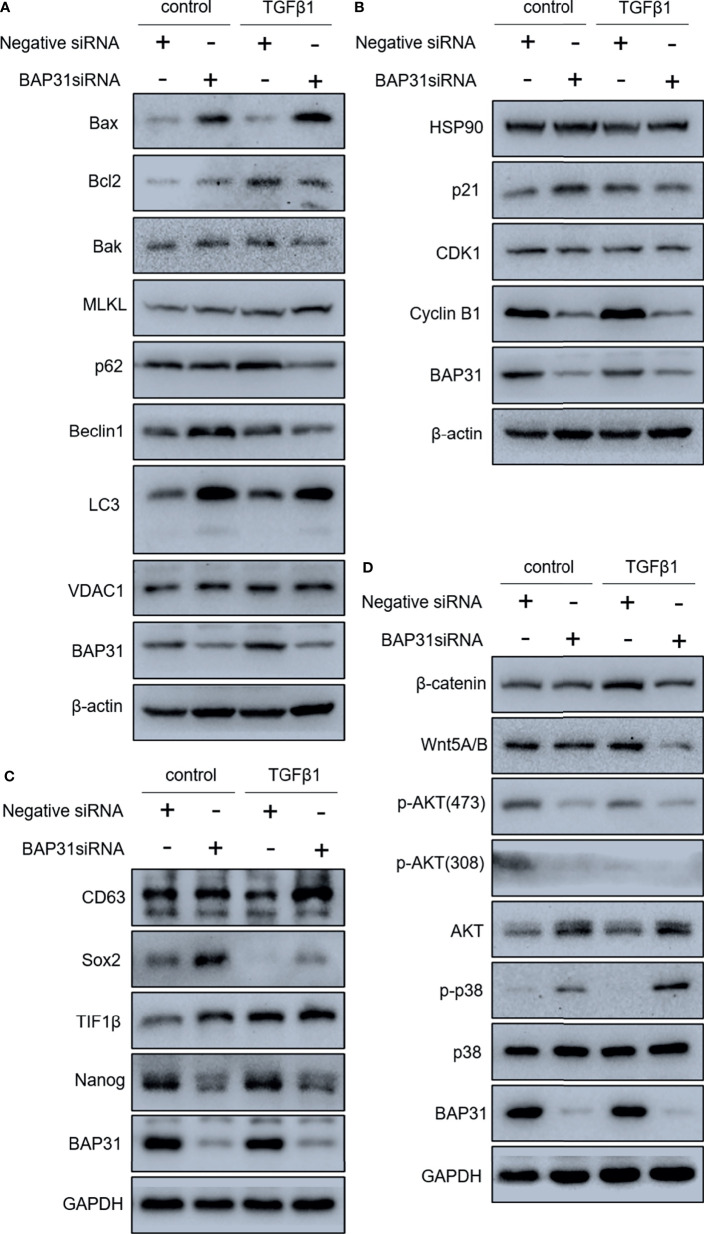
Gene expression in A549 lung cancer cells received combinatorial treatment with TGFβ and BAP31 siRNA via Western blot. **(A)** Expression of cell death-related genes. **(B)** Gene expression related to cell cycle distribution. **(C)** Expression of genes associated with stemness. **(D)** Determination of gene expression in signaling pathways.

Considering the above results that BAP31 played important role in cell death, the occurrence of apoptosis in the cells received TGFβ treatment was firstly determined. As shown in **Figure 6A**, TGFβ treatment could also decrease the proportion of viable cells when compared with control group, and TGFβ treatment in combination with BAP31 knockdown could further enhance the proportion of death cells. Western blot analysis found that the ratio of Bax/Bcl2 was enhanced in the cells with TGFβ and BAP31 siRNA treatment (**Figure 7A**). The expression of MLKL in the cells with combinatorial treatment of TGFβ and BAP31 siRNA was also highest among the examined groups. In addition, the expression of SQSTM1/p62 and Beclin 1 was decreased, and the expression of LC3 was increased in the cells with TGFβ and BAP31 siRNA treatment when compared to the control. These results demonstrate that downregulation of BAP31 would make TGFβ treated cancer cells be in favor of undergoing apoptosis, necroptosis and autophagy.

The cell cycle distribution was examined by flow cytometry using PI staining. As shown in **Figure 6B**, TGFβ treatment was shown to decrease the proportion of cancer cells at G0/G1 phase. Furthermore, BAP31 knockdown could make more TGFβ treated cells into G0/G1 phase. Results of CCK-8 assay also found that BAP31 knockdown could downregulate the proliferation of TGFβ treated cells (**Figures 6C, D**). Compared to the cells with TGFβ treatment alone, the expression of p21 and cyclin B1 was shown to be decreased in the cells with both TGFβ and BAP31 siRNA treatment (**Figure 7B**), indicating that BAP31 participated in the regulation of cell cycle in TGFβ induced EMT process.

### Wnt Signaling Contributed to the Cell Death Induction of BAP31 Knockdown

**Figure 8:**
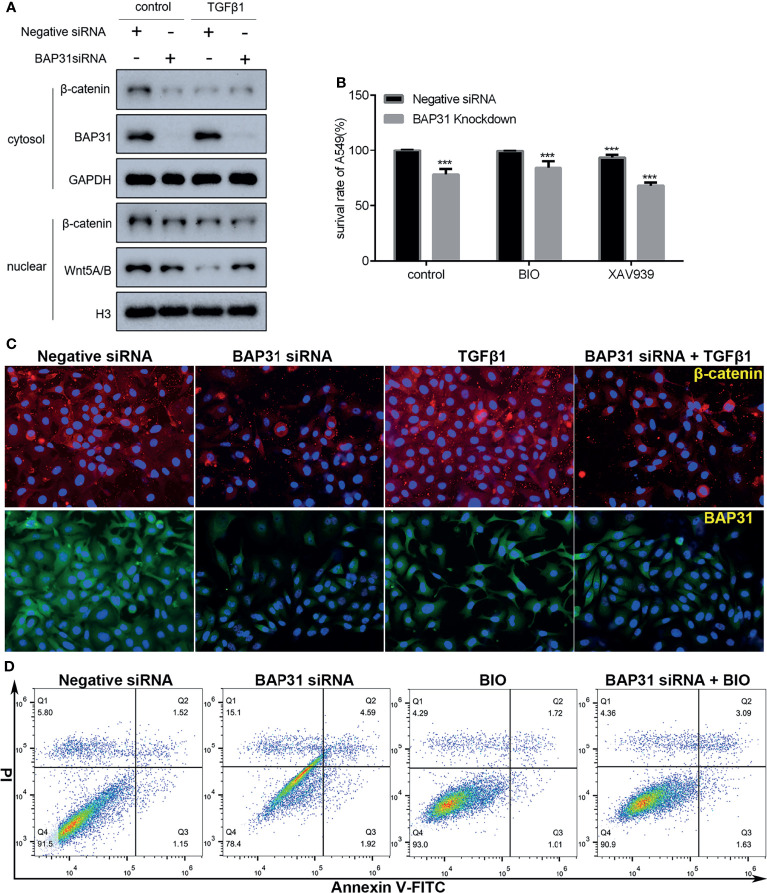
Investigation on the role of Wnt/β-catenin signaling in the A549 lung cancer cells. **(A)** Expression of nuclear β-catenin as evaluated by Western blot. **(B)** The survival of lung cancer cells was examined by the CCK-8 assay. The BAP31 siRNA knockdown cells received combinatorial treatment with BIO or XAV939. ***p < 0.001 versus negative siRNA in control group. **(C)** Distribution of β-catenin (red) and BAP31 (green) as determined by immunofluorescence. Nuclei were stained with Hoechst 33342 (blue). **(D)** Apoptosis was determined by annexin V-FITC/PI staining using flow cytometry.

The AKT, ERK and Wnt signaling are all important pathways for cell survival. As shown in **Figure 7D**, the phosphorylation of AKT at both Threonine 308 and Serine 473 was decreased in the cells with both TGFβ and BAP31 siRNA treatment. The phosphorylation of p38 MAPK was found to be upregulated in the TGFβ treated cells in combination with BAP31 siRNA treatment. The expression of both Wnt5A/B and β-catenin was decreased in the cells with both TGFβ and BAP31 siRNA treatment. To further uncover the role of these multiple signaling pathways in BAP31 participated cell death induction in cancer cells, Wnt signaling was selected to be further investigated. As shown in **Figure 8A**, the expression of both Wnt5A/B and β-catenin was also decreased in the nuclear protein from cells treated with both TGFβ and BAP31 siRNA. Meanwhile, results of immunofluorescence also confirmed that BAP31 knockdown would prevent the β-catenin translocate into nuclear (**Figure 8C**). Therefore, it urged us to consider whether activation of Wnt signaling would reverse the cell death induction of BAP31 knockdown. As shown in **Figure 8B**, BIO treatment could promote the survival rate in BAP31 knockdown A549 cells. In contrast, applying Wnt signaling inhibitor XAV939 could further decrease the proliferation in BAP31 knockdown cells. Results of cytometry analysis also found that BIO treatment could prevent the death in cells transfected with BAP31 siRNA (**Figure 8D**).

## Discussion

Migration and metastasis are the hallmark of cancer. In the present study, the cell migration was reduced after BAP31 knockdown. TGFβ is a key cytokine orchestrating EMT, and TGFβ treatment would make epithelial cells change from cuboidal to an elongated spindle shape (21, 22). To further confirm the underlying mechanism of BAP31 in migration, TGFβ treatment was applied to induce EMT process in lung cancer cells. It found that TGFβ induced enhancement on cancer cell migration could also be reduced after BAP31 knockdown. In addition, the proliferation in TGFβ treated cells was decreased by BAP31 knockdown. Cell proliferation is a fundamental biological process required for organismal development and tissue homeostasis. Inhibition of cell proliferation is a basic goal for cancer therapy. Dysregulation of the cell-cycle progression is the major factor leading to uncontrolled cell proliferation (23). In this study, the proportion of cells in G0/G1 phase was decreased in the cells with TGFβ treatment, but the G0/G1 phase arrest turned up when downregulating the expression of BAP31. It is well known that each step of cell cycle progresses is regulated by the actions of cyclins and their counterpart cyclin-dependent kinases (CDKs) (24). The expression of p21, CDK1 and cyclin B1 was shown to be altered in BAP31 knockdown cells, suggesting that regulation on cell cycle distribution contributed to BAP31 participated cancer cell migration.

Modulation on apoptosis has been recognized as the major function of BAP31 as early as two decades ago (1–3). Therefore, it urged us to consider whether this function would also be involved in the BAP31 participated cancer cell migration. Results of both annexin V-FITC/PI staining assay and Western blot analysis supported our hypothesis. Besides apoptosis, the other death/recycling pathways, such as necroptosis and autophagy, are also an integral part of the homeostasis as well as the pathophysiology in the life of living organisms. Many reports have documented that MLKL is critical in the occurrence of necroptosis (25, 26). The high expression of MLKL was presented in the cells with TGFβ treatment, especially after BAP31 knockdown. It seems that necroptosis was induced when BAP31 expression was interfered. Autophagy is a highly conserved catabolic process which controlled by a set of signaling events (27). This lipid phosphatidylethanolamine-conjugated form of LC3 has been commonly considered as an autophagosome marker (27, 28). The formation of LC3-II was increased in the cells with BAP31 knockdown alone or in combination with TGFβ treatment, suggesting that autophagy induction was triggered by downregulation of BAP31. Therefore, BAP31 regulated migration was possibly associated with its function on apoptosis, necroptosis and autophagy.

The Wnt/β-catenin signal transduction cascade is the key signaling regulating development and stemness (29, 30). The role of Wnt signaling in carcinogenesis and development has been well documented in varied type of cancers. Wnt signaling affects the establishment of cancer stem cells and promotion to metastasis (29). In this study, the expression of Wnt5A/B and β-catenin was decreased in the cells received both TGFβ and BAP31 siRNA treatment. Especially, the nuclear β-catenin was downregulated in the cells with TGFβ treatment and/or BAP31 knockdown. It is well known that the activation of Wnt signaling pathway depends on the nuclear translocation of β-catenin (31). Therefore, it seems that the Wnt/β-catenin signaling was possibly inactivated after BAP31 knockdown. Results of the present study also showed that the expression of Sox2 and Nanog was decreased in the TGFβ treated cells in combination with BAP31 knockdown. The impact on cancer stemness of BAP31 might also be connected with Wnt/β-catenin signaling. Applying BIO to activate Wnt/β-catenin signaling, the downregulation on cancer cell survival after BAP31 knockdown was restored. Therefore, targeting BAP31 and Wnt/β-catenin signaling coordinately would alter the process of cancer development.

The expression of E-cadherin was also shown to be downregulated in TGFβ treated cells in combination with BAP31 knockdown. E-cadherin is a critical mediator of stable cell to cell adhesion and essential for cancer cell dissemination (32). Loss of E-cadherin expression would lead to loss of contact inhibition and increase cell motility of cancer (33). In this point of view, downregulation of BAP31 would possibly make the cells acquire the ability of metastasis. Actually, E-cadherin has been shown to couple with death receptors to the cytoskeleton to modulate the onset of apoptosis (34), and loss of E-cadherin mediated cell adhesion is considered as an early trigger of apoptosis in a photodynamic treatment (35). Furthermore, regulation of apoptosis induction is also an important function of TGFβ signaling (36, 37). Therefore, the downregulation of E-cadherin in BAP31 knockdown cells might also be a hint of apoptosis. Additionally, E-cadherin/β-catenin complex is suggested as a target for anticancer and antimetastatic drugs (38, 39). Targeting BAP31 to decrease E-cadherin/β-catenin complex would be a novel strategy for cancer treatment, especially in combination with some anticancer drugs.

## Data Availability Statement

The original contributions presented in the study are included in the article/**Supplementary Material**. Further inquiries can be directed to the corresponding authors.

## Author Contributions

TL and YYH conceived and designed the experiments. TL, ZH, ZT, CL, LC, TW, XZ, and YHH performed the experiments and analyzed the data. YYH and BW wrote the manuscript. TL and BW reviewed the manuscript. All authors approved the manuscript.

## Funding

This study was funded by the National Natural Science Foundation of China (Nos.81502582, 31670770, 2016YFC1302402, and 31370784). Funding was also provided by the Fundamental Research Funds for the Central Universities (N182004002), Natural Science Foundation of Liaoning Province (2021-MS-104), Fundamental Scientific Research Fund of Liaoning Provincial Education Department (LJKQZ2021002), Liaoning Revitalization Talents Program (XLYC1902063), and Key Research and Development Plan of Liaoning Province (2020JH2/10300080).

## Conflict of Interest

The authors declare that the research was conducted in the absence of any commercial or financial relationships that could be construed as a potential conflict of interest.

## Publisher’s Note

All claims expressed in this article are solely those of the authors and do not necessarily represent those of their affiliated organizations, or those of the publisher, the editors and the reviewers. Any product that may be evaluated in this article, or claim that may be made by its manufacturer, is not guaranteed or endorsed by the publisher.

## Supplementary Material

Supplementary Figure 1 The survival of A549 lung cancer cells with transient transfection of pcDNA3.1(+)-BAP31-Flag plasmid. **(A)** The cell survival was examined by the CCK-8 assay. **(B)** Apoptosis was determined by annexin V-FITC/PI staining using flow cytometry. **(C)** Cell cycle distribution was examined by PI staining using flow cytometry. **(D)** Expression of BAP31 and cyclin B1 as evaluated by Western blot analysis.

Supplementary Figure 2 The survival of UC3 bladder cancer cells with BAP31 knockdown. **(A)** The survival of cells with BAP31 knockdown using shRNA was examined by the MTT assay. shBAP31-4: cells from colony 4# with BAP31 knockdown using shRNA. shBAP31-15: cells from colony 15# with BAP31 knockdown using shRNA. **(B)** The colony formation of UC3 cells with BAP31 knockdown using shRNA (mean ± SEM of duplicate experiments). **(C)** Expression of BAP31 in cells with BAP31 knockdown using shRNA as evaluated by Western blot analysis. **(D, E)** mRNA expression as examined by qPCR in cells with BAP31 knockdown using shRNA (mean ± SEM of duplicate experiments). **(F, G)** Apoptosis was determined by annexin V-FITC/PI staining using flow cytometry in cells with BAP31 siRNA knockdown. Expression of BAP31 as evaluated by Western blot analysis. **p*<0.05 versus control, ***p*<0.01 versus control and ****p*<0.001 versus control.

Supplementary Figure 3 The survival of BAP31 knockdown A549 lung cancer cells treated with MgCl_2_ or Z-VAD-FMK. **(A)** Apoptosis was determined by annexin V-FITC/PI staining using flow cytometry. **(B)** The cell survival was examined by the CCK-8 assay. ****p*<0.001 versus negative siRNA in control group.
