# Supplementary material for: BAP31 Regulates Wnt Signaling to Modulate Cell Migration in Lung Cancer
Source: Front Oncol. 2022 Mar 10;12:859195. doi: 10.3389/fonc.2022.859195 (PMC8960194; doi:10.3389/fonc.2022.859195)

Fig2B

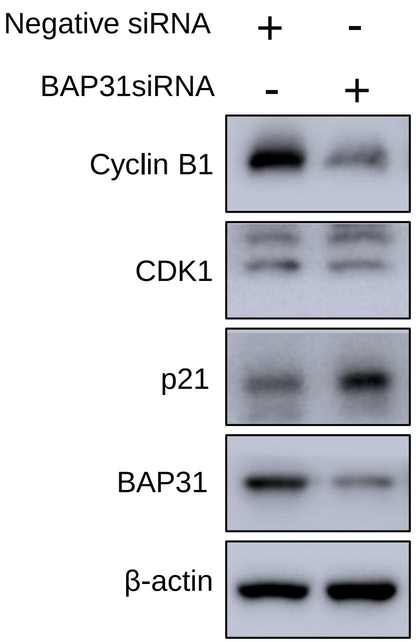

Fig2B cyclinb1

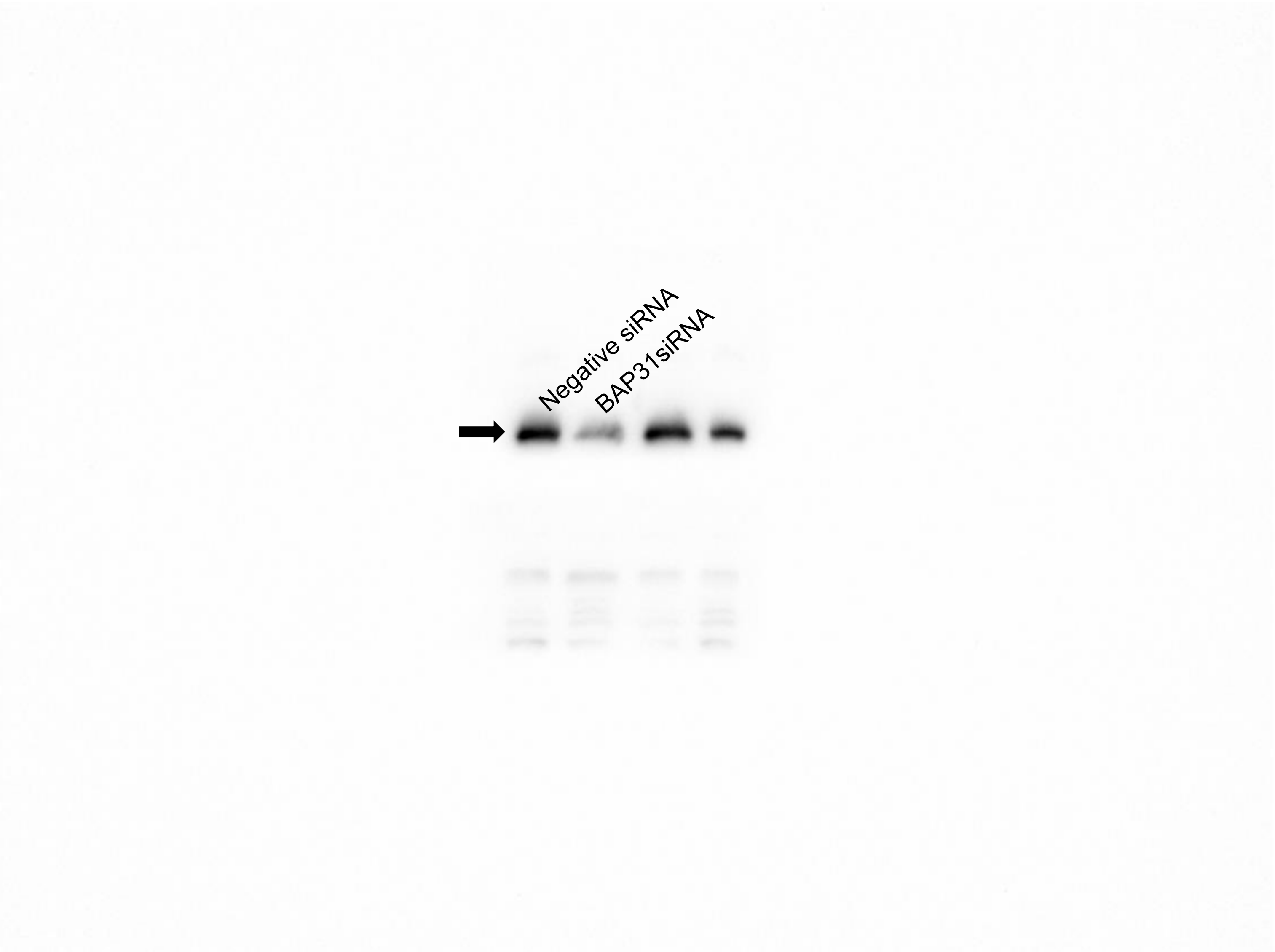

Fig2B cdk1

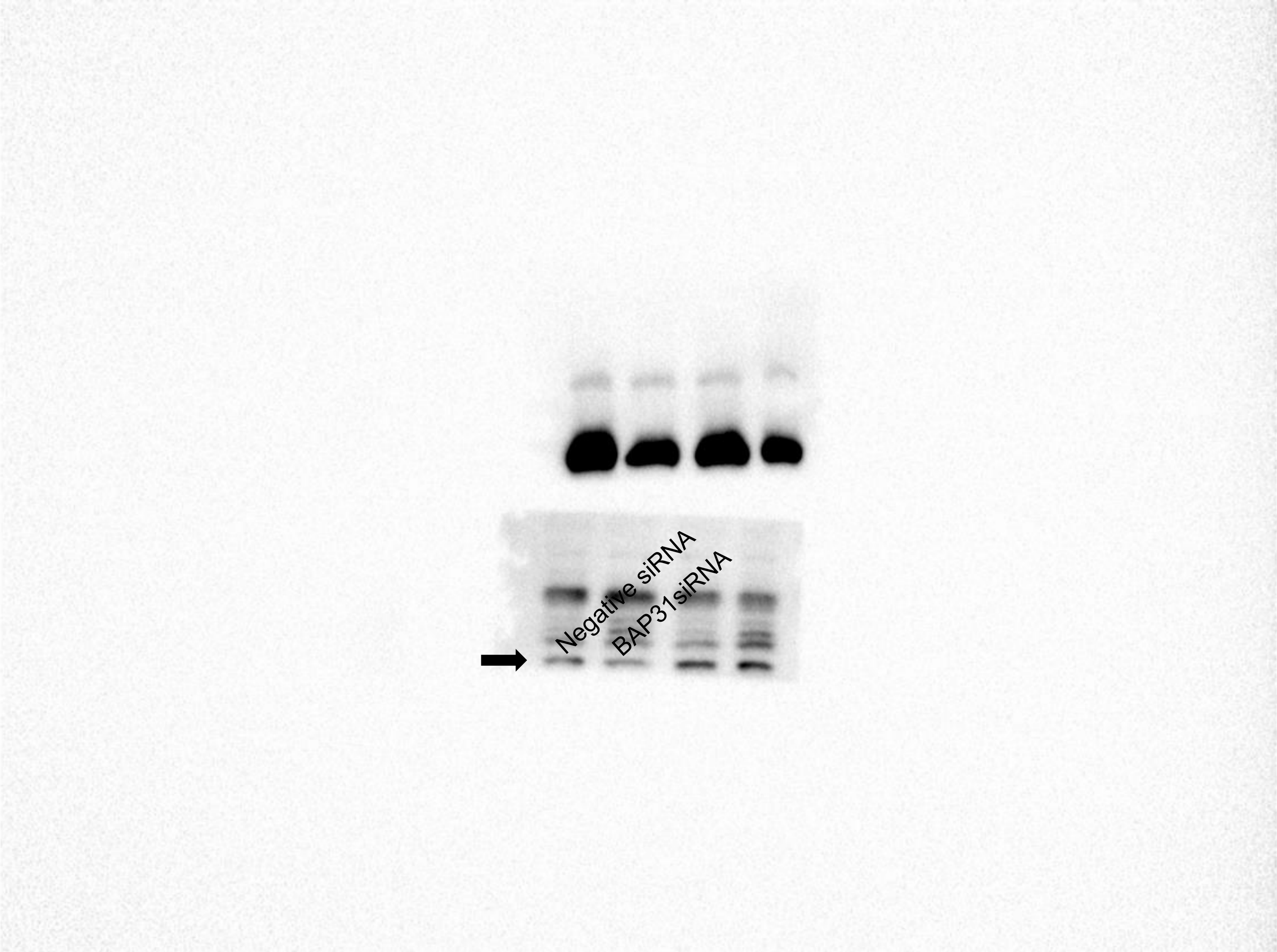

Fig2B p21

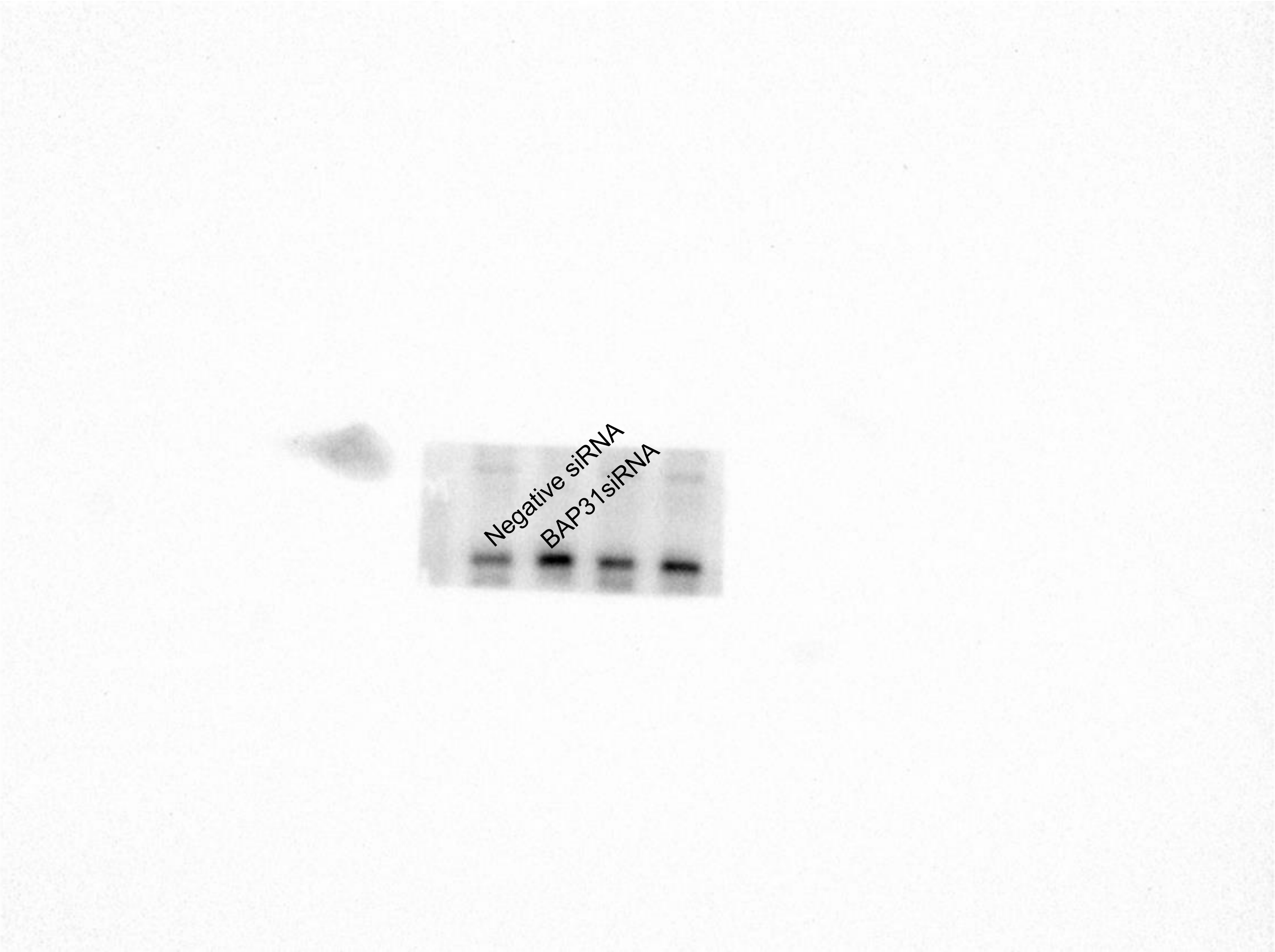

Fig2B bap31

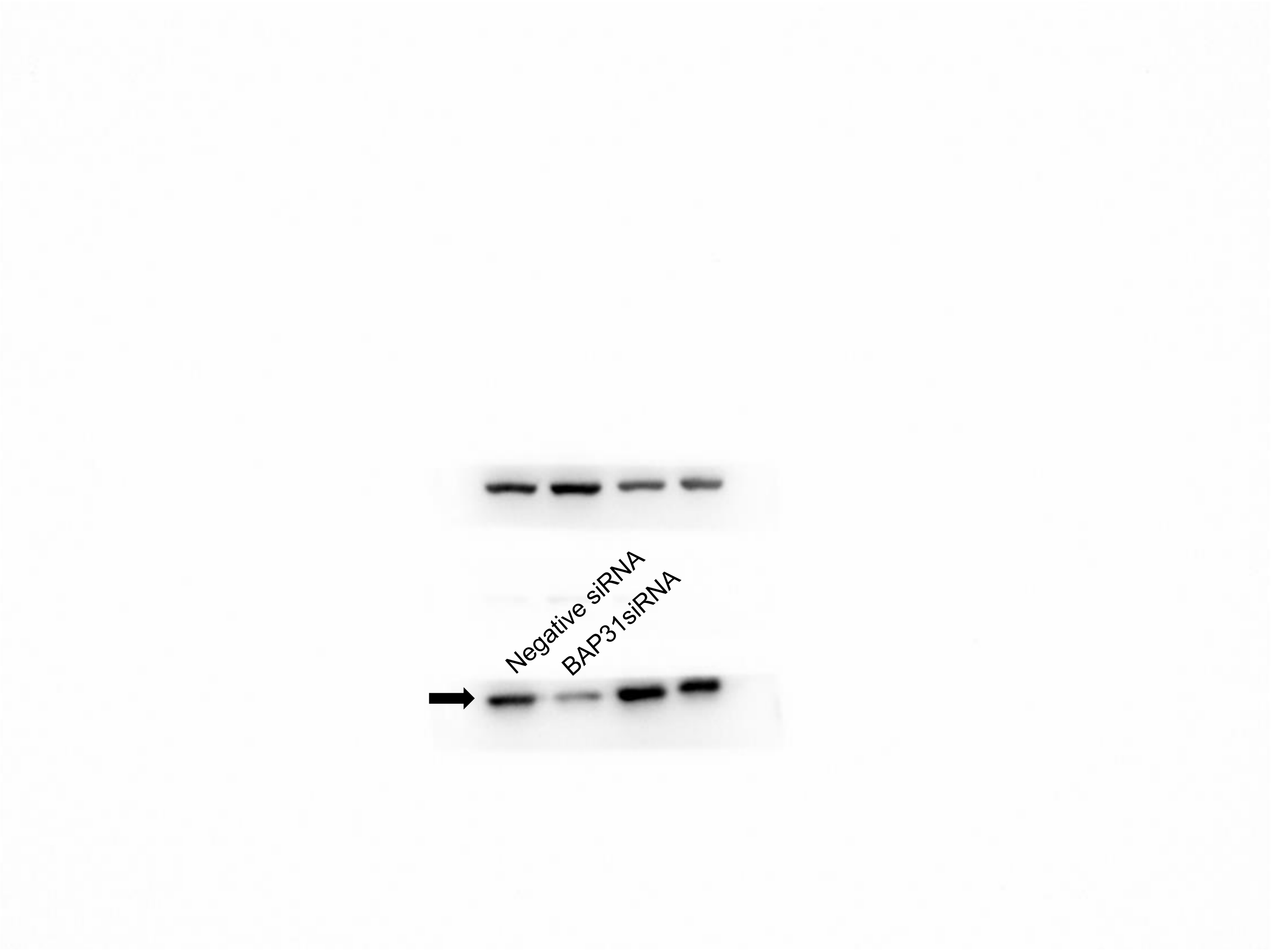

Fig2B beta-actin

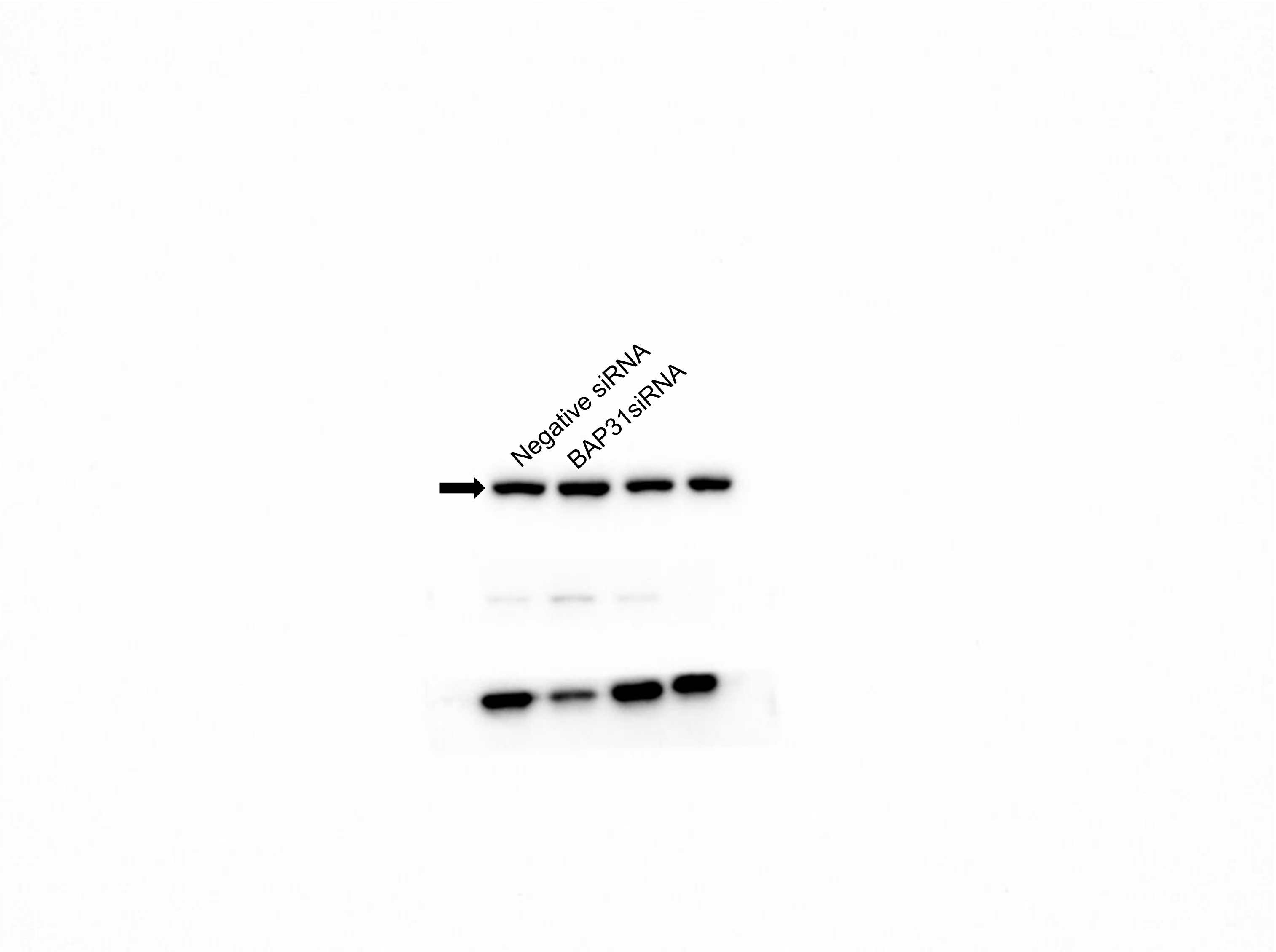

Fig2E

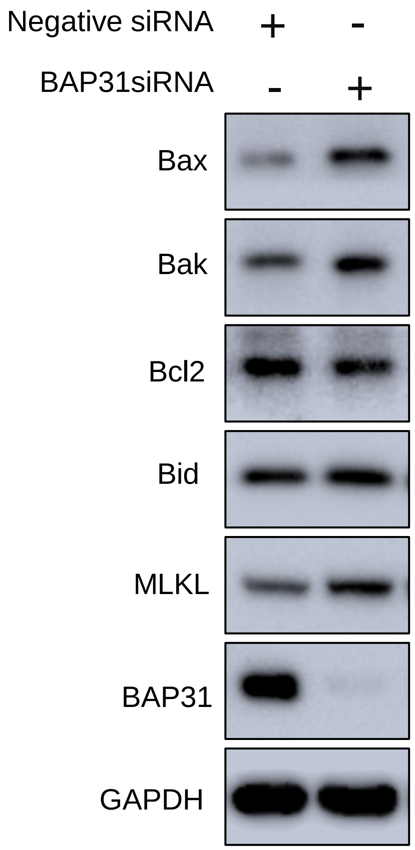
Negative siRNA + -
BAP31siRNA - +
Bax
Bak
Bcl2
Bid
MLKL
BAP31
GAPDH

Fig2E bax

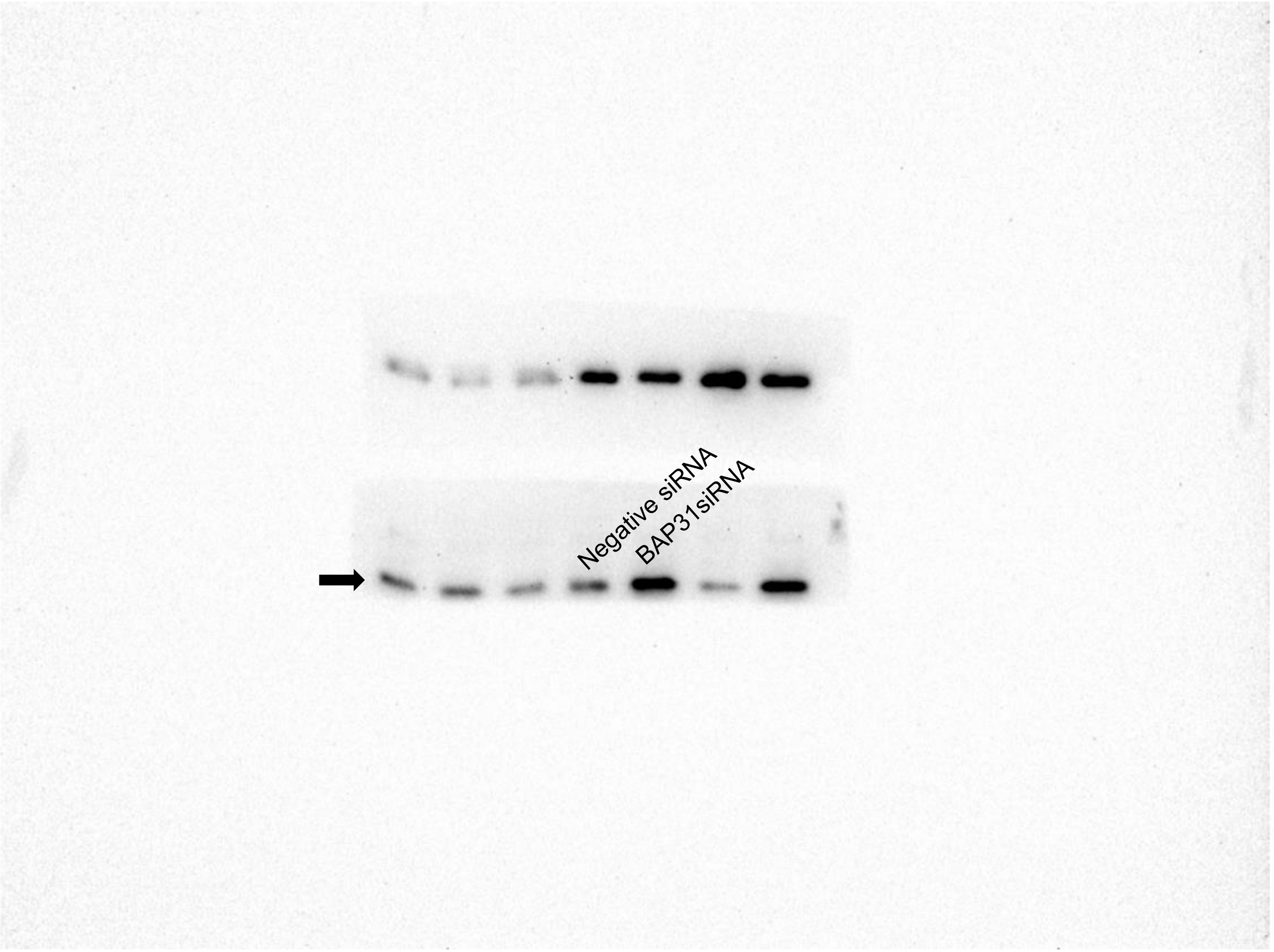

Fig2E bak

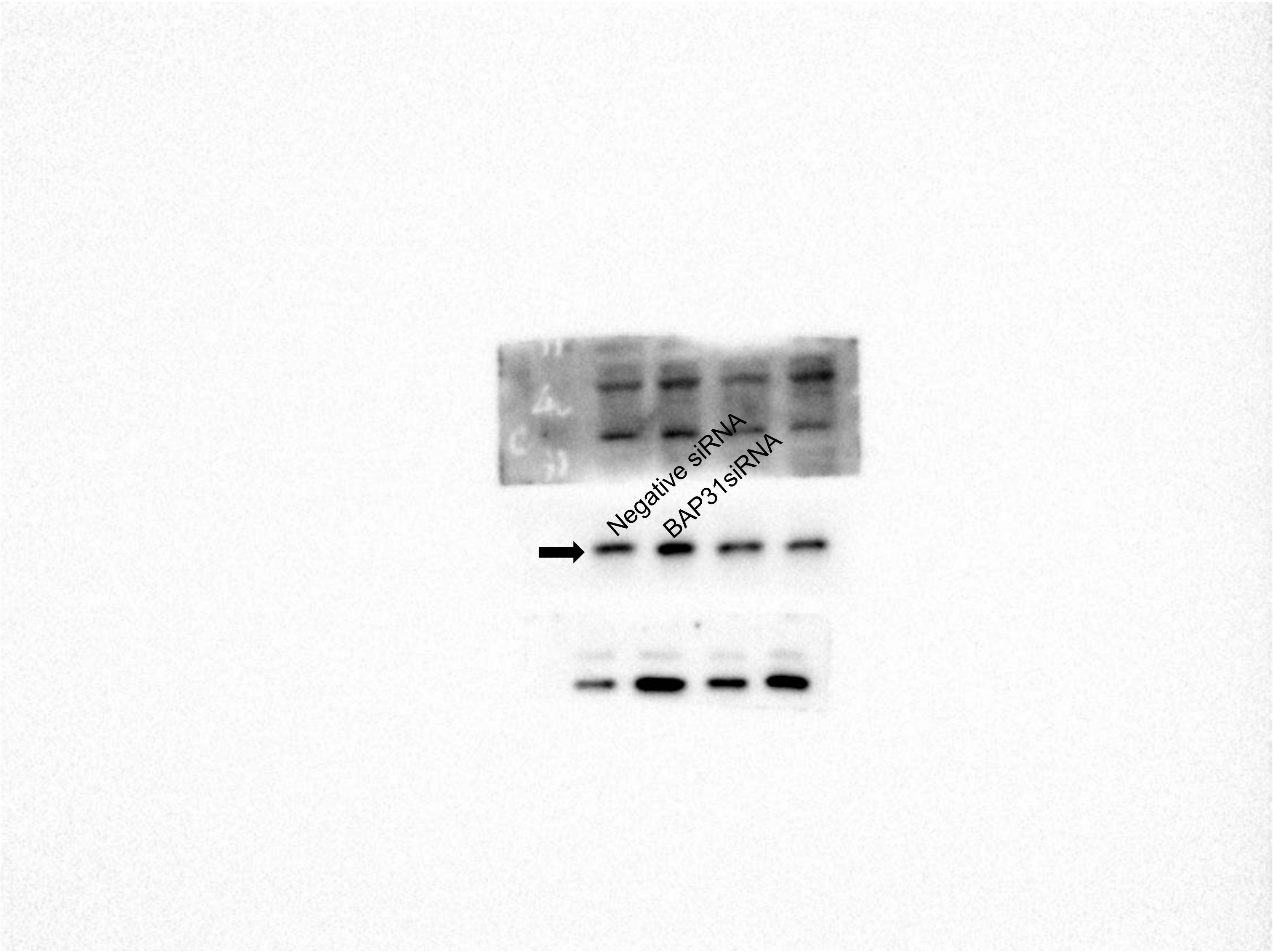

Fig2E BCL2

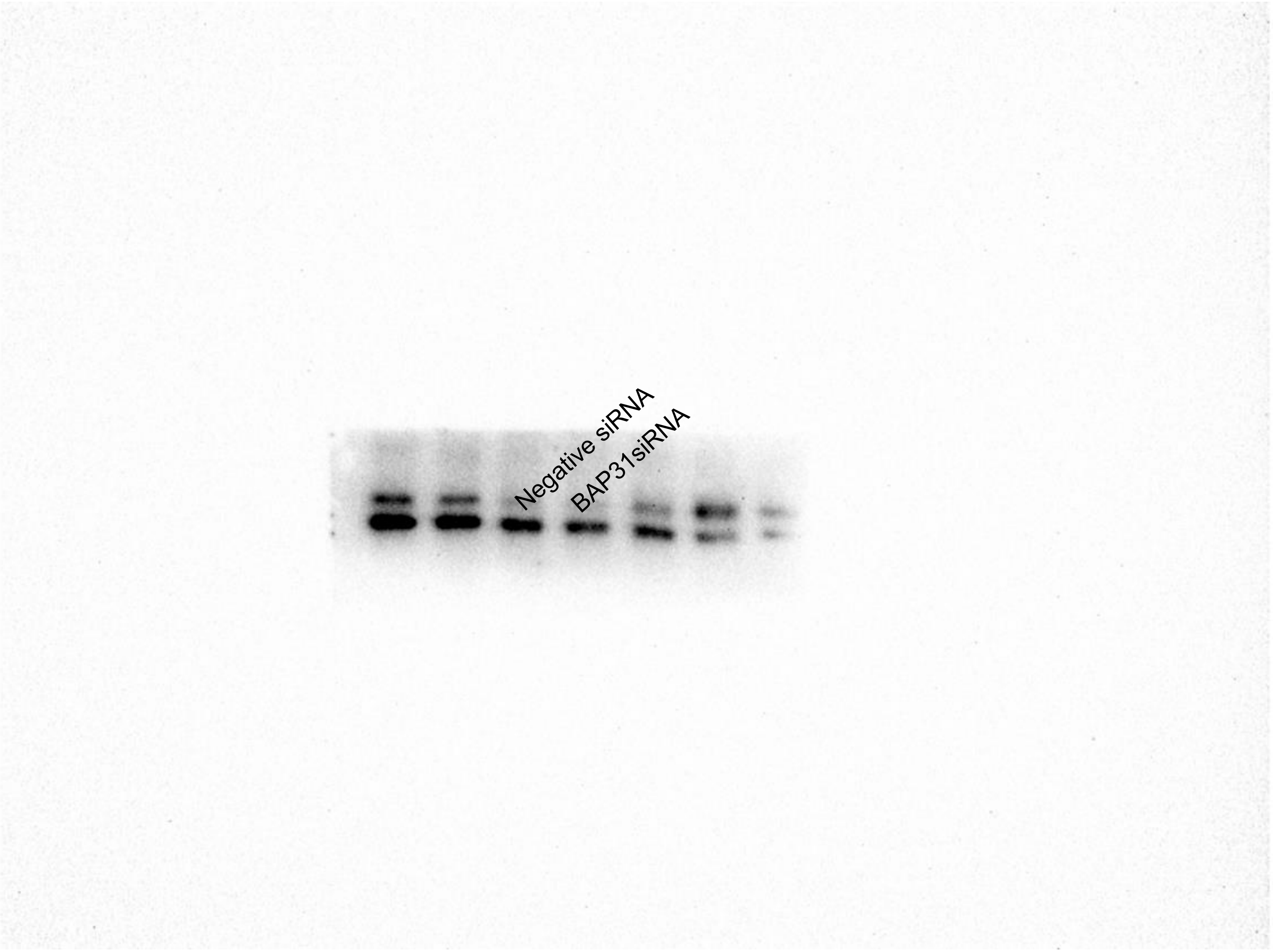

Fig2E Bid

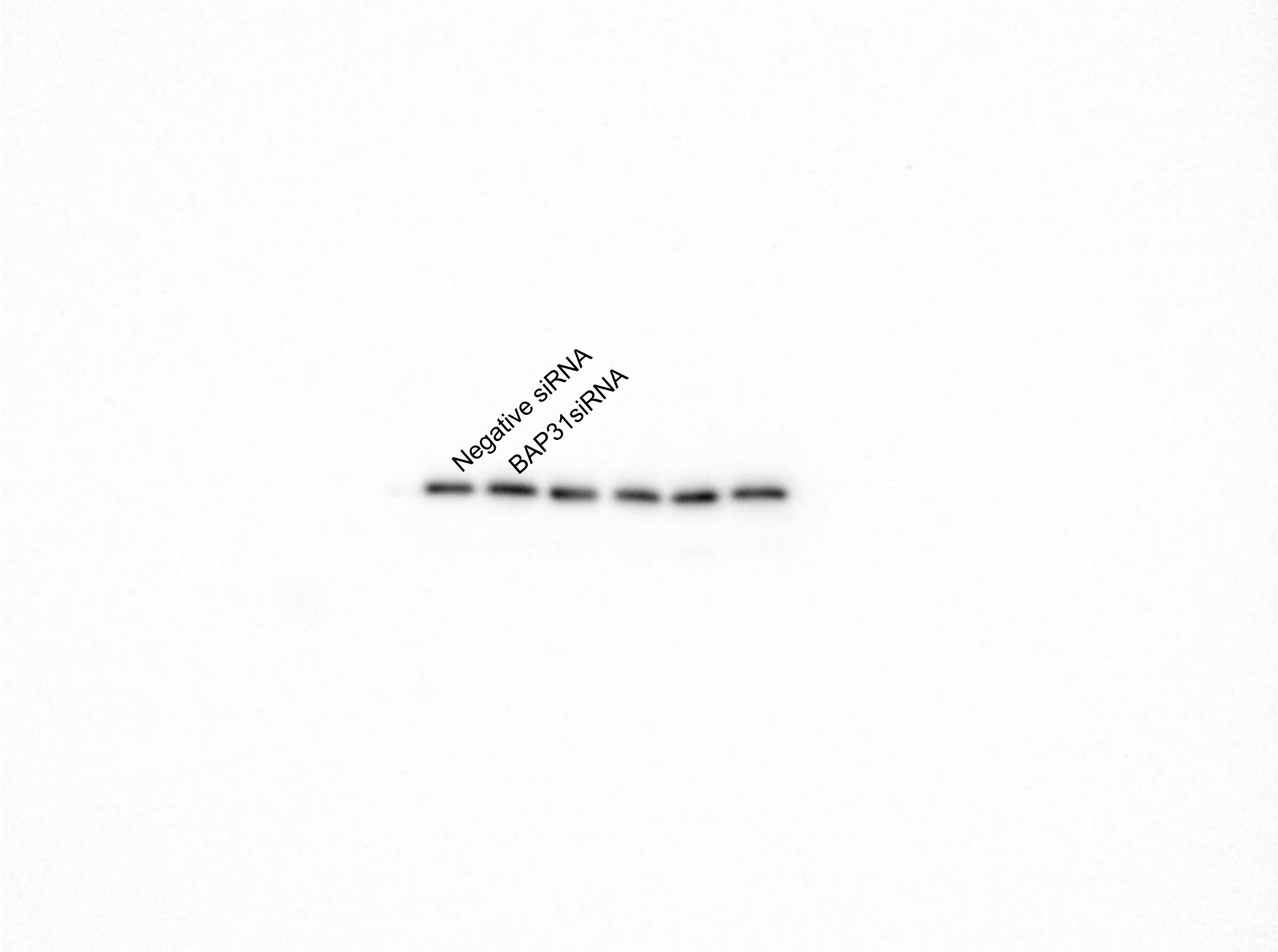

Fig2E MLKL

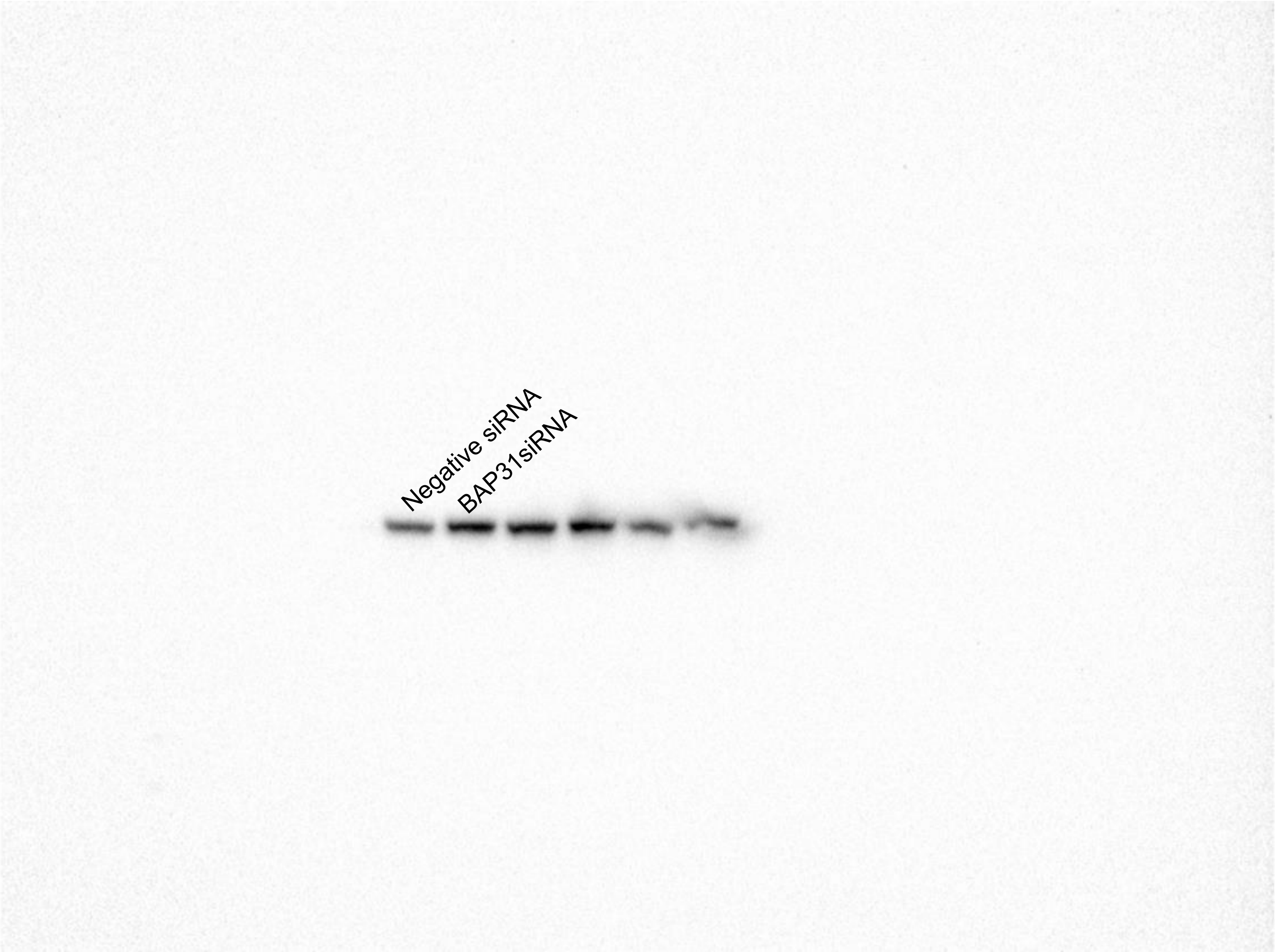

Fig2E BAP31

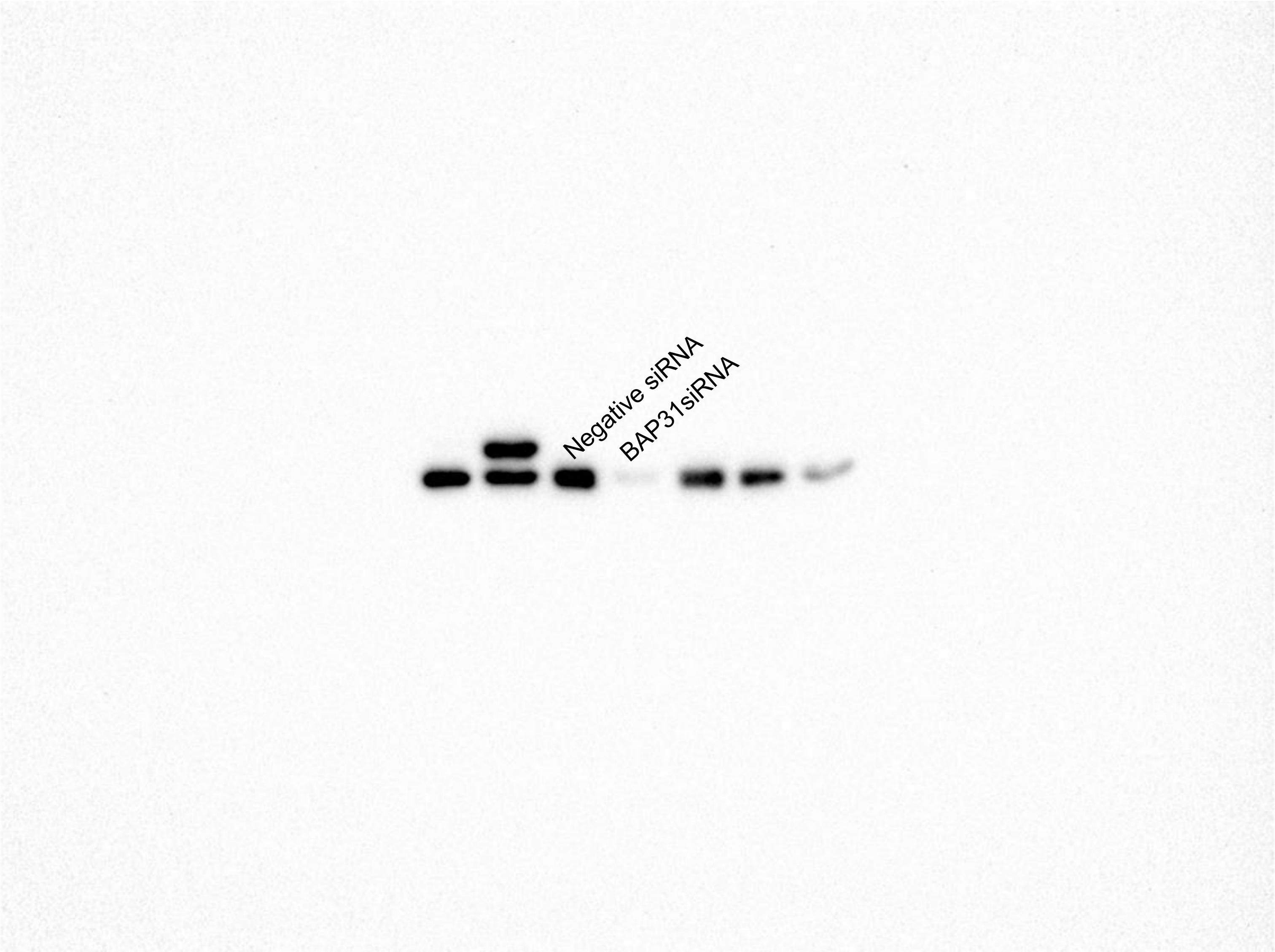

Fig2E gapdh

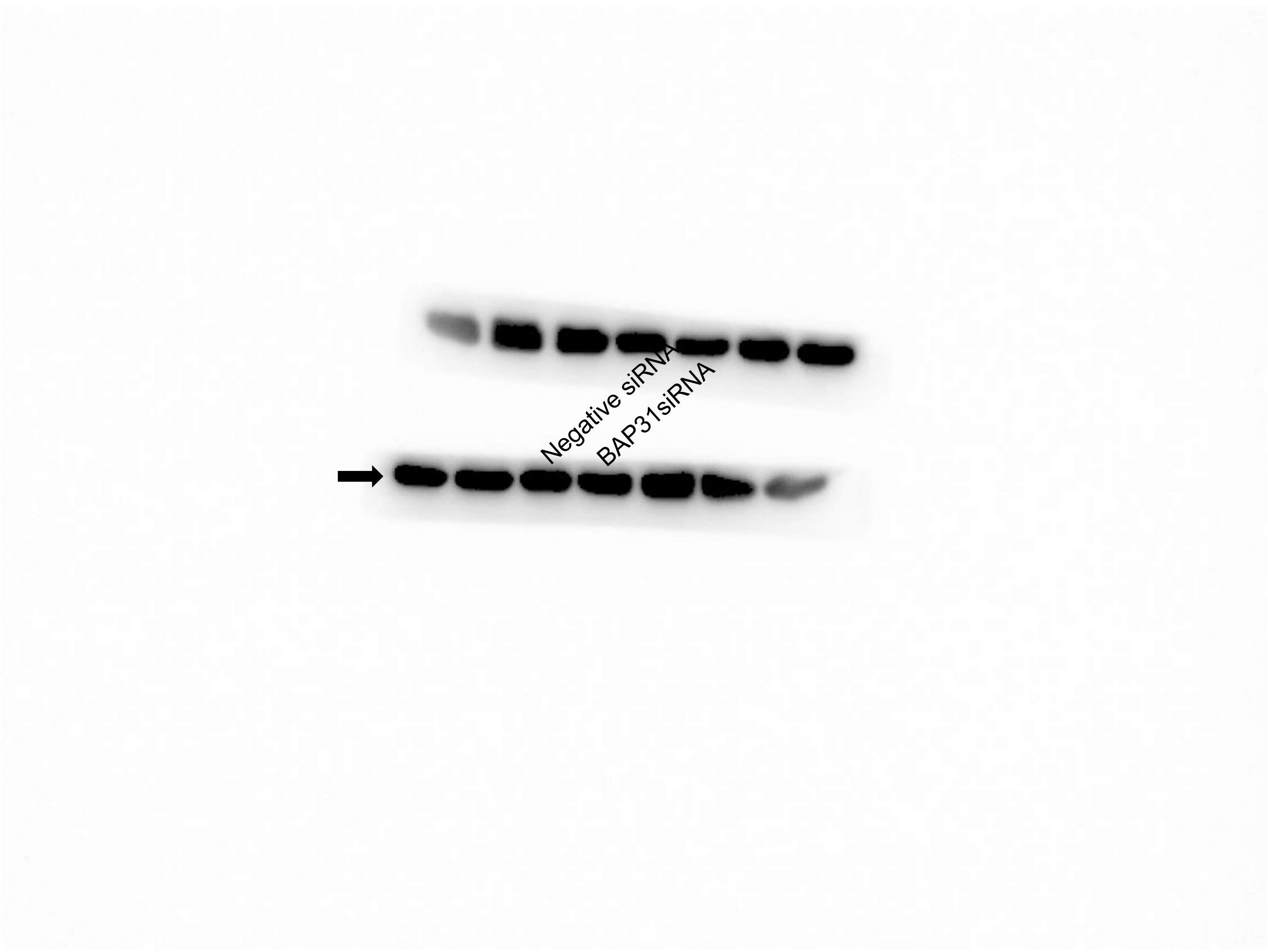

Supplement: Supplementary file 1 [file DataSheet_1.zip › raw data/Fig 2/Fig 2 western blot.pdf]
