# Supplementary material for: BAP31 Regulates Wnt Signaling to Modulate Cell Migration in Lung Cancer
Source: Front Oncol. 2022 Mar 10;12:859195. doi: 10.3389/fonc.2022.859195 (PMC8960194; doi:10.3389/fonc.2022.859195)

Fig3A

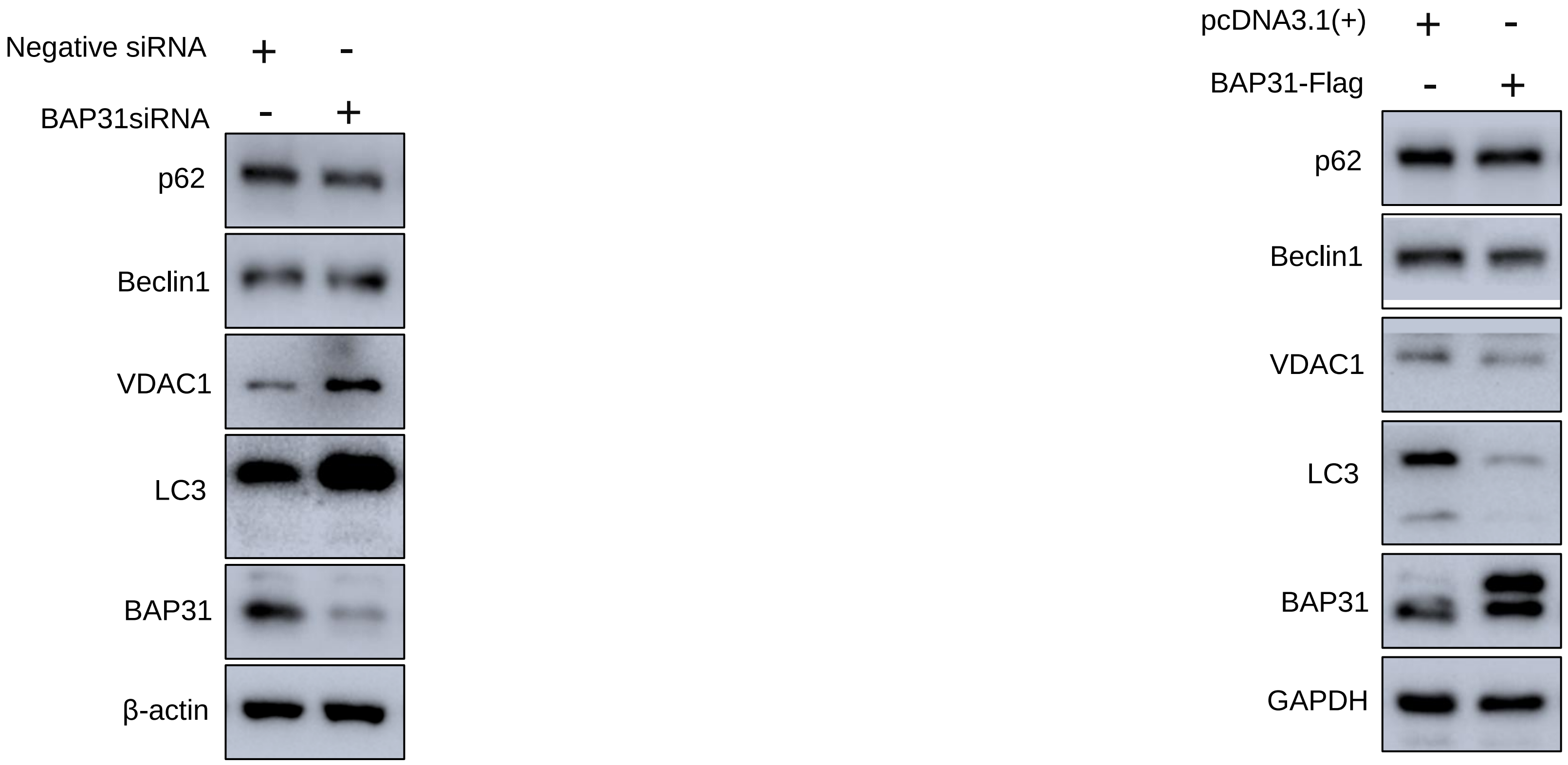
Negative siRNA + -
BAP31siRNA - +
p62
Beclin1
VDAC1
LC3
BAP31
β-actin
pcDNA3.1(+) + -
BAP31-Flag - +
p62
Beclin1
VDAC1
LC3
BAP31
GAPDH

Fig3A p62

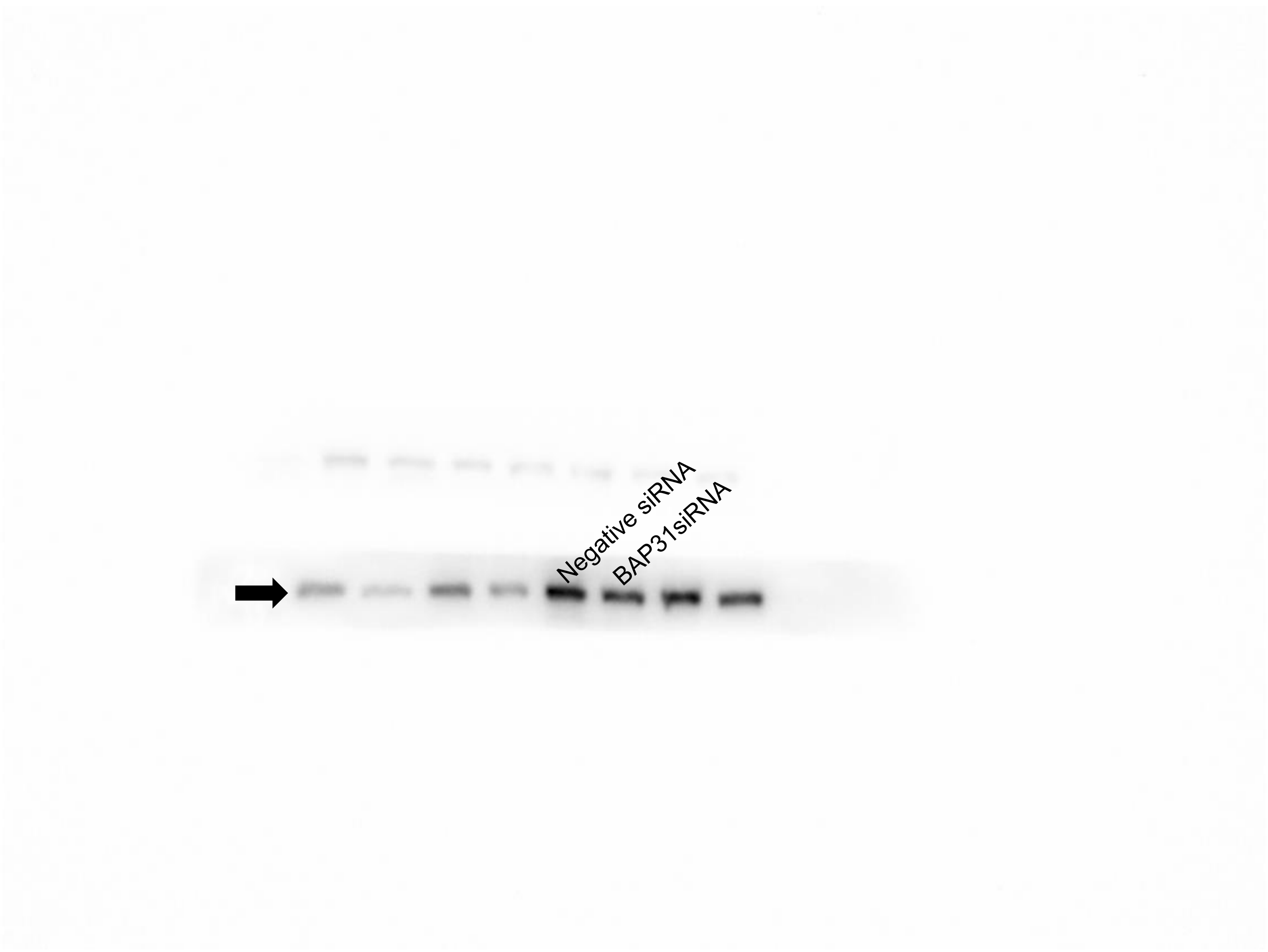

Fig3A beclin1

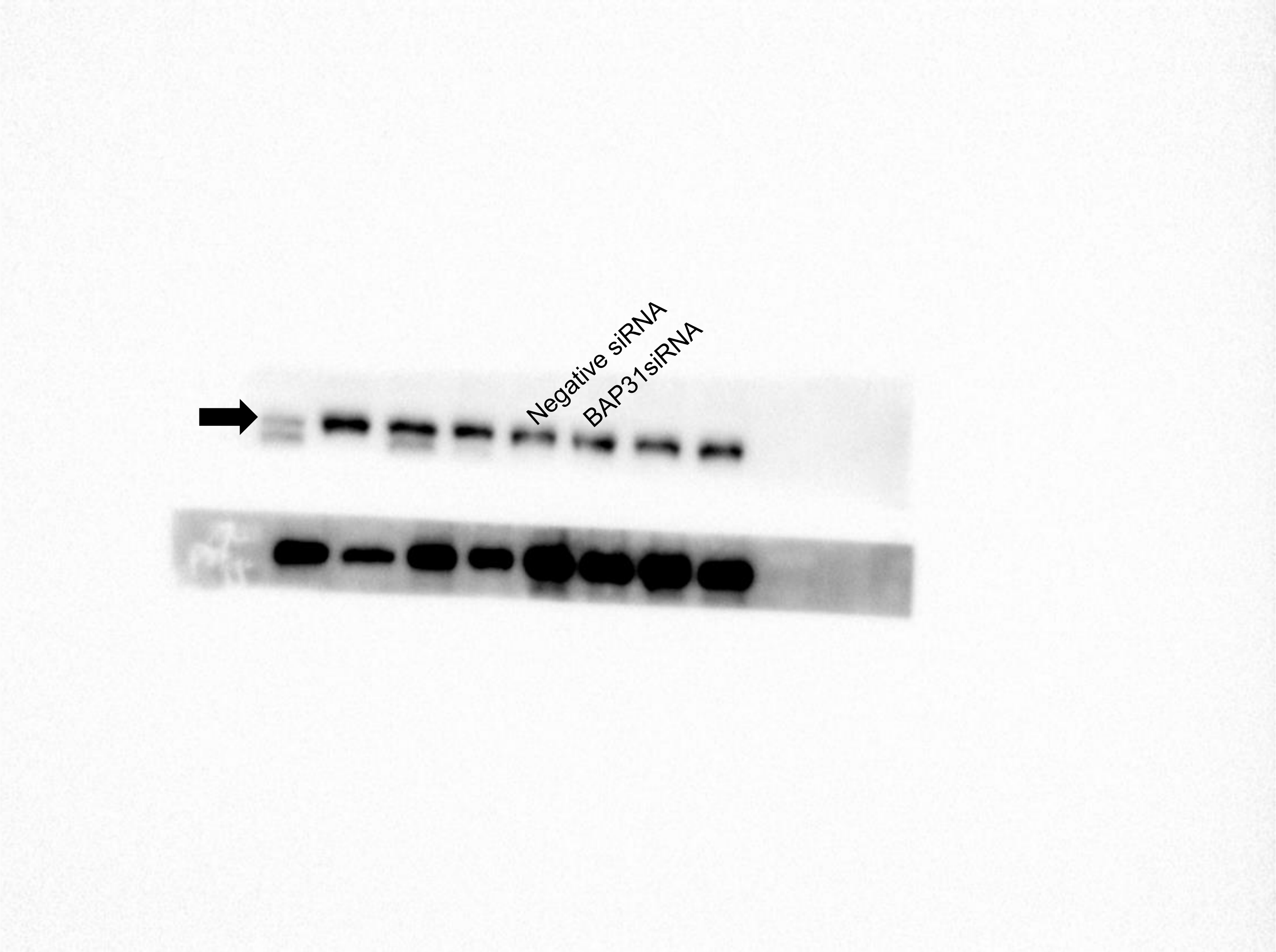

Fig3A vdac1

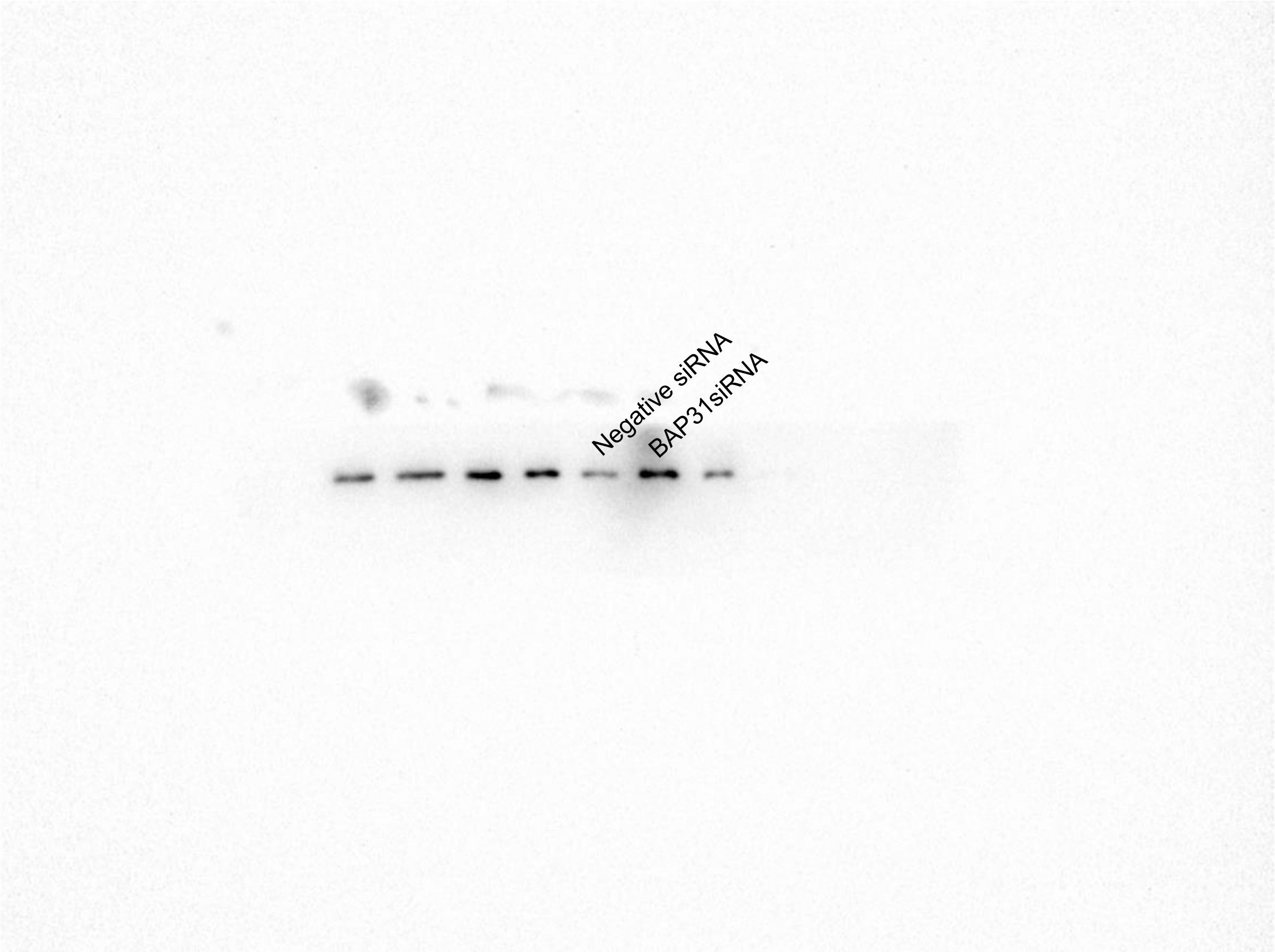

Fig3A lc3

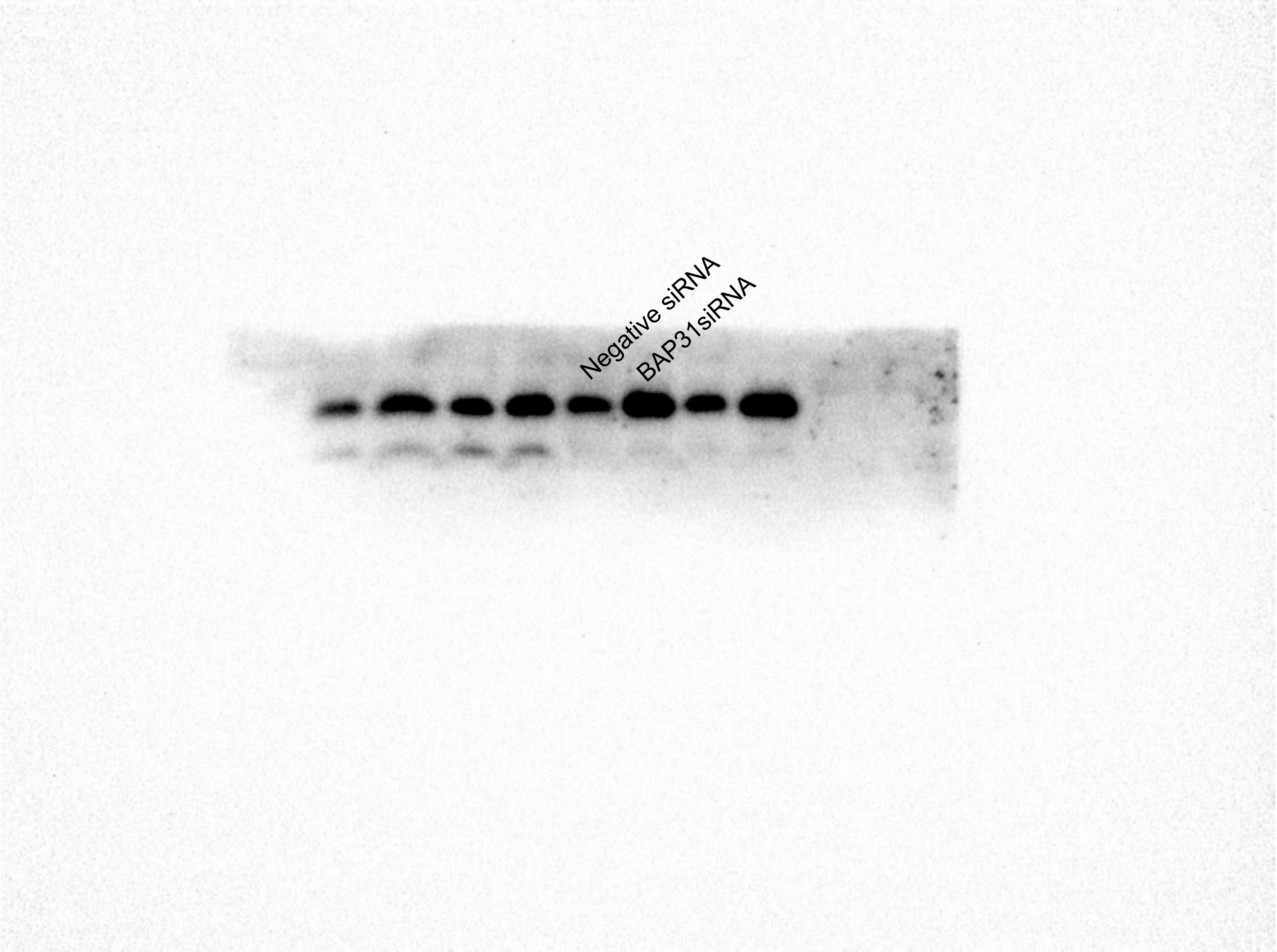

Fig3A bap31

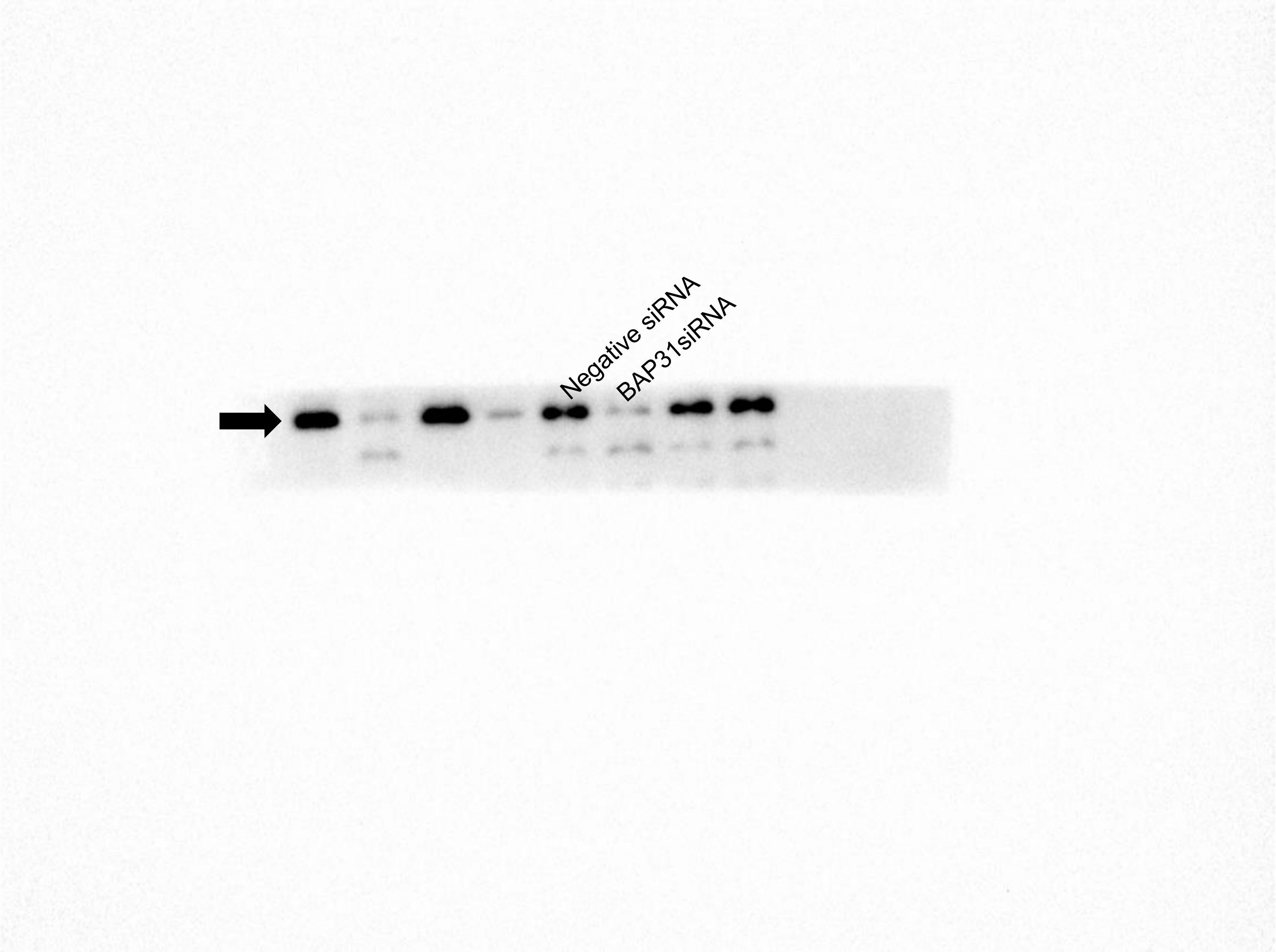

Fig3A beta-actin

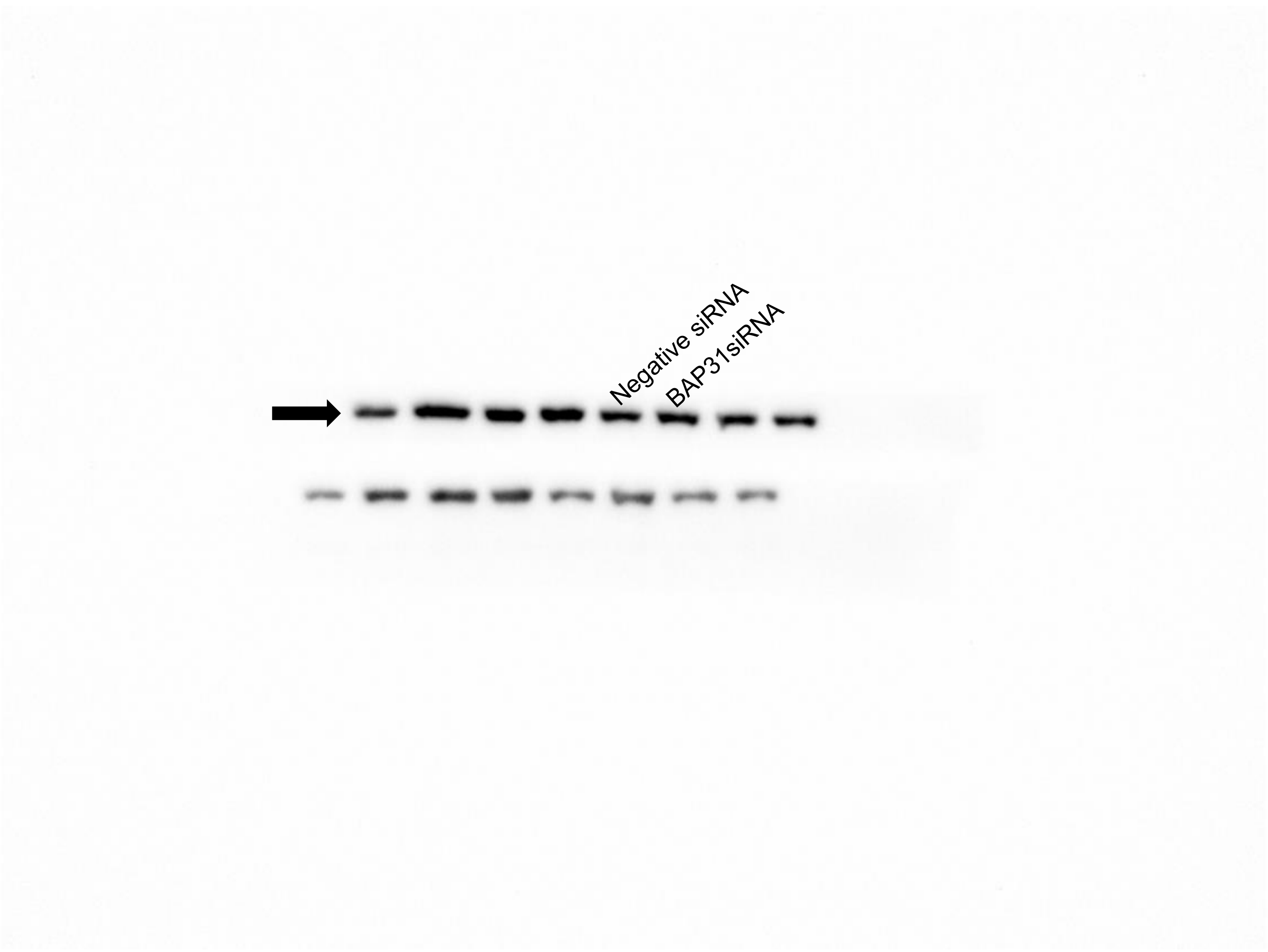

Fig3A p62

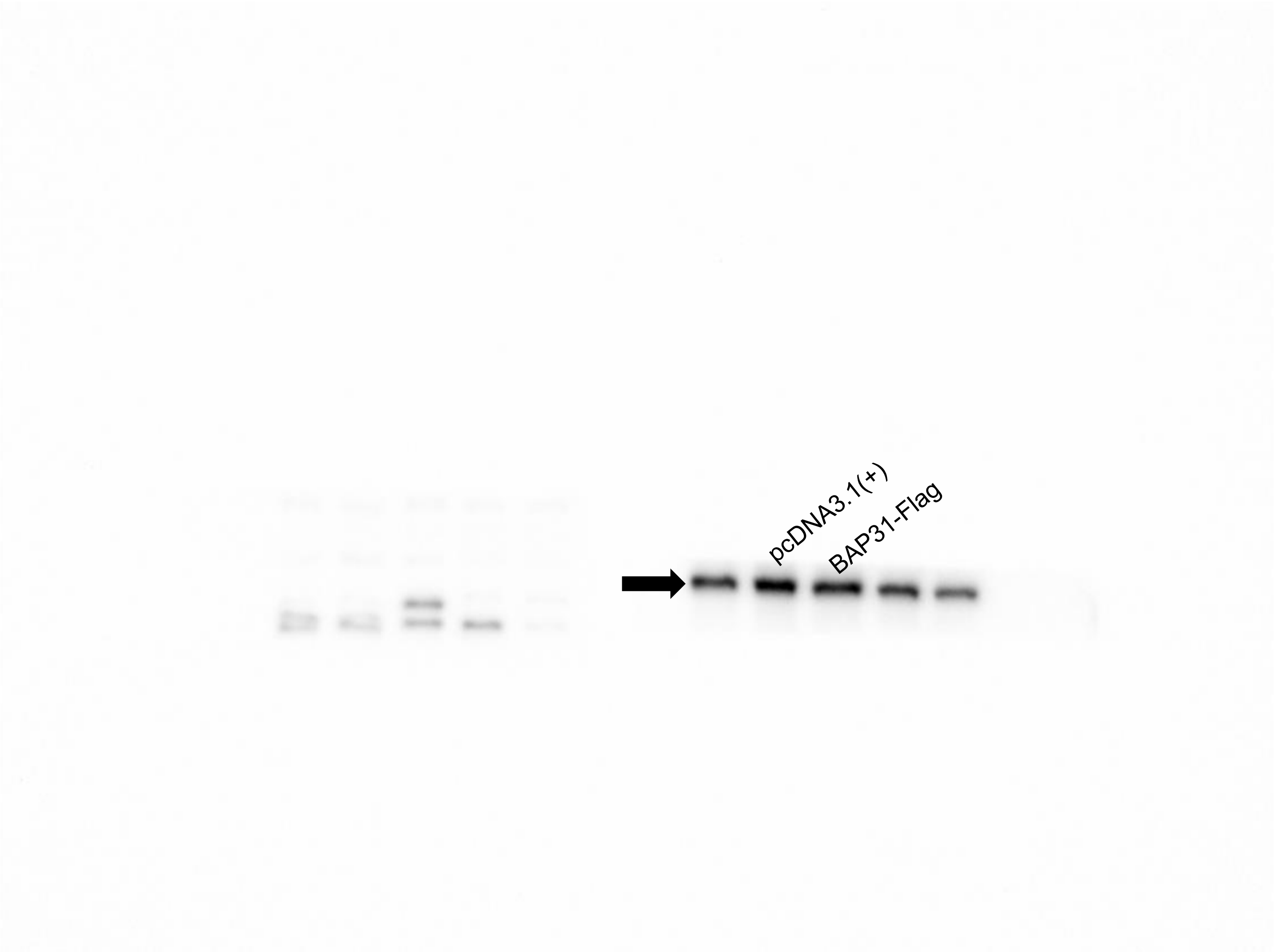

Fig3A beclin1

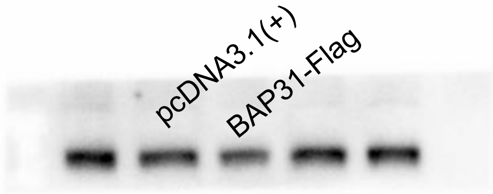

Fig3A vdac1

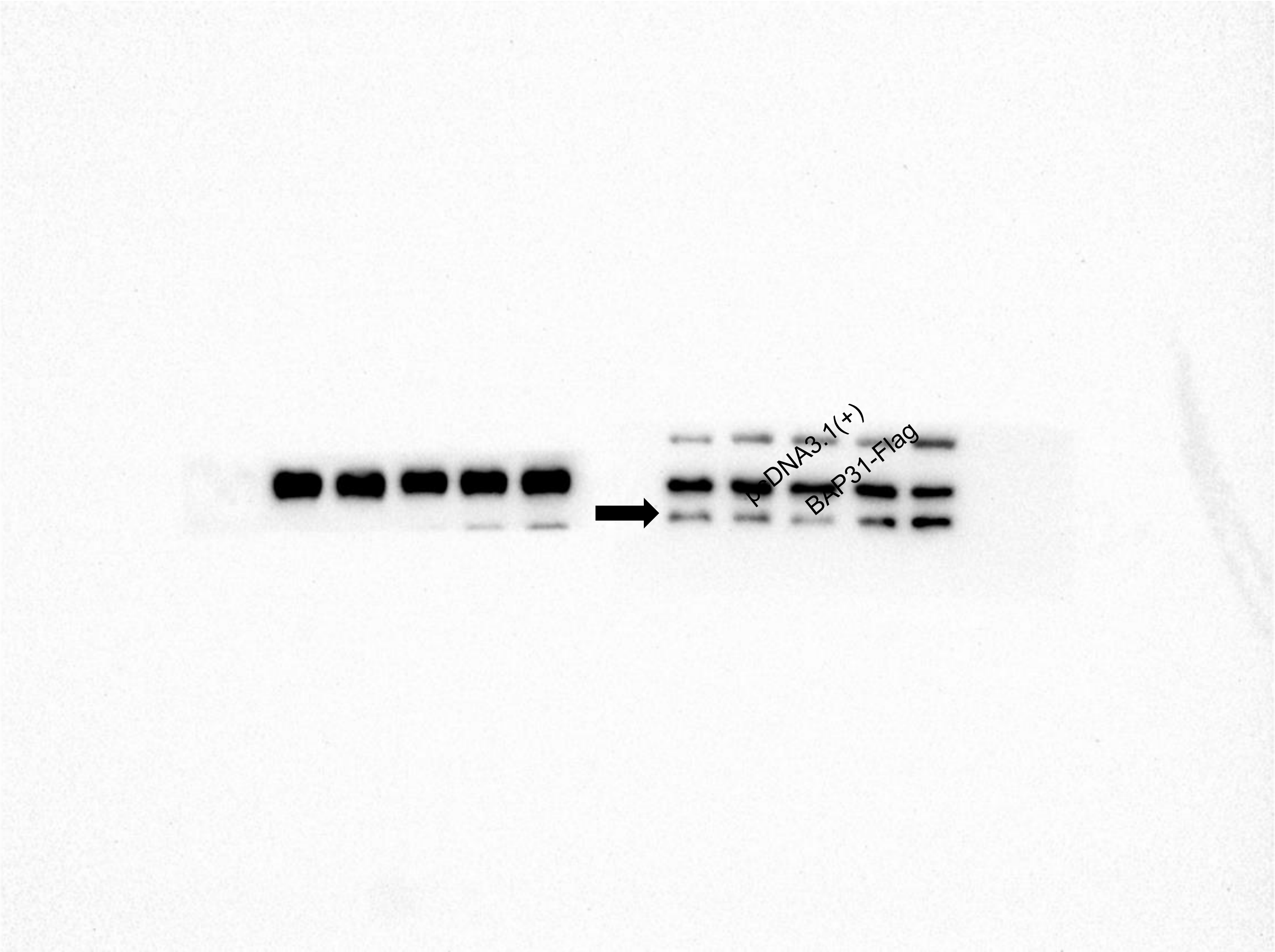

Fig3A lc3

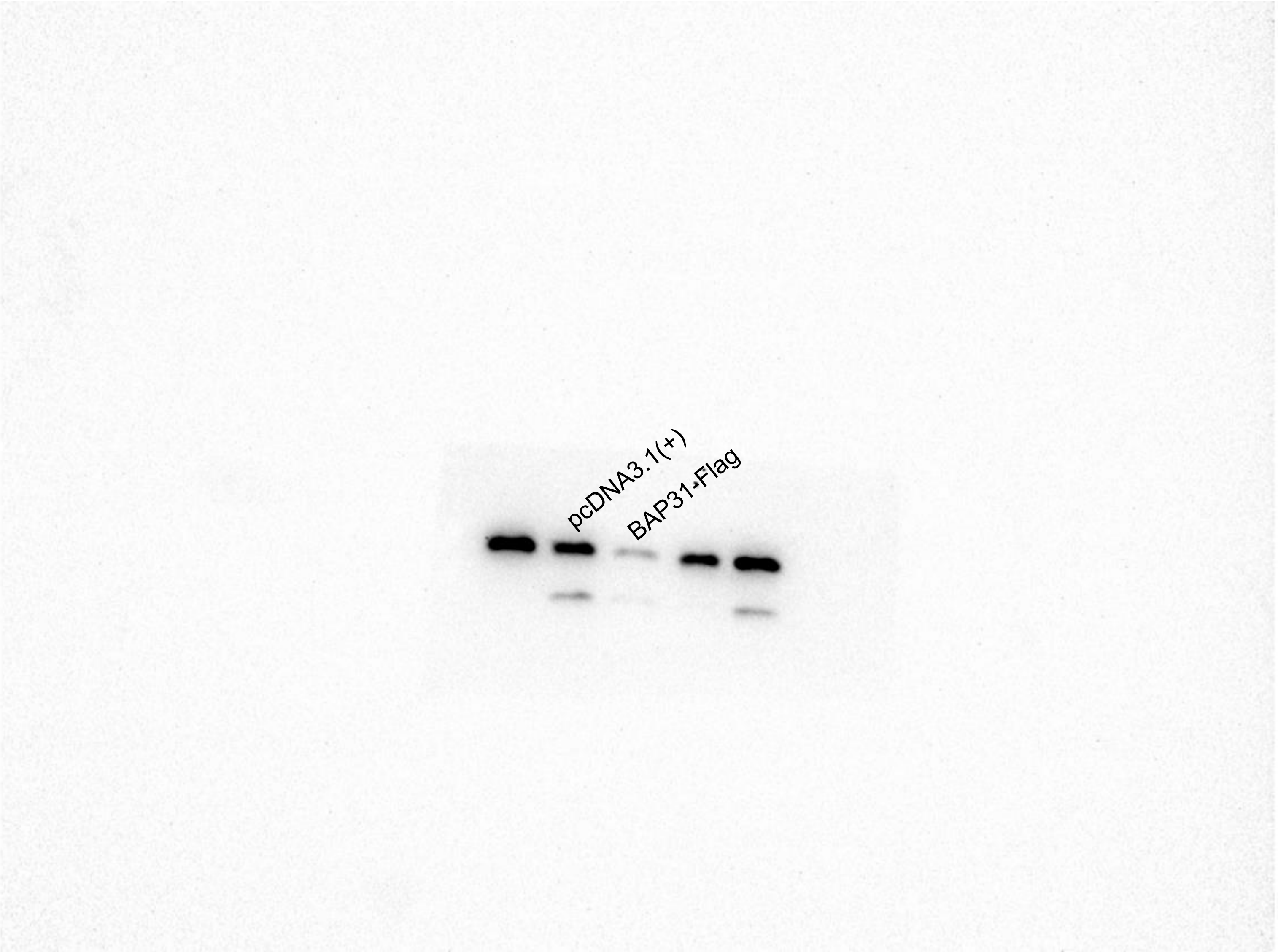

Fig3A bap31

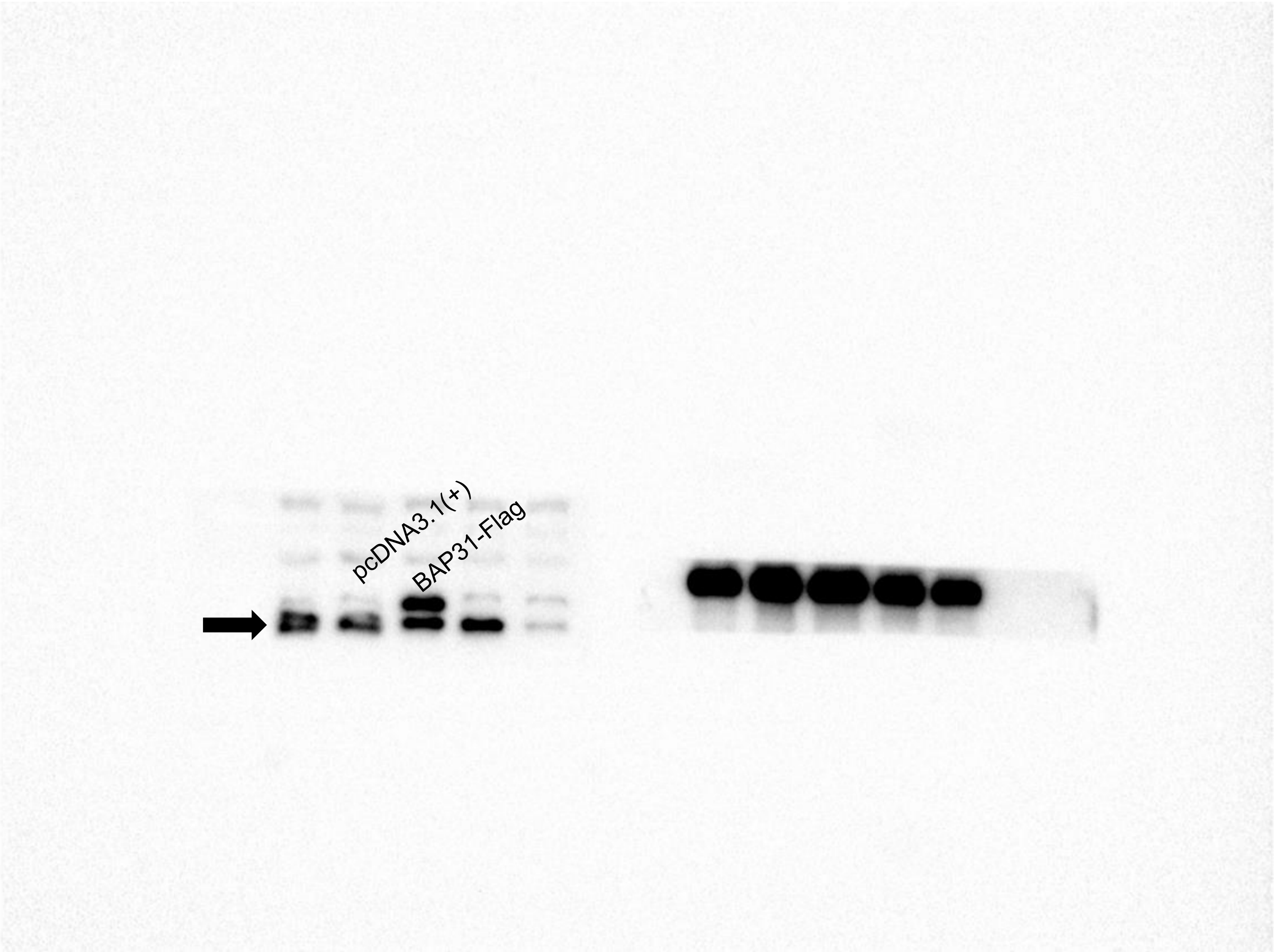

Fig3A gapdh

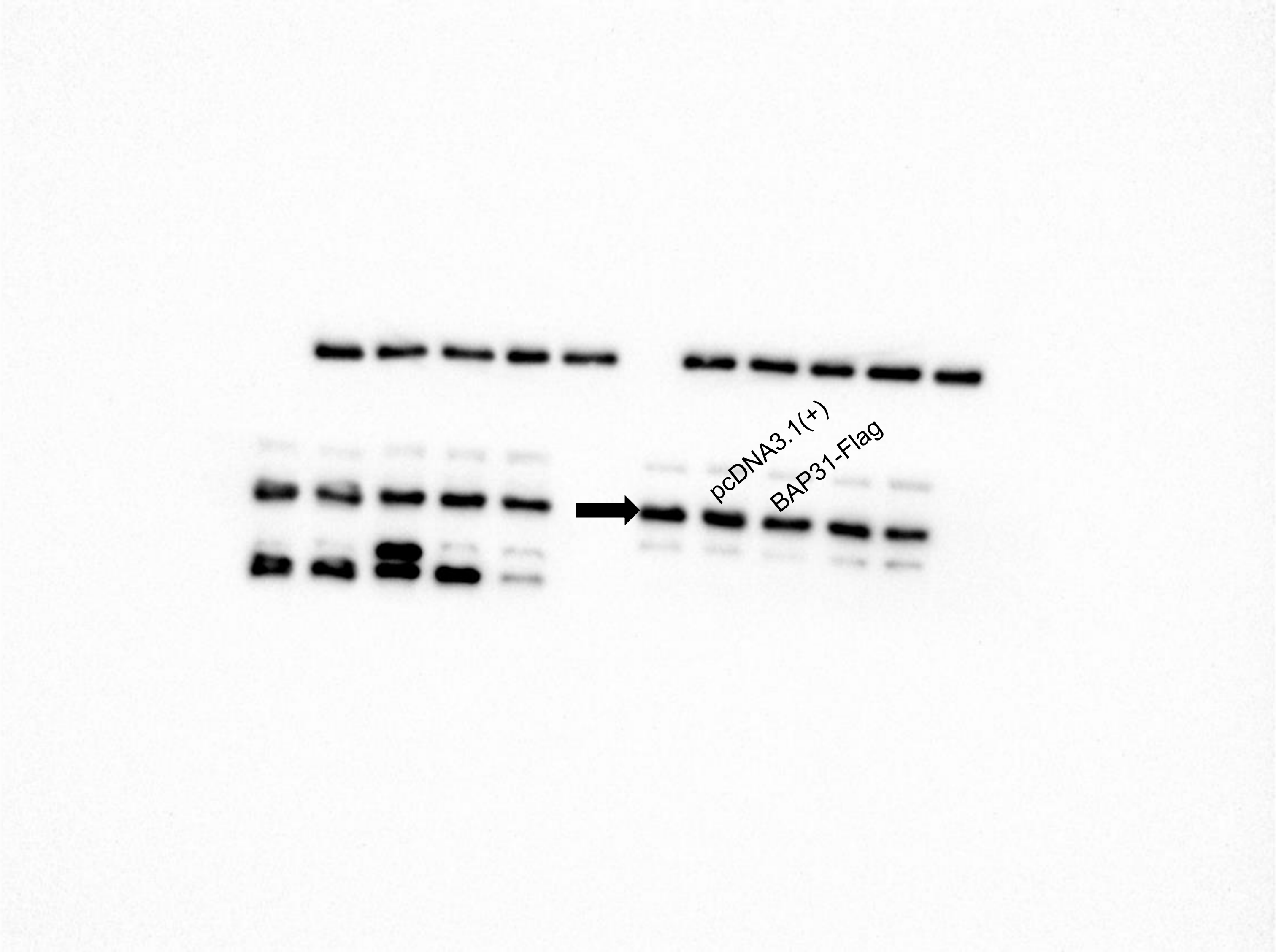

Fig3D

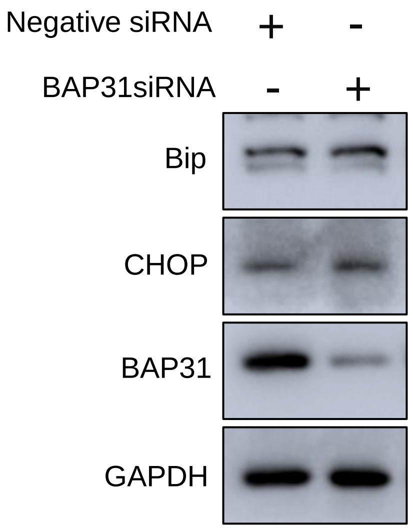

Fig3D bip

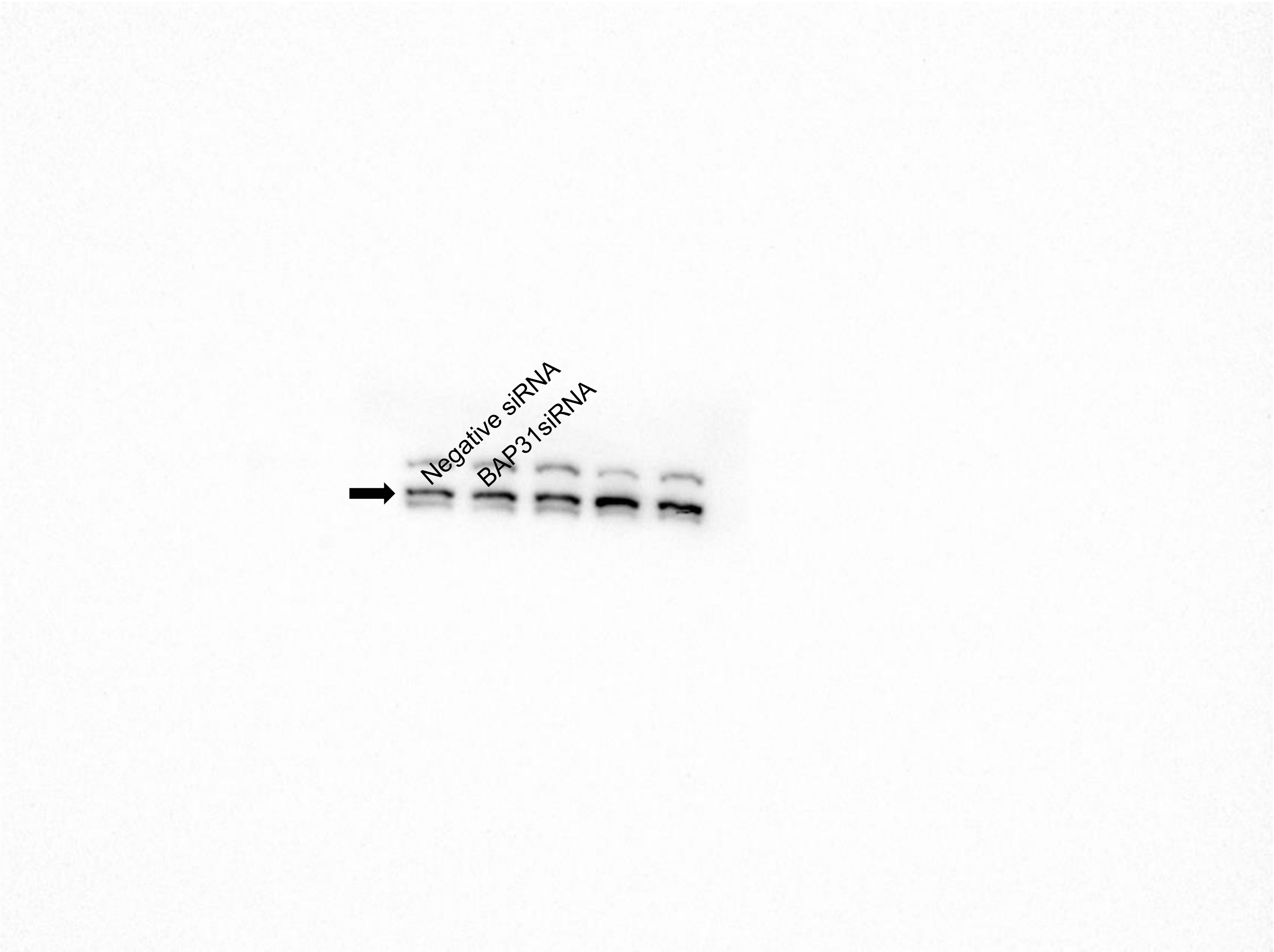

Fig3D chop

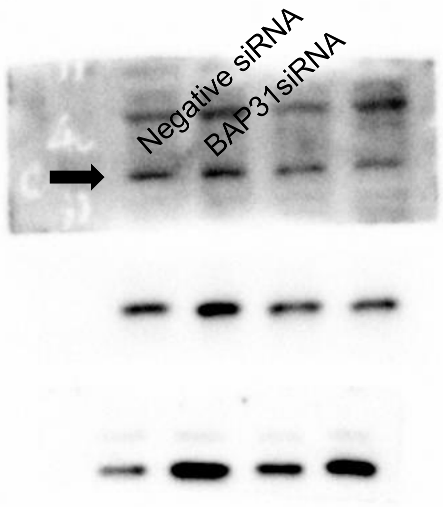

Fig3D bap31

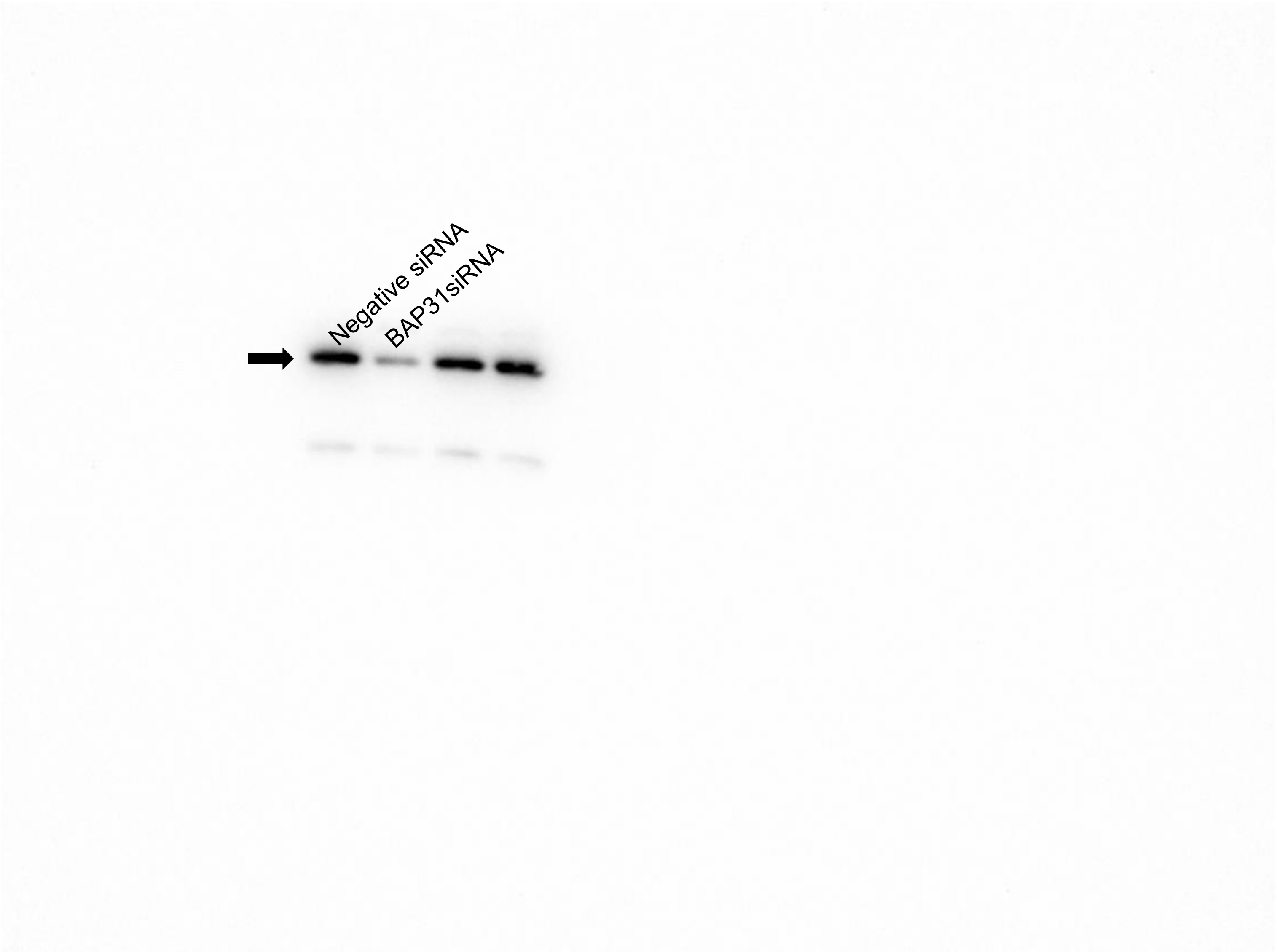

Fig3D gapdh

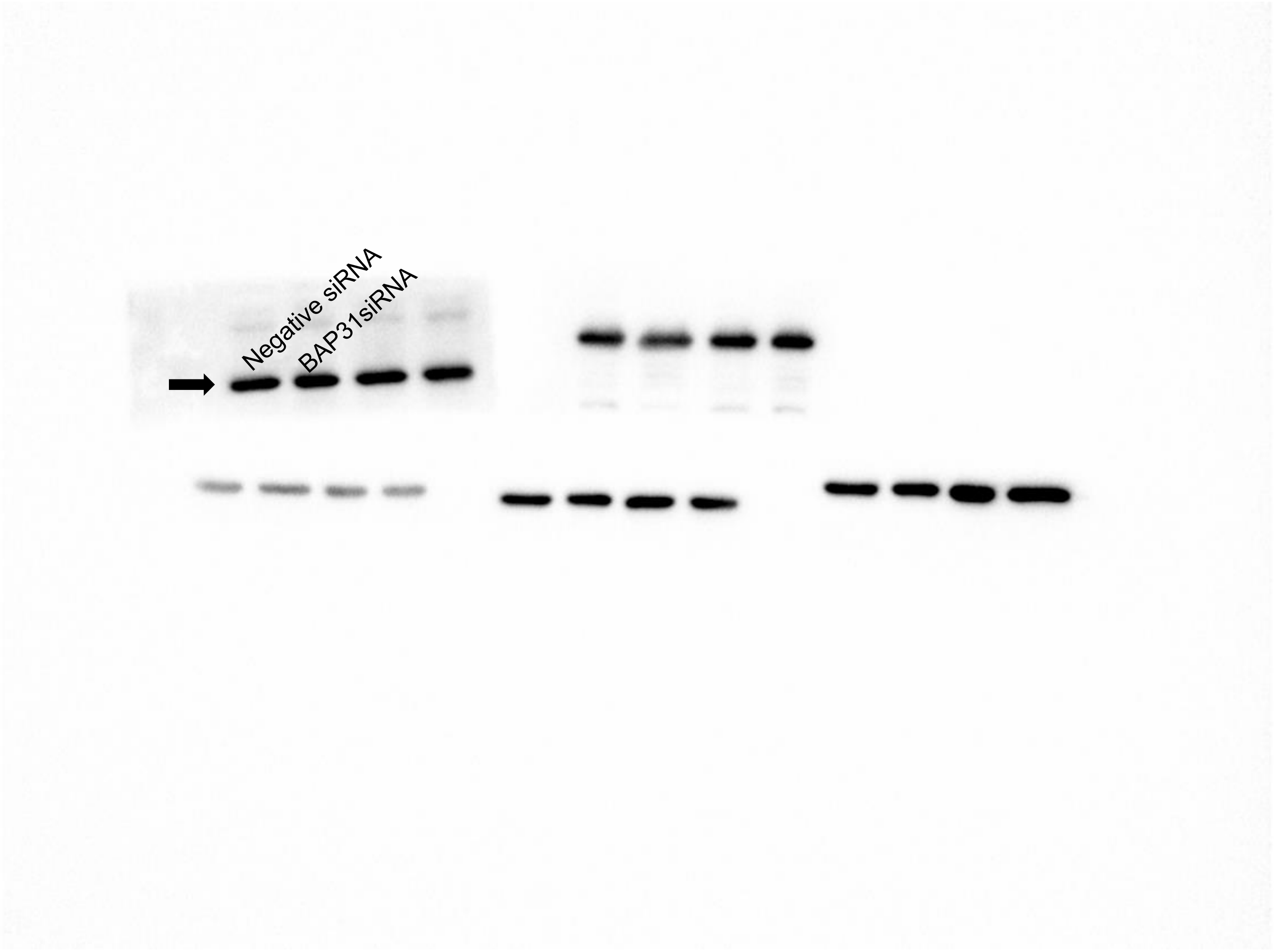

Supplement: Supplementary file 1 [file DataSheet_1.zip › raw data/Fig 3/Fig 3 western blot.pdf]
