# Supplementary material for: BAP31 Regulates Wnt Signaling to Modulate Cell Migration in Lung Cancer
Source: Front Oncol. 2022 Mar 10;12:859195. doi: 10.3389/fonc.2022.859195 (PMC8960194; doi:10.3389/fonc.2022.859195)

Fig4C

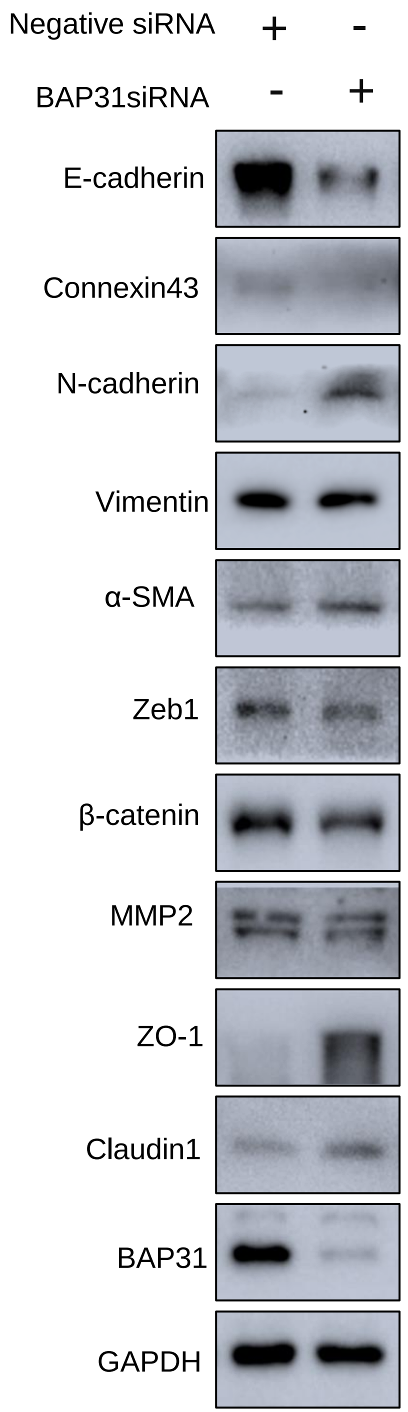

Negative siRNA + -
BAP31siRNA - +
E-cadherin
Connexin43
N-cadherin
Vimentin
α-SMA
Zeb1
β-catenin
MMP2
ZO-1
Claudin1
BAP31
GAPDH

Fig4C e-cadherin

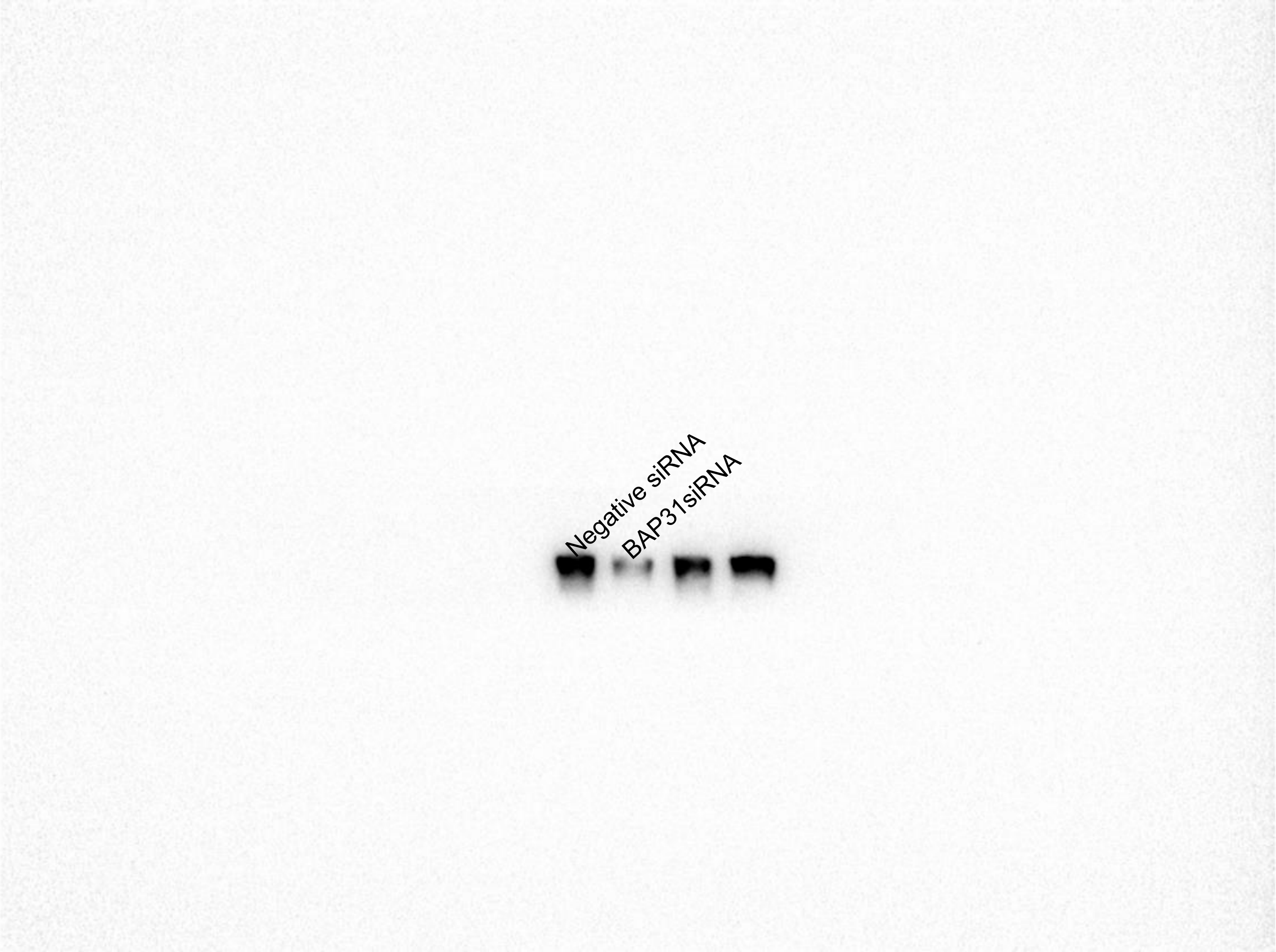

Fig4C connexin43

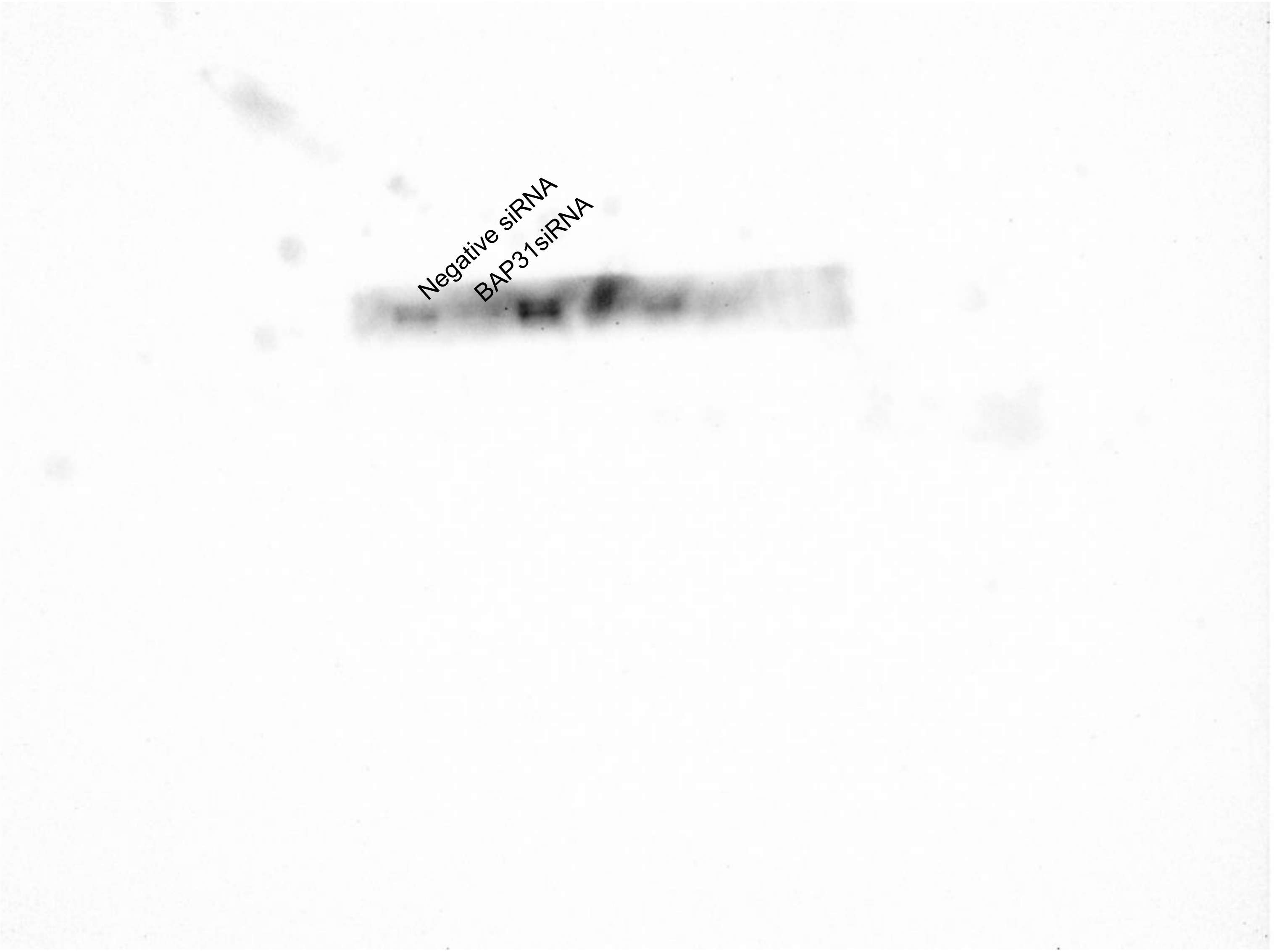

Fig4C n-cadherin

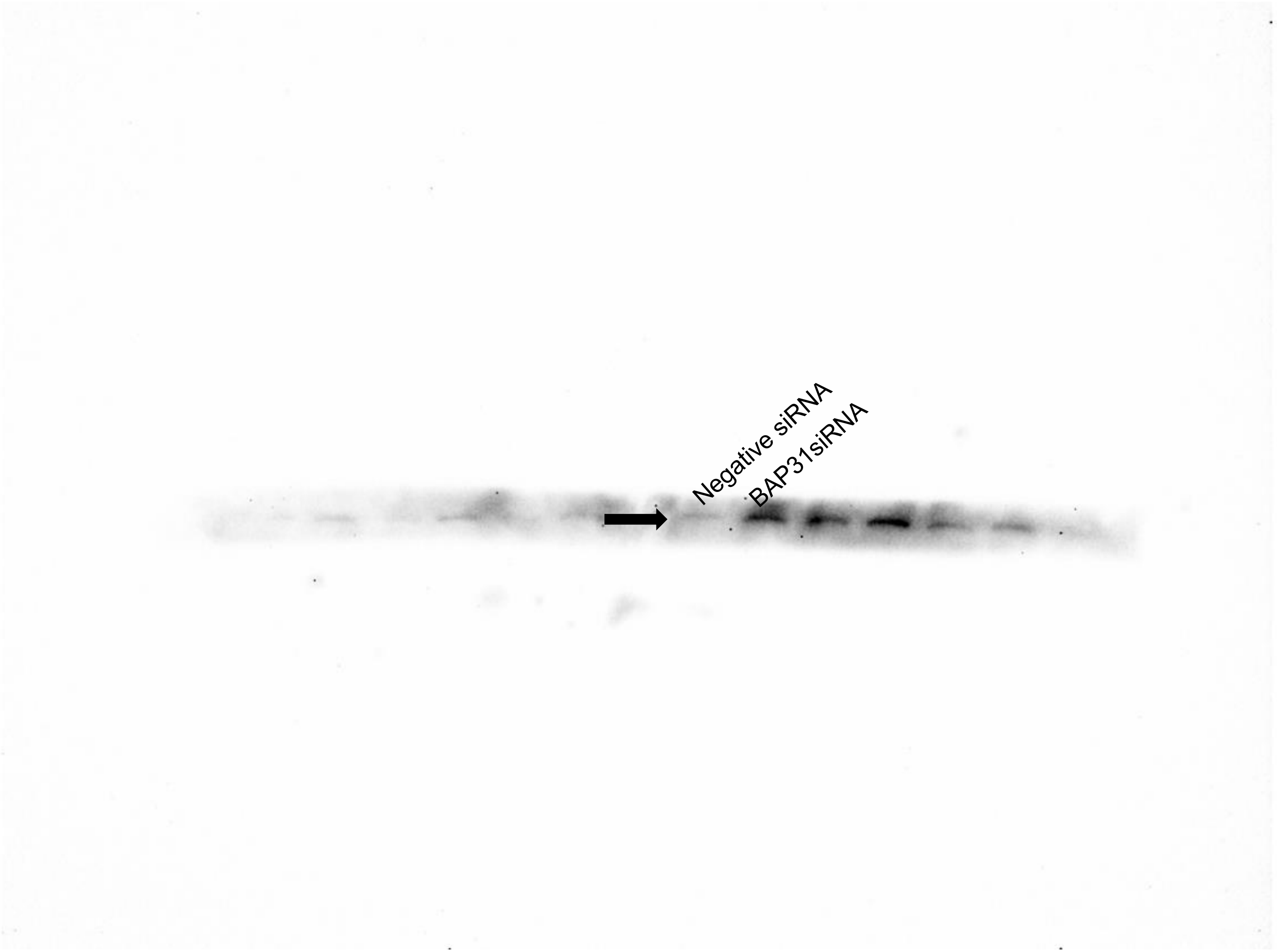

Fig4C vimentin

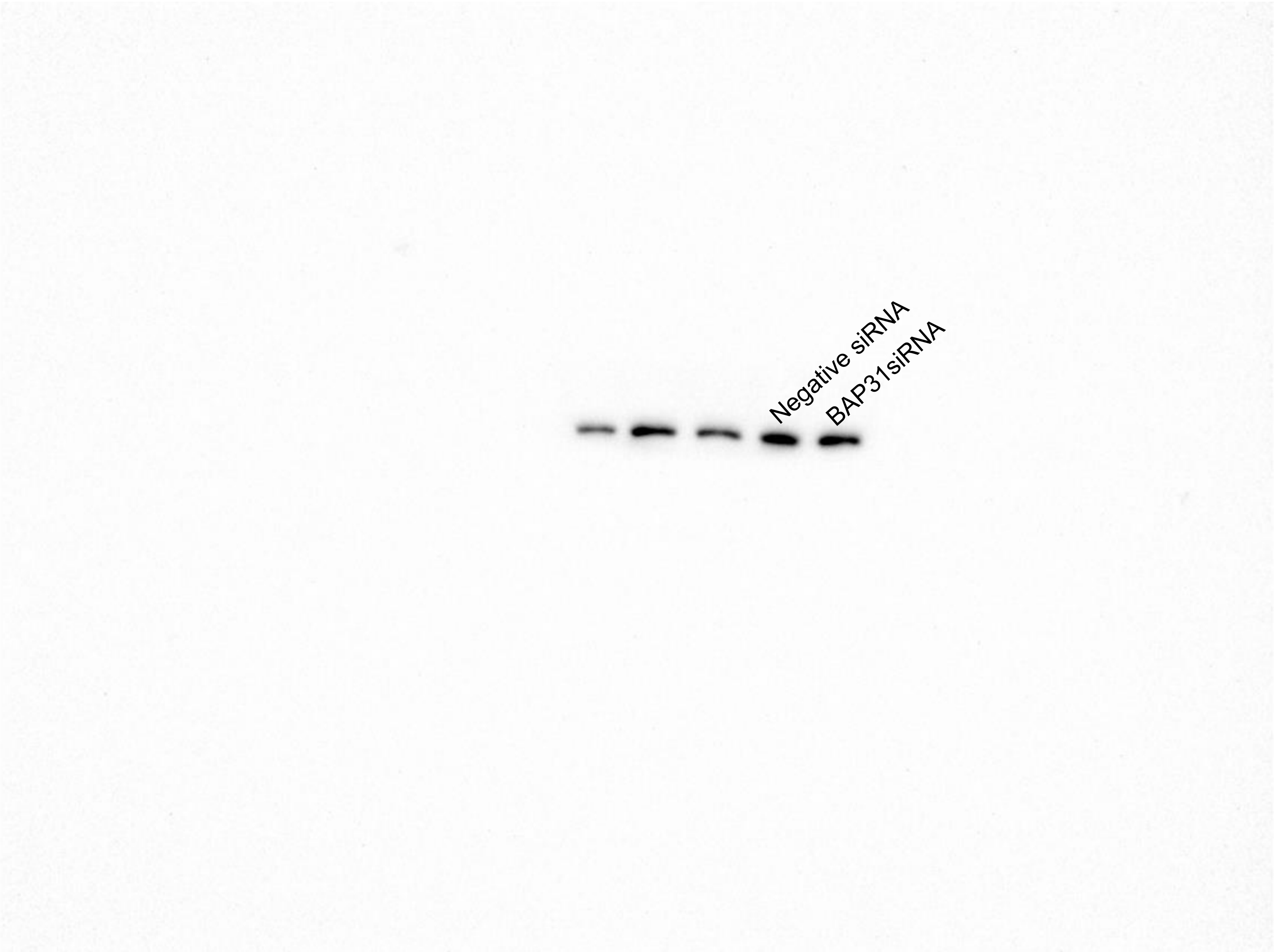

Fig4C a-SMA

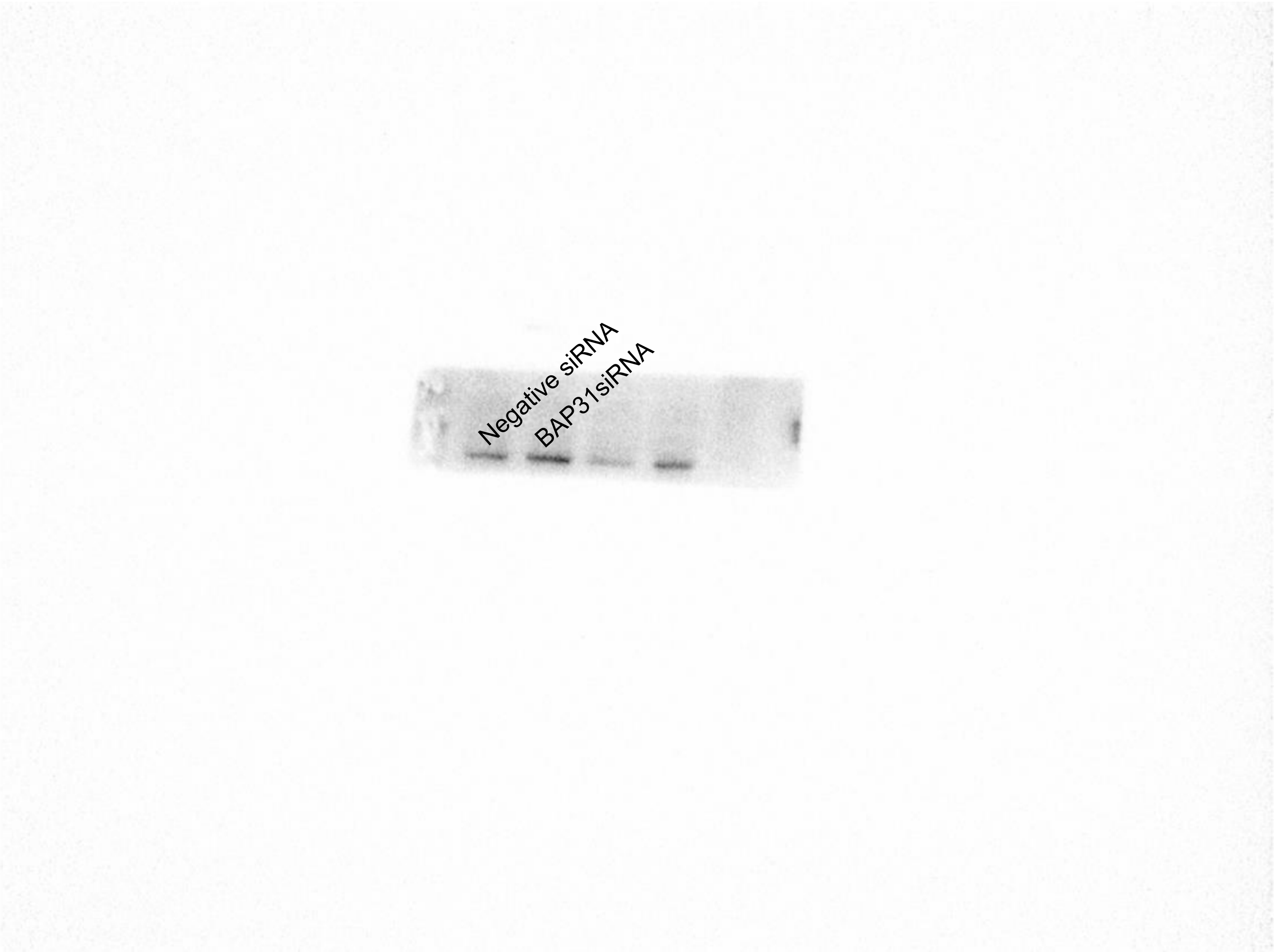

Fig4C ZEB1

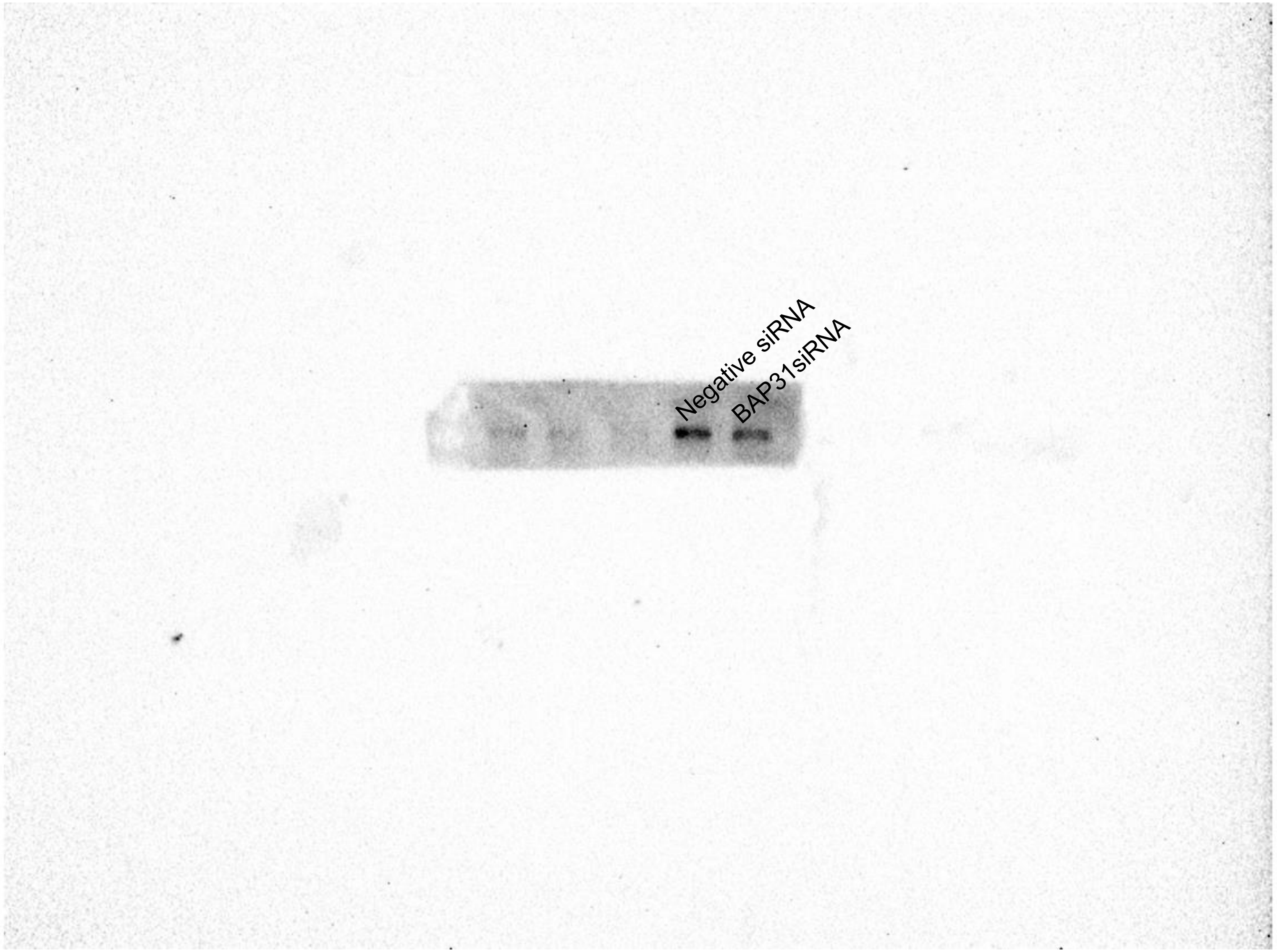

Fig4C beta-catenin

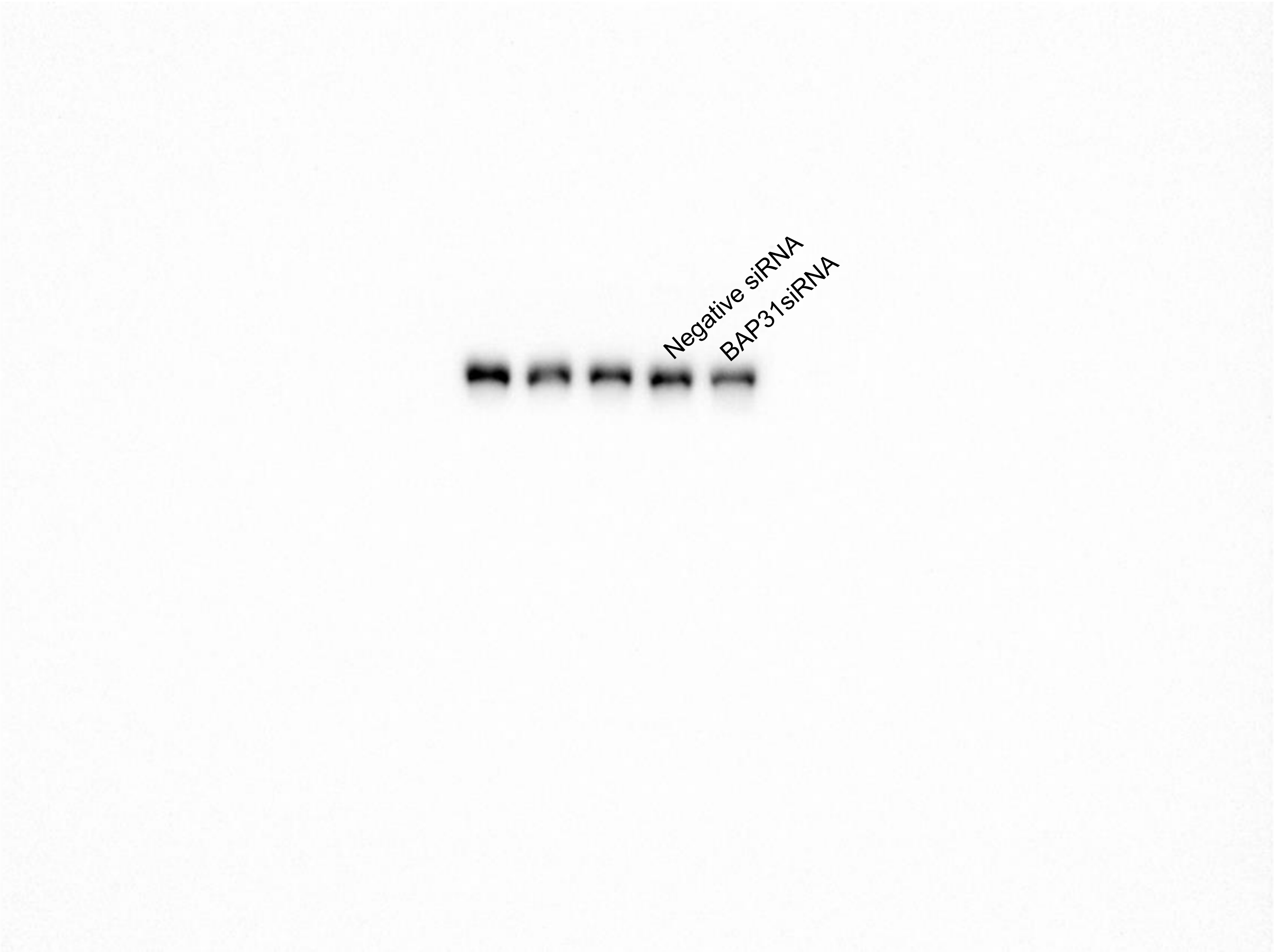

Fig4C mmp2

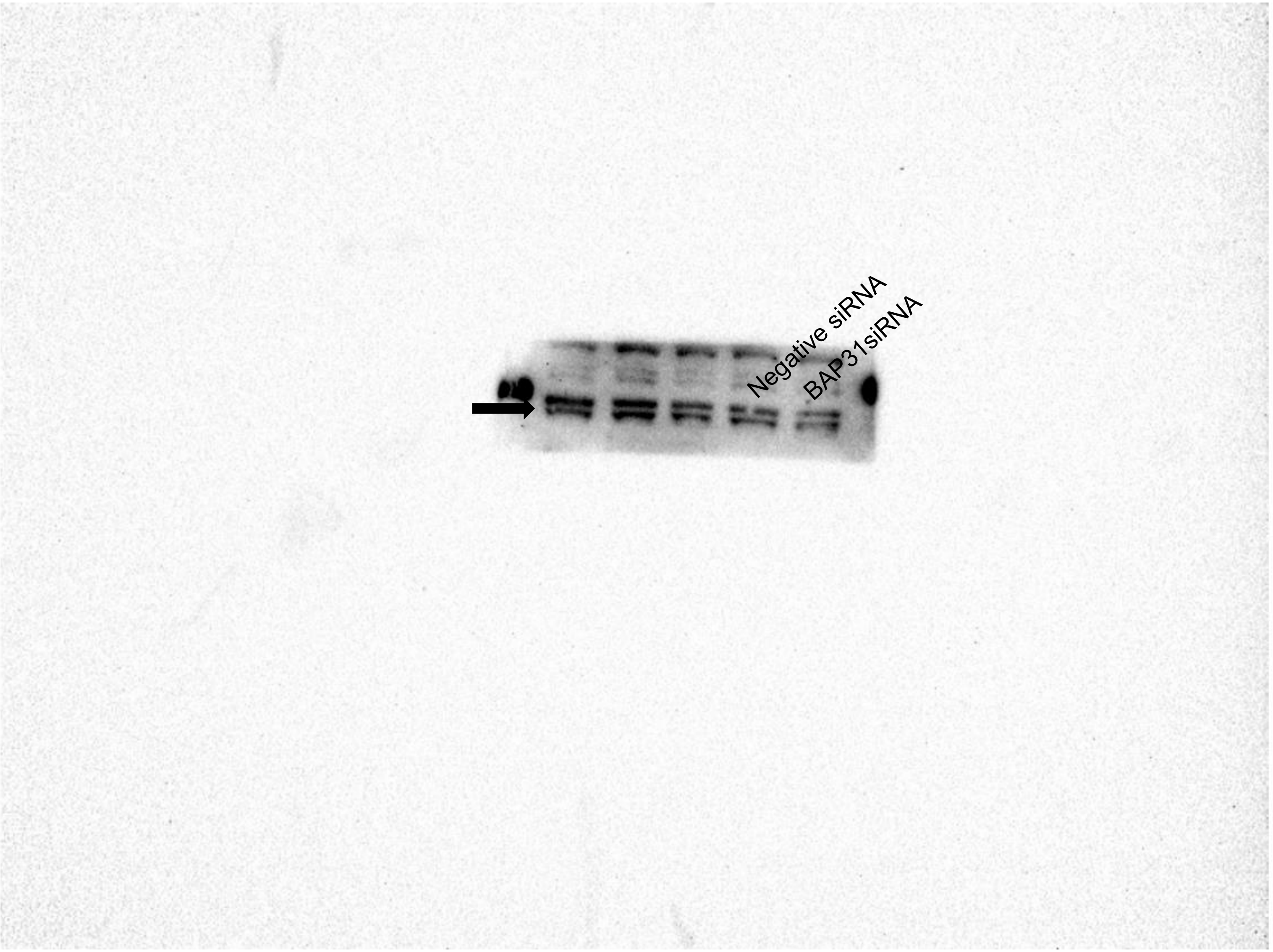

Fig4C zo-1

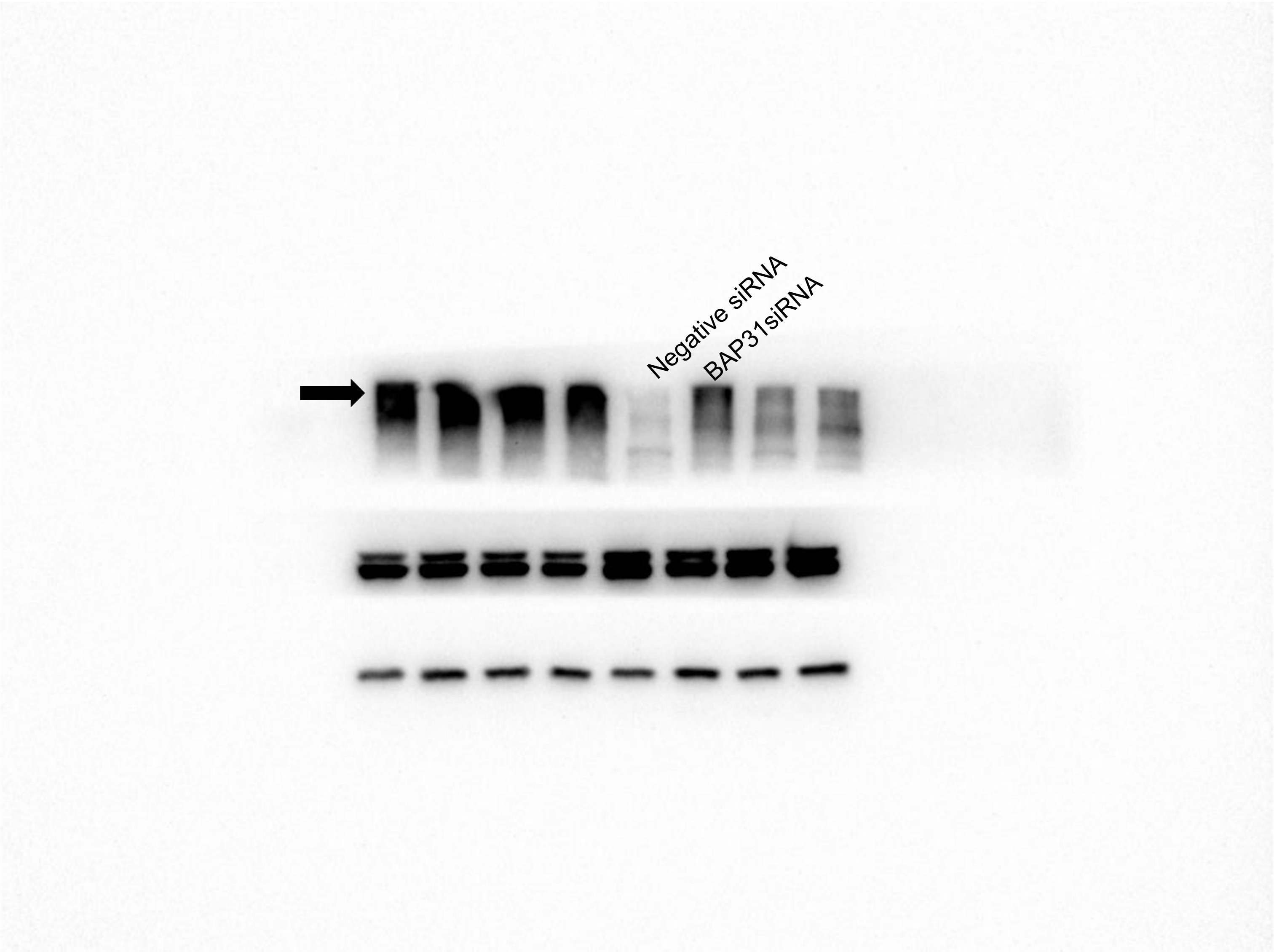

Fig4C claudin1

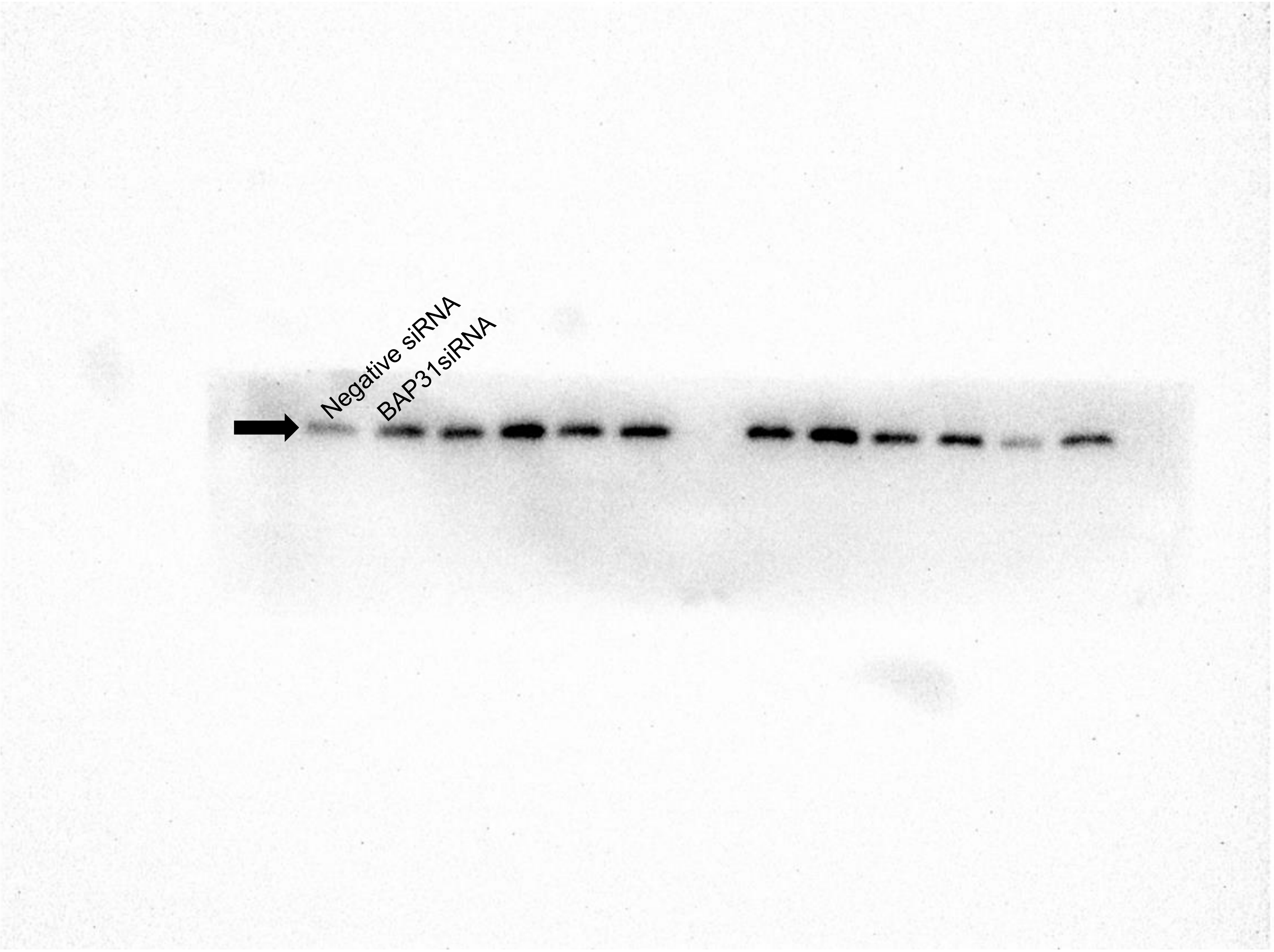

Fig4C bap31

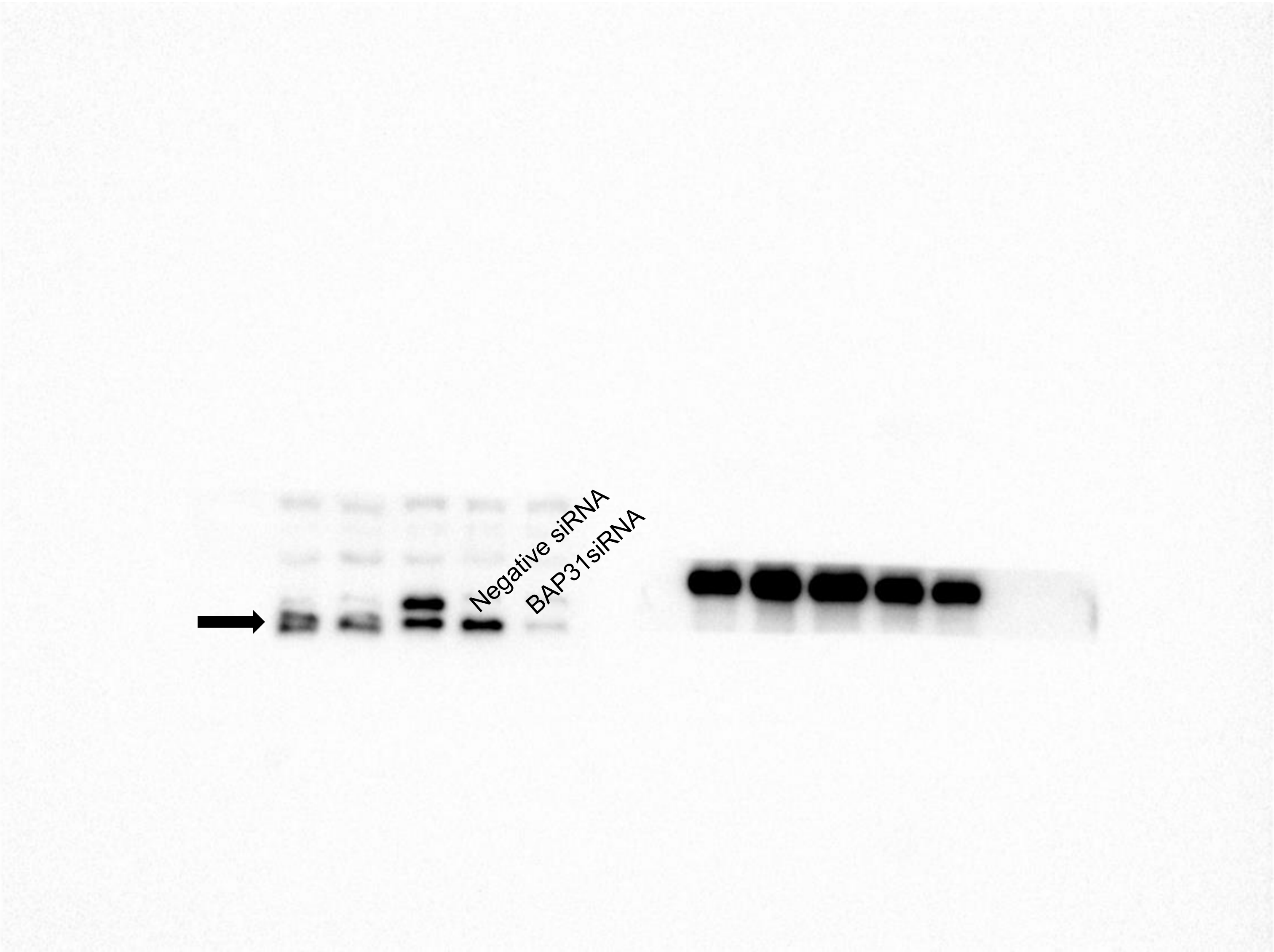

Fig4C gapdh

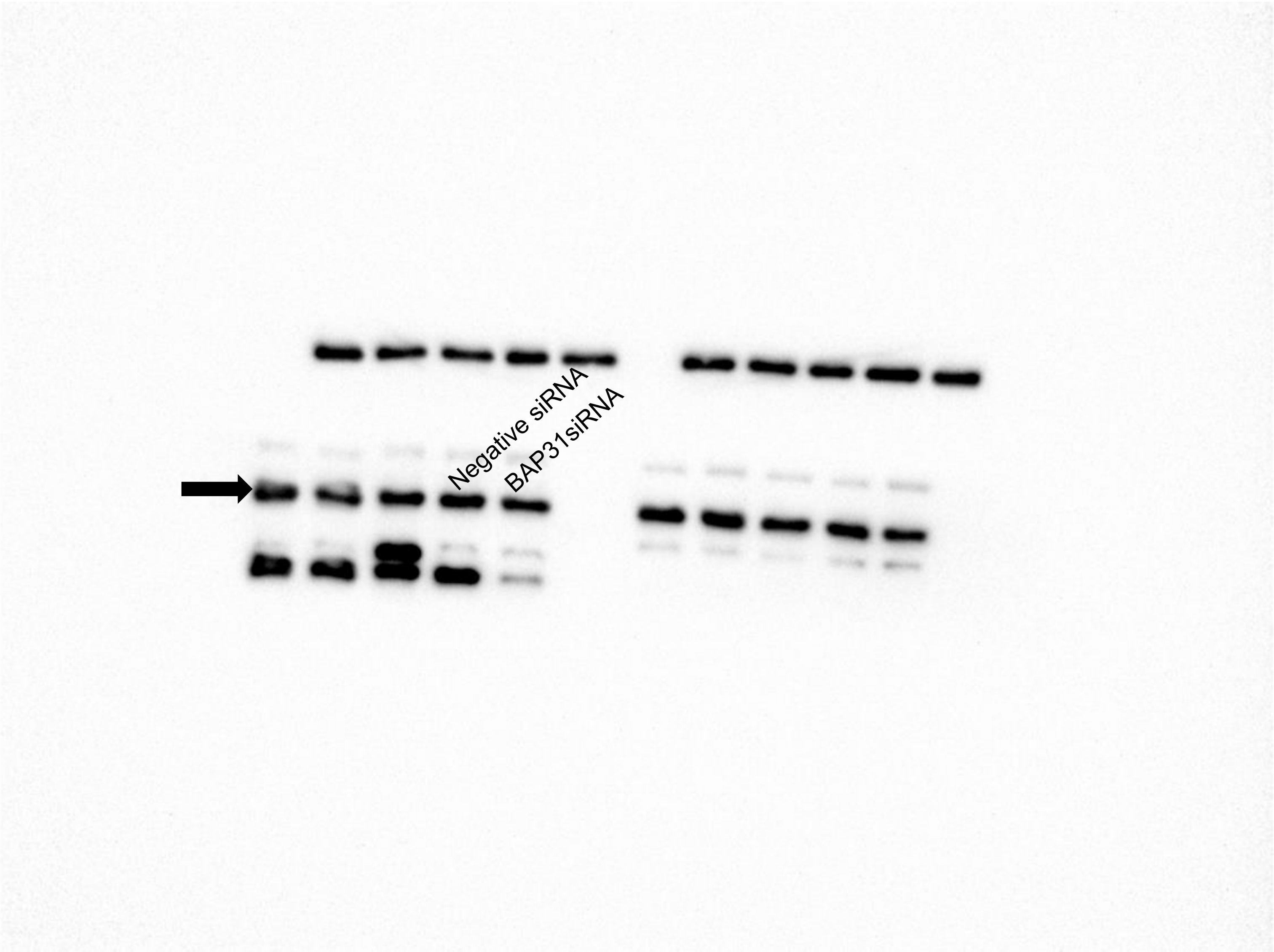

Fig4D

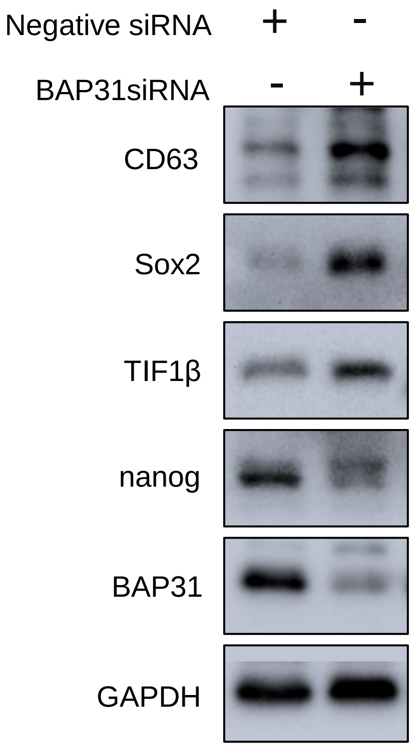

Negative siRNA + -
BAP31siRNA - +
CD63
Sox2
TIF1β
nanog
BAP31
GAPDH

Fig4D CD63

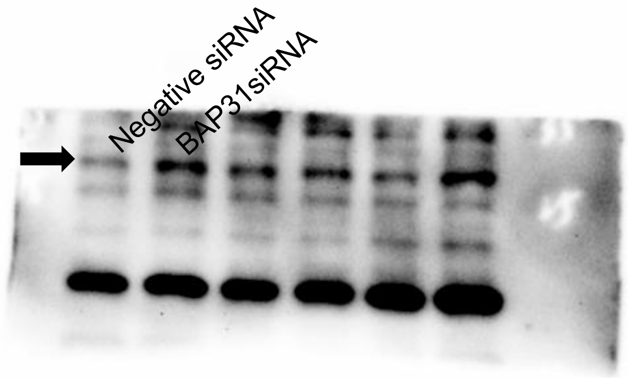

Fig4D sox2

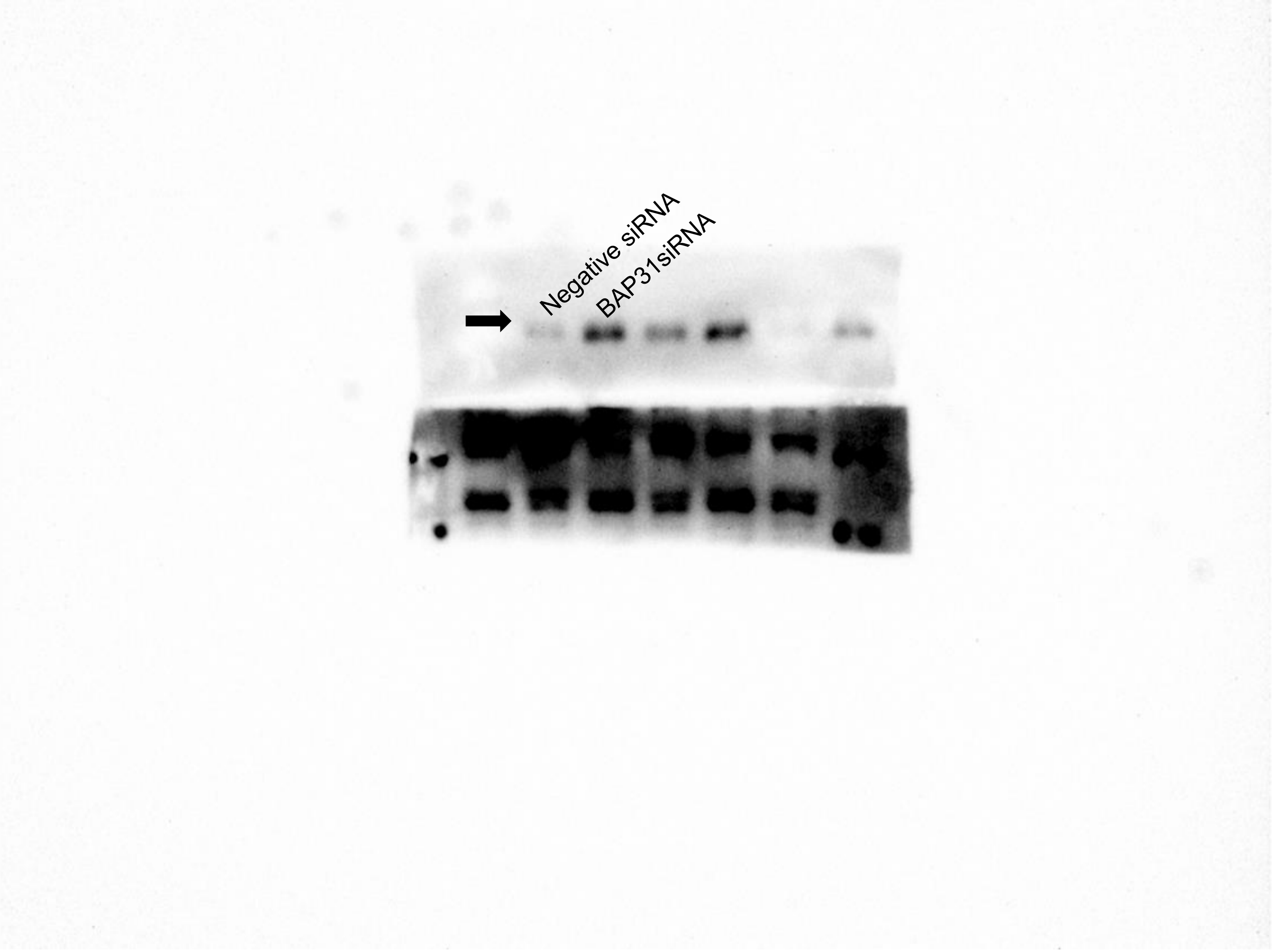

Fig4D tifbeta

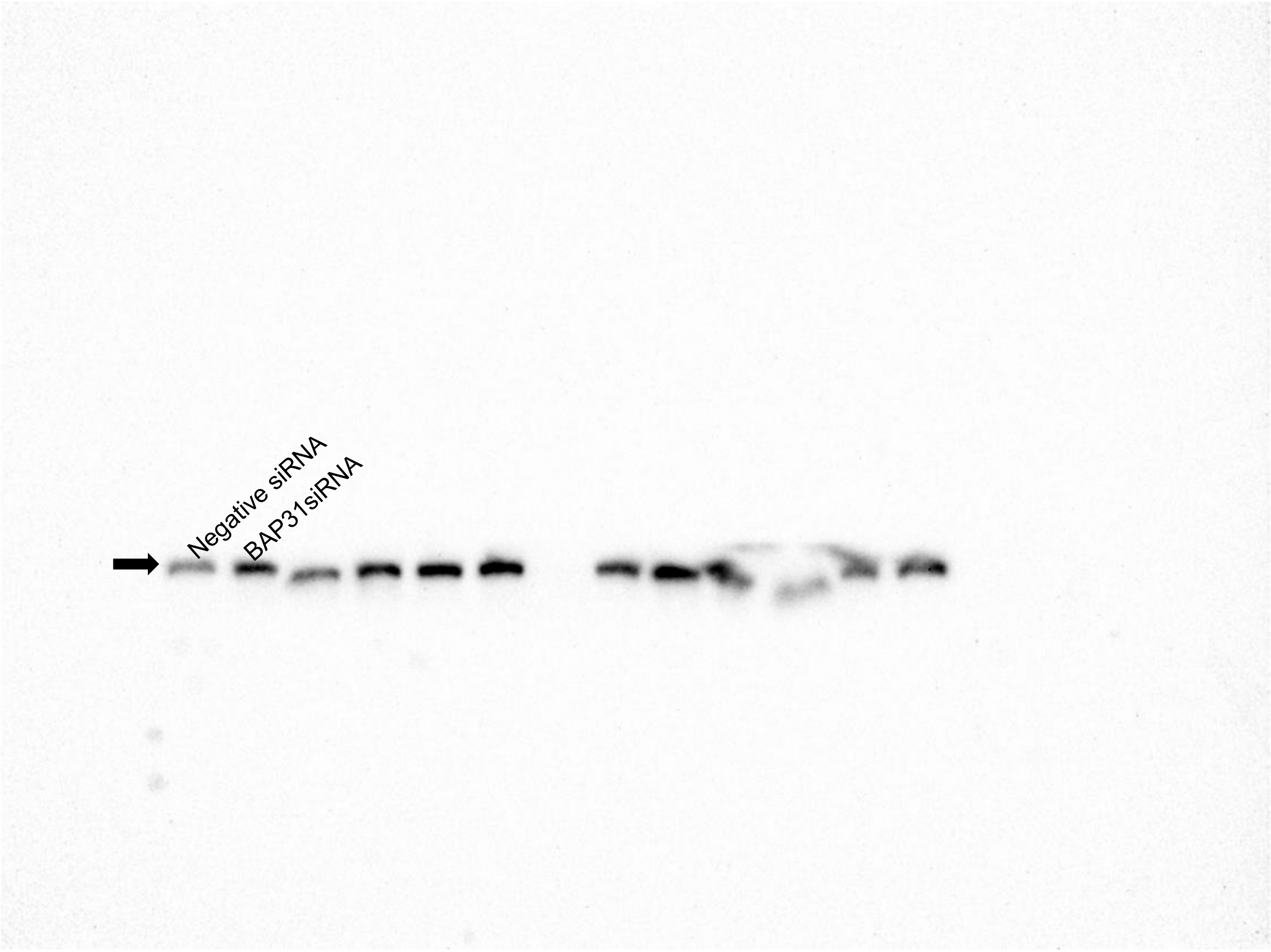

Fig4D nanog

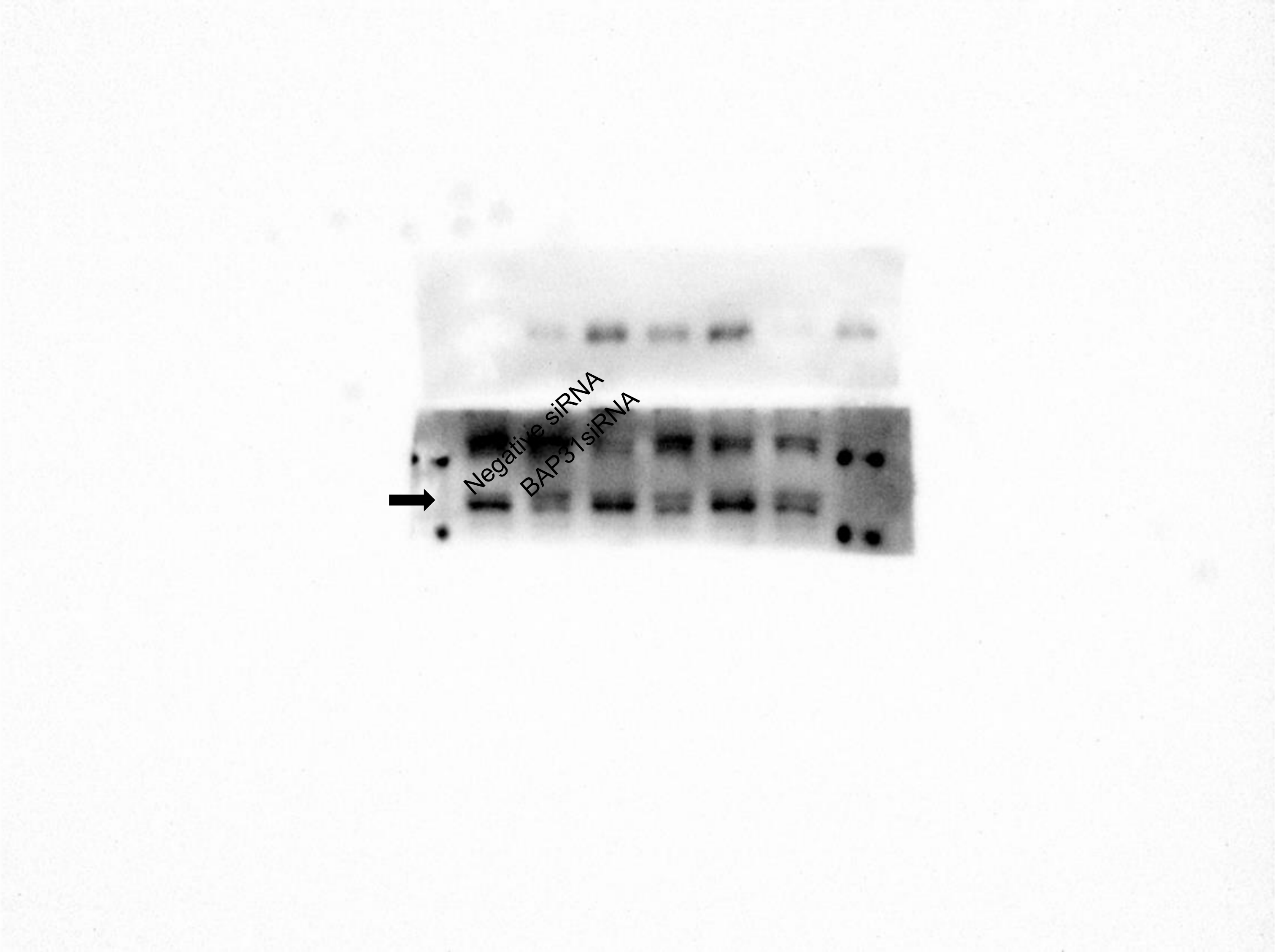

Fig4D bap31

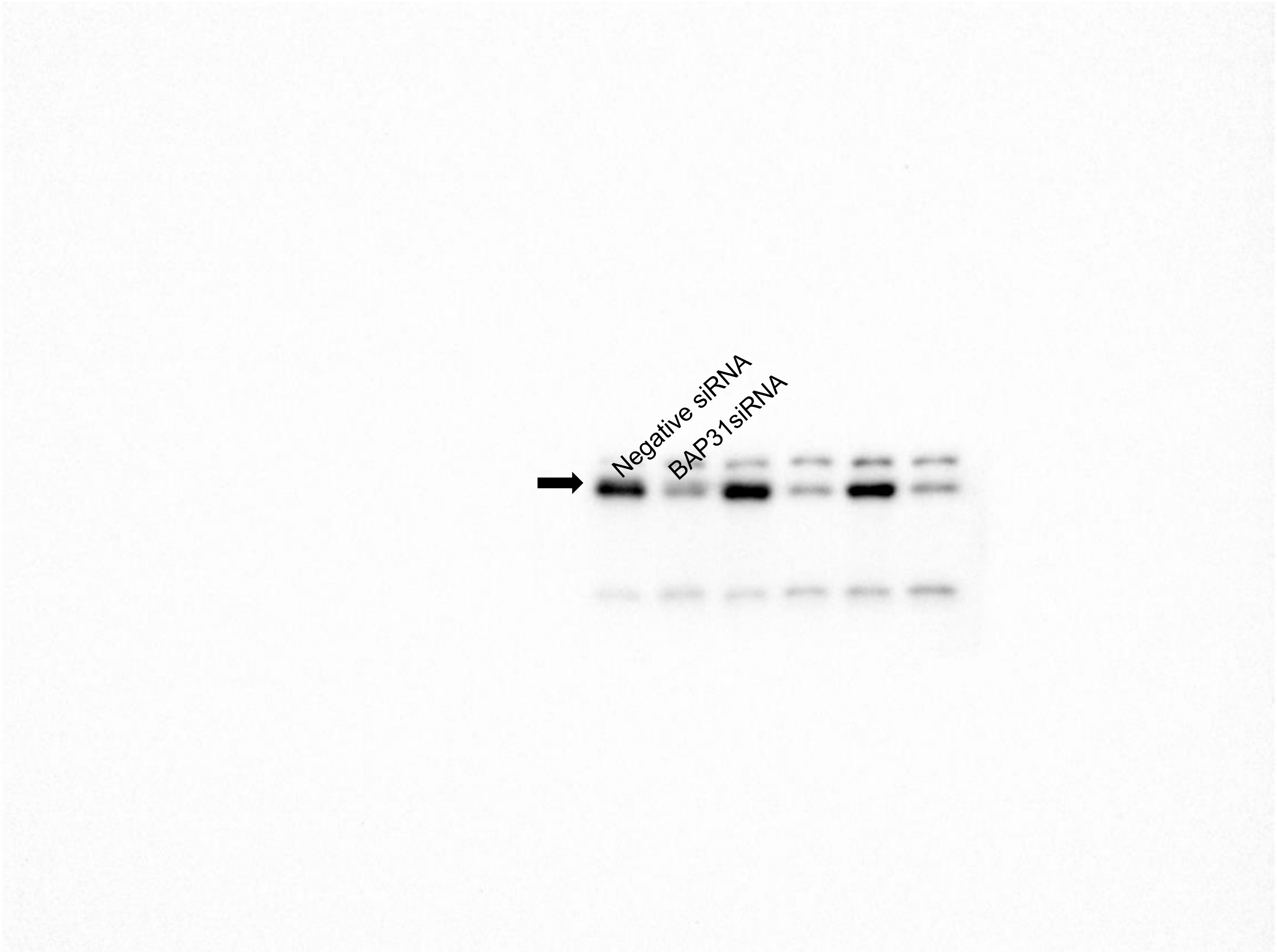

Fig4D gapdh

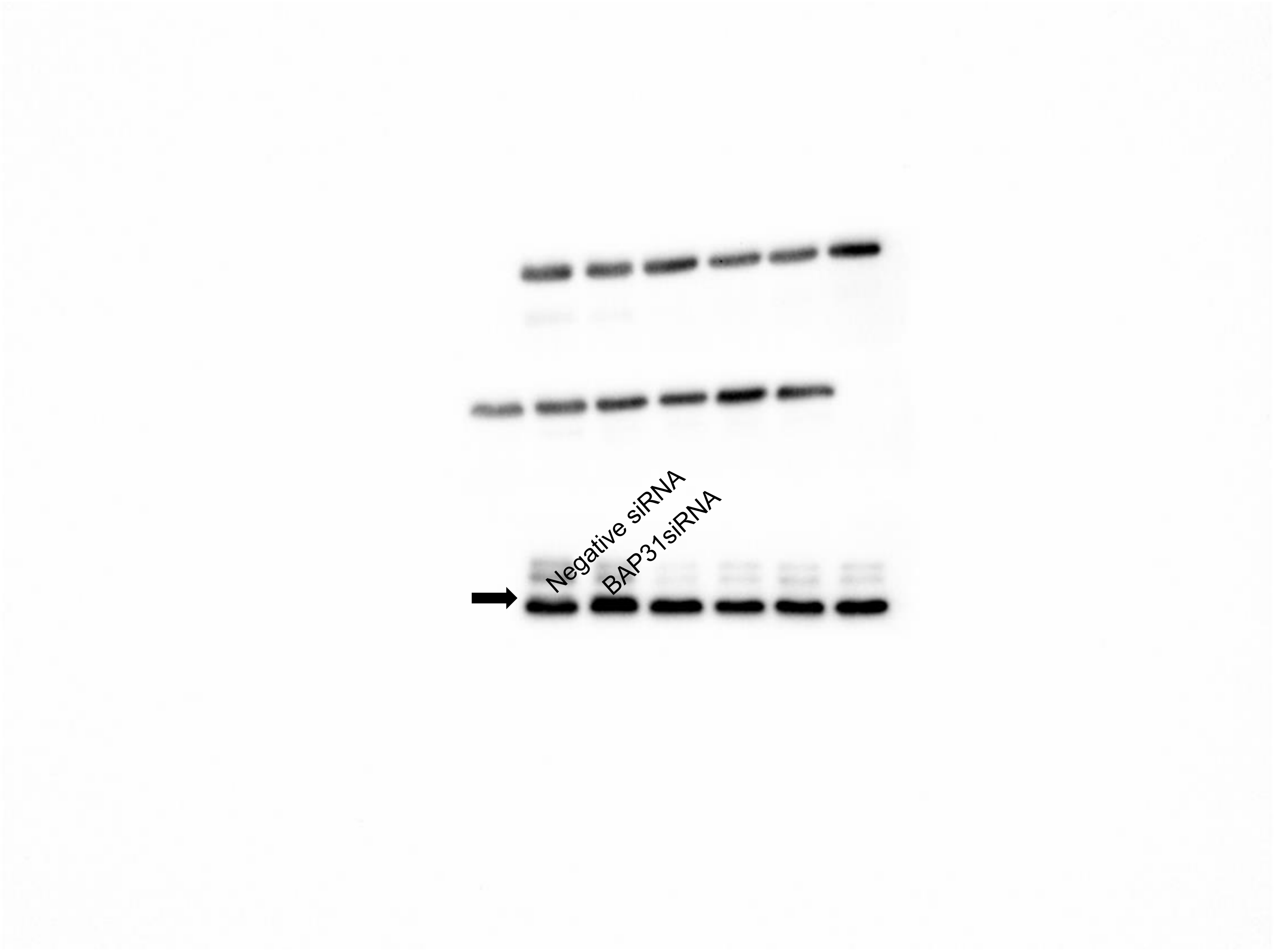

Supplement: Supplementary file 1 [file DataSheet_1.zip › raw data/Fig 4/Fig 4 western blot.pdf]
