# Supplementary material for: BAP31 Regulates Wnt Signaling to Modulate Cell Migration in Lung Cancer
Source: Front Oncol. 2022 Mar 10;12:859195. doi: 10.3389/fonc.2022.859195 (PMC8960194; doi:10.3389/fonc.2022.859195)
Supplement: Supplementary file 1 [file DataSheet_1.zip › raw data/Fig 5/Fig 5 western blot.pdf]

Fig5C

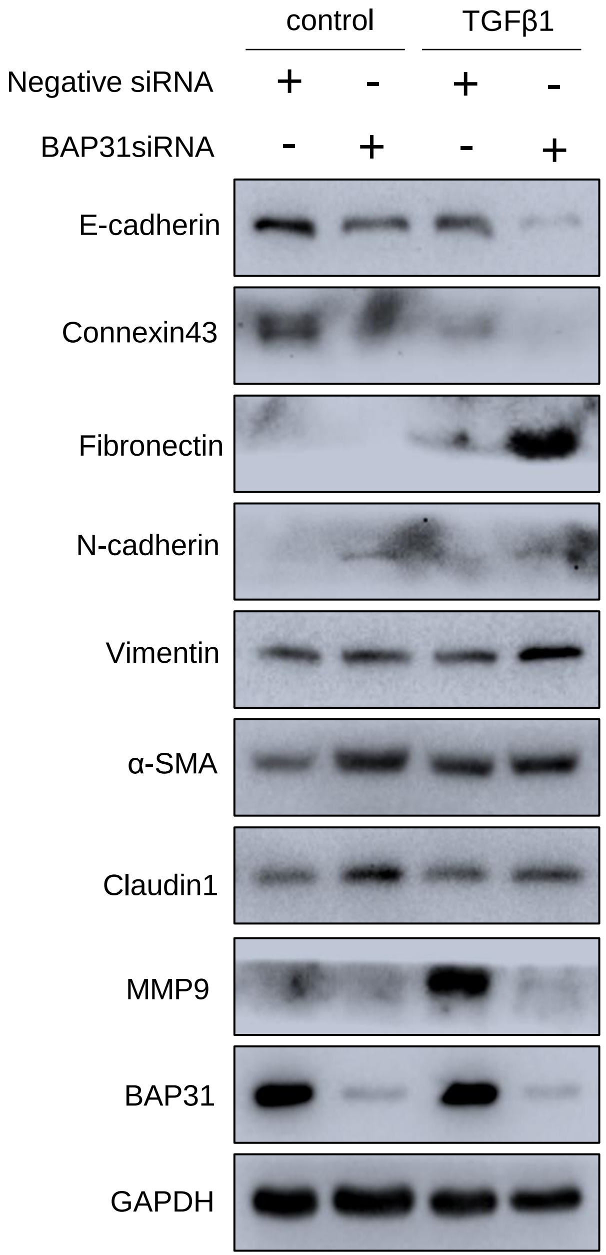
control
TGFβ1
Negative siRNA + - + -
BAP31siRNA - + - +
E-cadherin
Connexin43
Fibronectin
N-cadherin
Vimentin
α-SMA
Claudin1
MMP9
BAP31
GAPDH

Fig5C e-cadherin

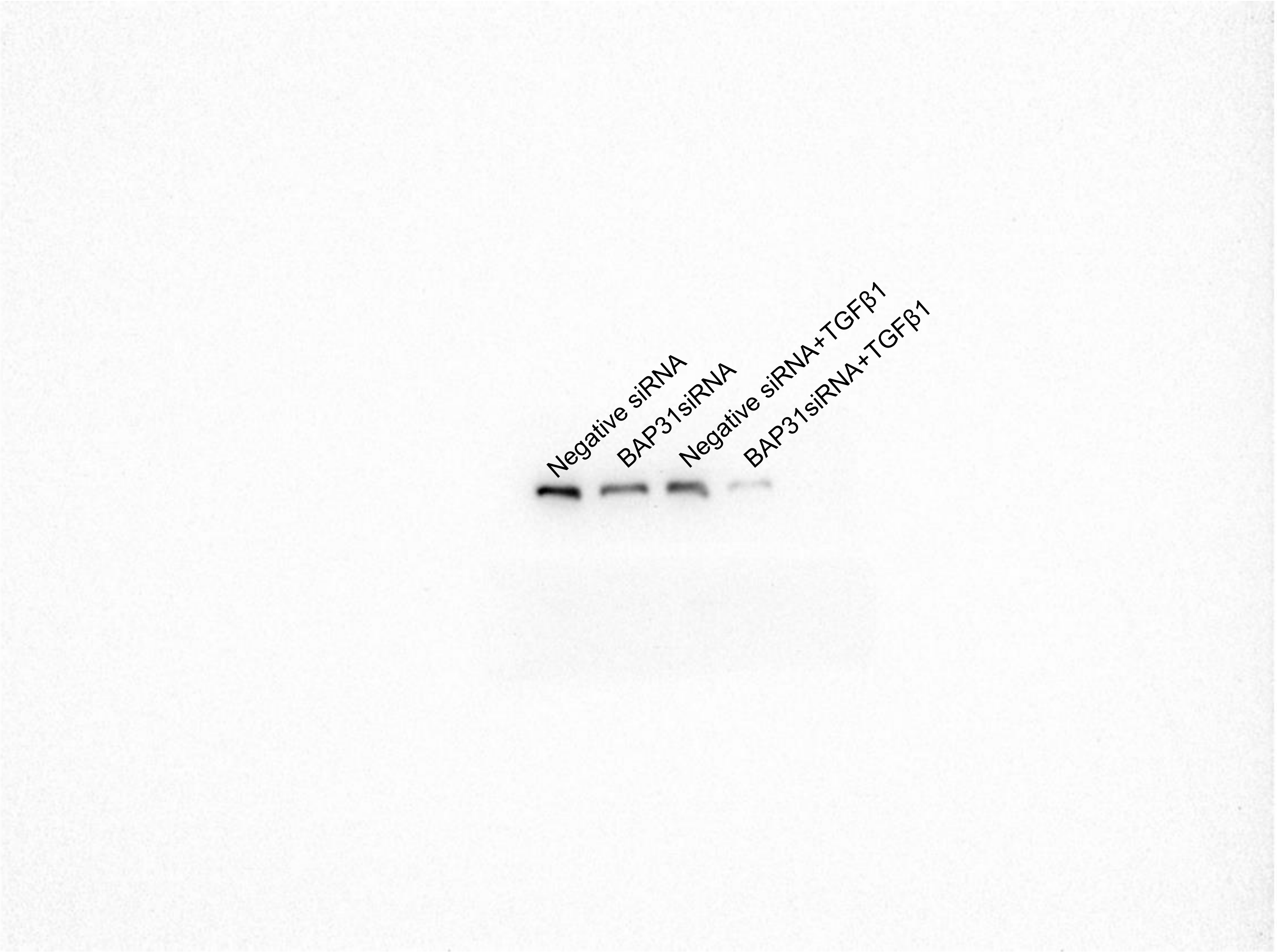

Fig5C connexin43

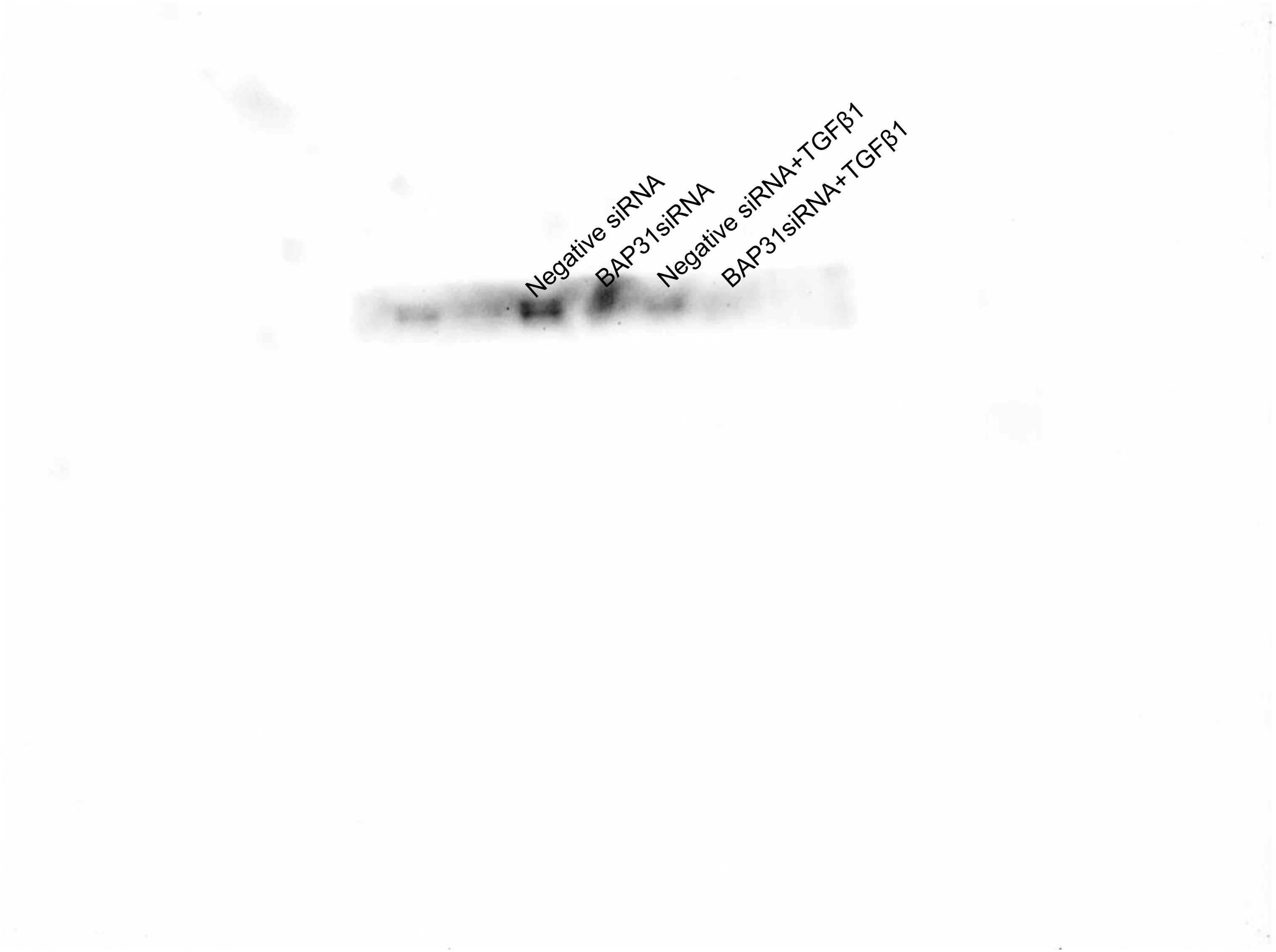

Fig5C fibronectin

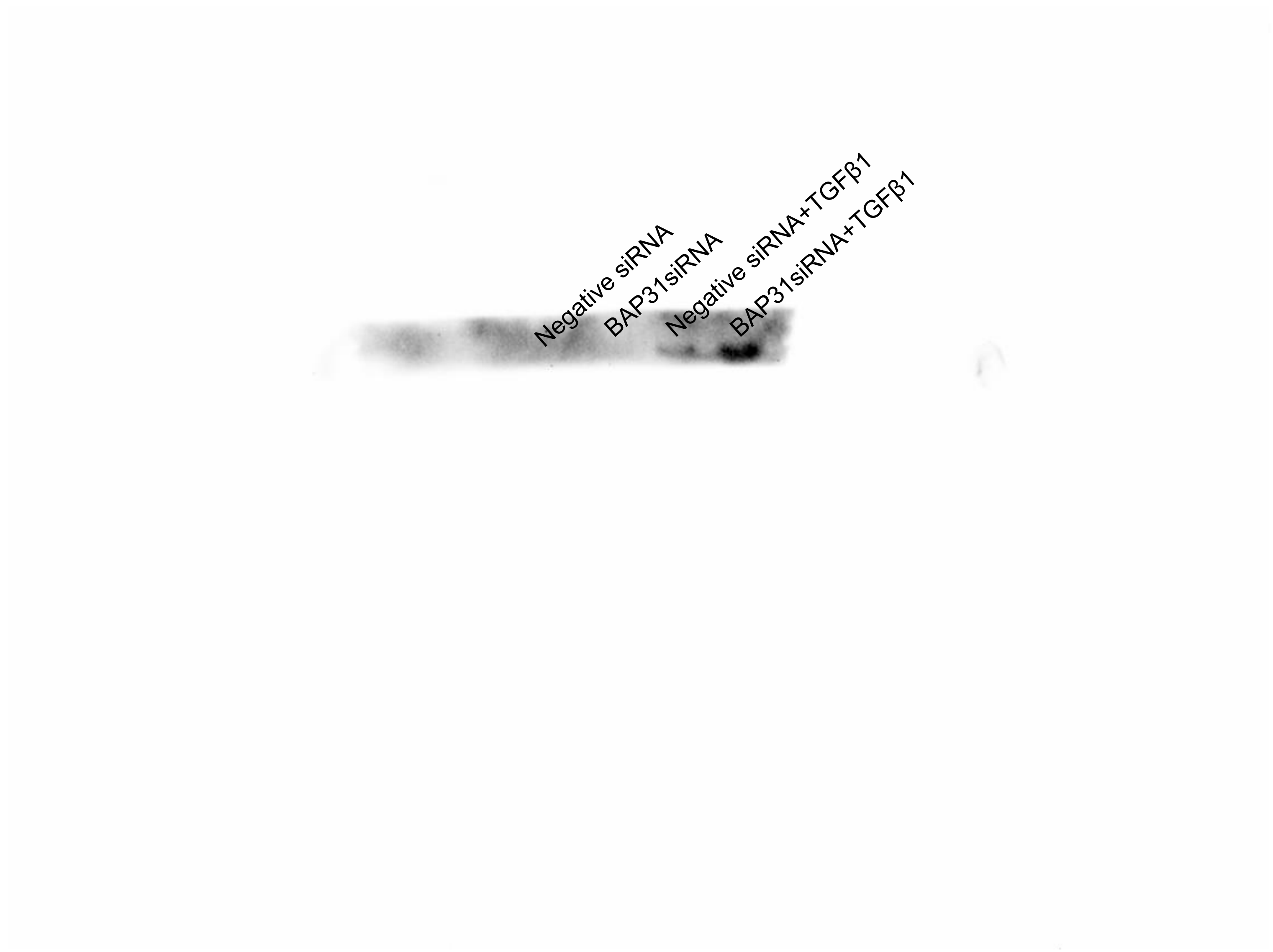

Fig5C n-cadherin

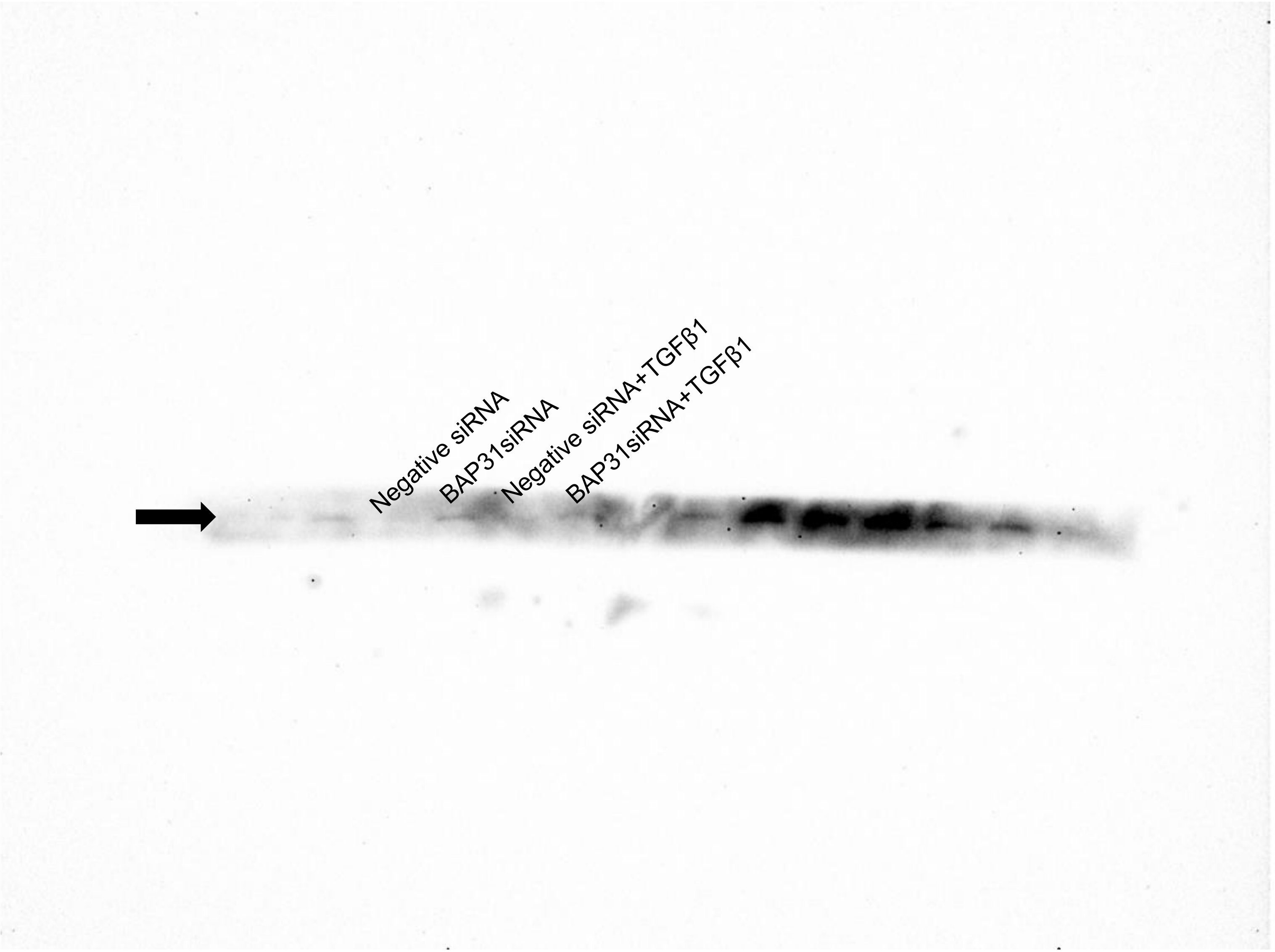

Fig5C vimentin

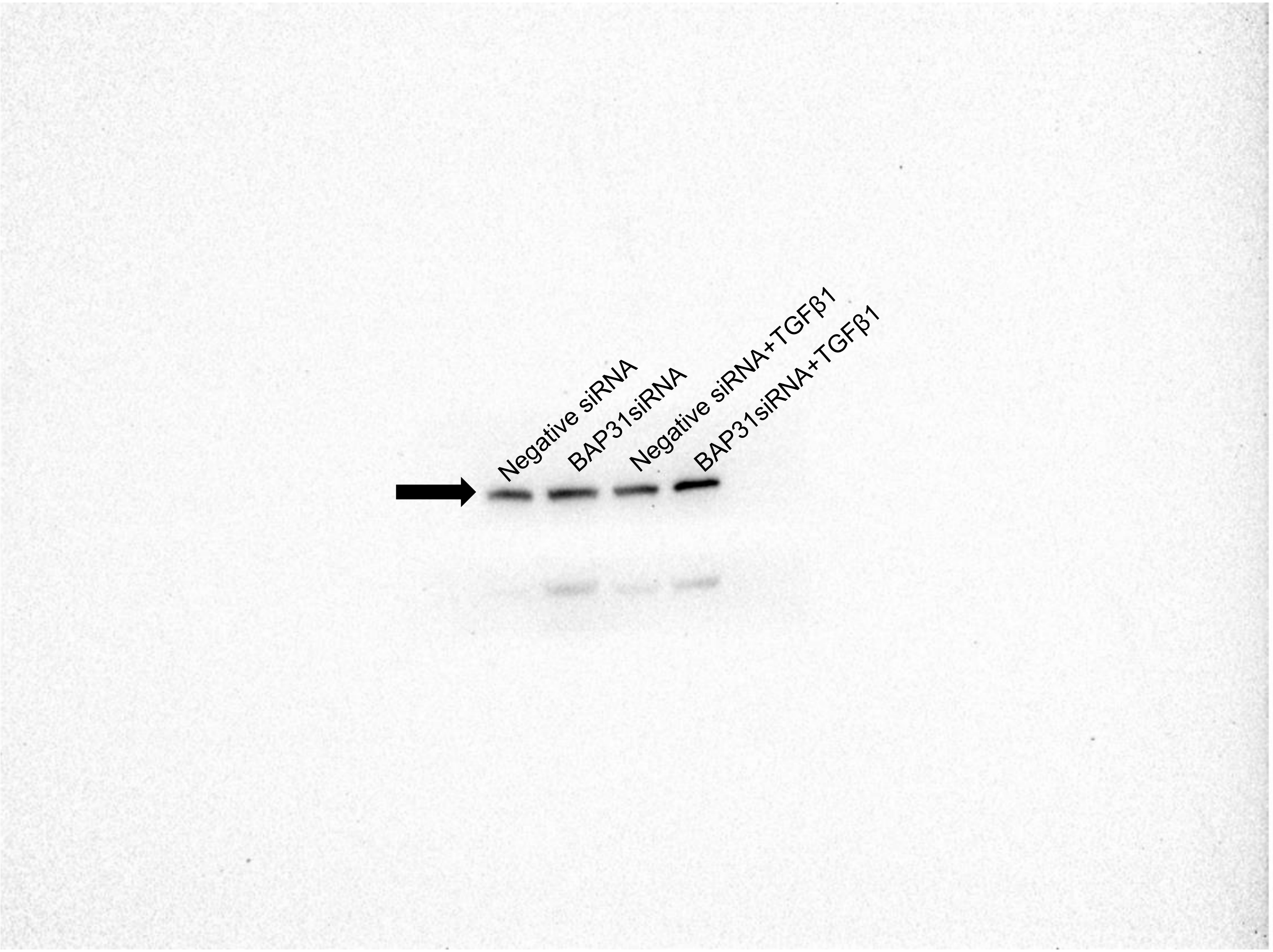

Fig5C α-SMA

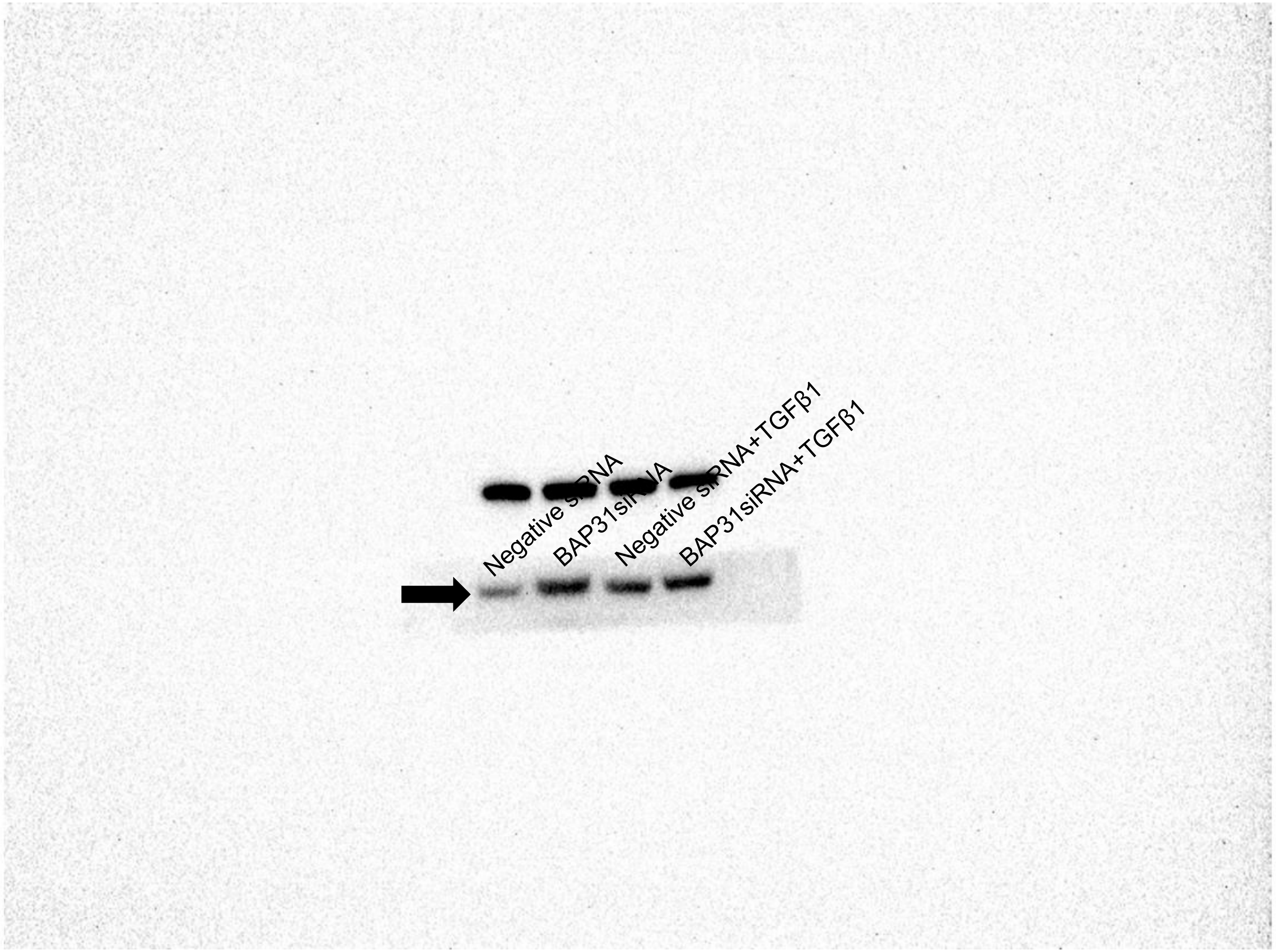

Negative siRNA
BAP31siRNA
Negative siRNA+TGFβ1
BAP31siRNA+TGFβ1

Fig5C claudin1

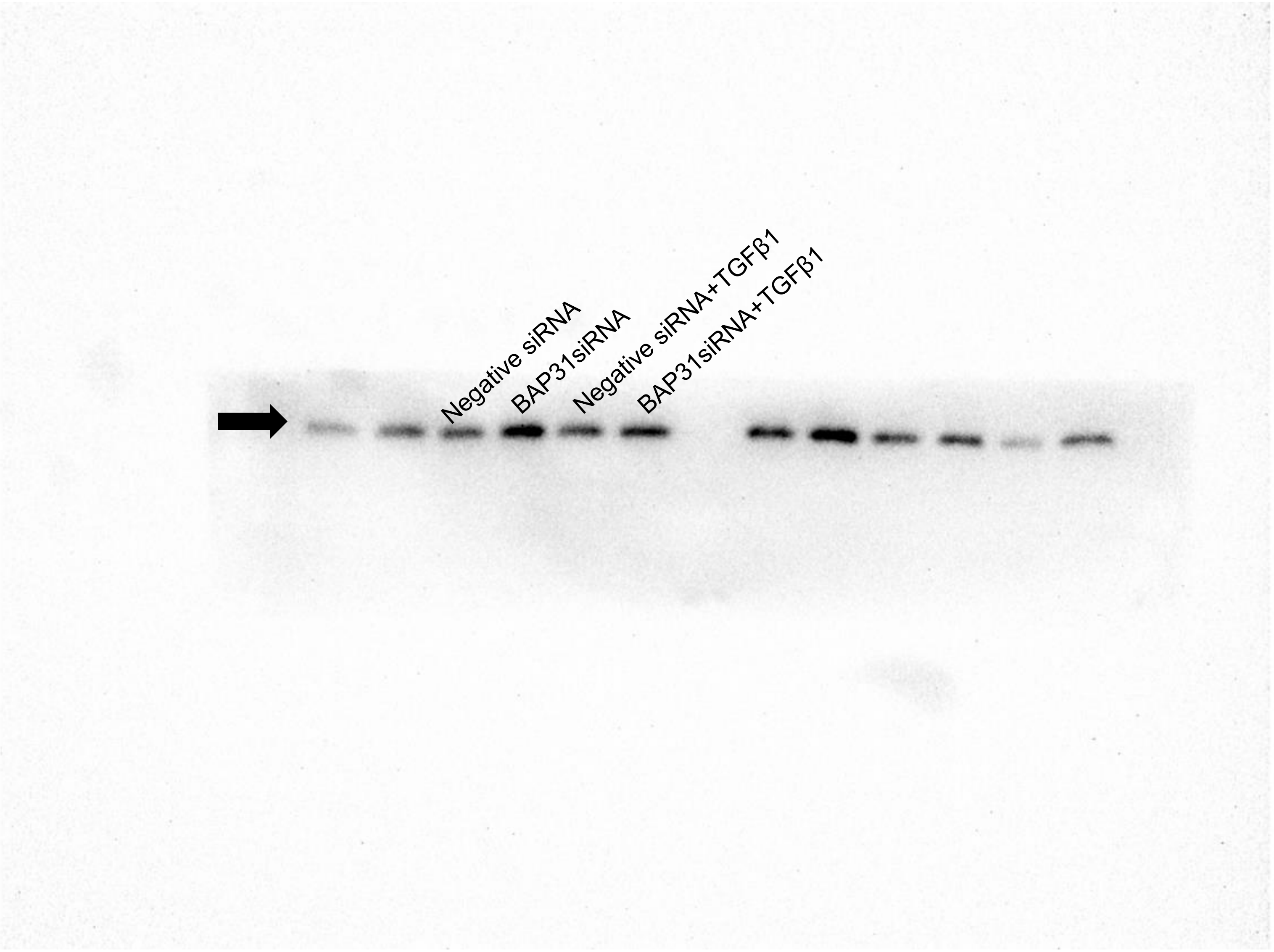

Fig5C mmp9

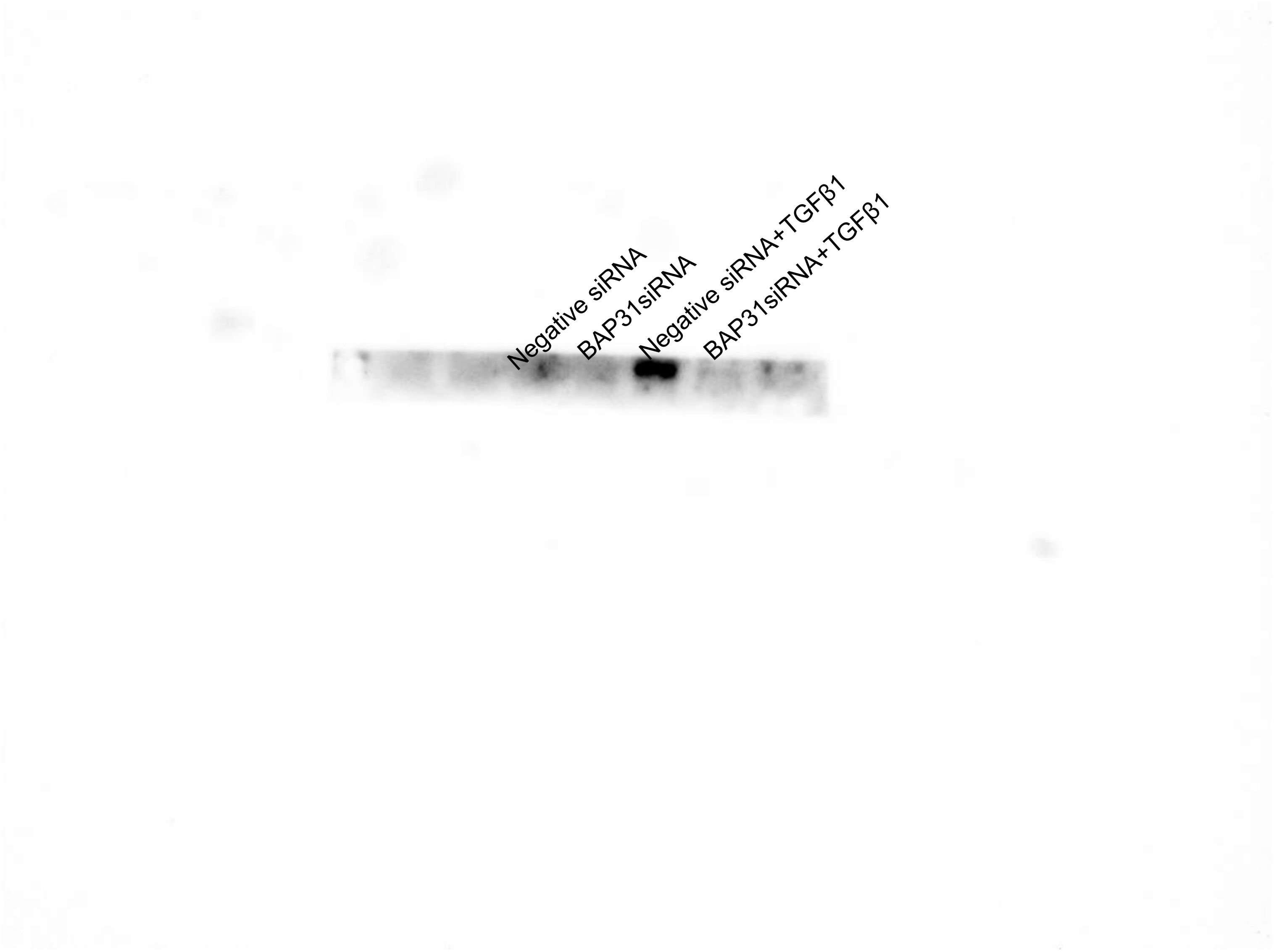
Negative siRNA
BAP31siRNA
Negative siRNA+TGFβ1
BAP31siRNA+TGFβ1

Fig5C BAP31

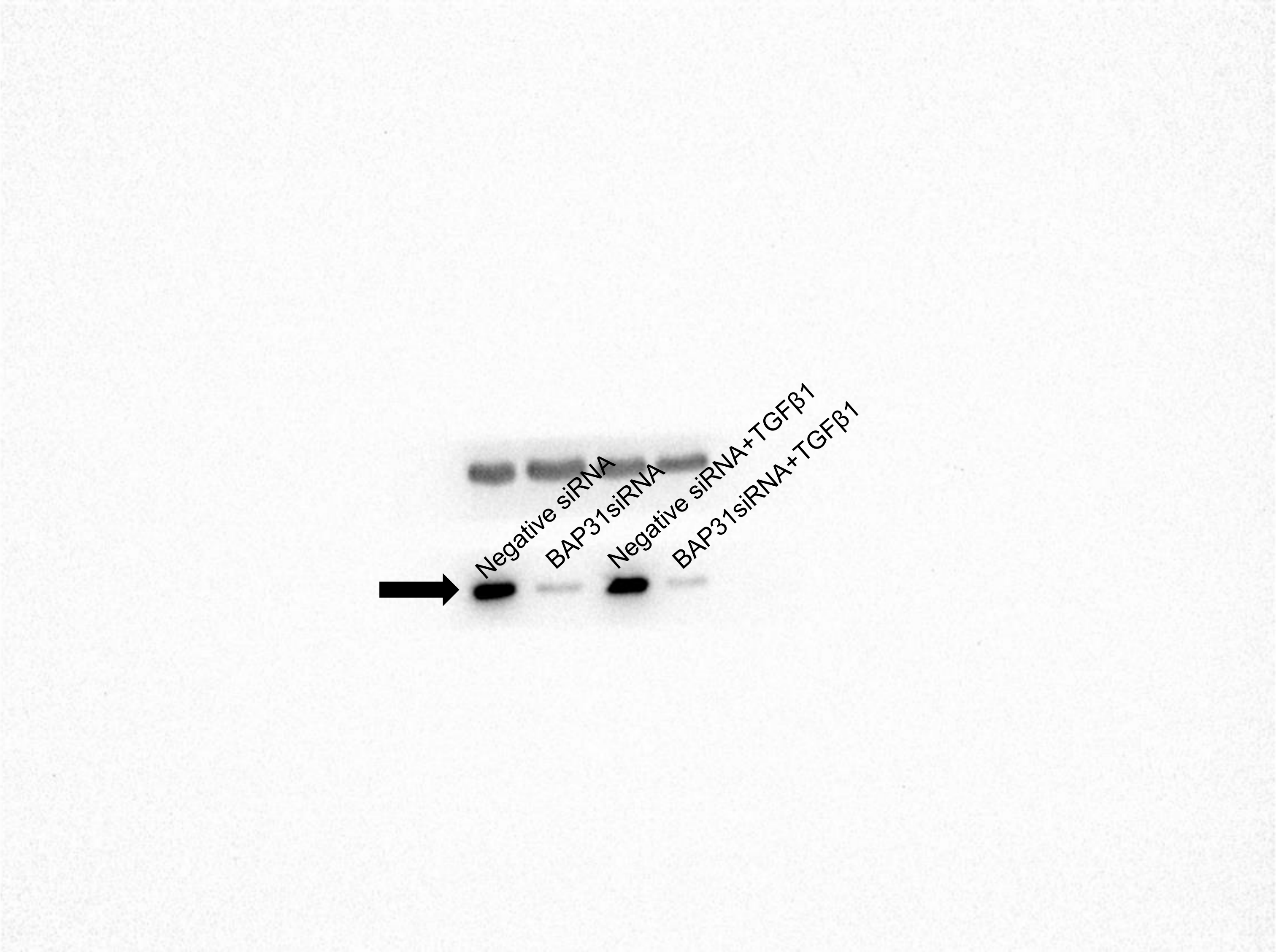
Negative siRNA
BAP31siRNA
Negative siRNA+TGFβ1
BAP31siRNA+TGFβ1

Fig5C gapdh

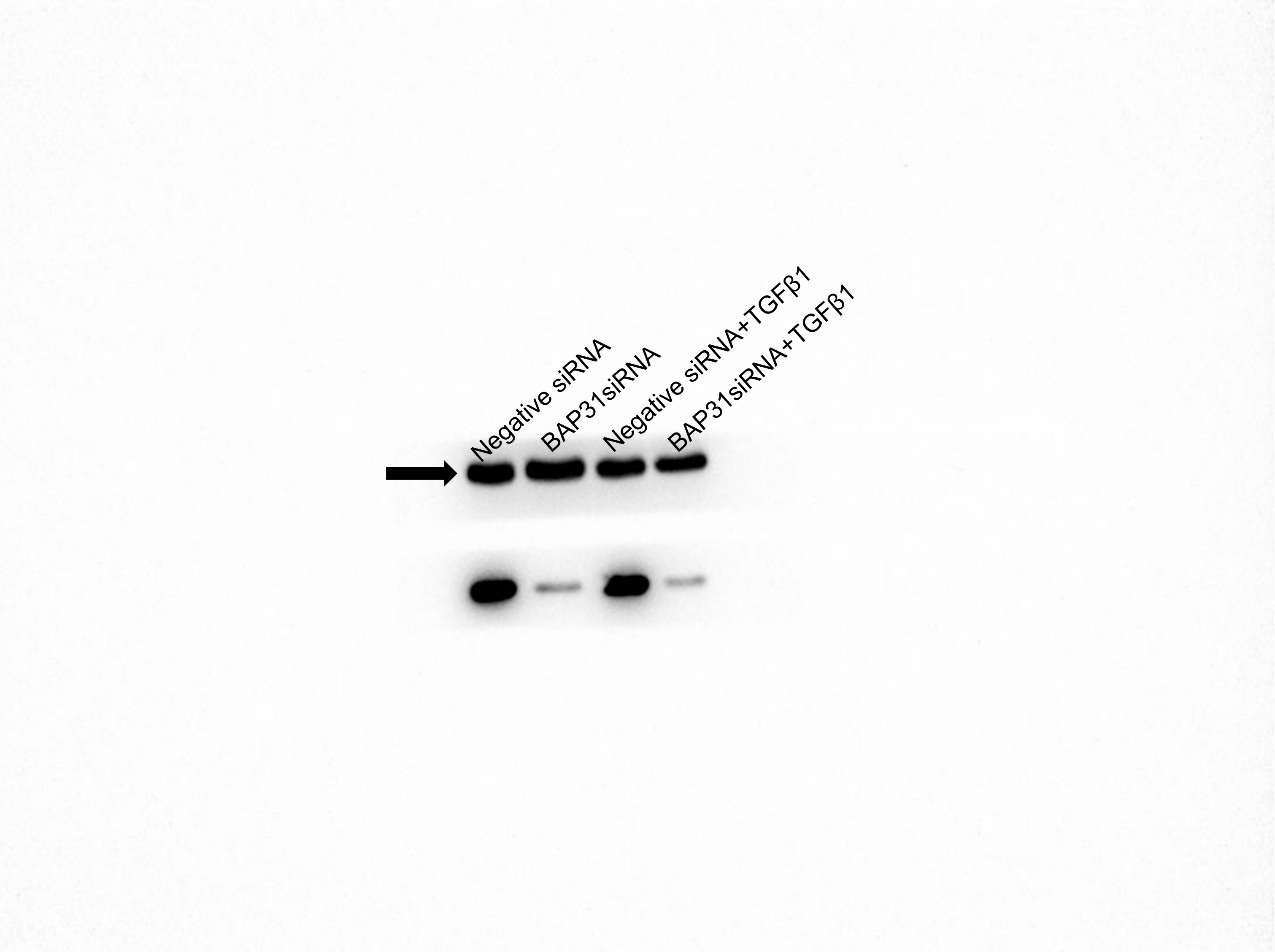
Negative siRNA
BAP31siRNA
Negative siRNA+TGFβ1
BAP31siRNA+TGFβ1
