# Supplementary material for: BAP31 Regulates Wnt Signaling to Modulate Cell Migration in Lung Cancer
Source: Front Oncol. 2022 Mar 10;12:859195. doi: 10.3389/fonc.2022.859195 (PMC8960194; doi:10.3389/fonc.2022.859195)
Supplement: Supplementary file 1 [file DataSheet_1.zip › raw data/Fig 7/Fig 7 western blot.pdf]

Fig6A

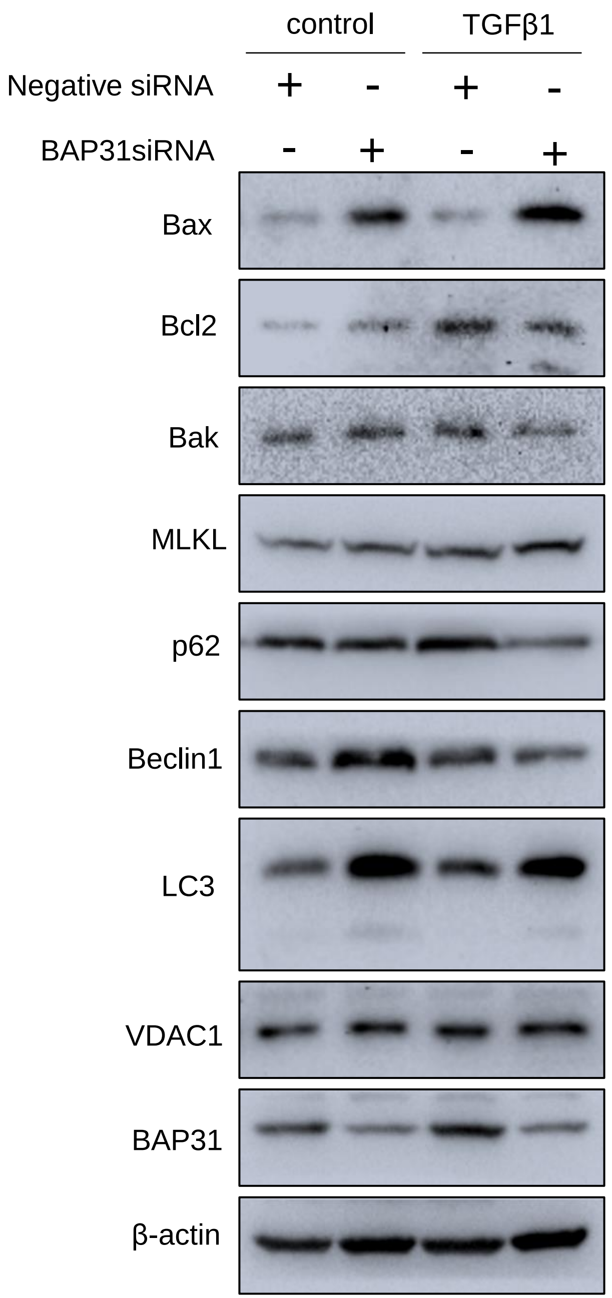
control
TGFβ1
Negative siRNA
+
-
+
-
BAP31siRNA
-
+
-
+
Bax
Bcl2
Bak
MLKL
p62
Beclin1
LC3
VDAC1
BAP31
β-actin

Fig6A Bax

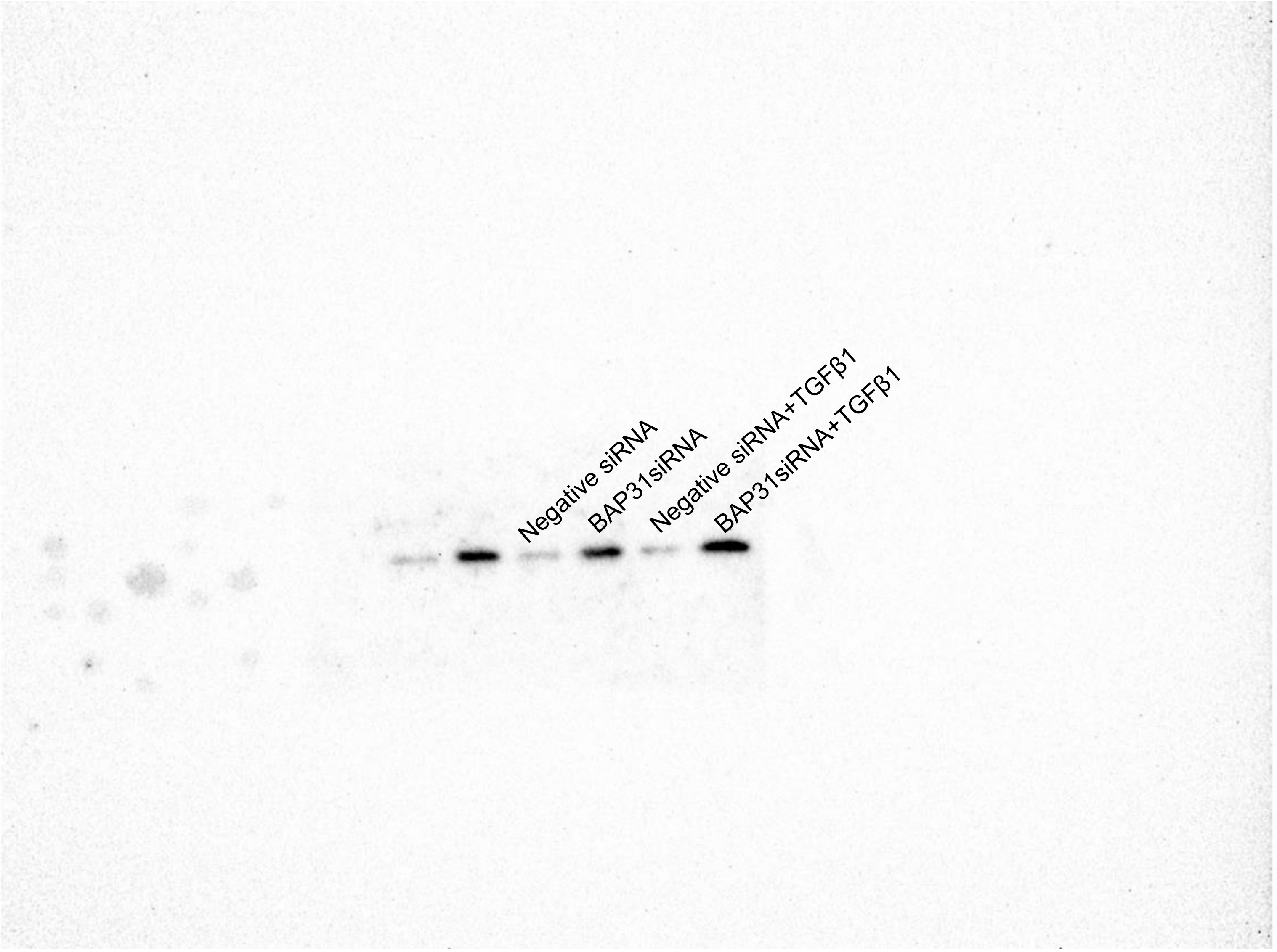
Negative siRNA
BAP31siRNA
Negative siRNA+TGFβ1
BAP31siRNA+TGFβ1

Fig6A Bcl2

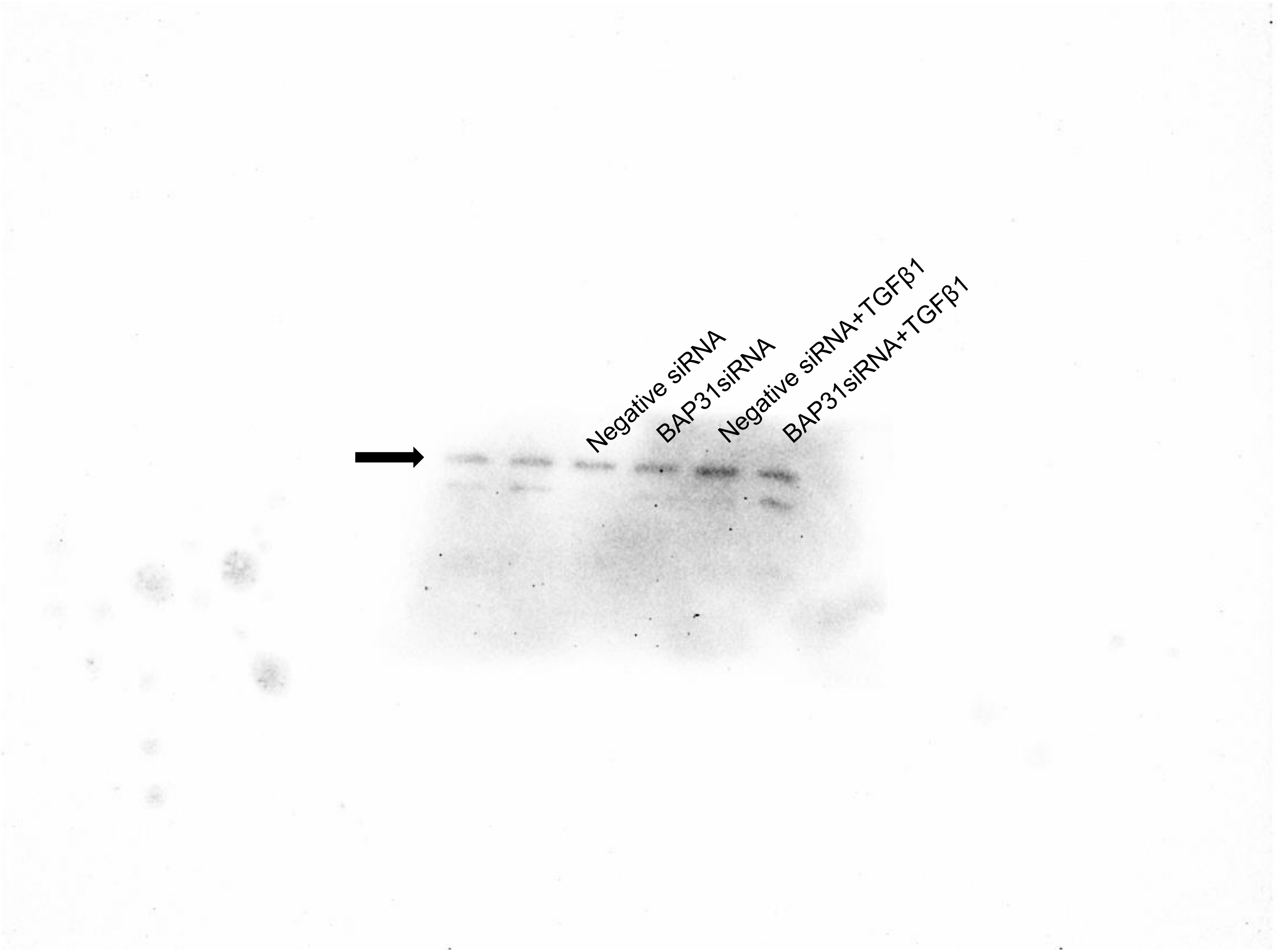

Negative siRNA
BAP31siRNA
Negative siRNA+TGFβ1
BAP31siRNA+TGFβ1

Fig6A Bak

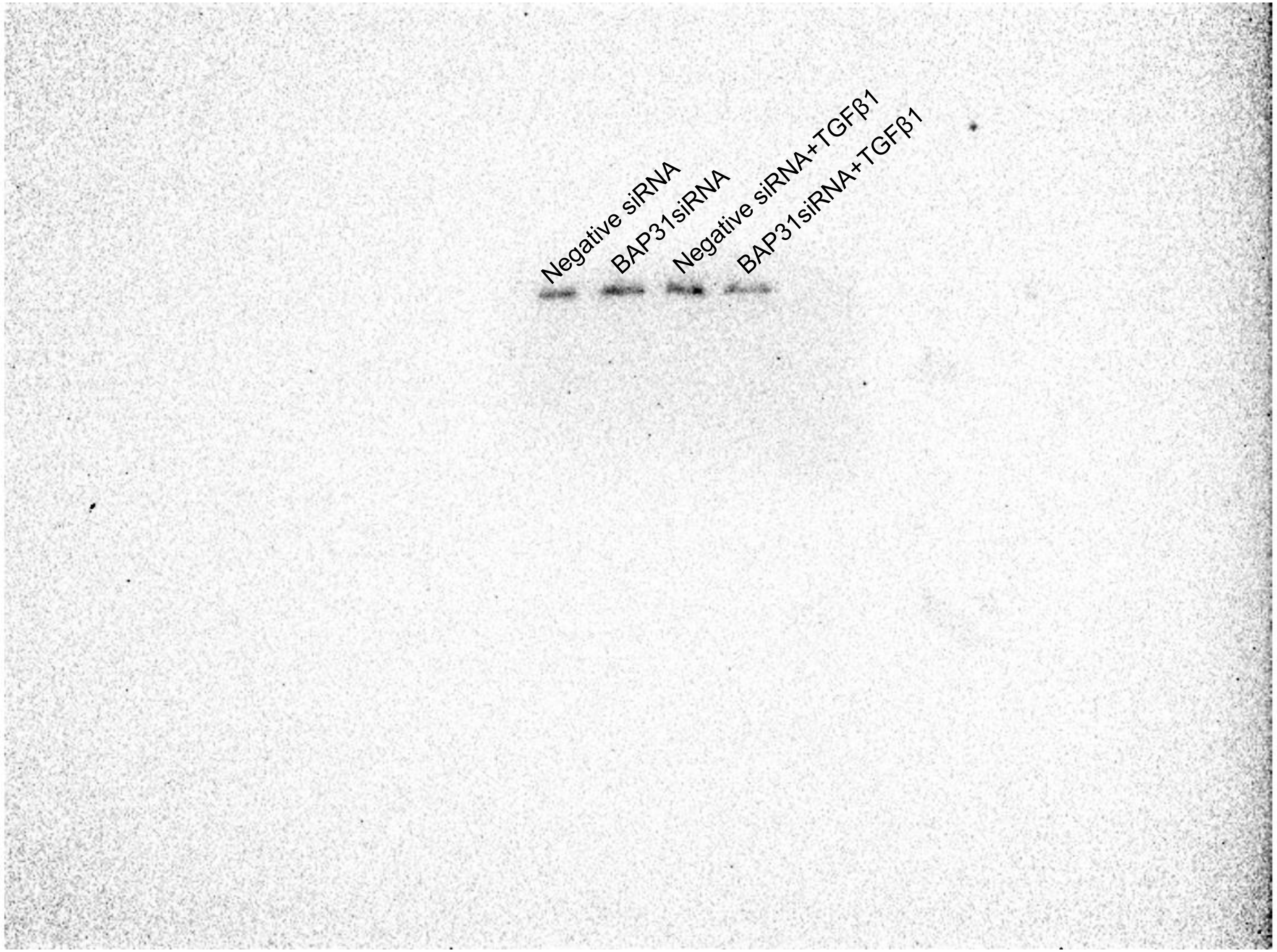
Negative siRNA
BAP31siRNA
Negative siRNA+TGFβ1
BAP31siRNA+TGFβ1

Fig6A mlkl

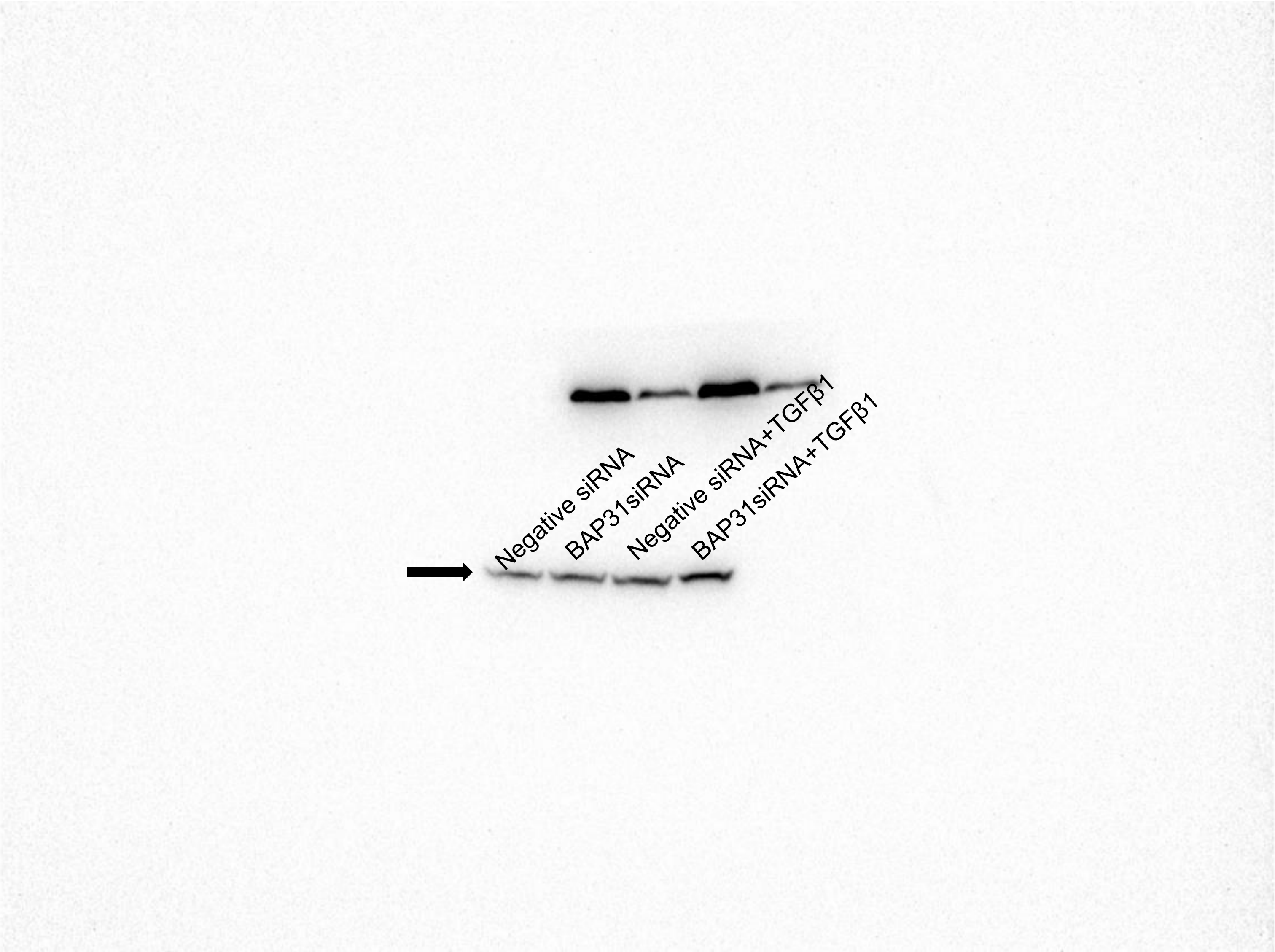
Negative siRNA
BAP31siRNA
Negative siRNA+TGFβ1
BAP31siRNA+TGFβ1

Fig6A p62

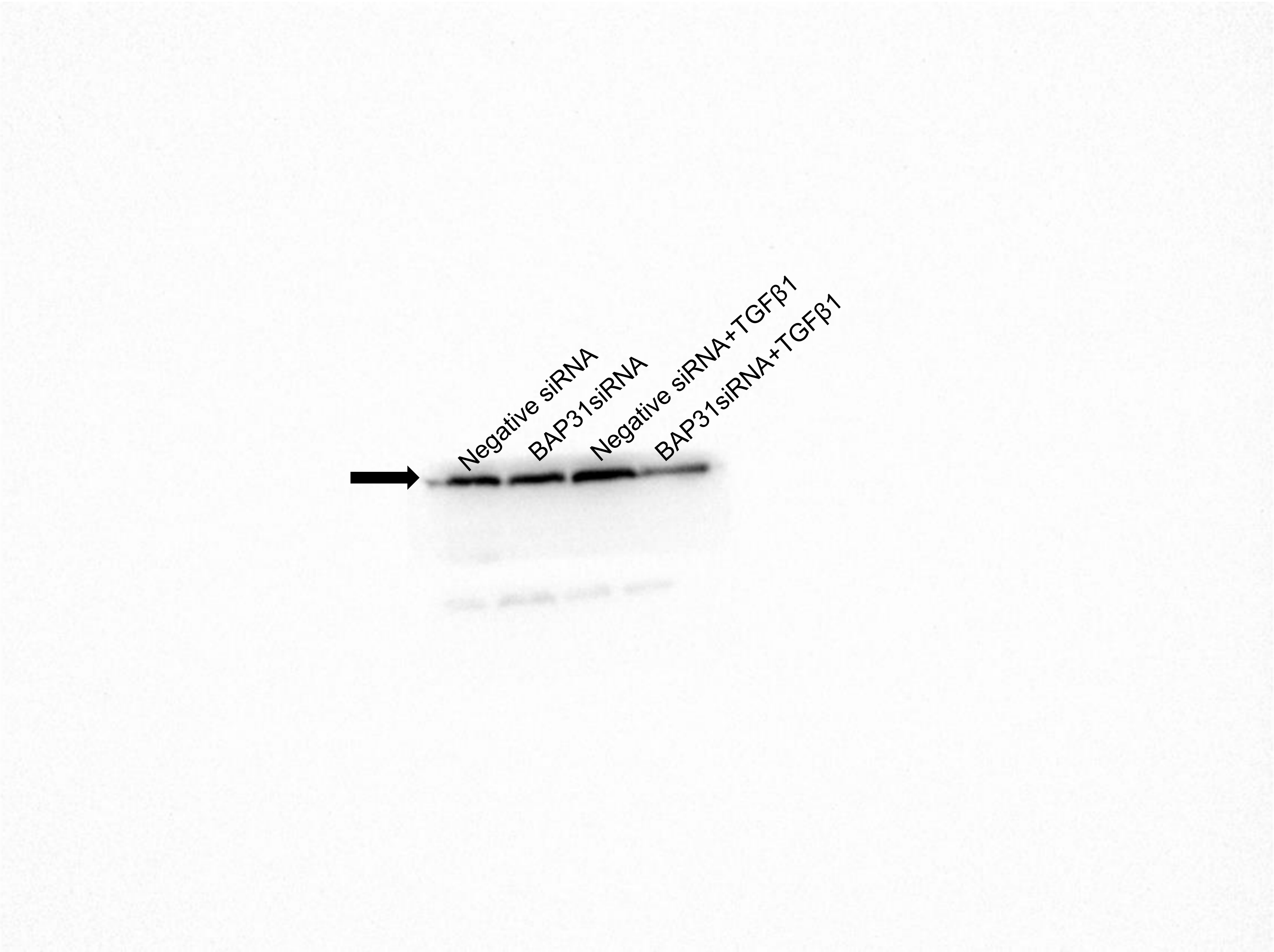
Negative siRNA
BAP31siRNA
Negative siRNA+TGFβ1
BAP31siRNA+TGFβ1

Fig6A beclin1

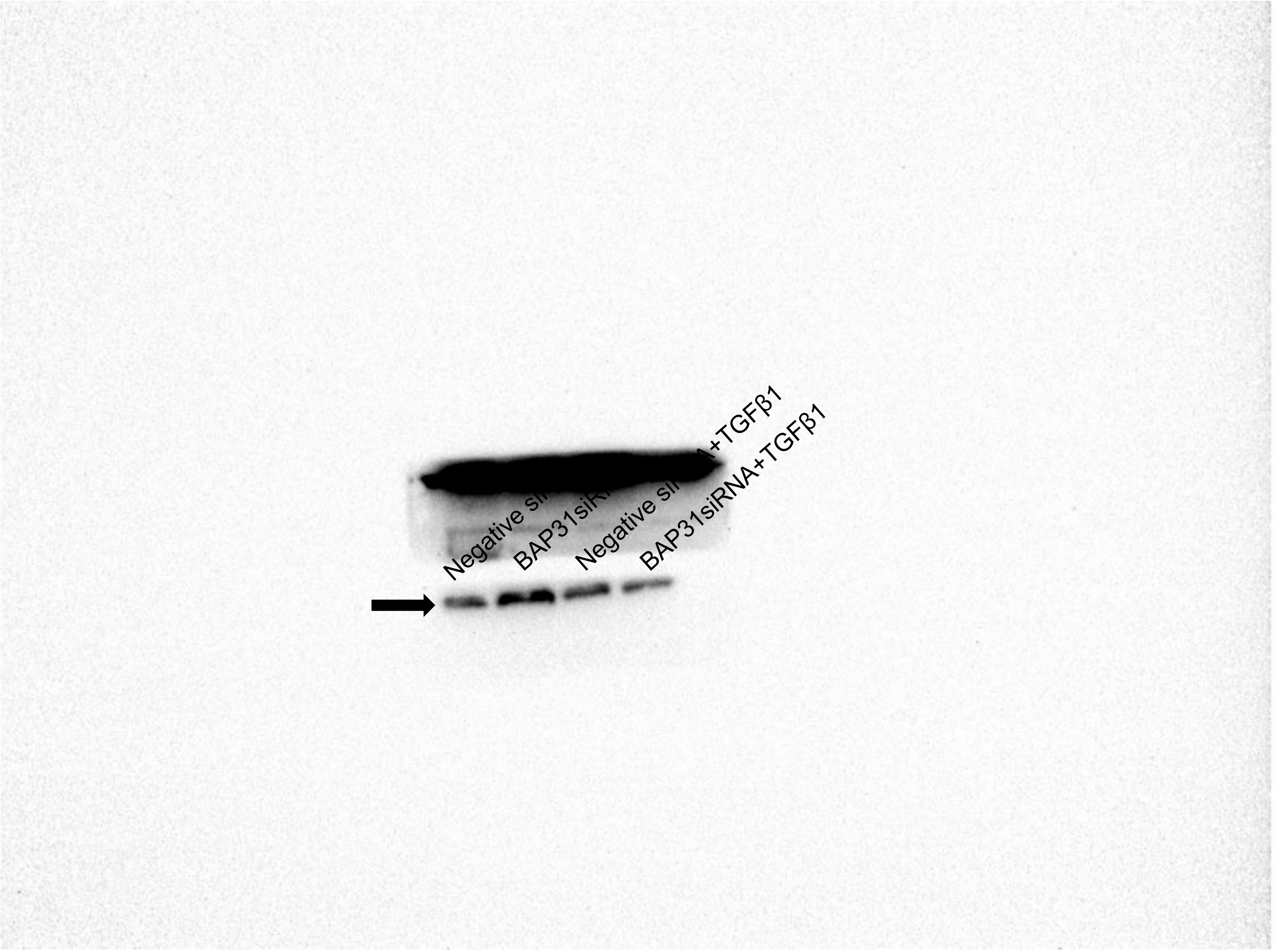

Fig6A lc3

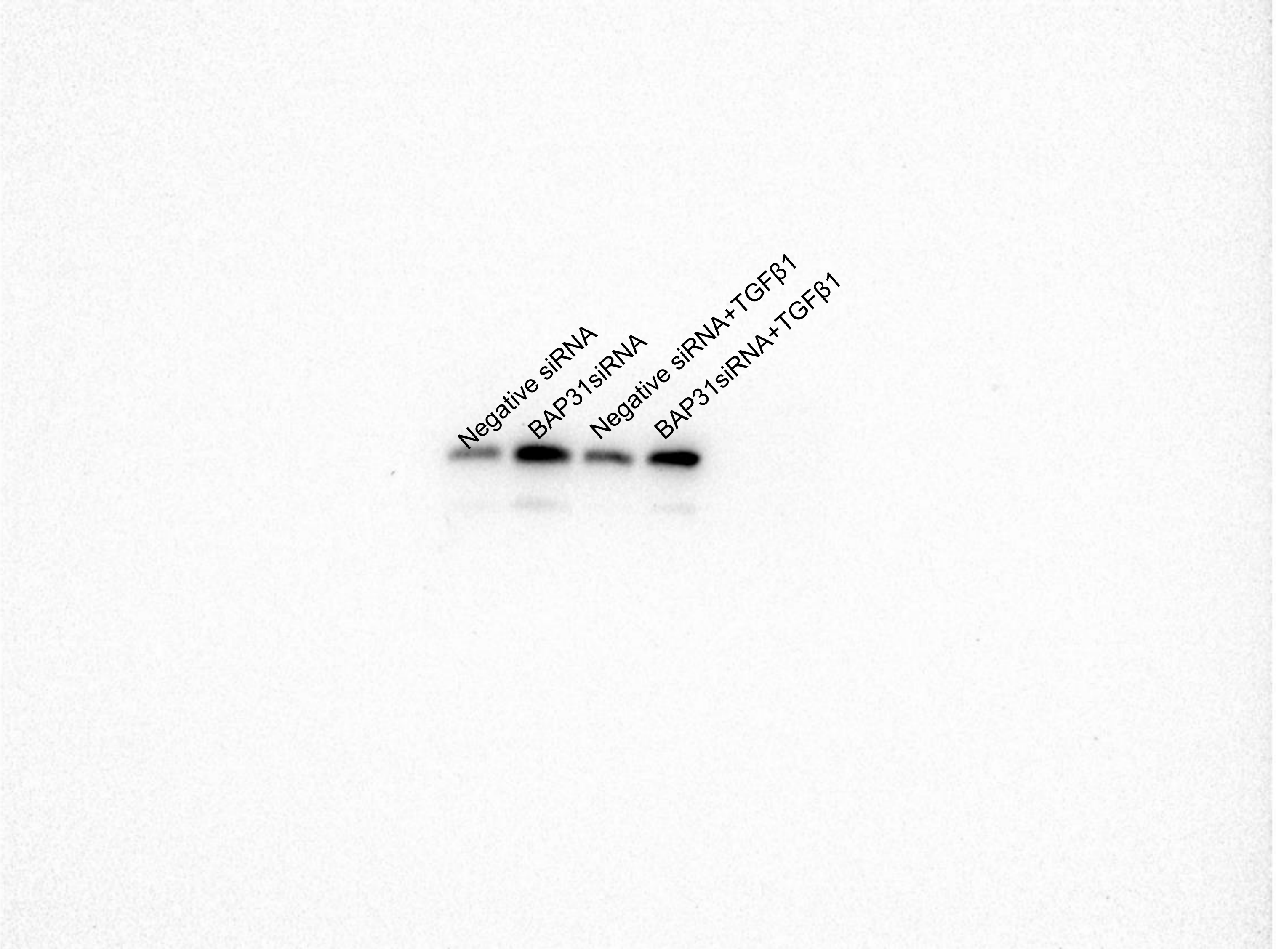
Negative siRNA
BAP31siRNA
Negative siRNA+TGFβ1
BAP31siRNA+TGFβ1

Fig6A vdac1

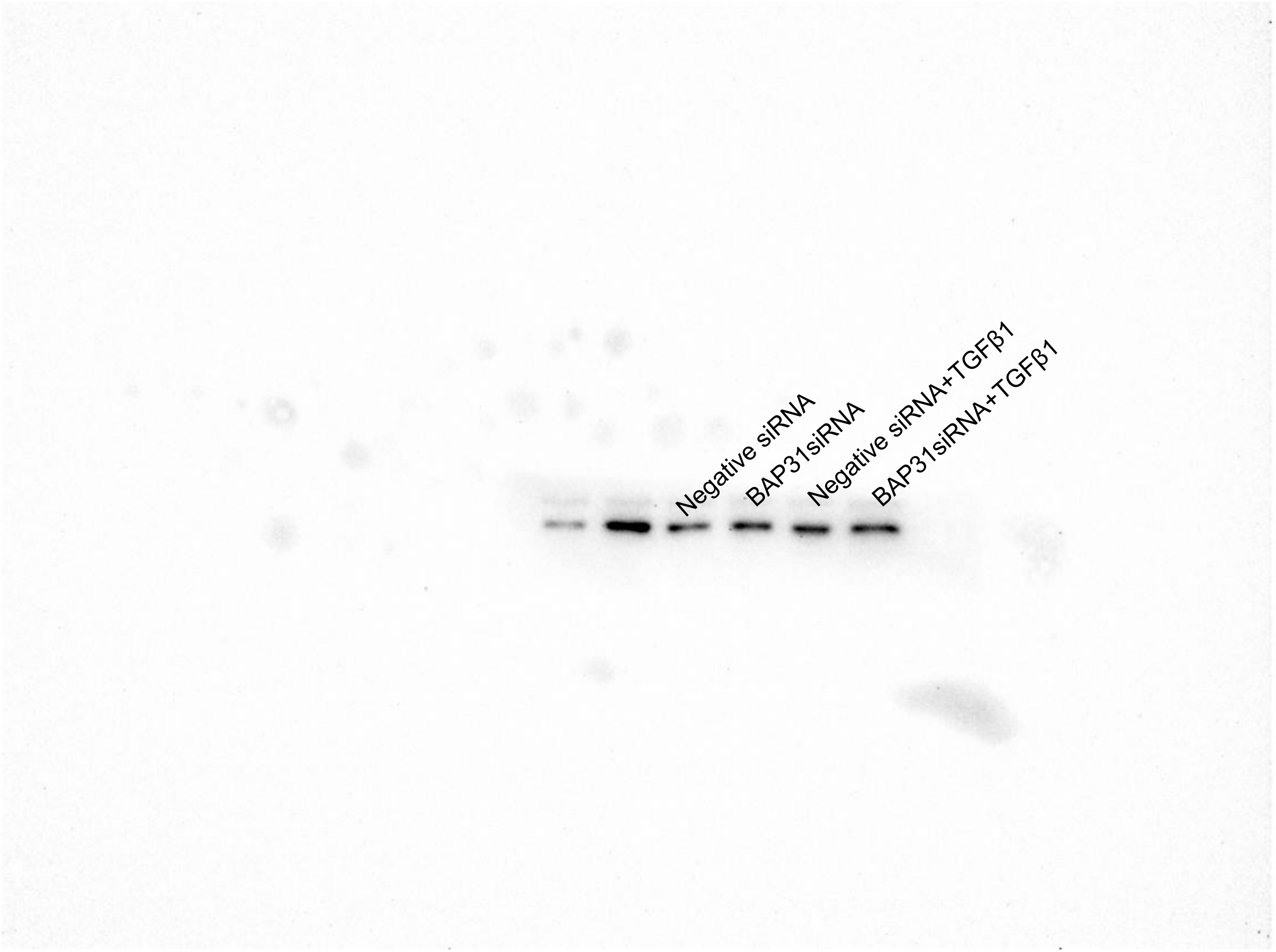
Negative siRNA
BAP31siRNA
Negative siRNA+TGFβ1
BAP31siRNA+TGFβ1

Fig6A bap31

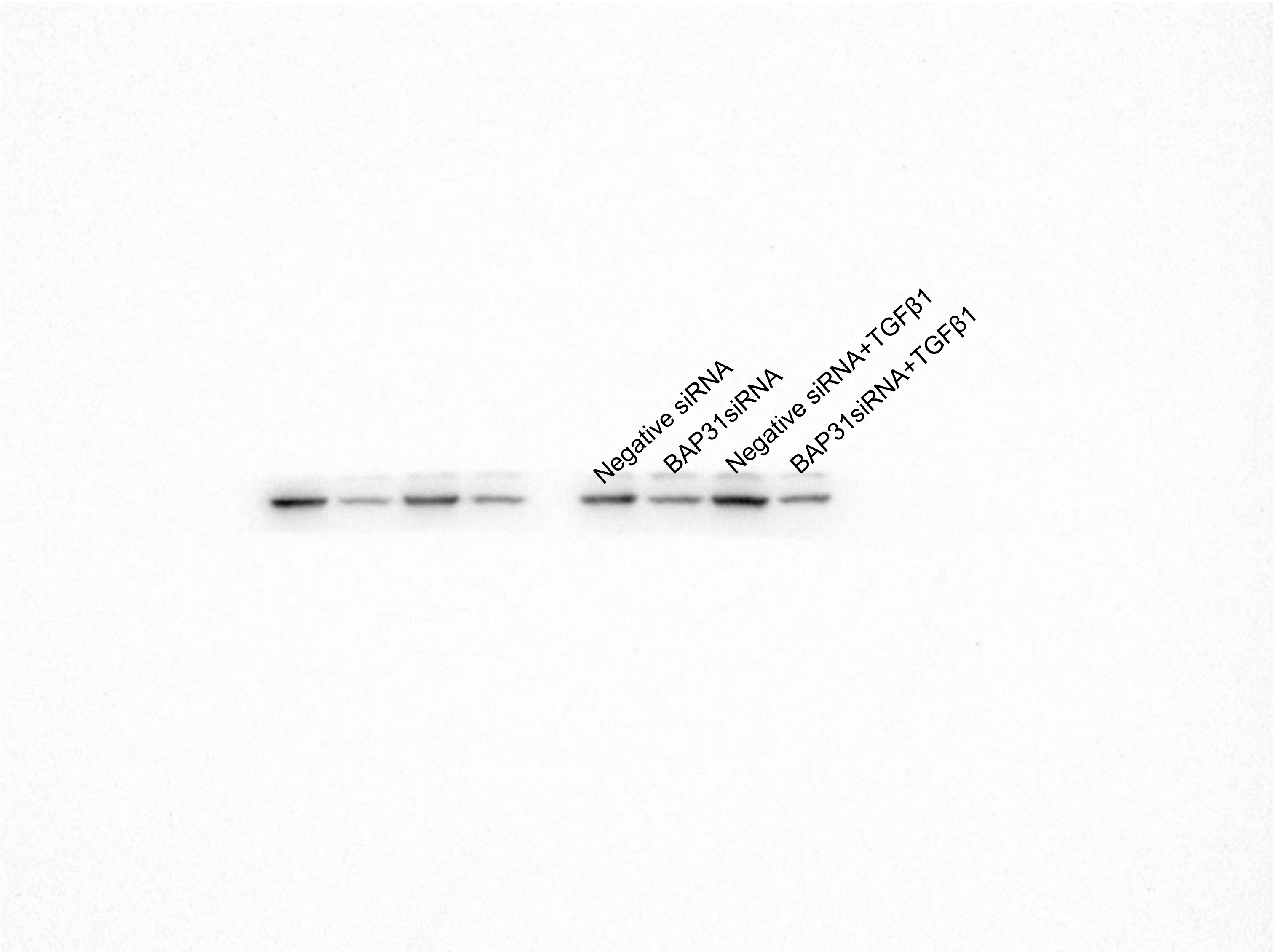
Negative siRNA
BAP31siRNA
Negative siRNA+TGFβ1
BAP31siRNA+TGFβ1

Fig6A beta-actin

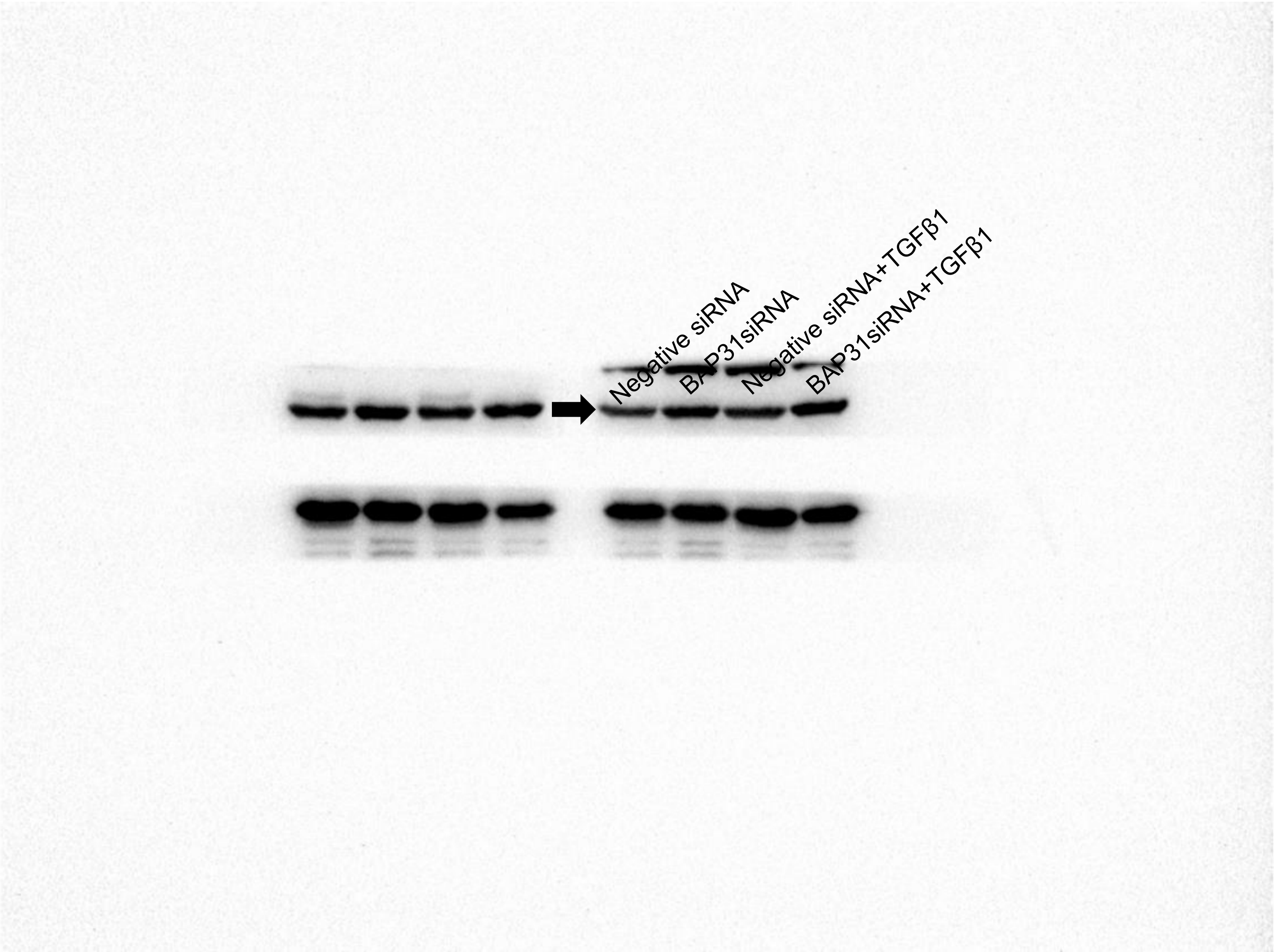

Fig6B

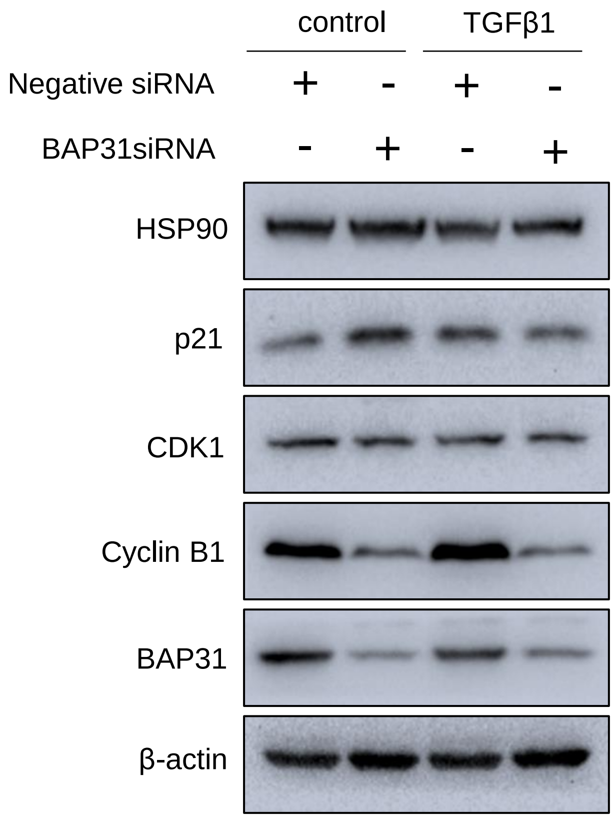
control
TGFβ1
Negative siRNA + - + -
BAP31siRNA - + - +
HSP90
p21
CDK1
Cyclin B1
BAP31
β-actin

Fig6B HSP90

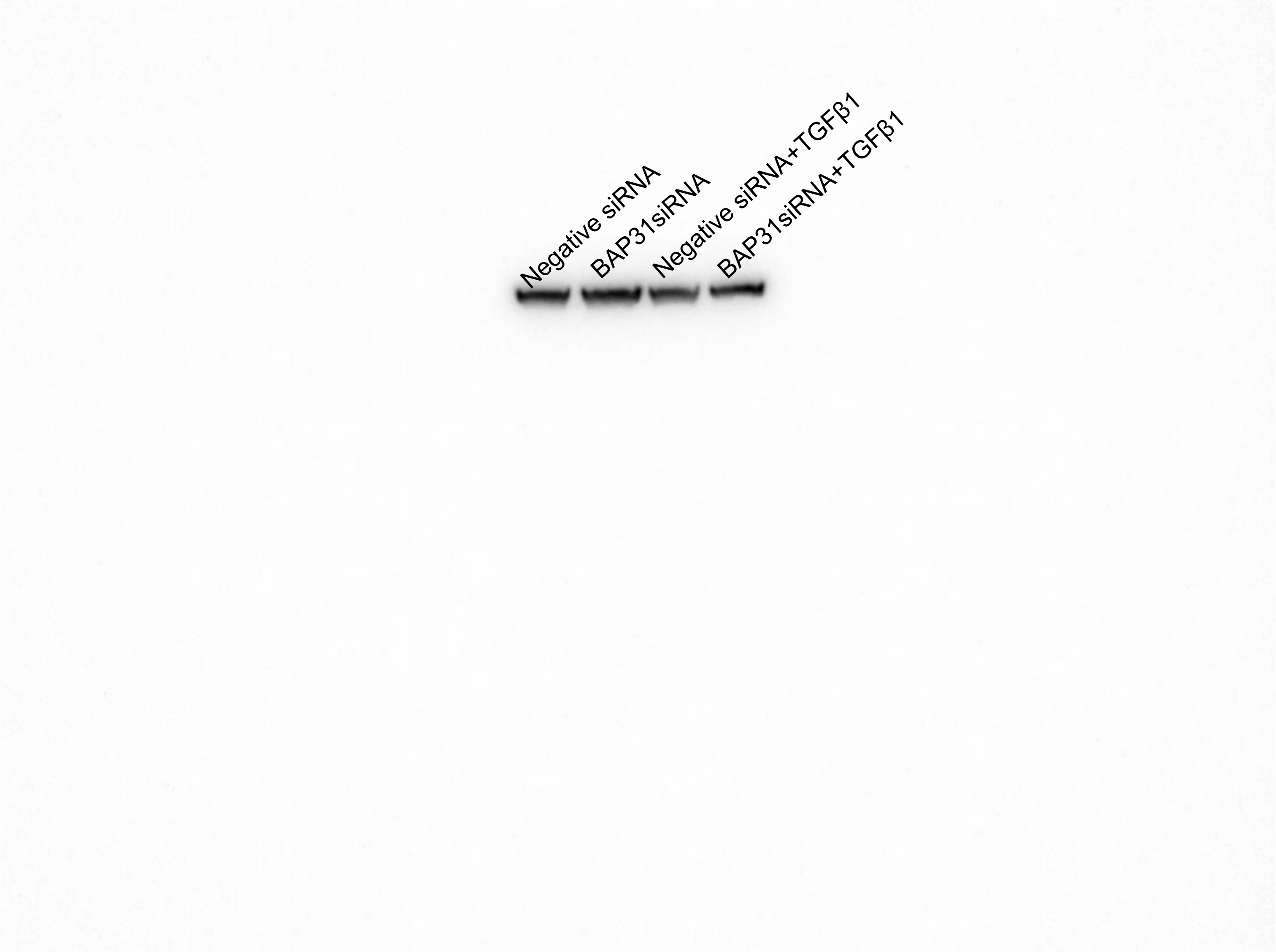
Negative siRNA
BAP31siRNA
Negative siRNA+TGFβ1
BAP31siRNA+TGFβ1

Fig6B p21

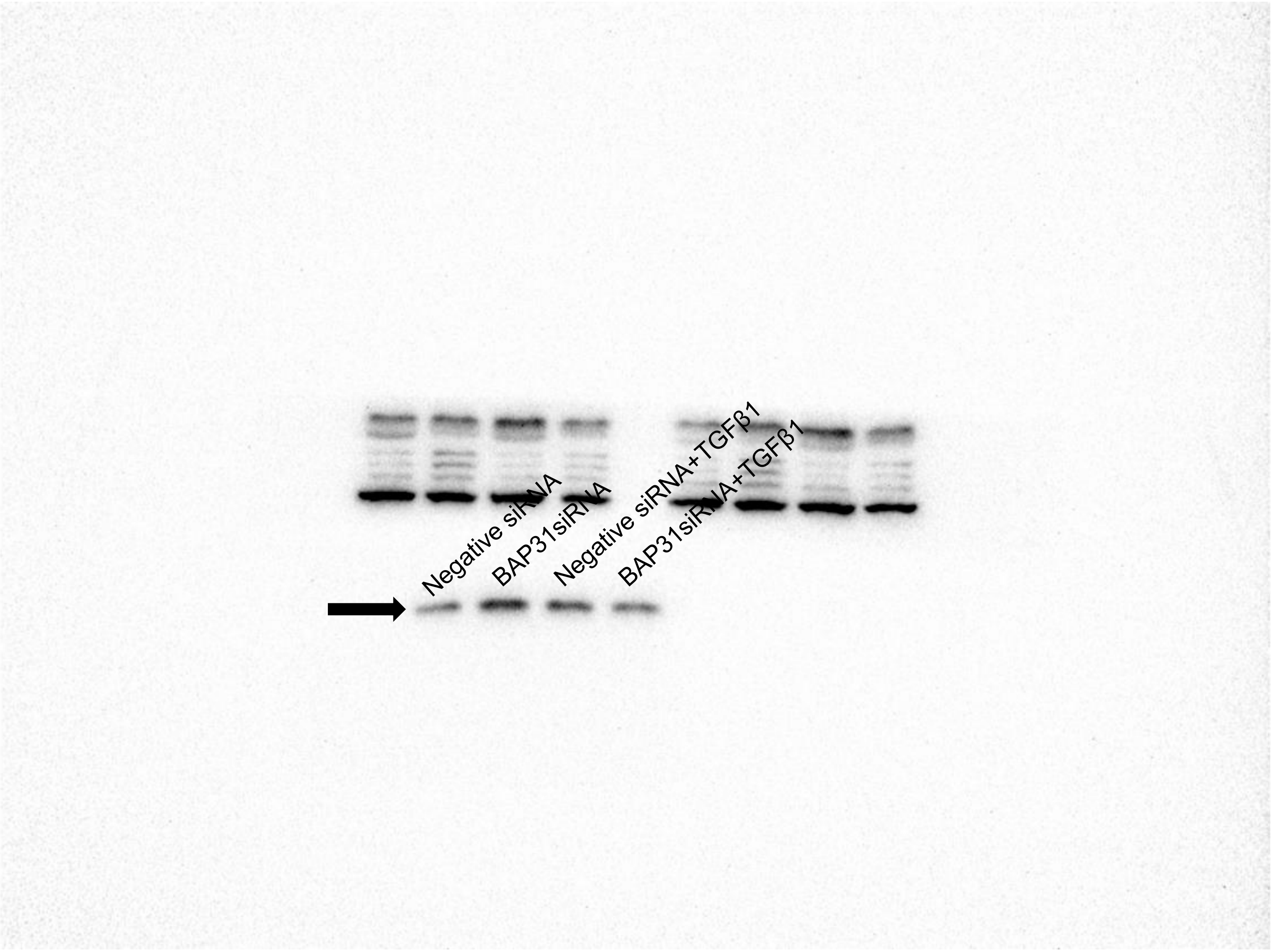
Negative siRNA
BAP31siRNA
Negative siRNA+TGFβ1
BAP31siRNA+TGFβ1

Fig6B CDK1

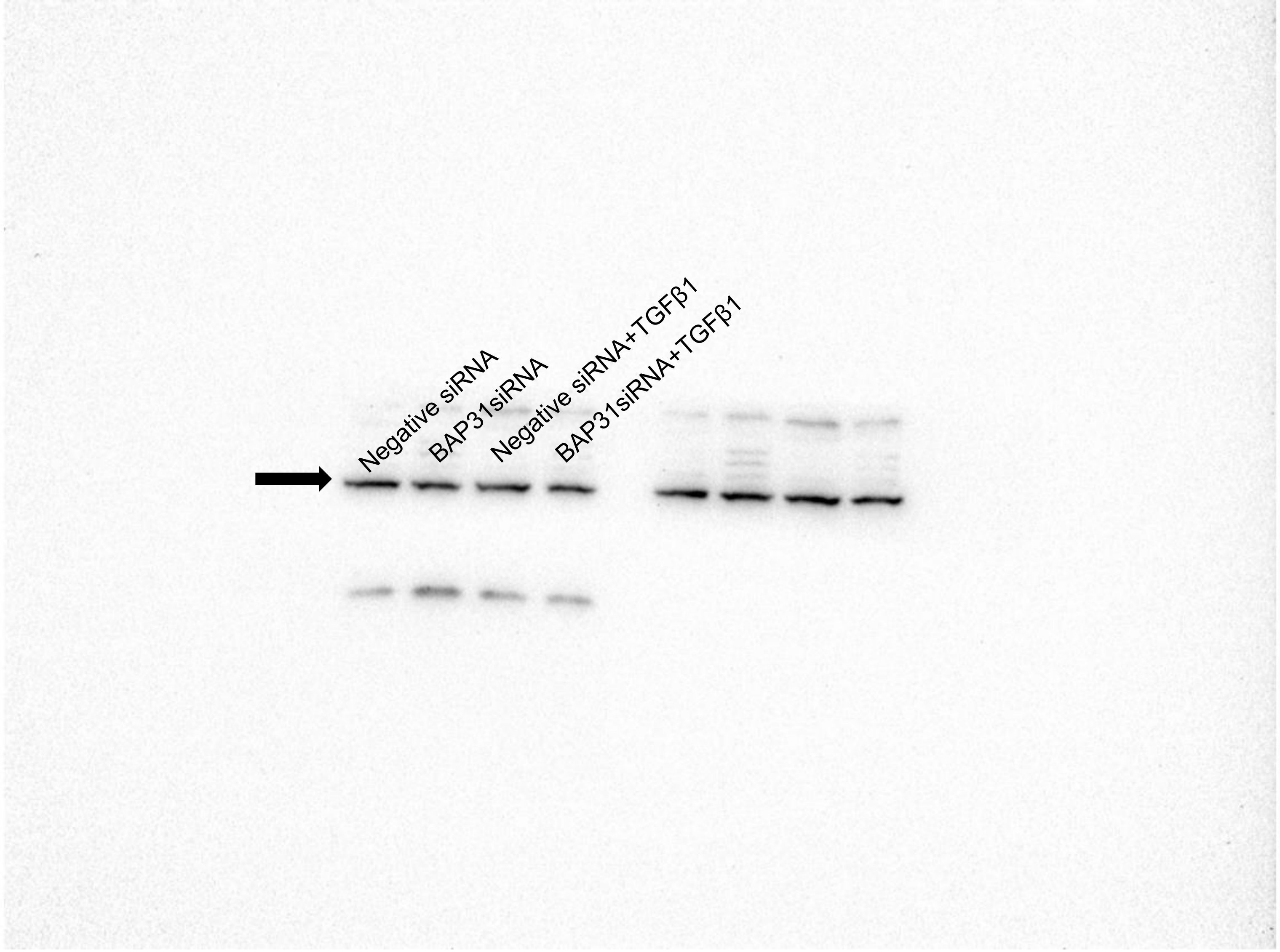
Negative siRNA
BAP31siRNA
Negative siRNA+TGFβ1
BAP31siRNA+TGFβ1

Fig6B cyclinb1

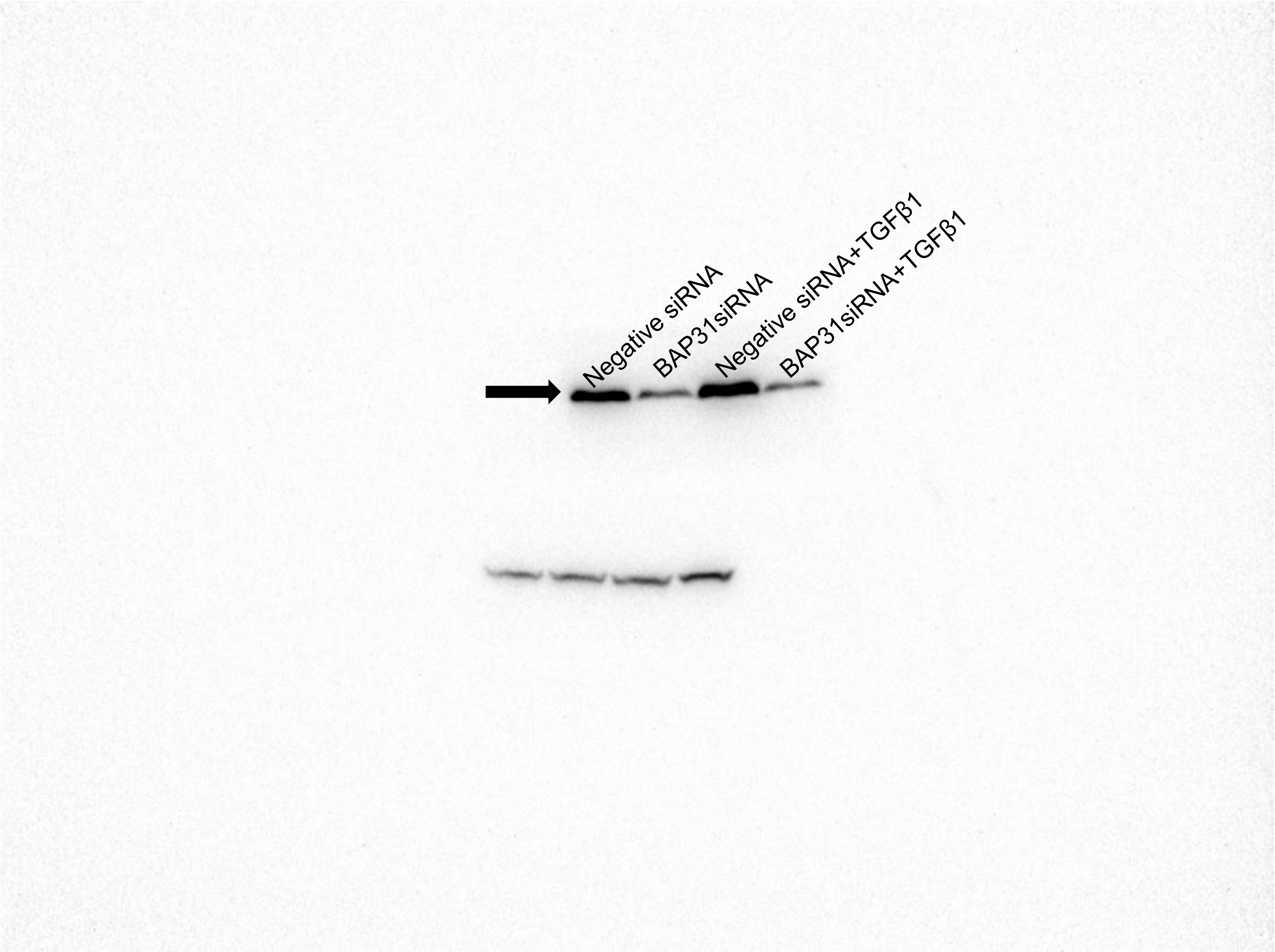

Fig6B bap31

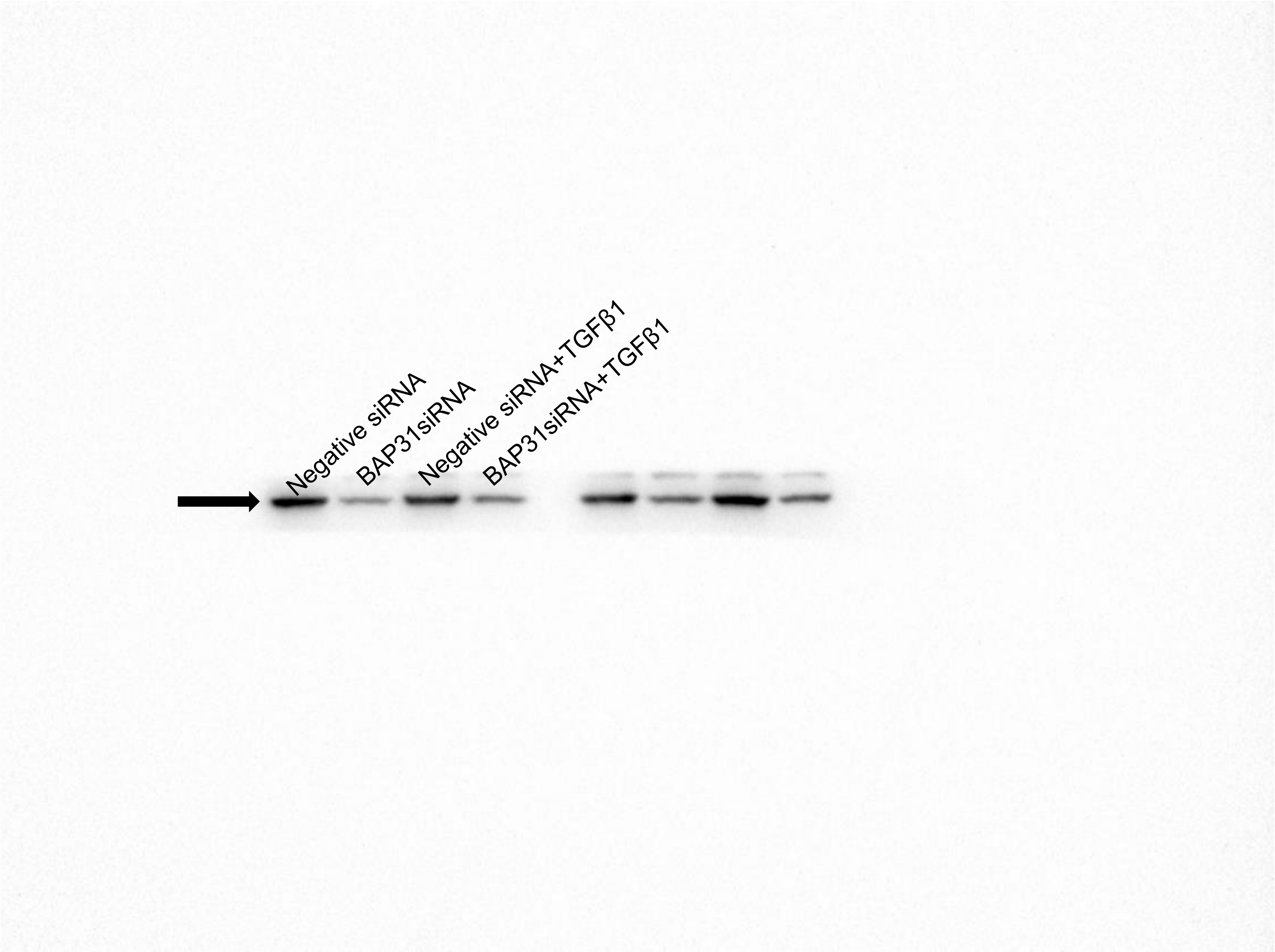
Negative siRNA
BAP31siRNA
Negative siRNA+TGFβ1
BAP31siRNA+TGFβ1

Fig6B beta-actin

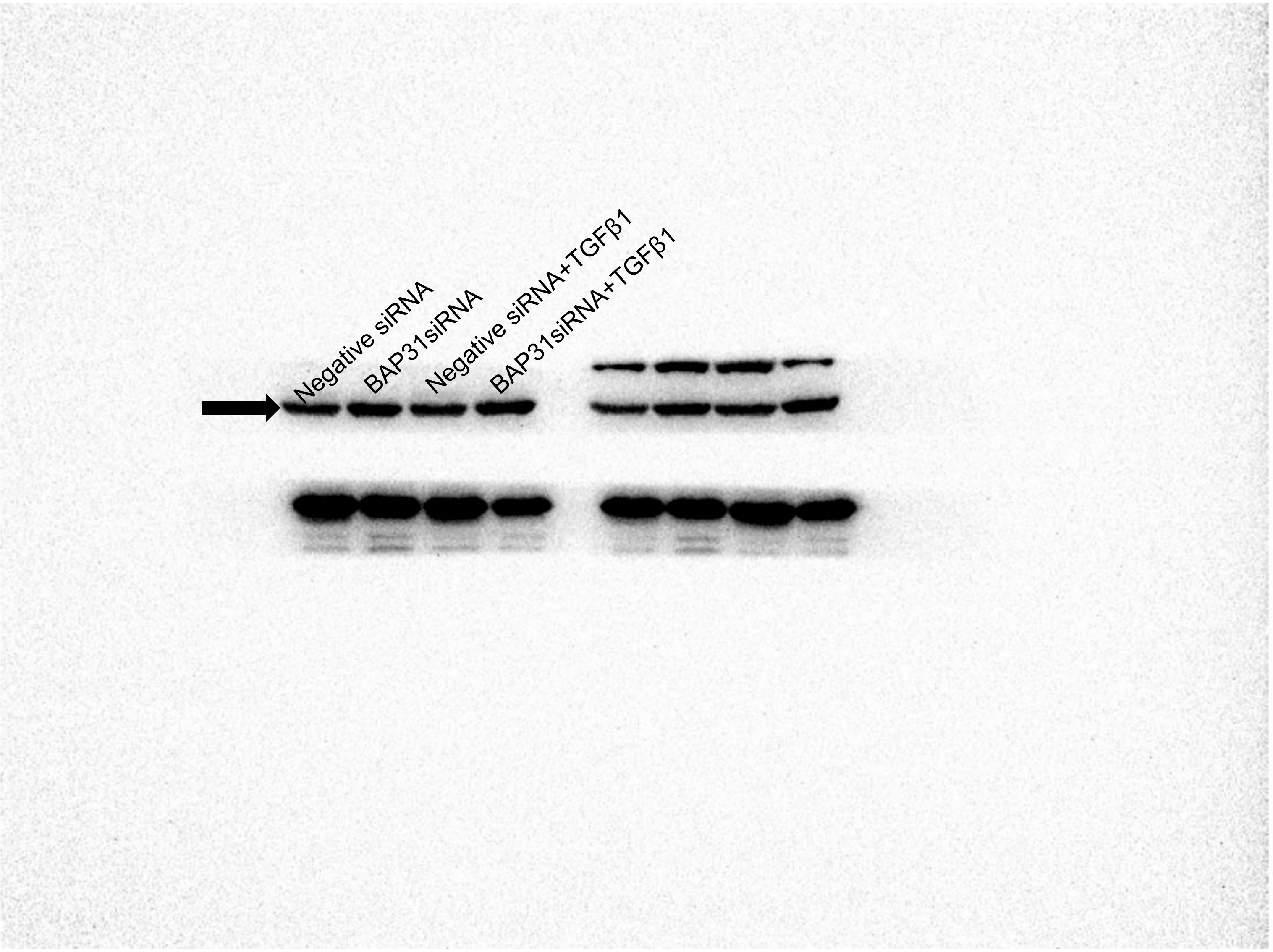

Fig6C

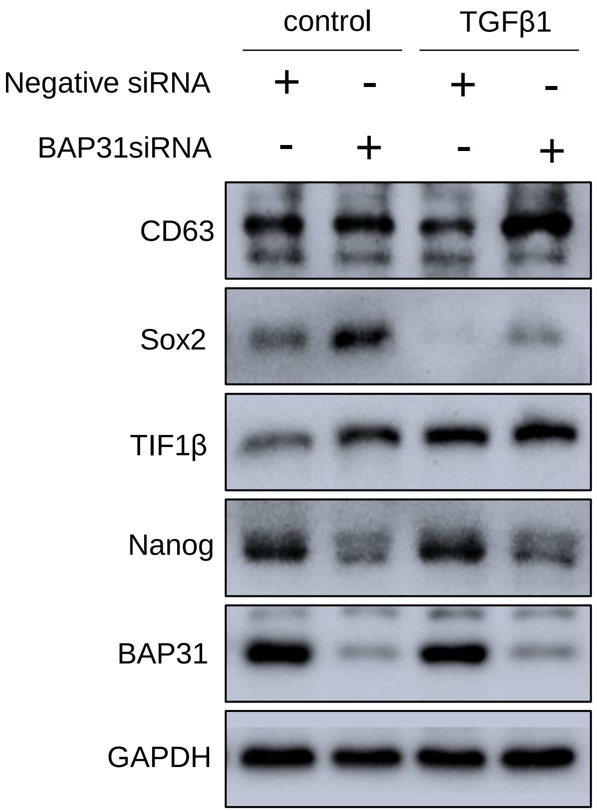
control
TGFβ1
Negative siRNA + - + -
BAP31siRNA - + - +
CD63
Sox2
TIF1β
Nanog
BAP31
GAPDH

Fig6C CD63

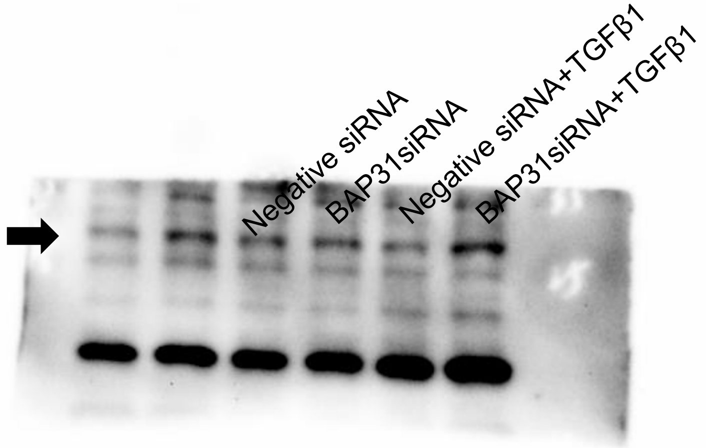
Negative siRNA
BAP31siRNA
Negative siRNA+TGFβ1
BAP31siRNA+TGFβ1

Fig6C sox2

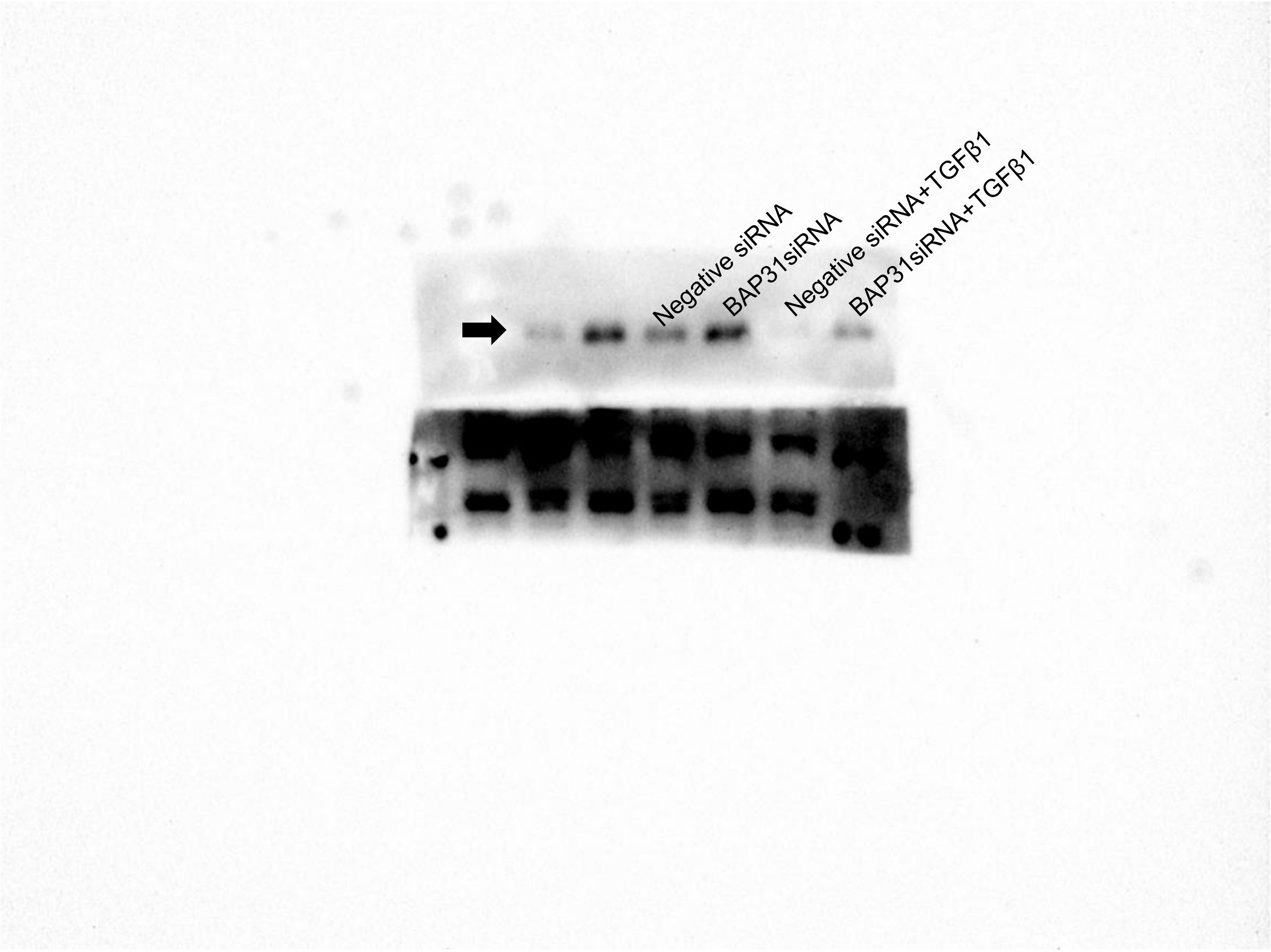
Negative siRNA
BAP31siRNA
Negative siRNA+TGFβ1
BAP31siRNA+TGFβ1

Fig6C TIF1β

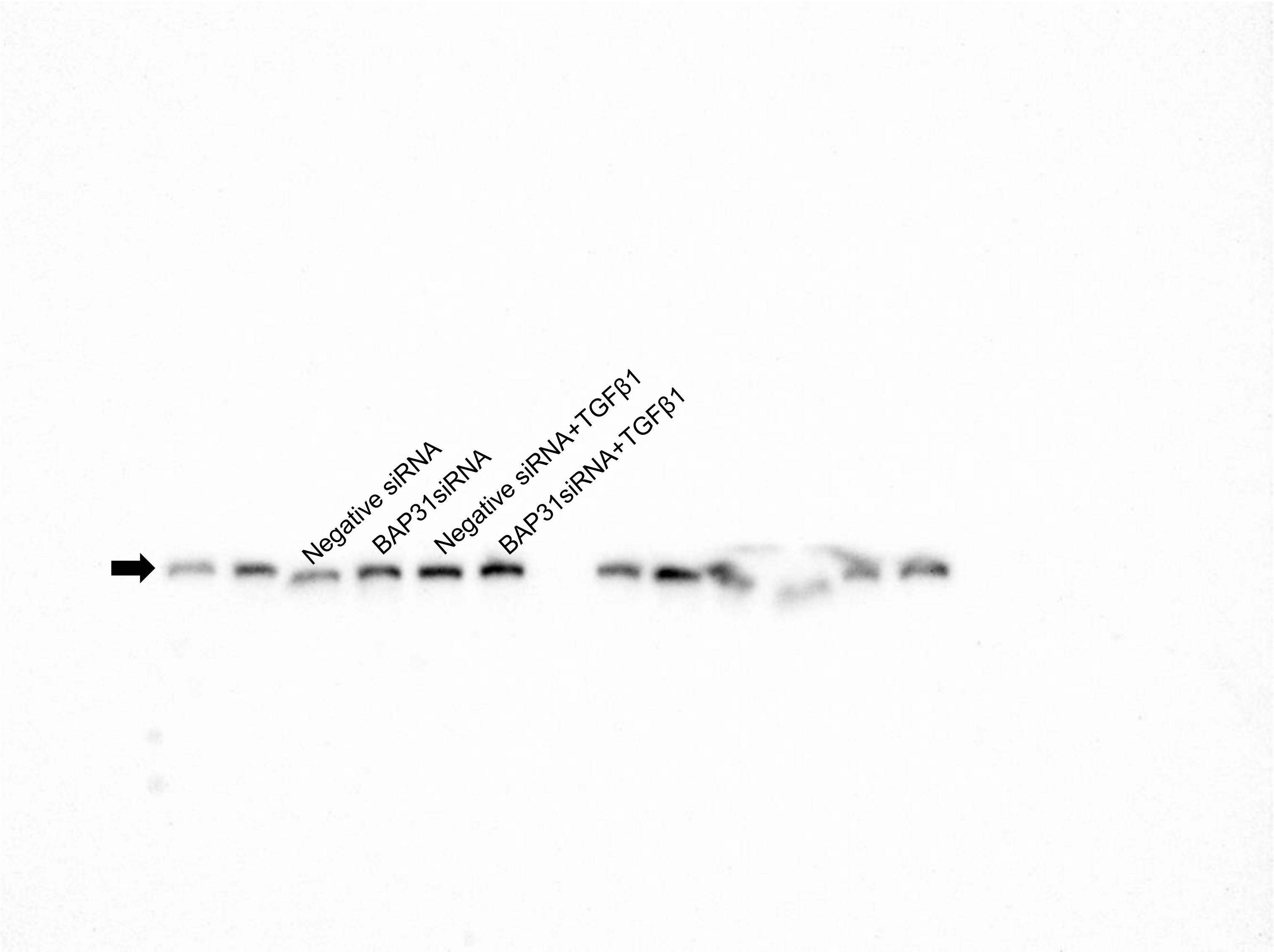
Negative siRNA
BAP31siRNA
Negative siRNA+TGFβ1
BAP31siRNA+TGFβ1

Fig6C nanog

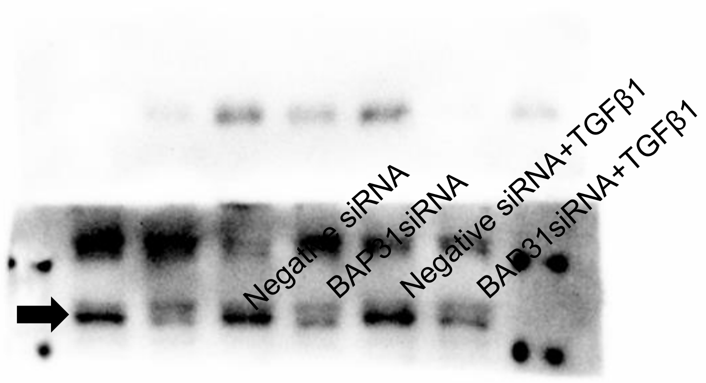
Negative siRNA
BAP31siRNA
Negative siRNA+TGFβ1
BAP31siRNA+TGFβ1

Fig6C BAP31

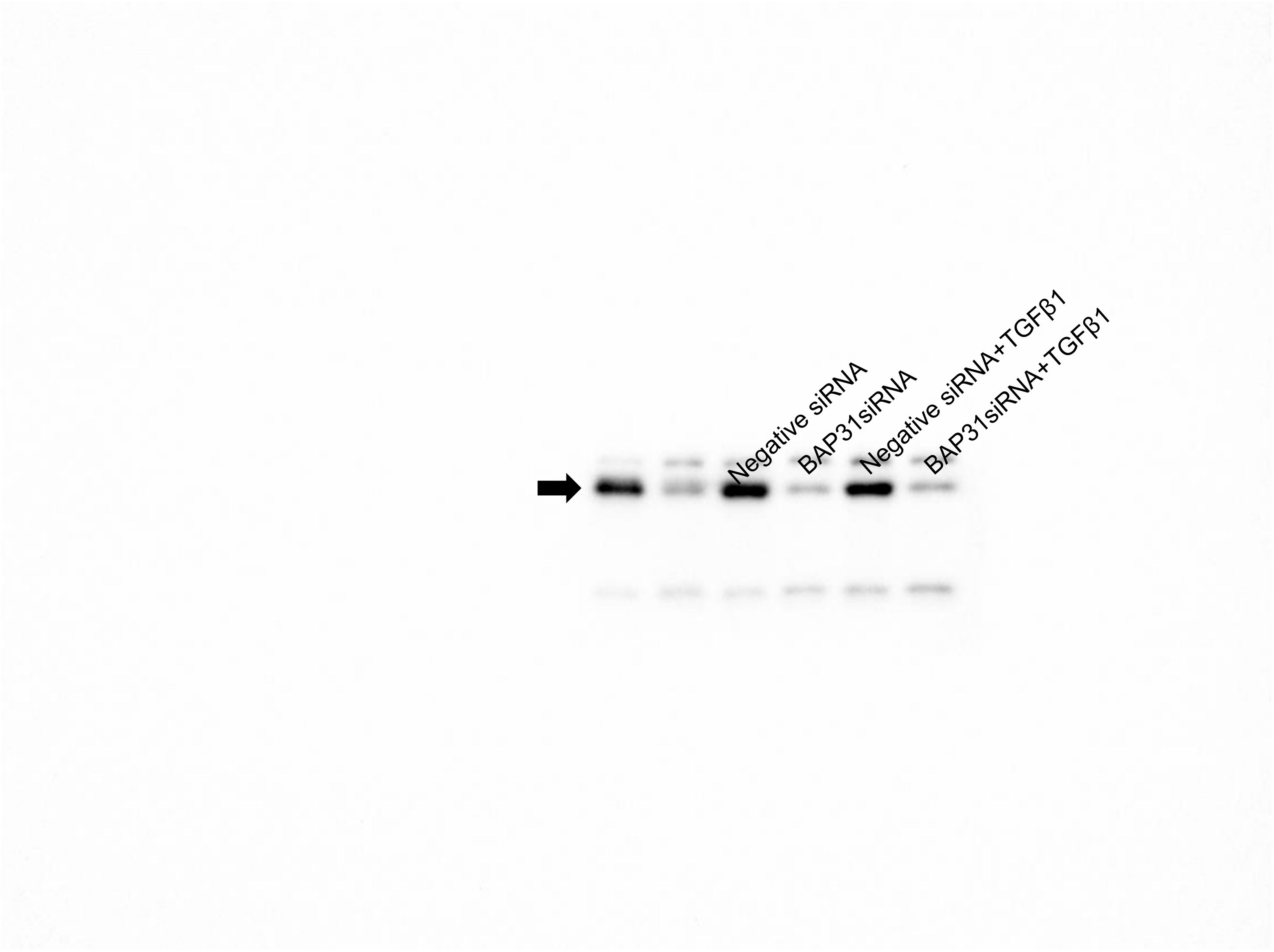
Negative siRNA
BAP31siRNA
Negative siRNA+TGFβ1
BAP31siRNA+TGFβ1

Fig6C gapdh

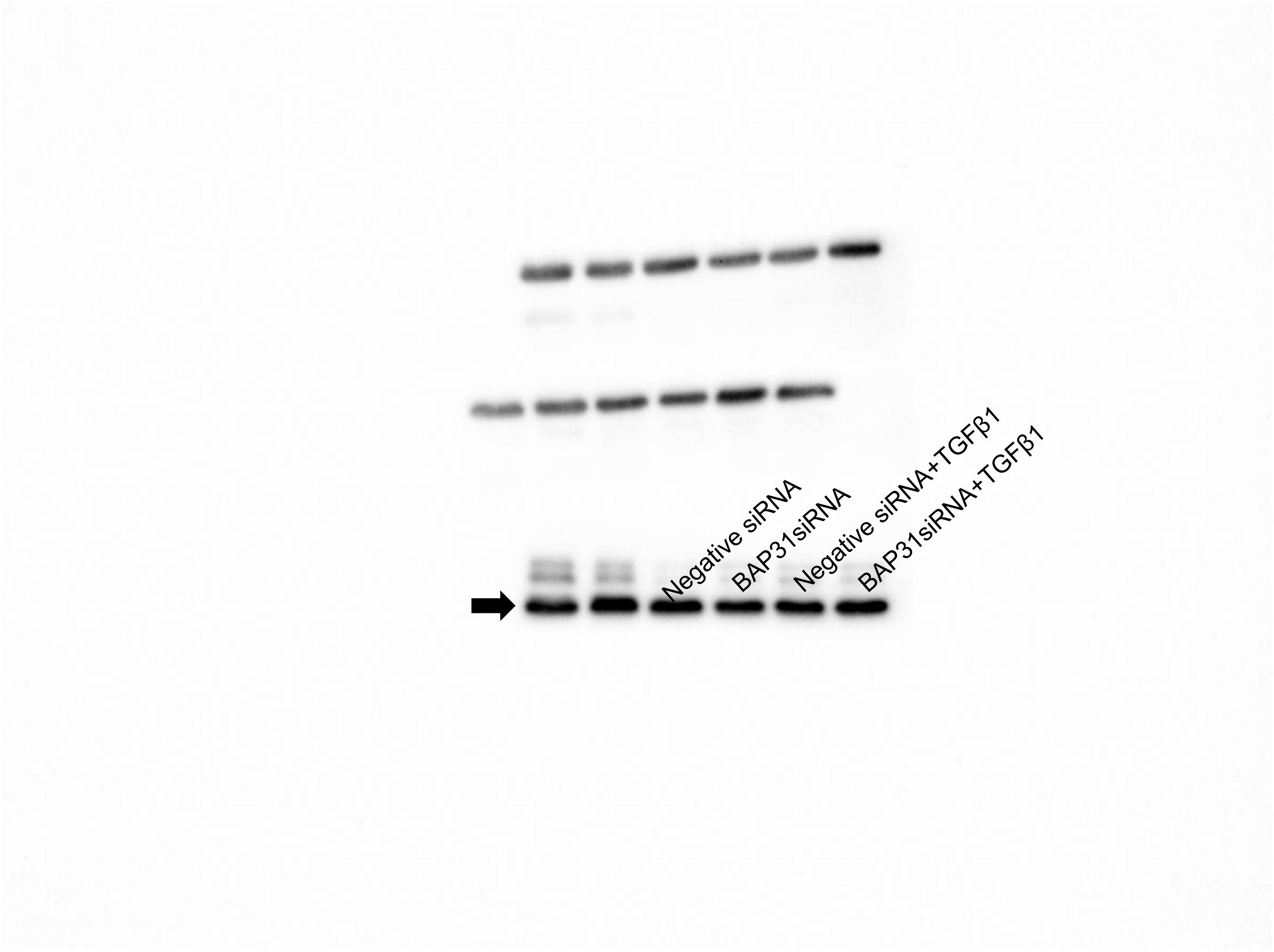
Negative siRNA
BAP31siRNA
Negative siRNA+TGFβ1
BAP31siRNA+TGFβ1

Fig6D

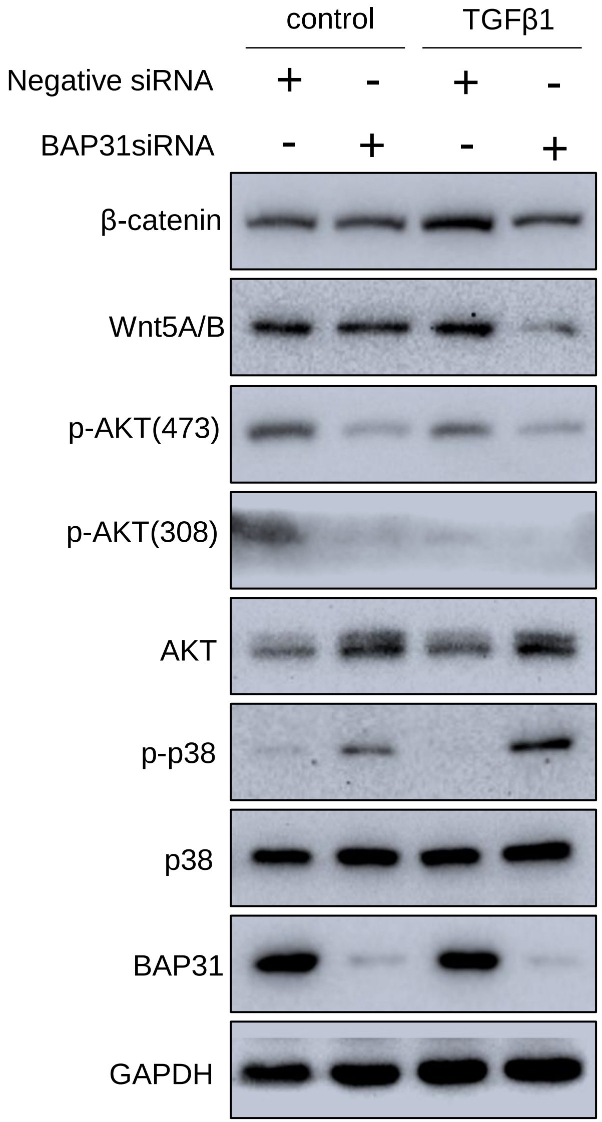
control
TGFβ1
Negative siRNA + - + -
BAP31siRNA - + - +
β-catenin
Wnt5A/B
p-AKT(473)
p-AKT(308)
AKT
p-p38
p38
BAP31
GAPDH

Fig6D β-catenin

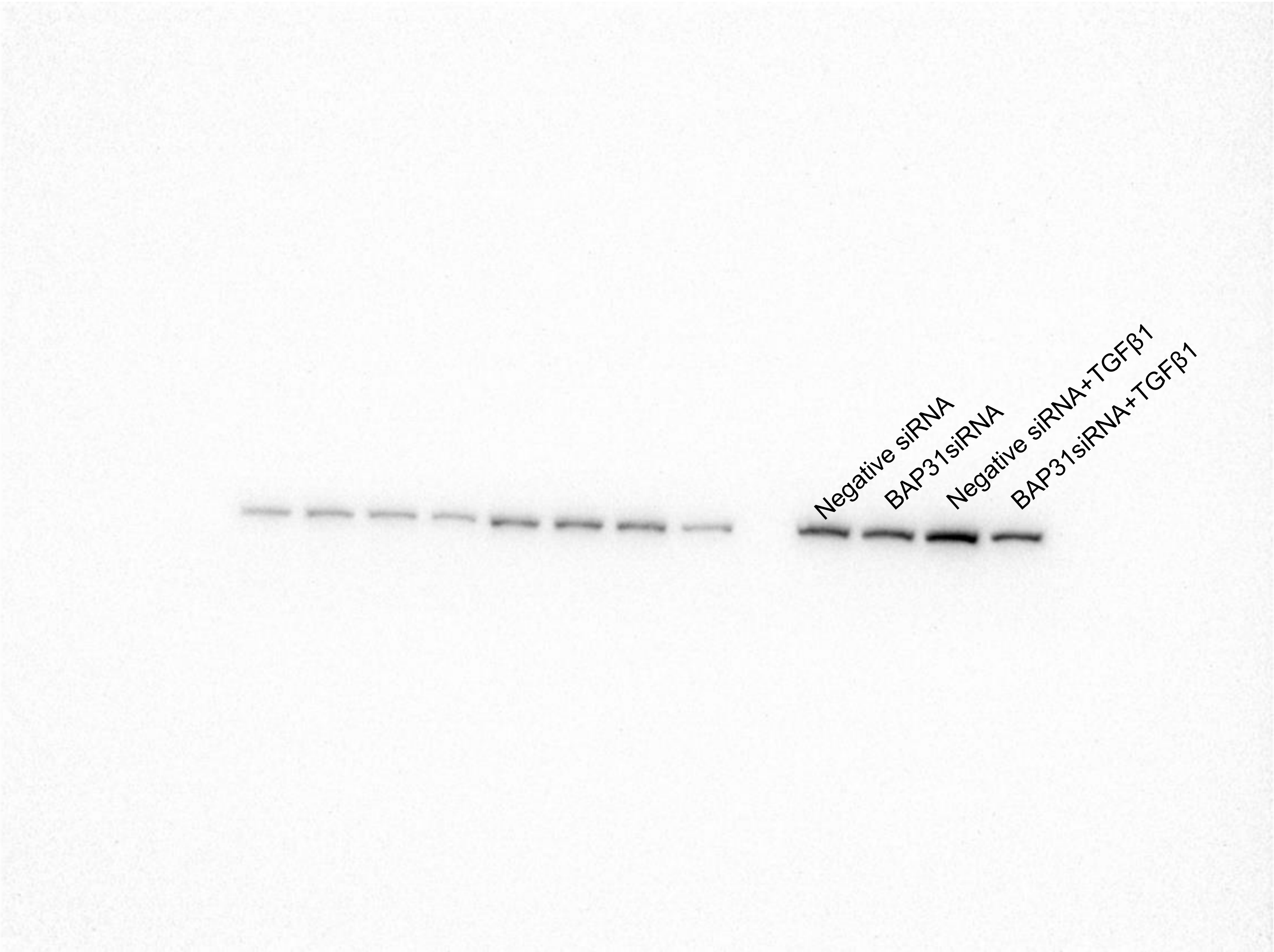

Fig6D wnt5ab

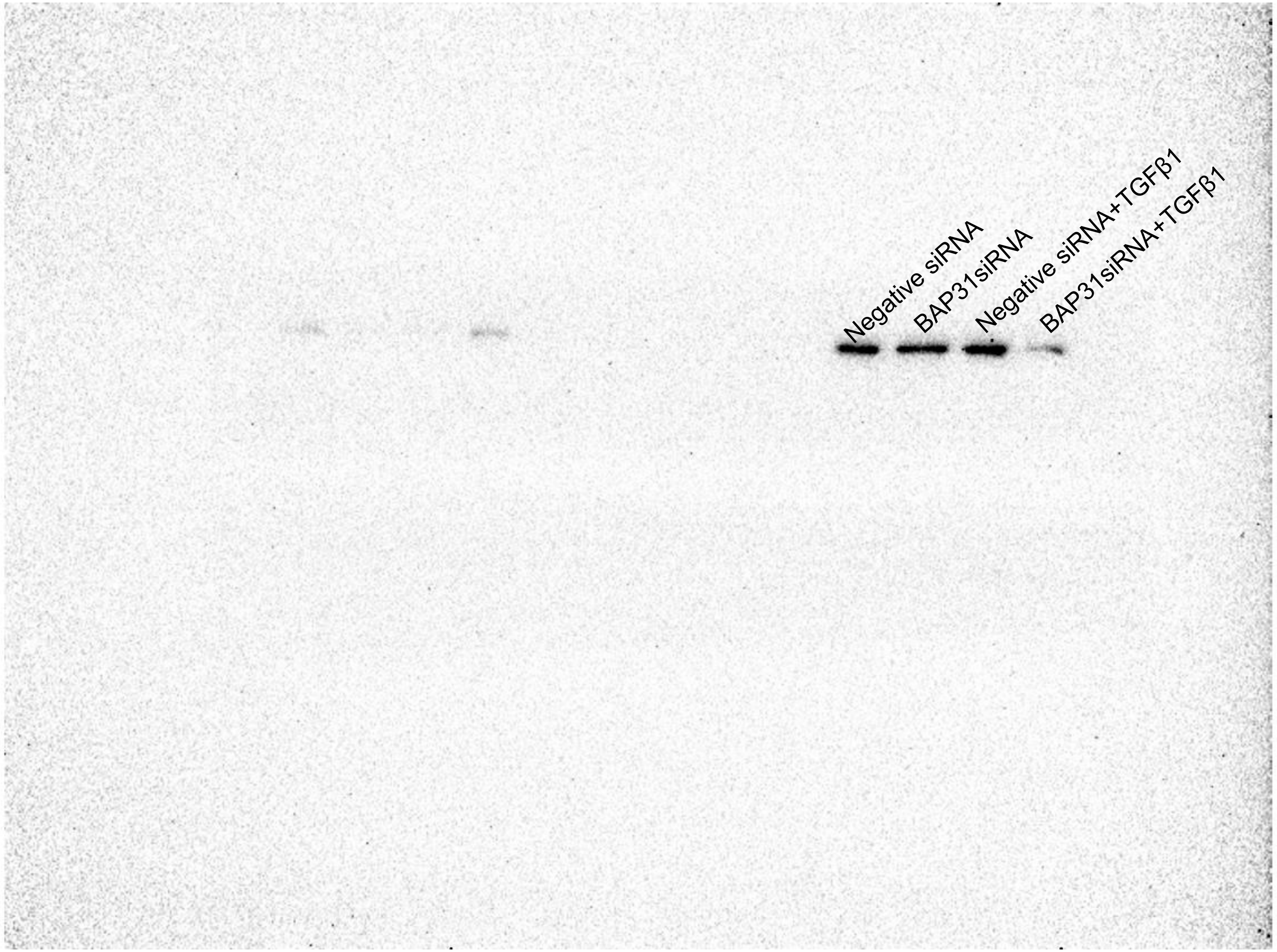

Fig6D p-akt(473)

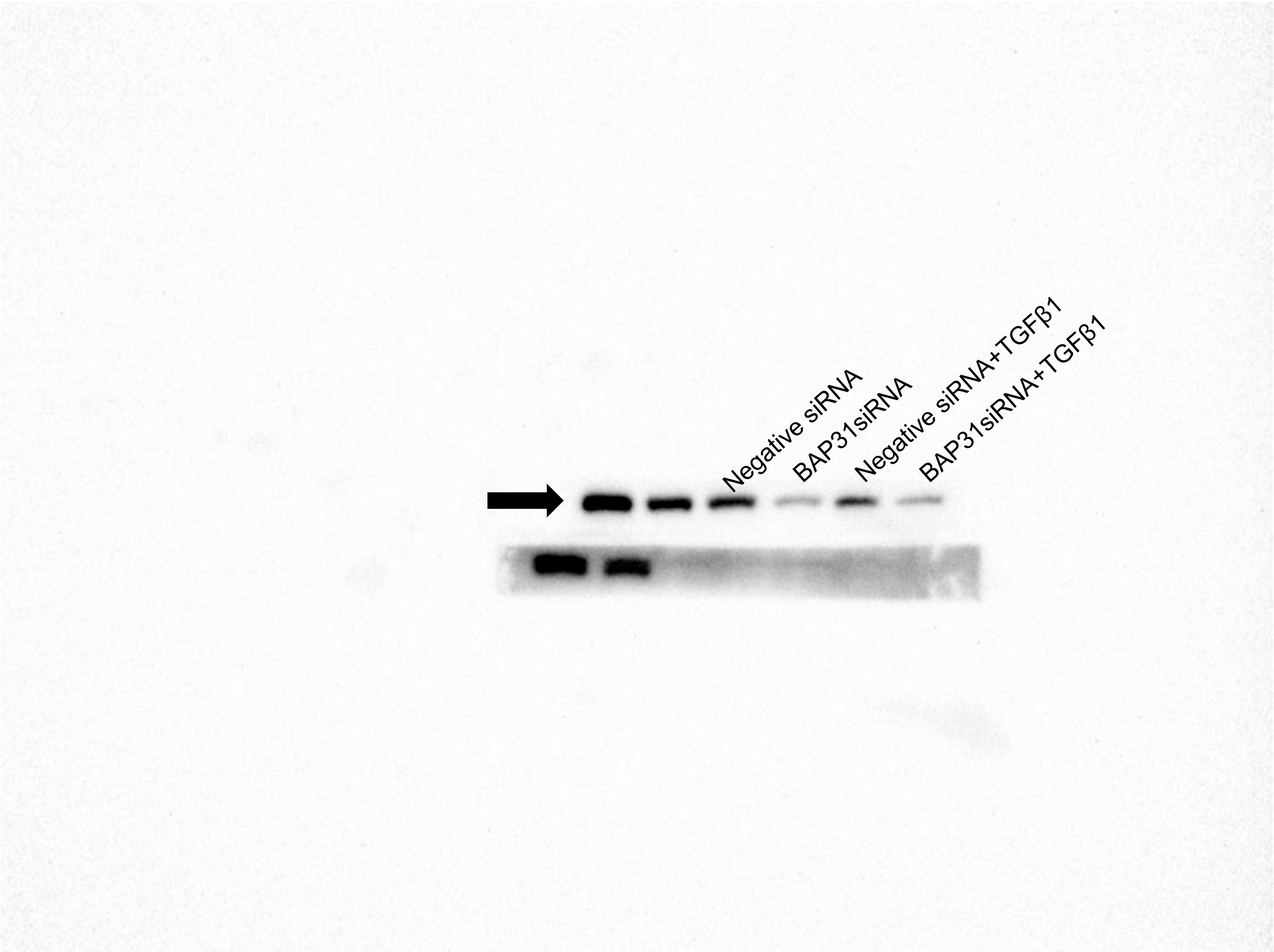

Fig6D p-AKT(308)

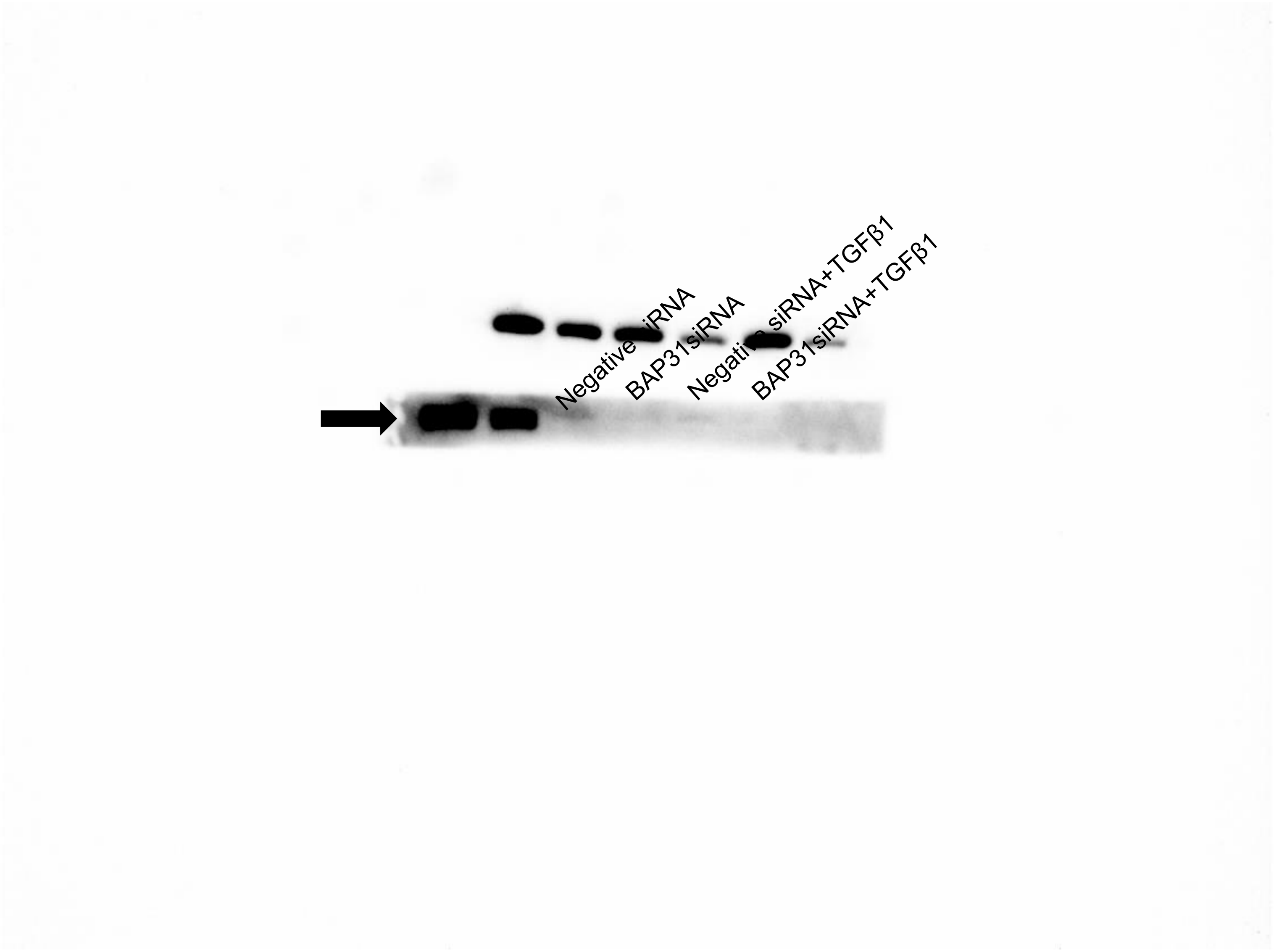

Fig6D AKT

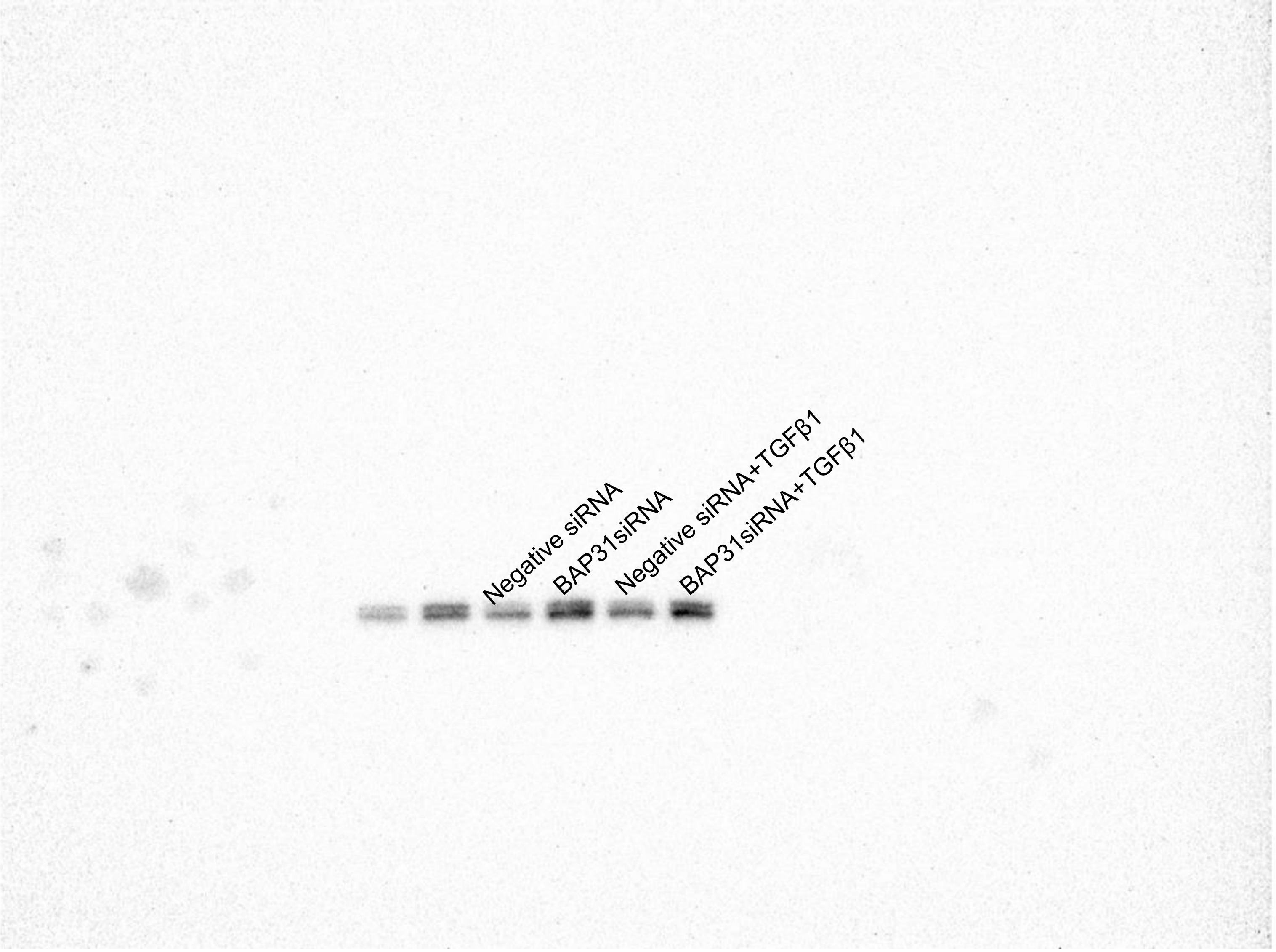
Negative siRNA
BAP31siRNA
Negative siRNA+TGFβ1
BAP31siRNA+TGFβ1

Fig6D p-p38

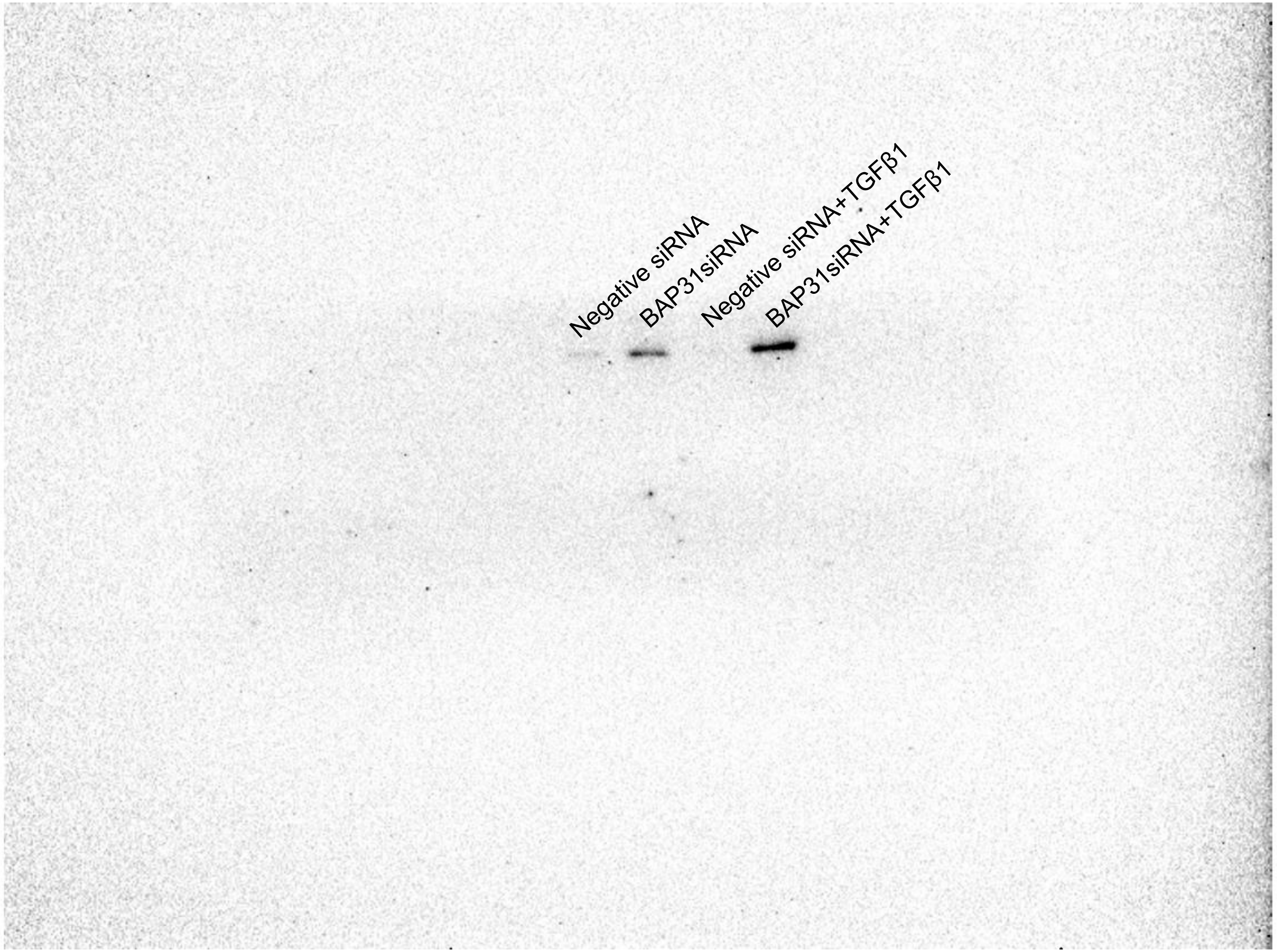
Negative siRNA
BAP31siRNA
Negative siRNA+TGFβ1
BAP31siRNA+TGFβ1

Fig6D p38

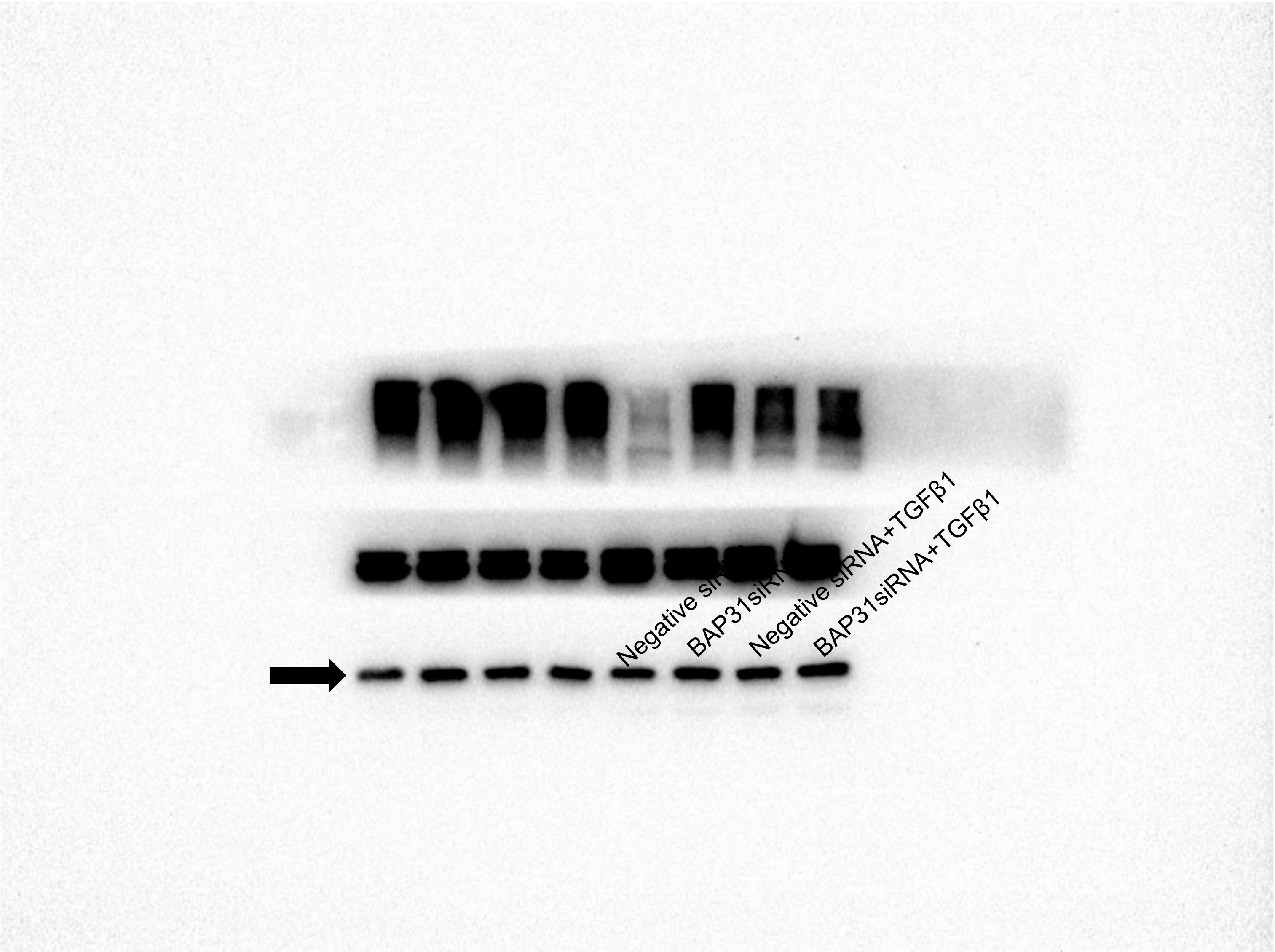
Negative si
BAP31siR
Negative siRNA+TGFβ1
BAP31siRNA+TGFβ1

Fig6D bap31

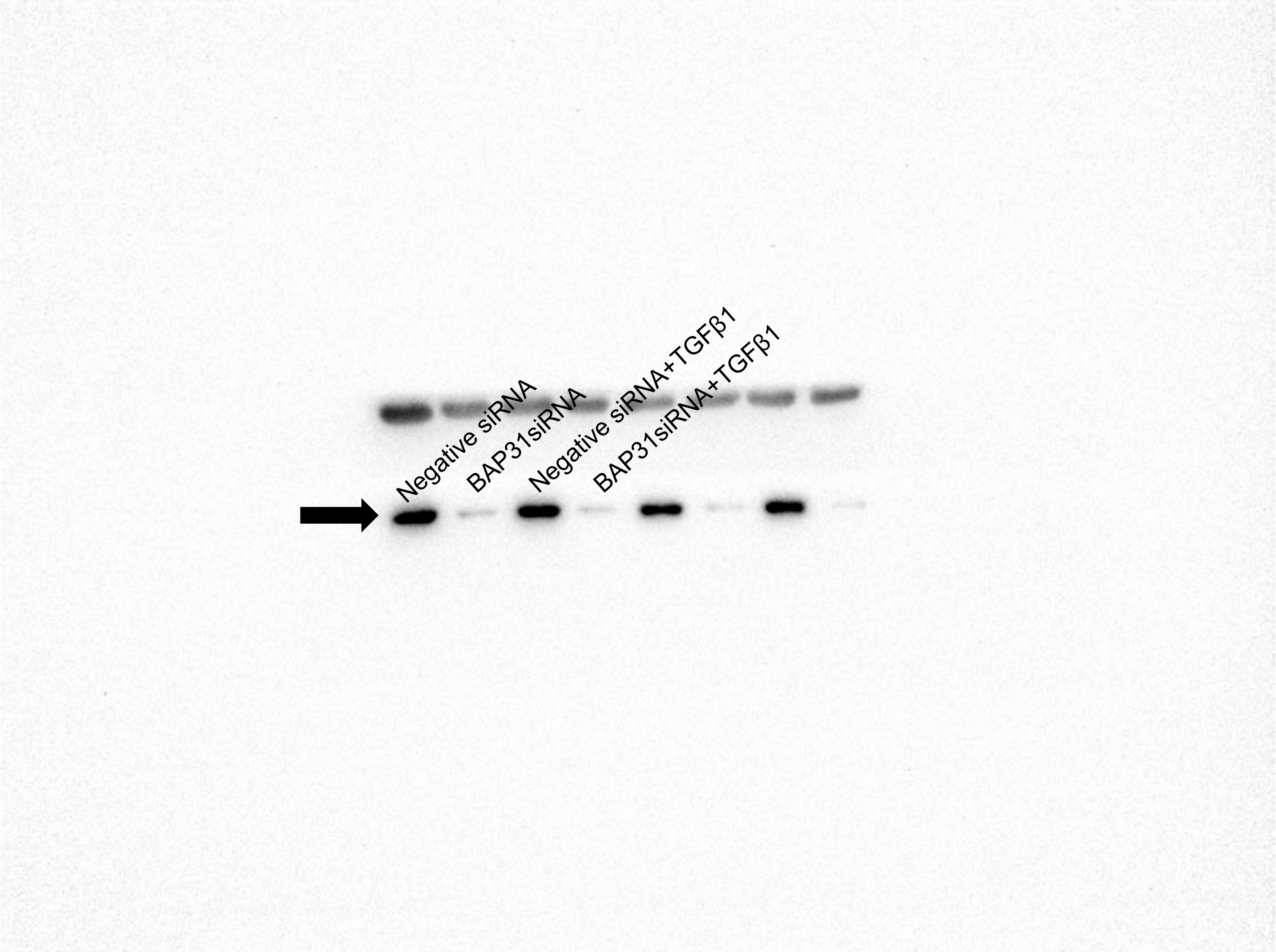
Negative siRNA
BAP31siRNA
Negative siRNA+TGFβ1
BAP31siRNA+TGFβ1

Fig6D gapdh

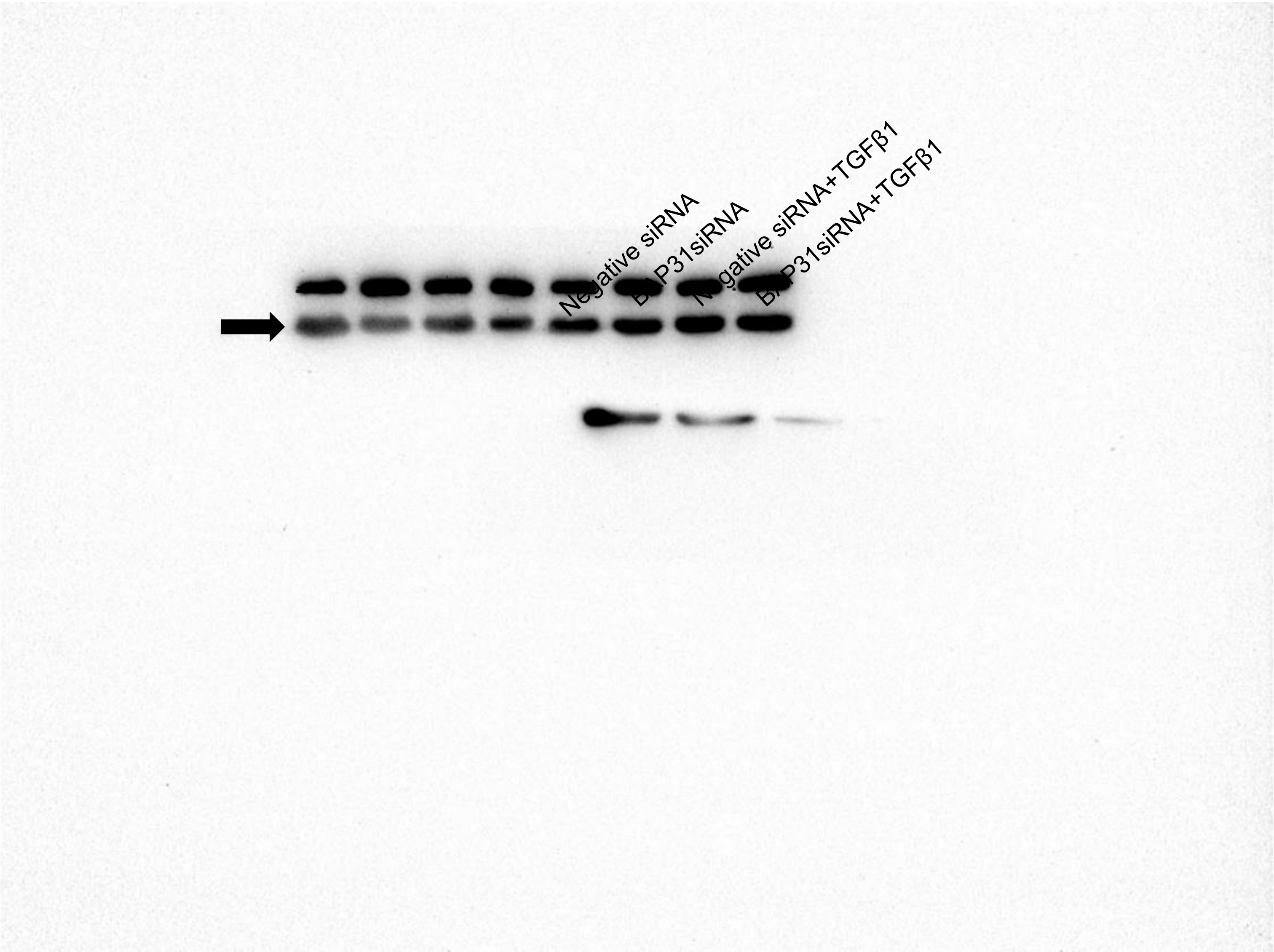
Negative siRNA
BAP31siRNA
Negative siRNA+TGFβ1
BAP31siRNA+TGFβ1
