# Supplementary figures and images for: BAP31 Regulates Wnt Signaling to Modulate Cell Migration in Lung Cancer
Source: Front Oncol. 2022 Mar 10;12:859195. doi: 10.3389/fonc.2022.859195 (PMC8960194; doi:10.3389/fonc.2022.859195)

# Overall Survival

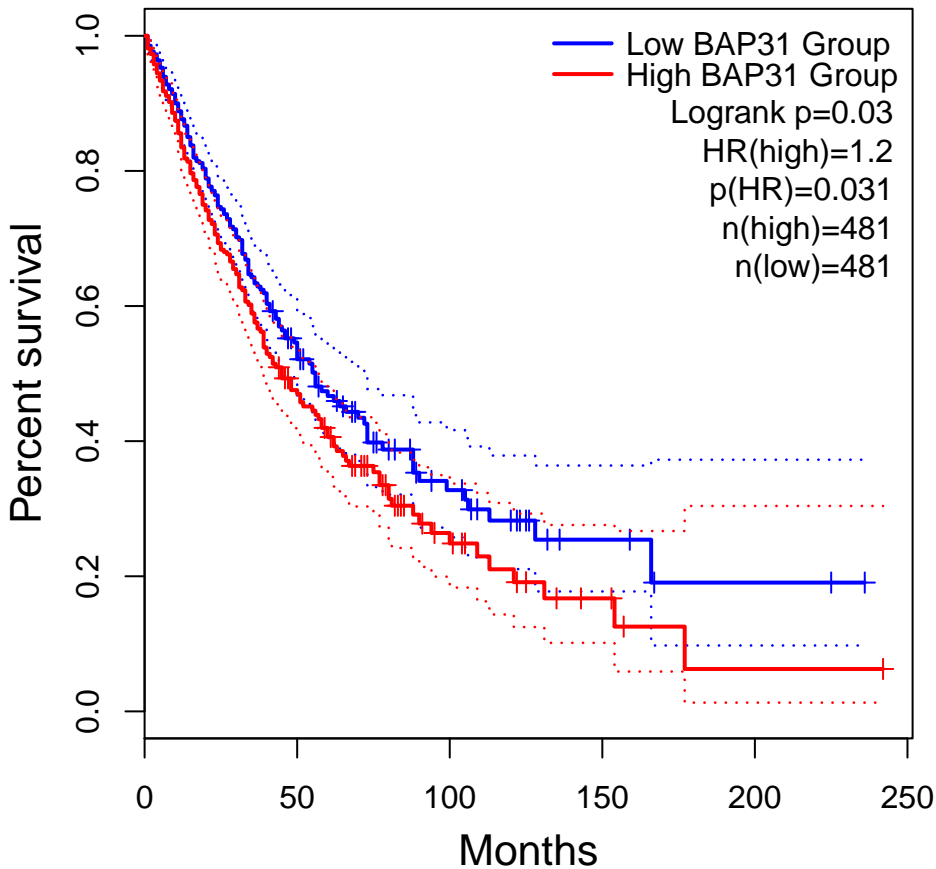

Supplement: Supplementary file 1 [file DataSheet_1.zip › raw data/Fig 1/BAP31_survival_ikifz(GEPIA2)lung cancer.pdf]

BCAP31 expression across TCGA cancer types

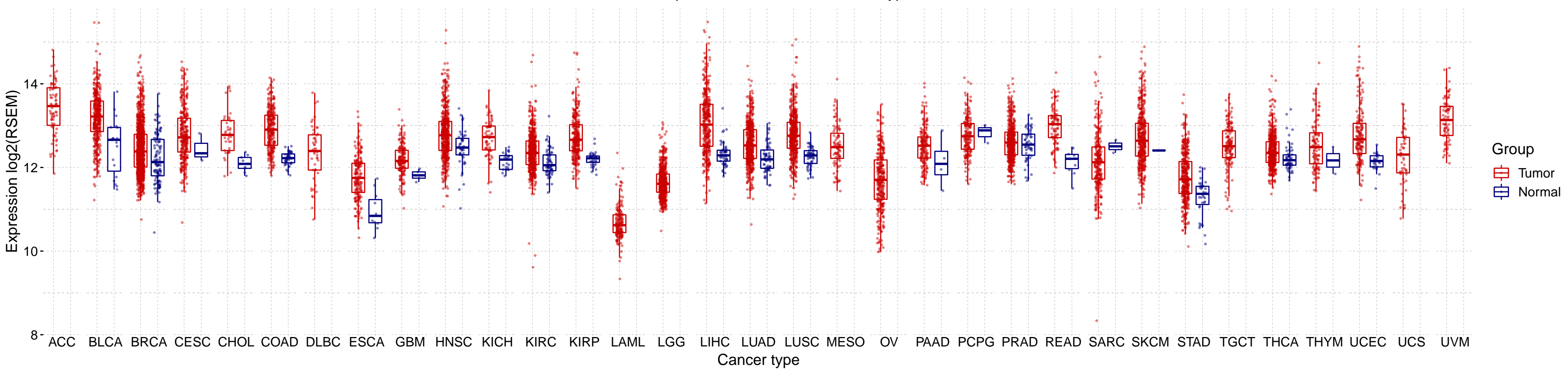

Supplement: Supplementary file 1 [file DataSheet_1.zip › raw data/Fig 1/GECA BAP31.pdf]

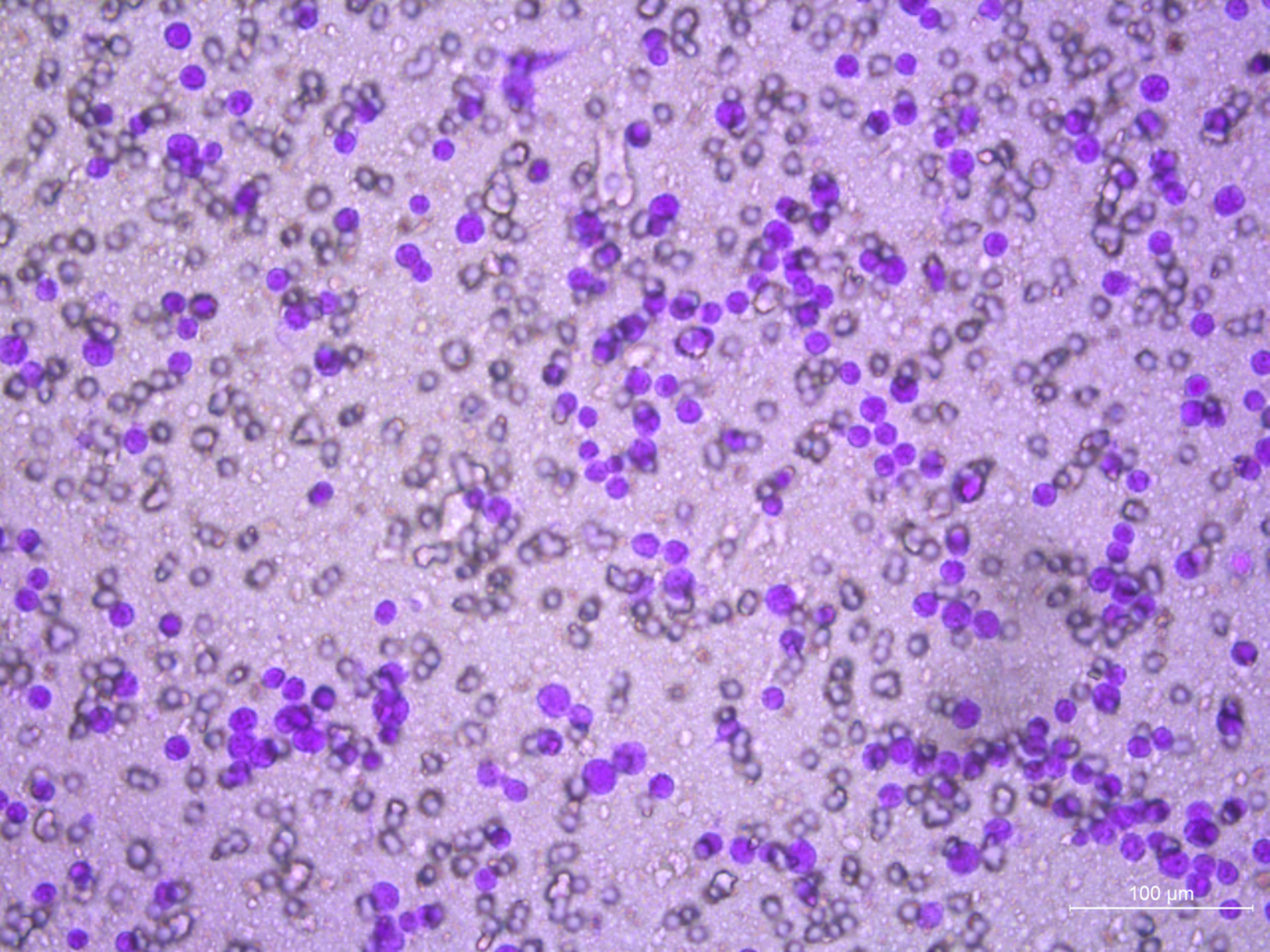

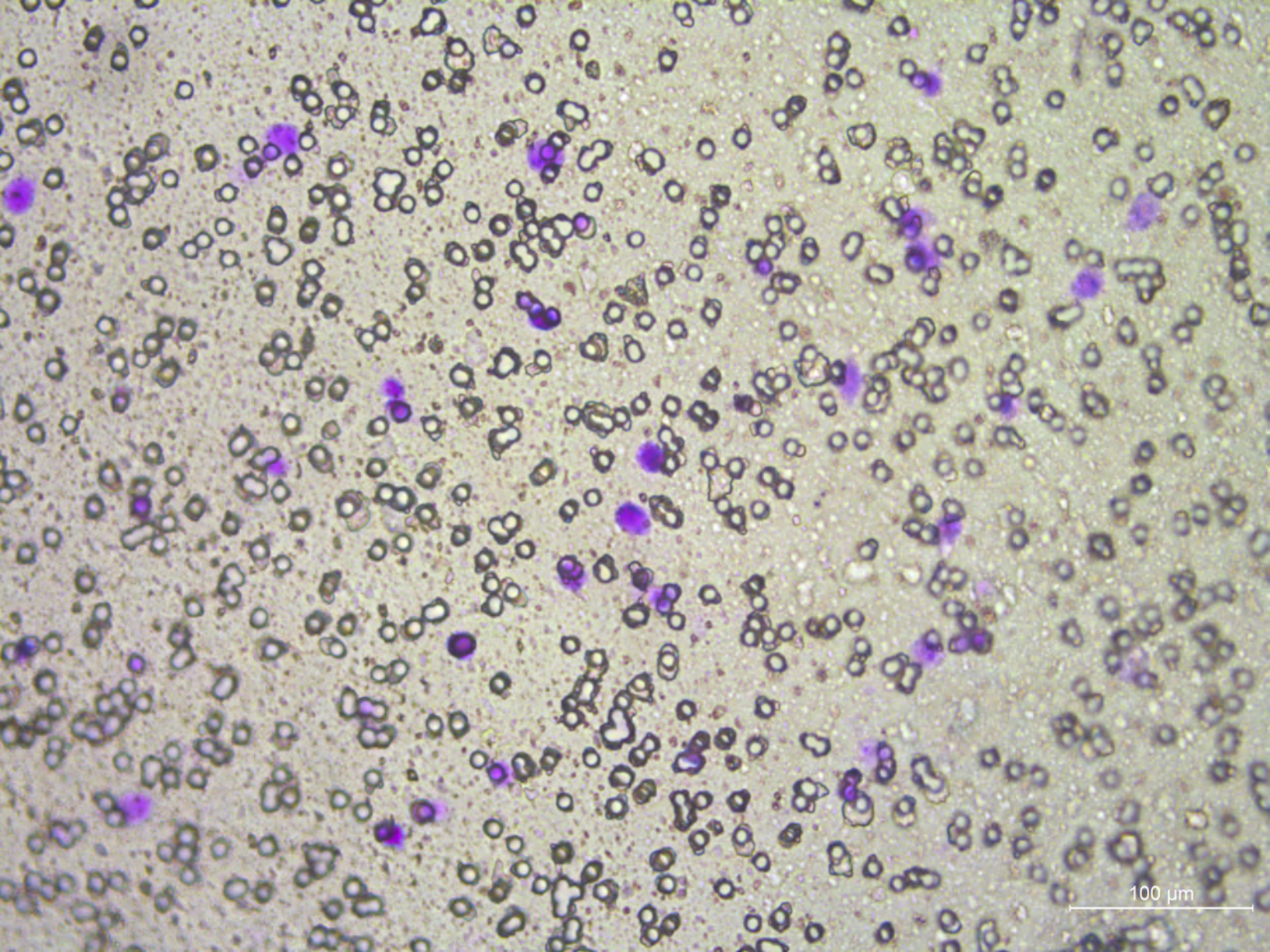

Supplement: Supplementary file 1 [file DataSheet_1.zip › raw data/Fig 4/transwell/1.pdf]

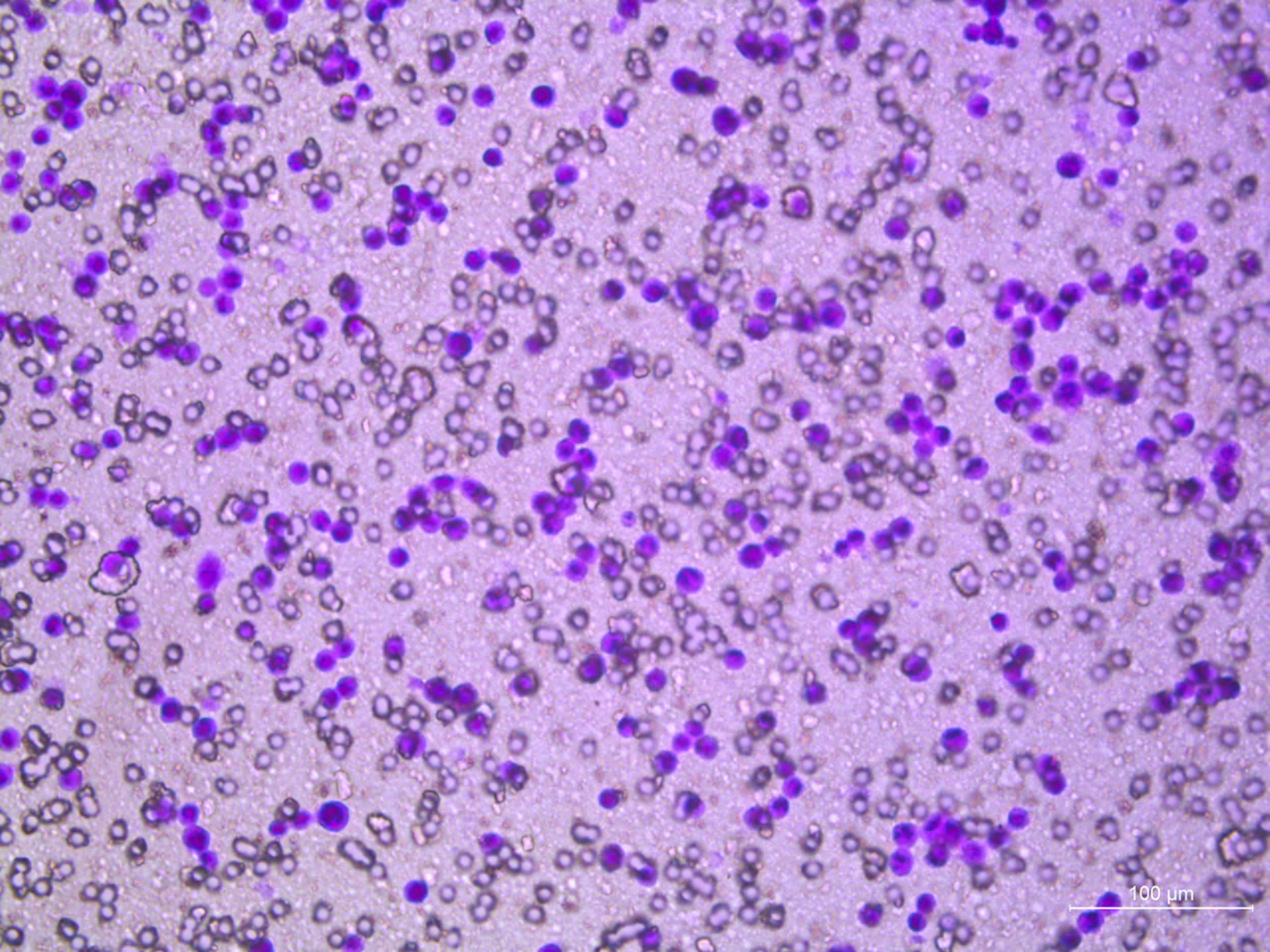

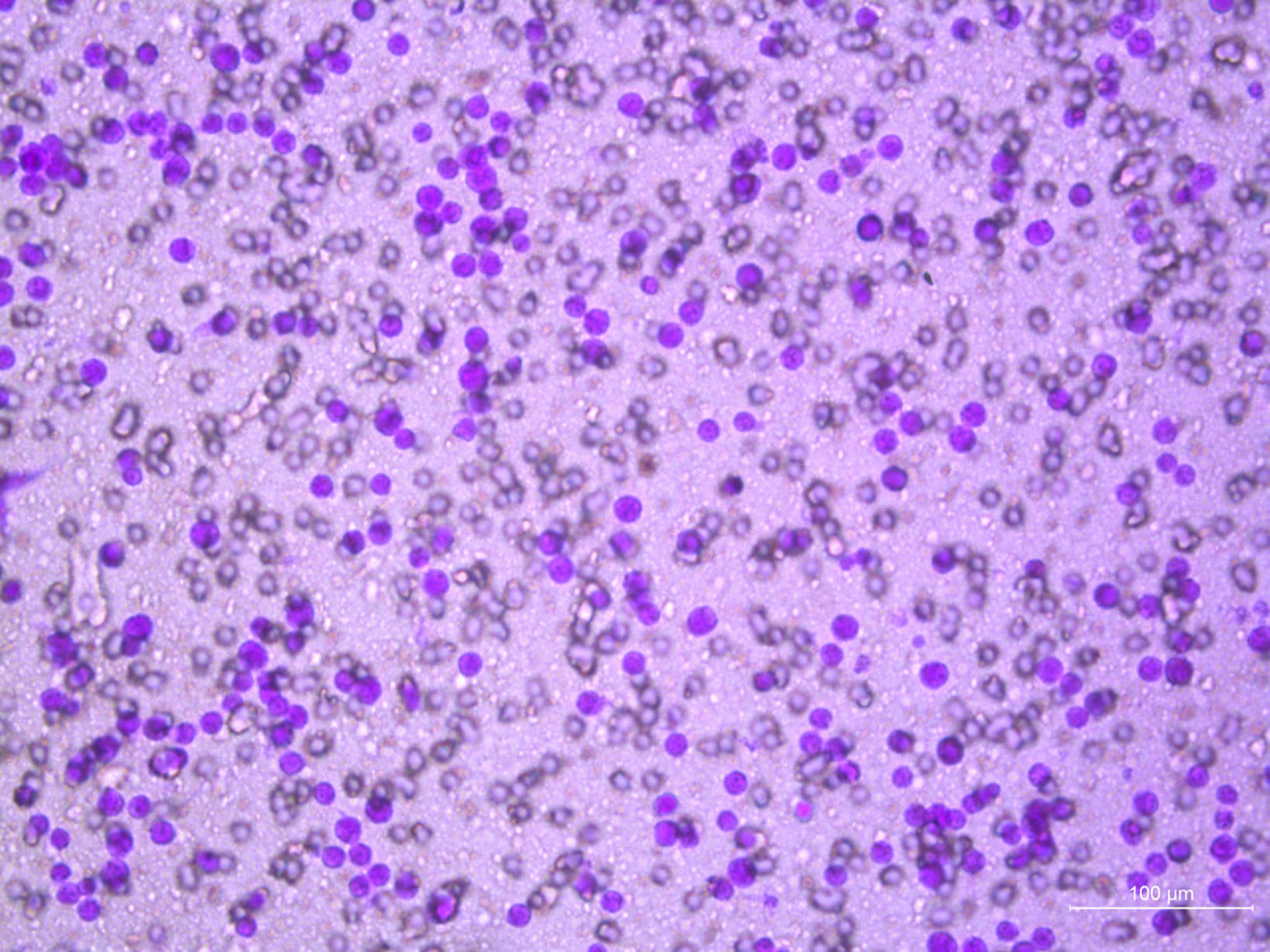

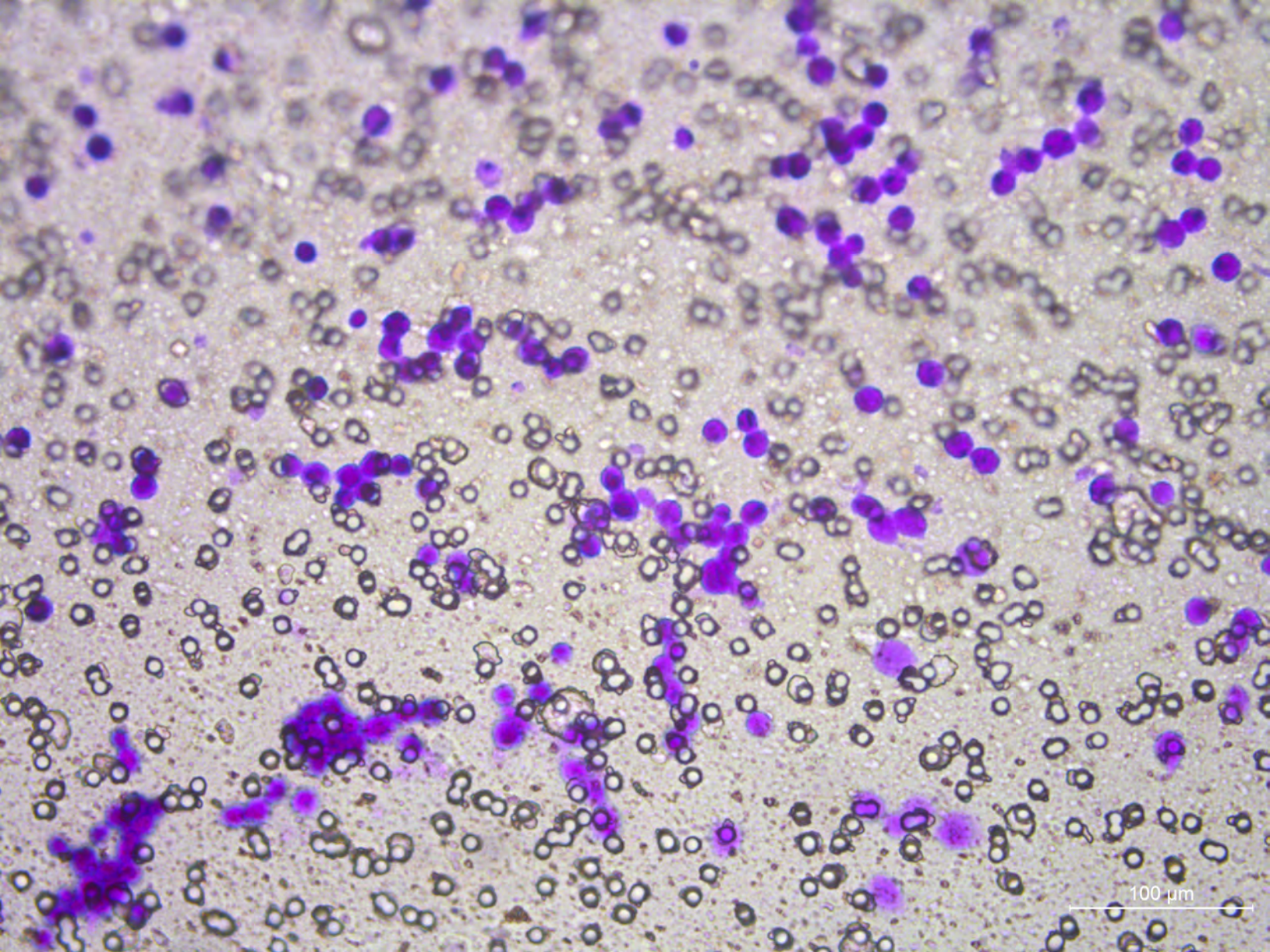

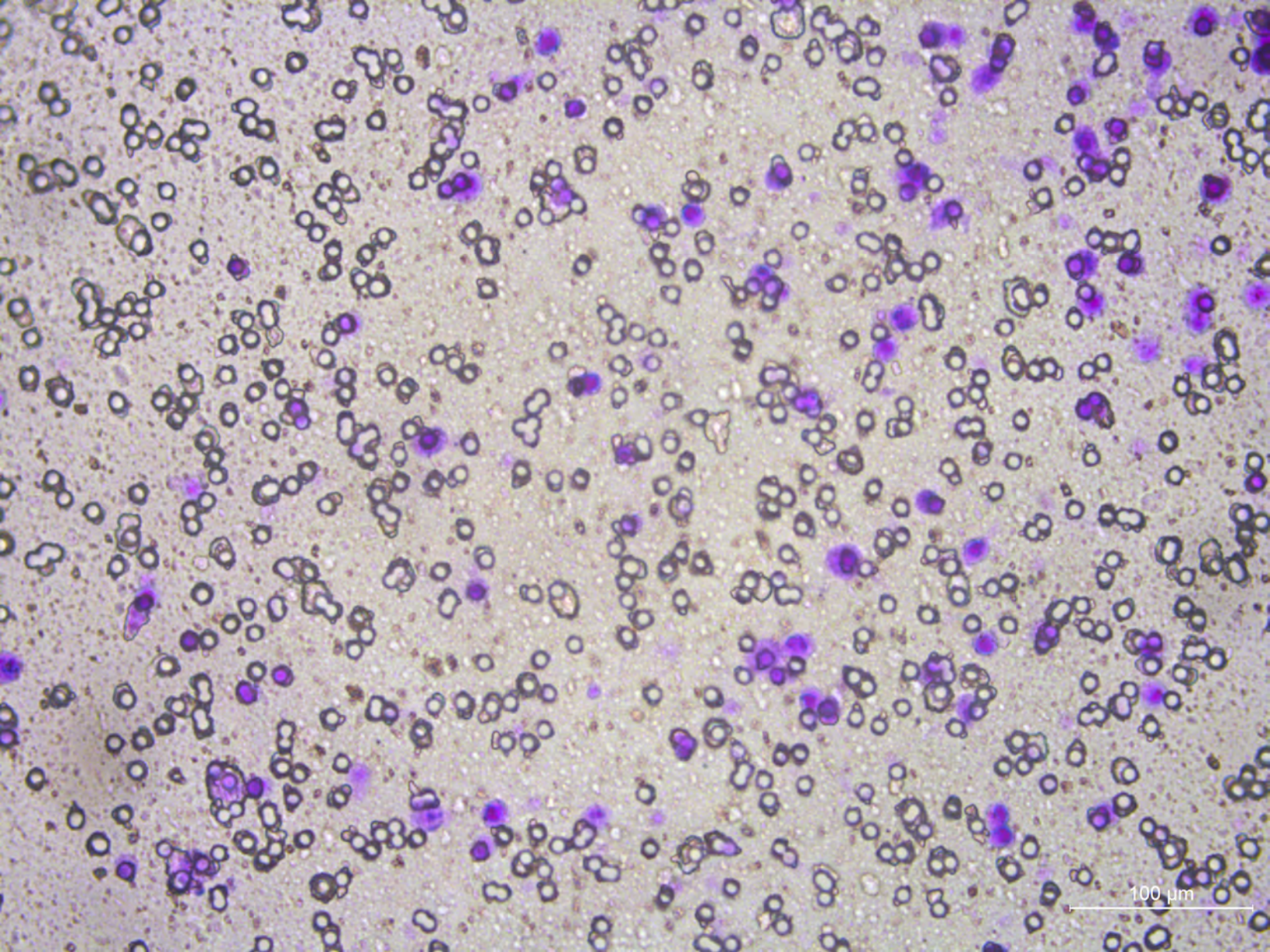

Supplement: Supplementary file 1 [file DataSheet_1.zip › raw data/Fig 5/transwell/2.pdf]

Fig6D

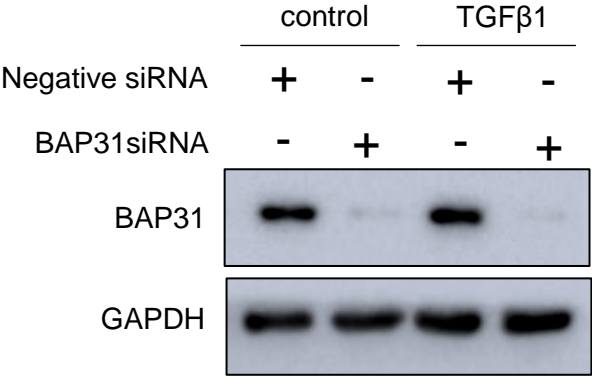

Fig6D BAP31

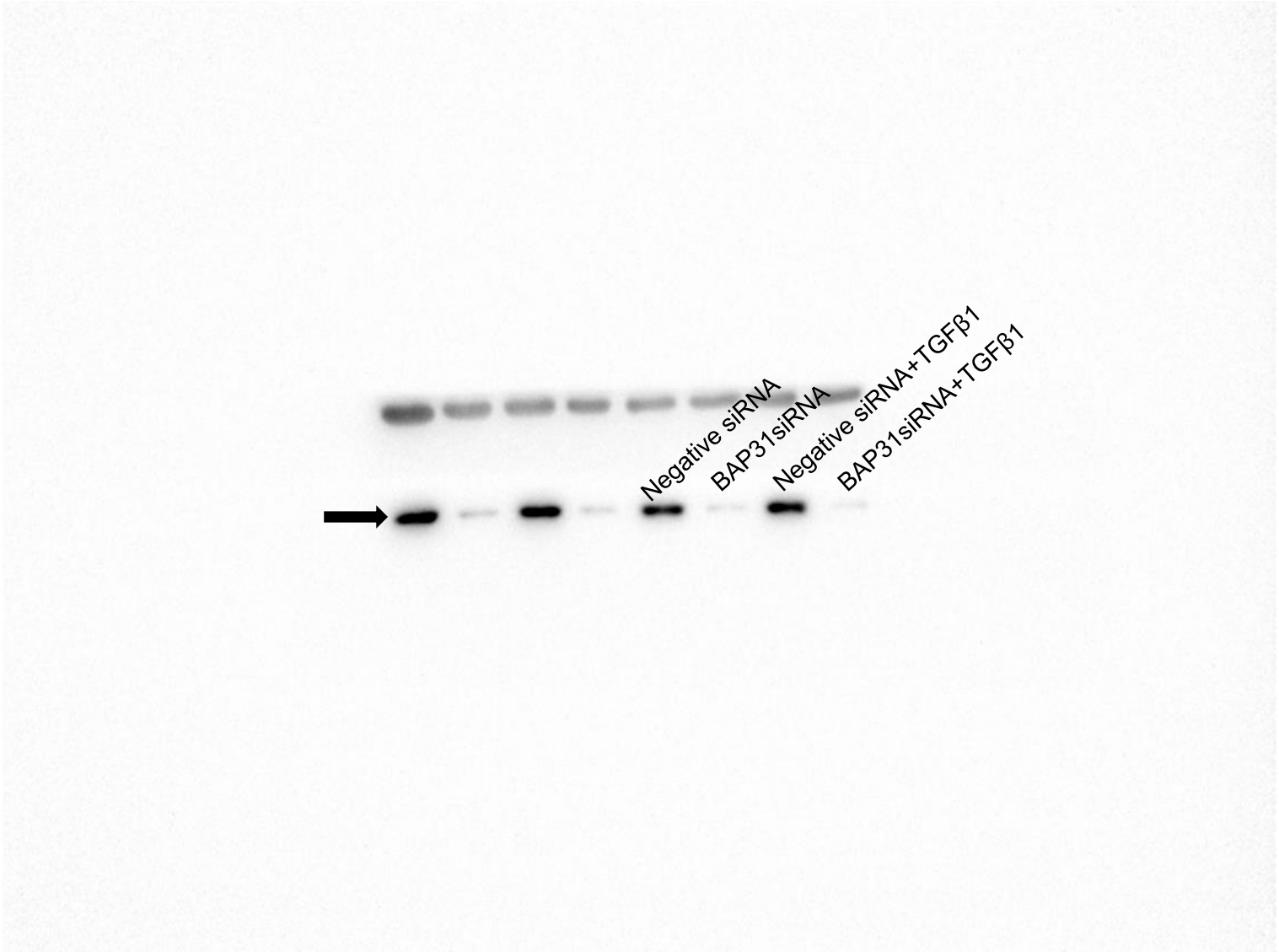

Fig6D gapdh

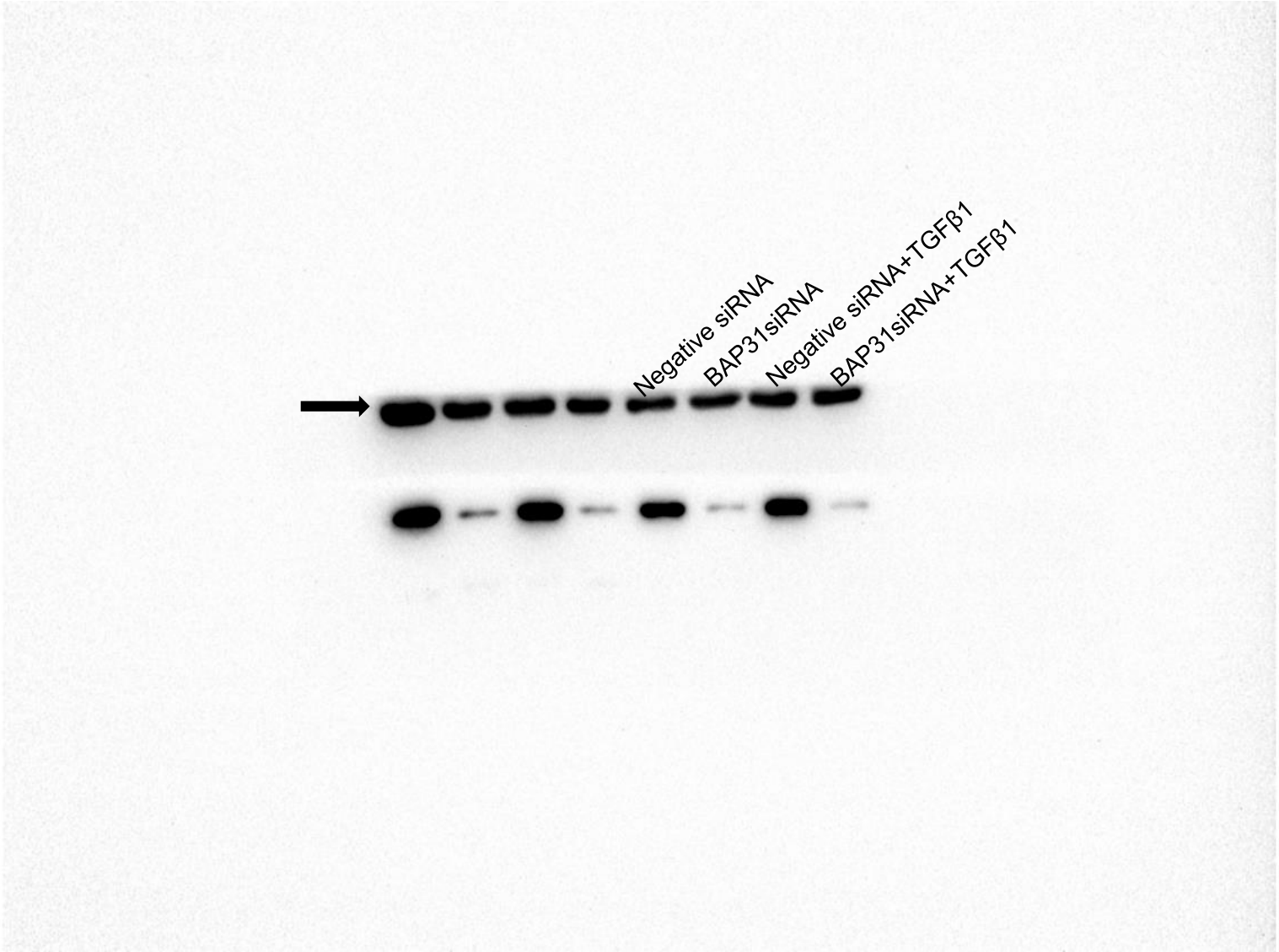

Supplement: Supplementary file 1 [file DataSheet_1.zip › raw data/Fig 6/Fig 6 western blot.pdf]

Fig8A

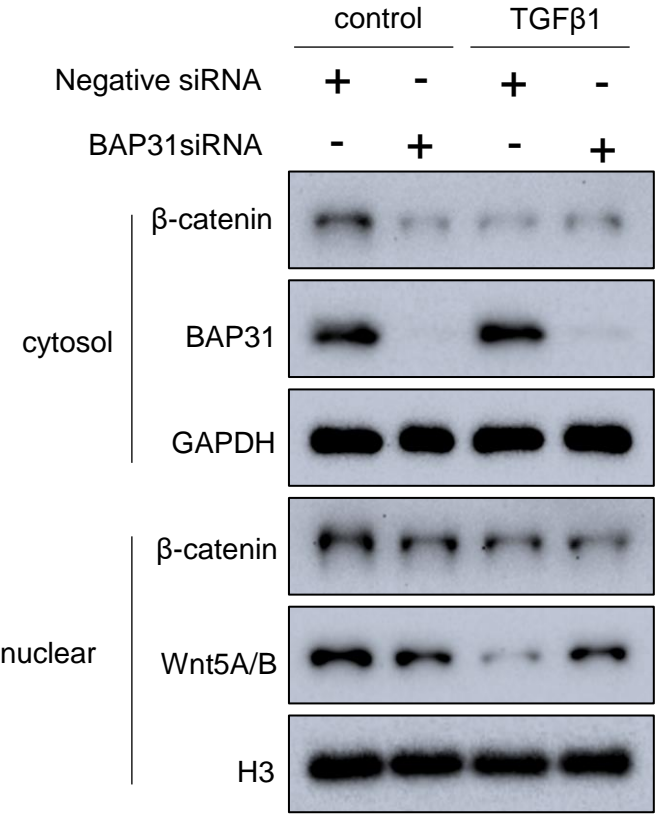

Fig8A  $\beta$ -catenin

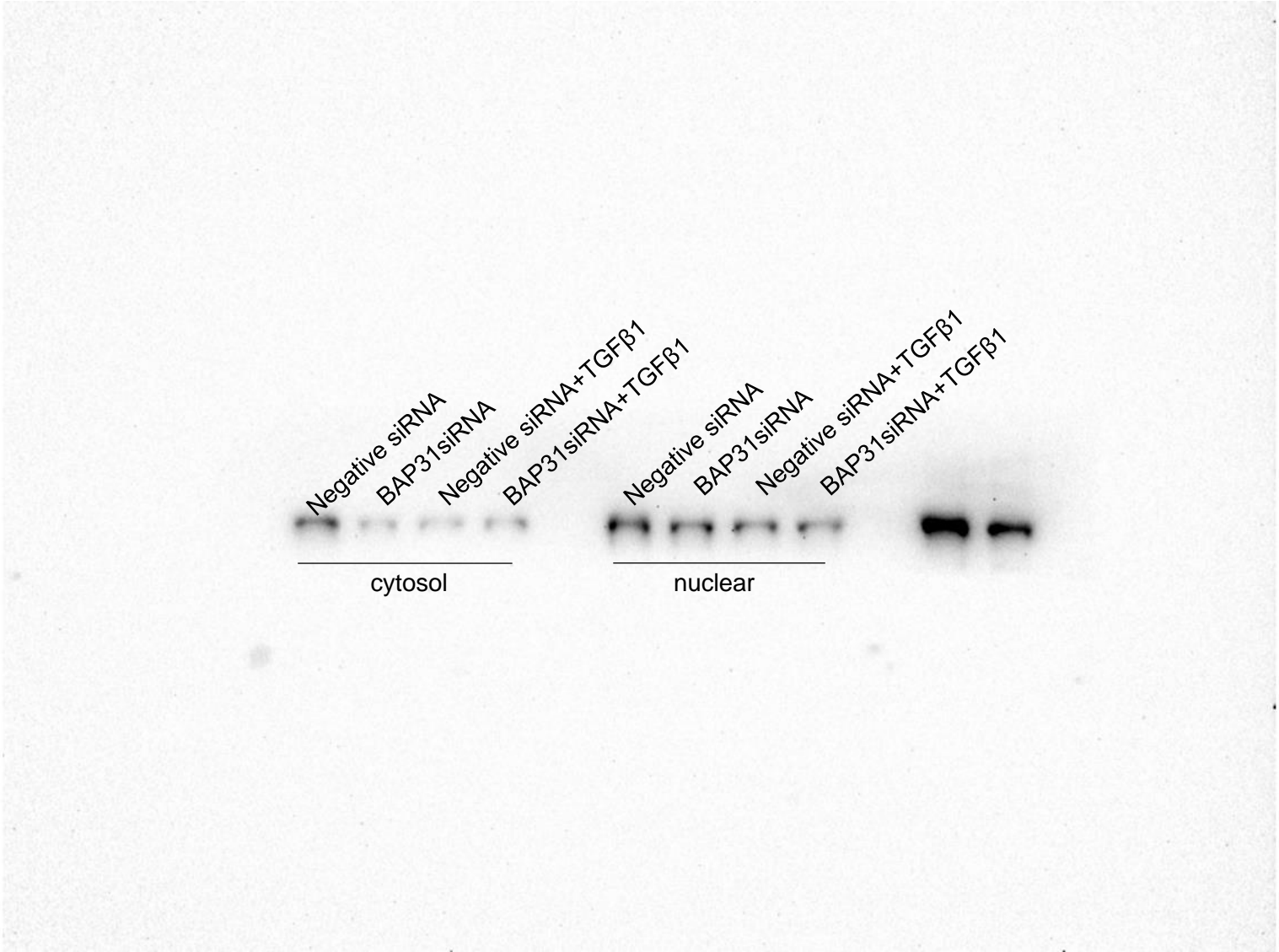

Fig8A BAP31

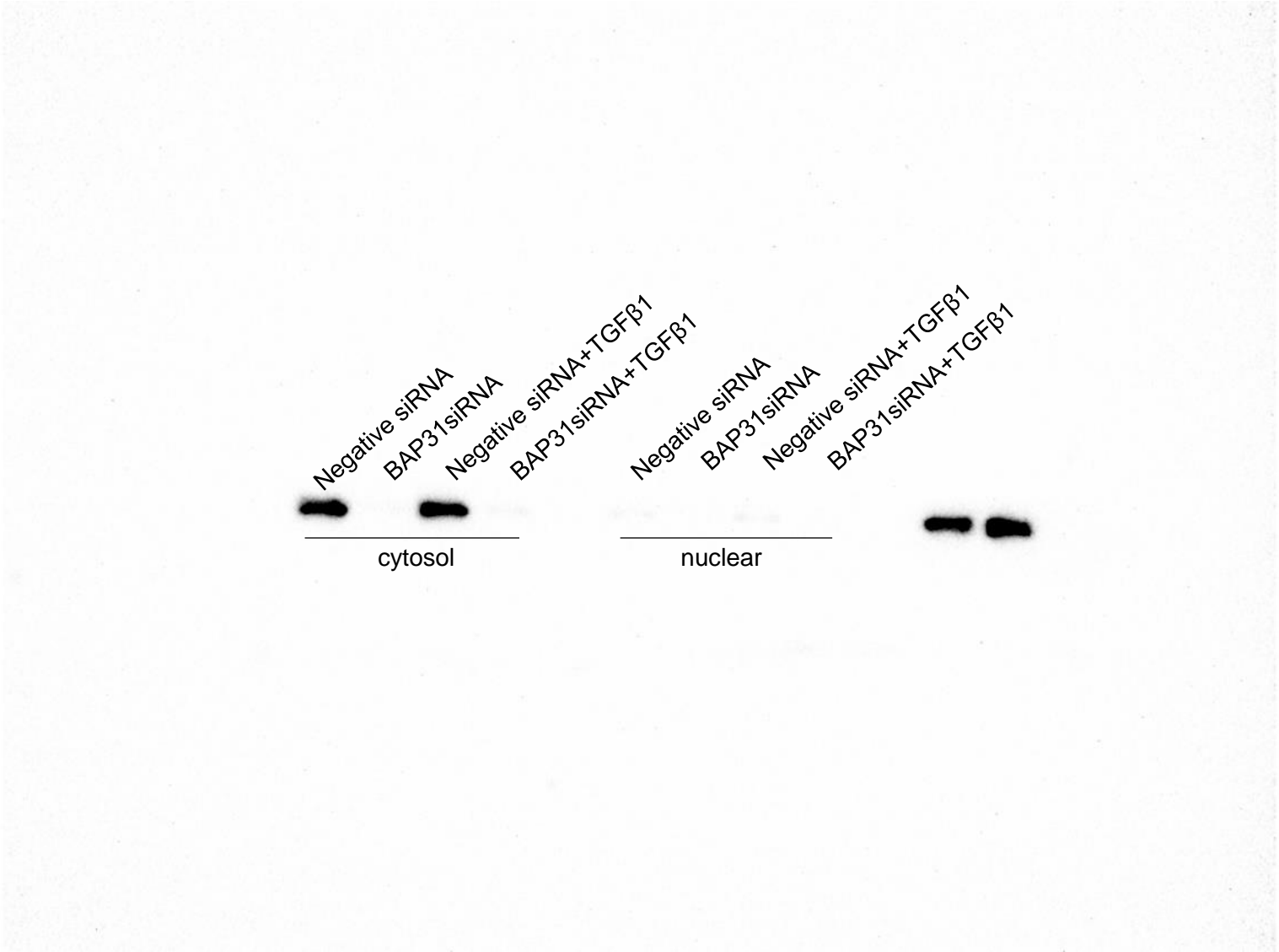

Fig8A wnt5ab

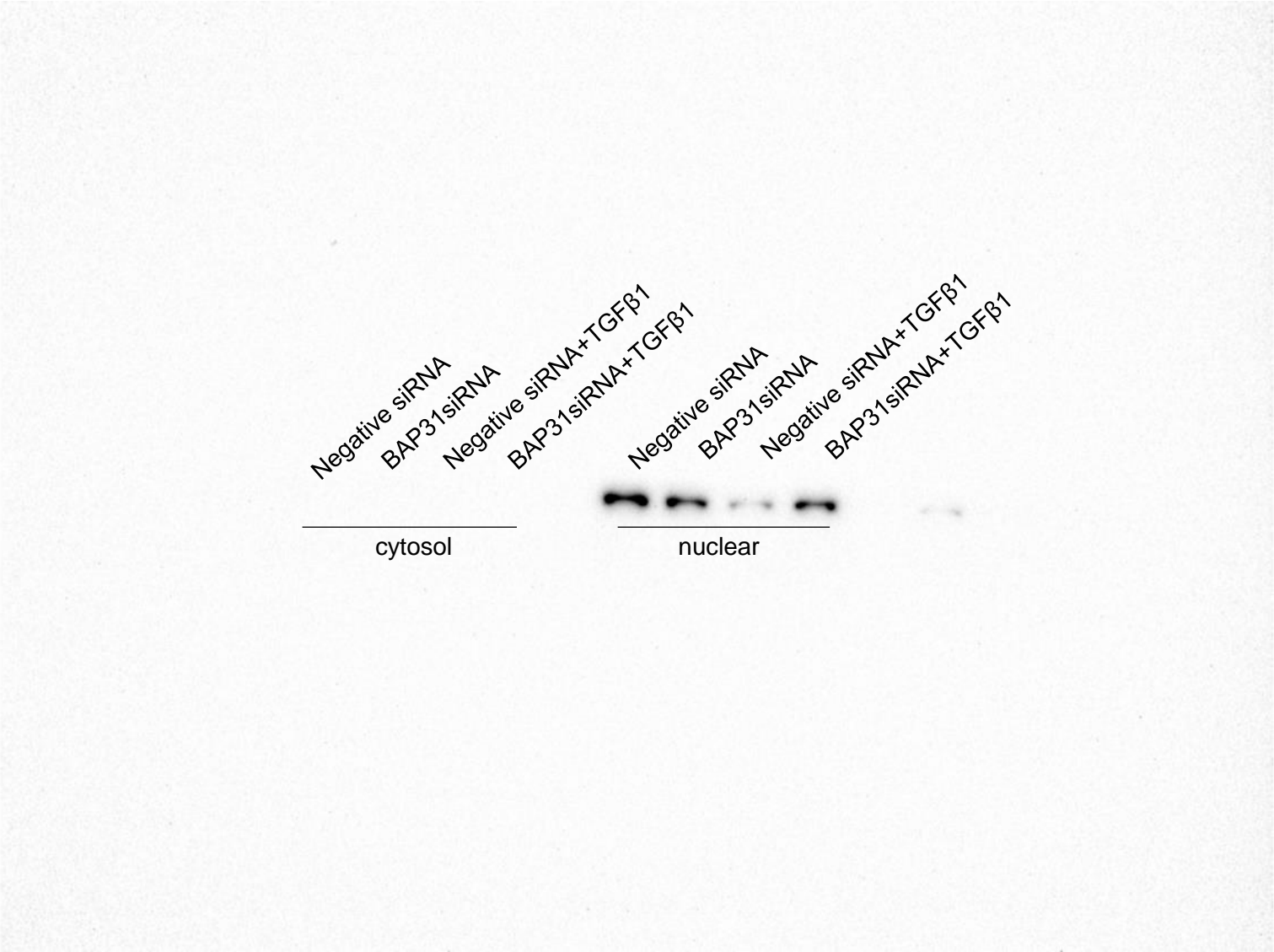

Fig8A gapdh

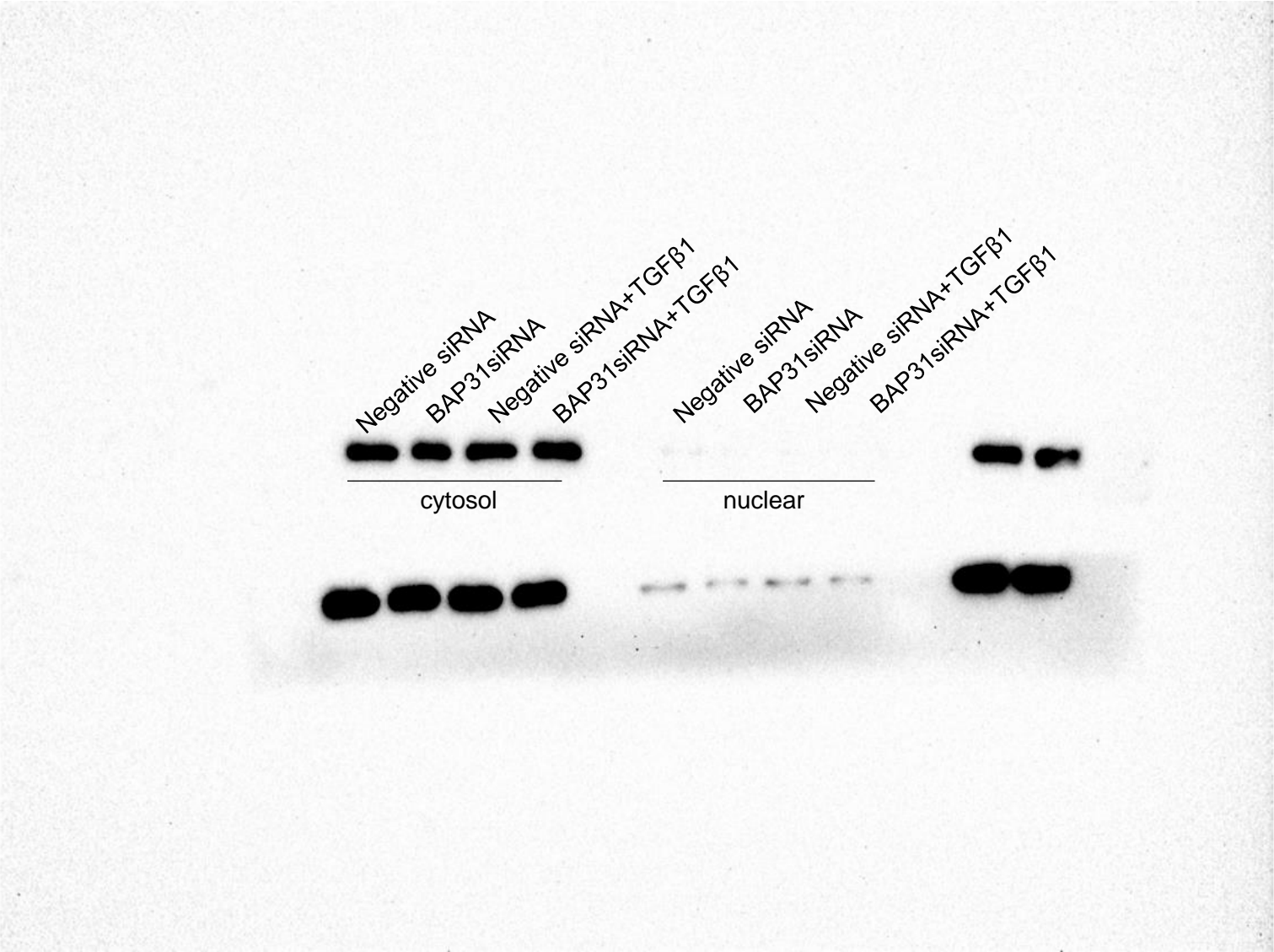

Fig8A h3

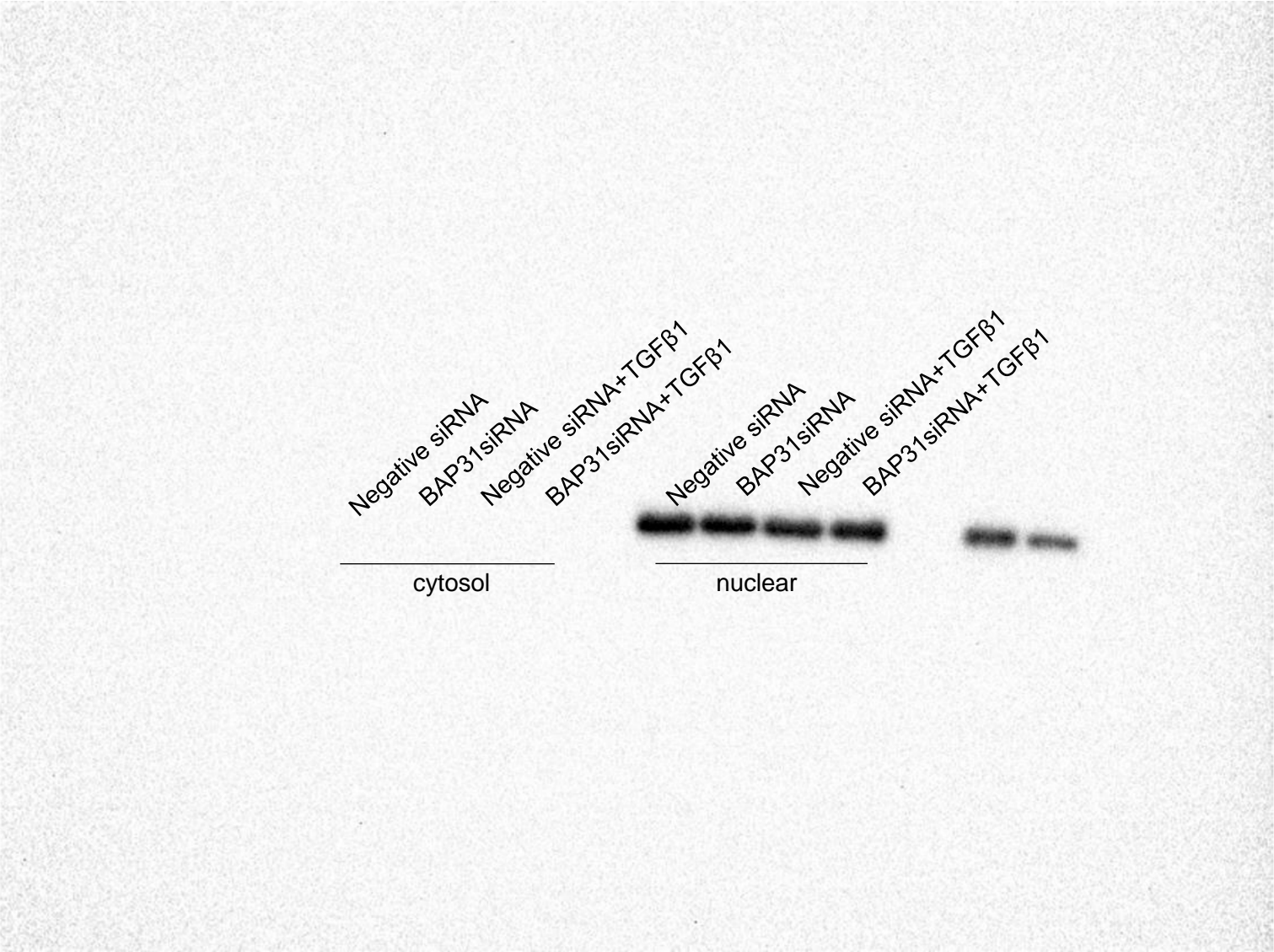

Supplement: Supplementary file 1 [file DataSheet_1.zip › raw data/Fig 8/Fig 8 western blot.pdf]

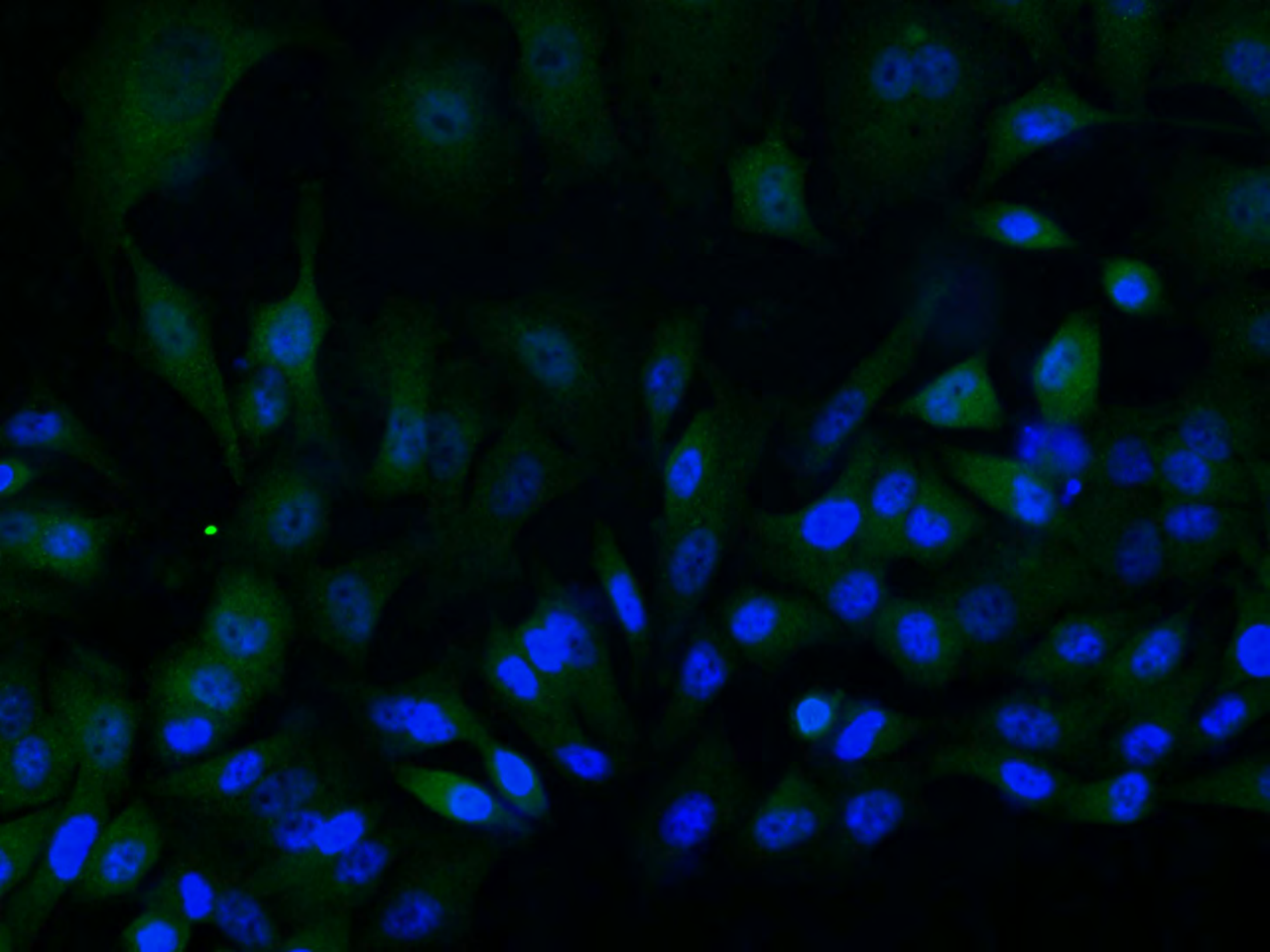

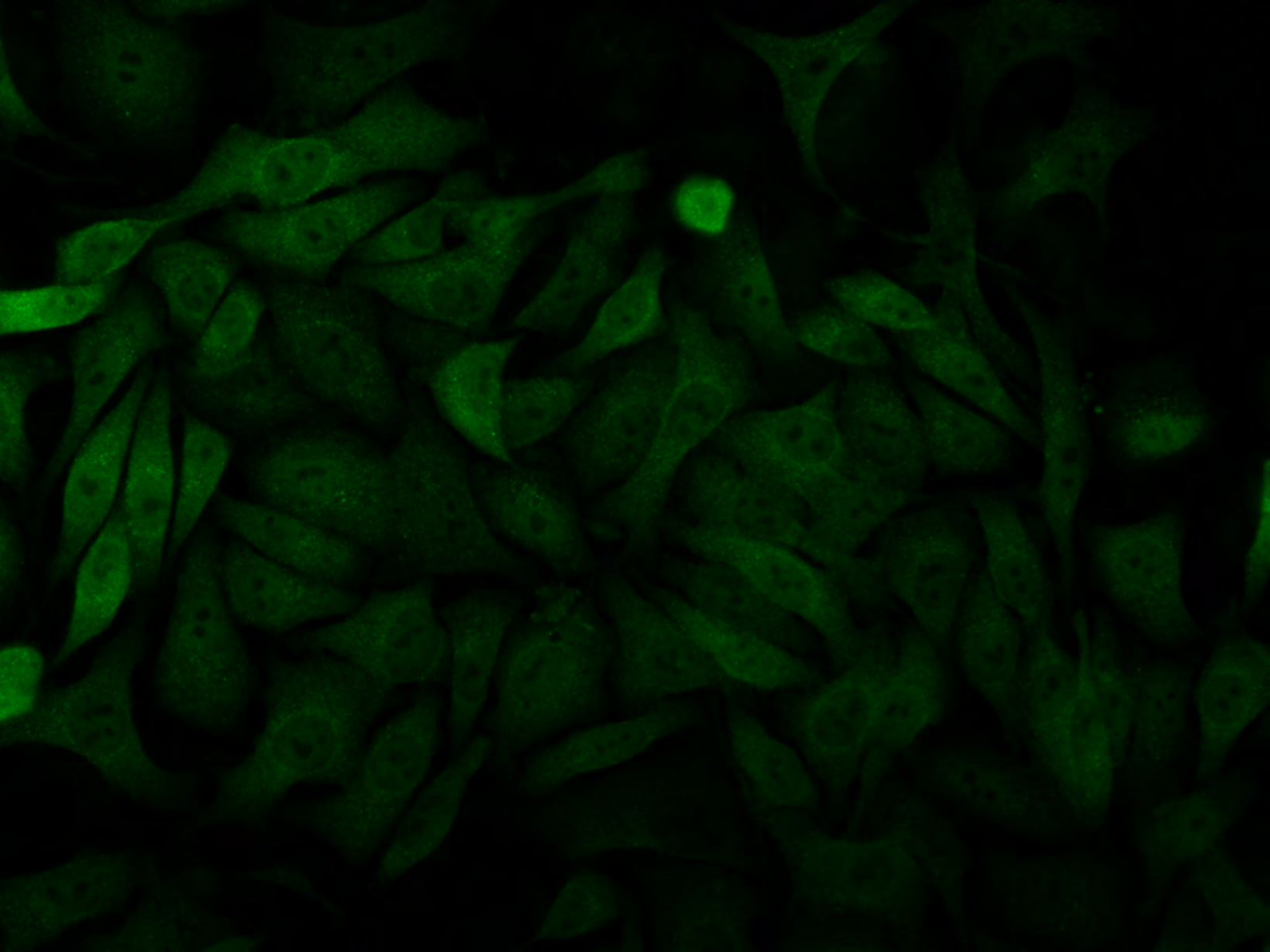



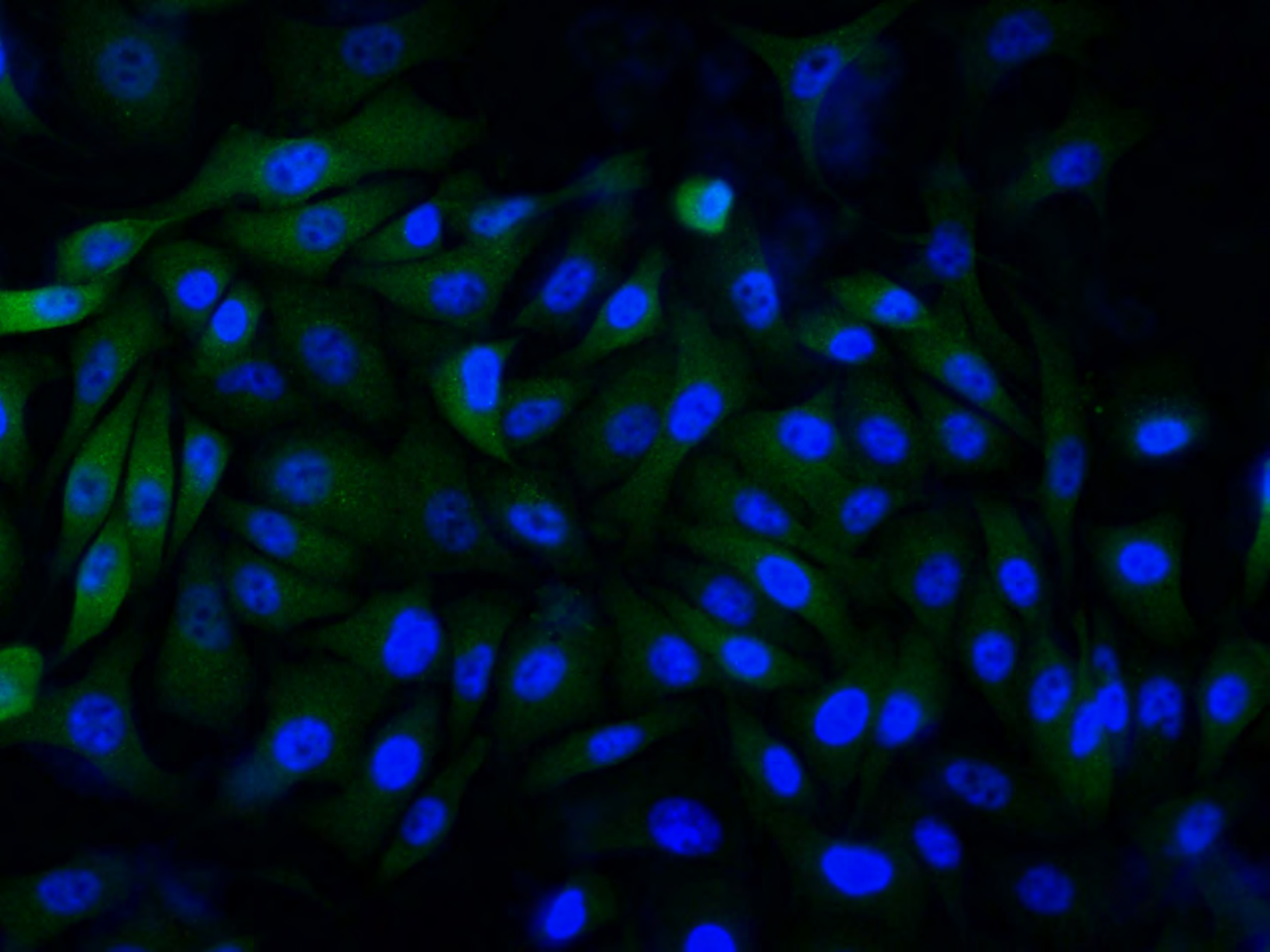





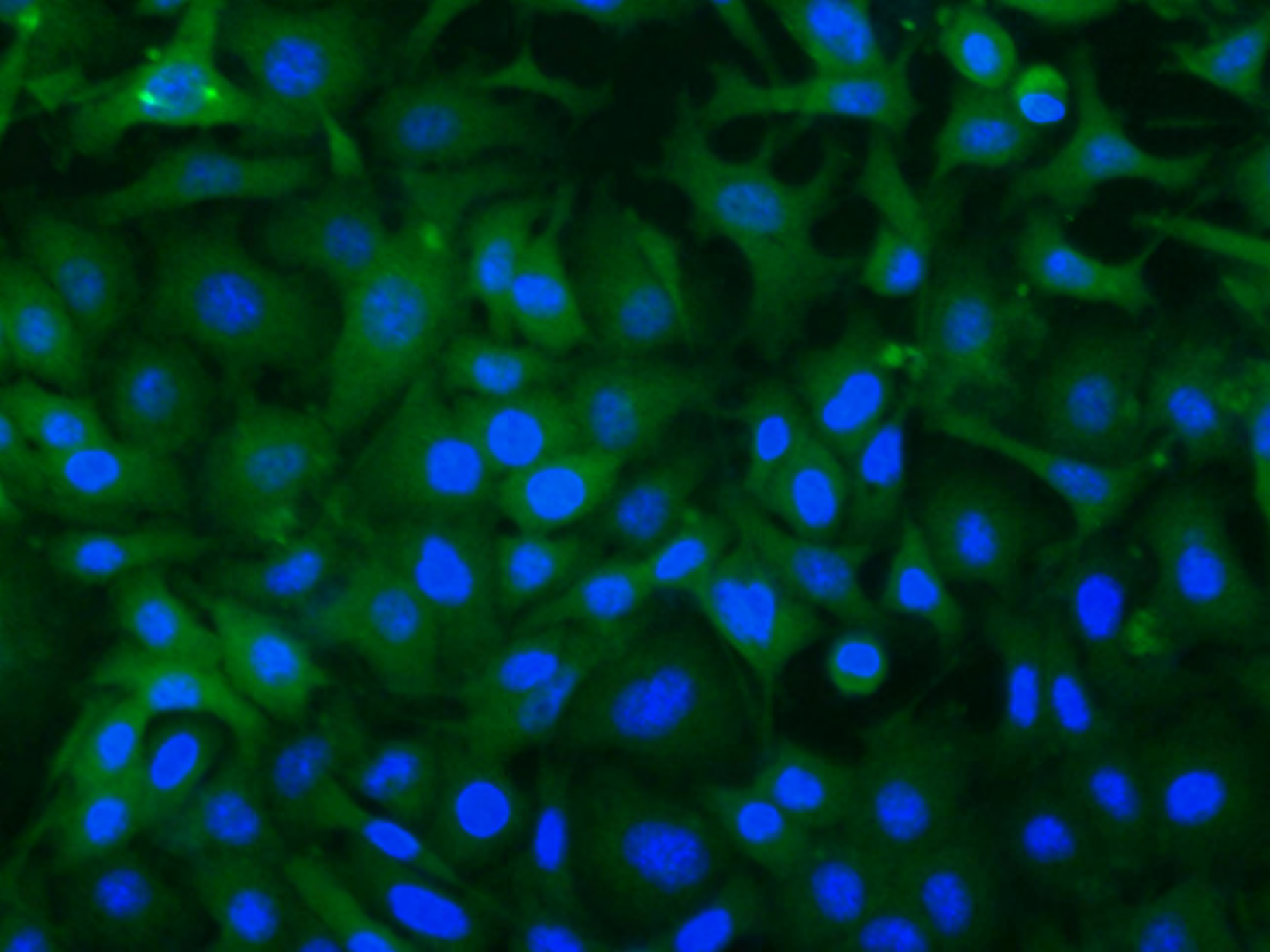

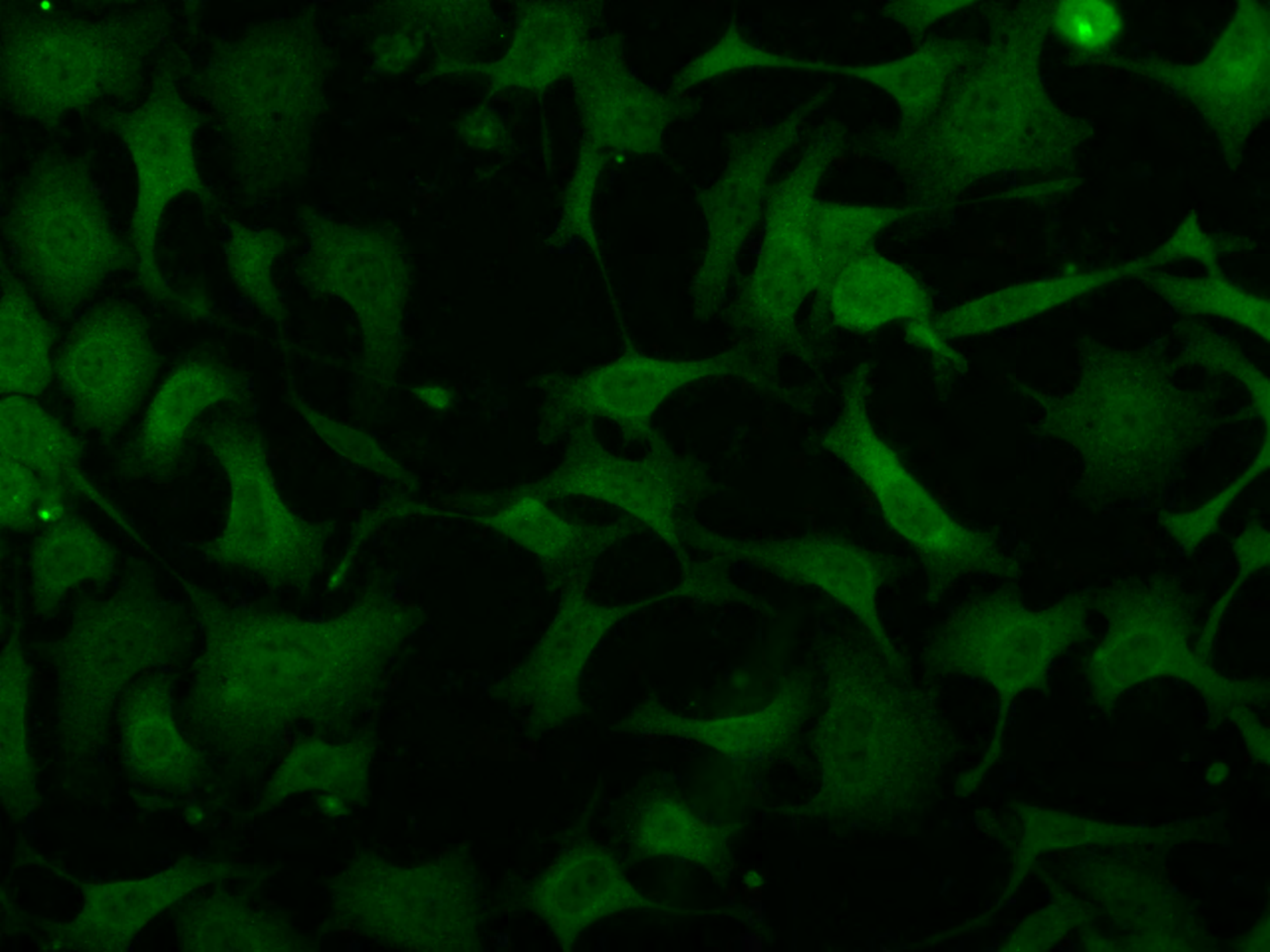



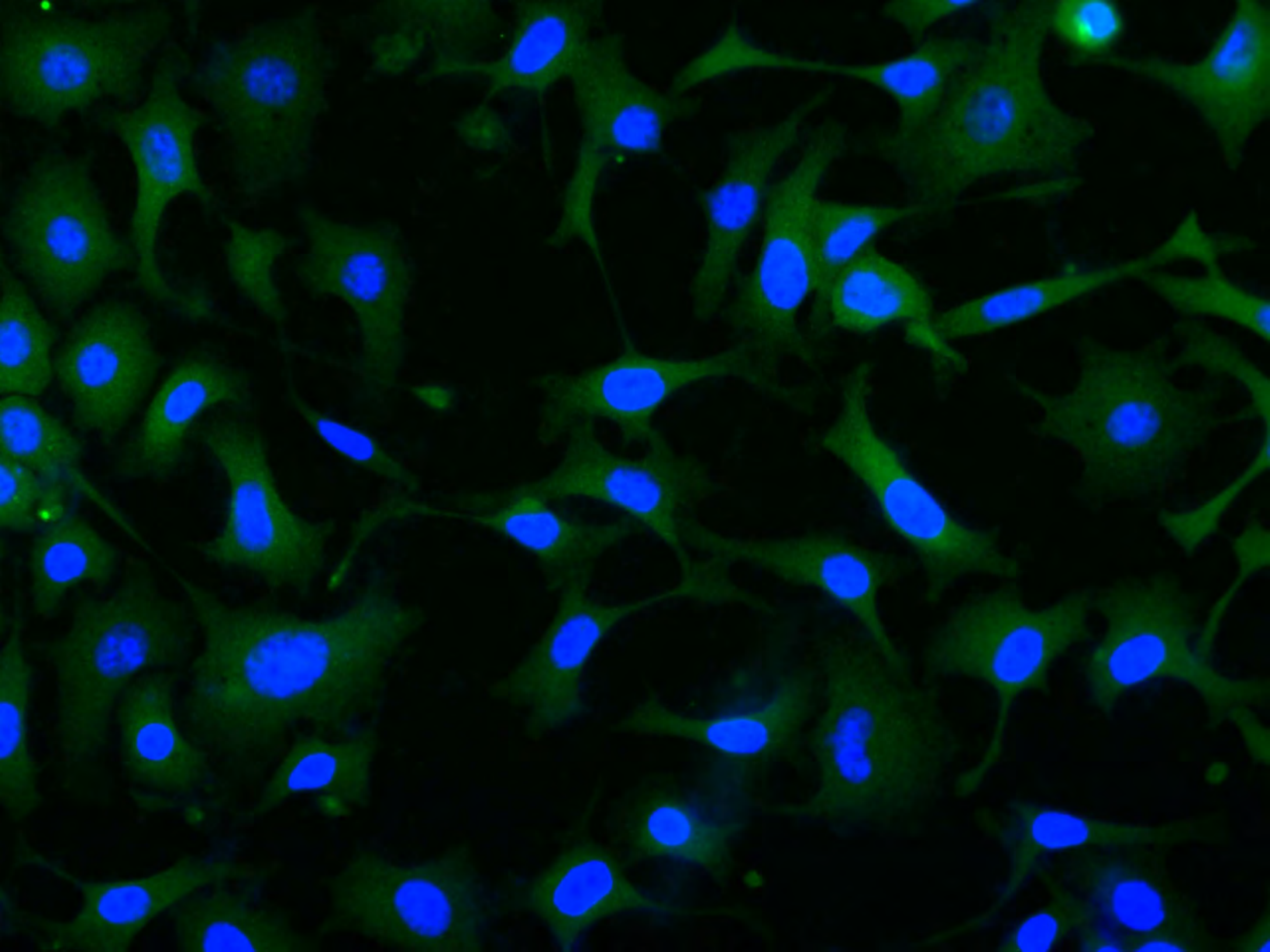

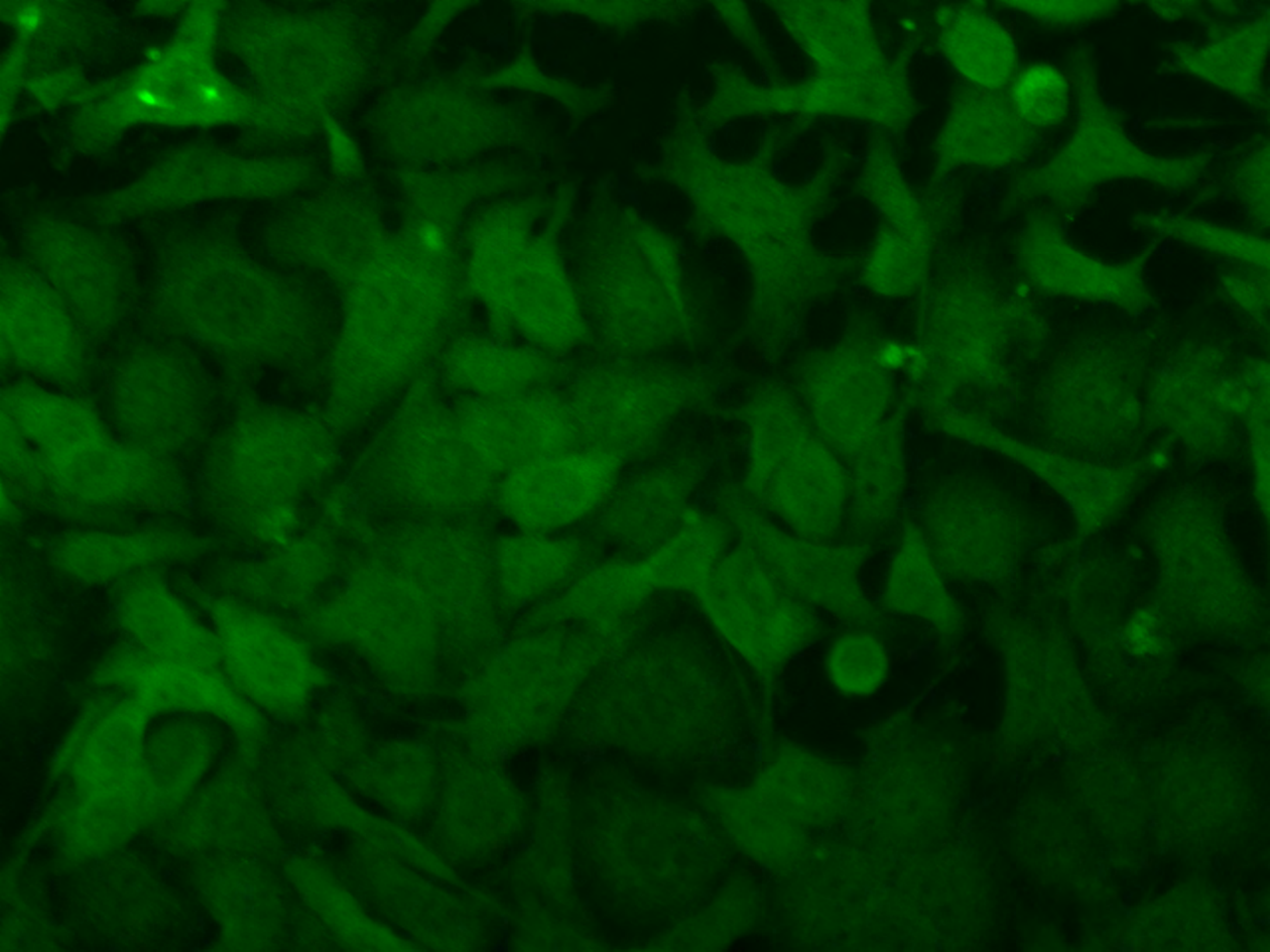

Supplement: Supplementary file 1 [file DataSheet_1.zip › raw data/Fig 8/β-catenin BAP31 immunofluorescence/BAP31/3.pdf]
