# Supplementary material for: BAP31 Regulates Wnt Signaling to Modulate Cell Migration in Lung Cancer
Source: Front Oncol. 2022 Mar 10;12:859195. doi: 10.3389/fonc.2022.859195 (PMC8960194; doi:10.3389/fonc.2022.859195)
Supplement: Supplementary Figure 1 — The survival of A549 lung cancer cells with transient transfection of pcDNA3.1(+)-BAP31-Flag plasmid. (A) The cell survival was examined by the CCK-8 assay. (B) Apoptosis was determined by annexin V-FITC/PI staining using flow cytometry. (C) Cell cycle distribution was examined by PI staining using flow cytometry. (D) Expression of BAP31 and cyclin B1 as evaluated by Western blot analysis. [file DataSheet_2.pdf]

BAP31 regulates Wnt signaling to modulate cell migration in lung cancer

Running Title: BAP31 in cancer cell migration

Tianye Li, Zhenzhen Hao, Zihan Tang, Chunting Li, Linglin Cheng, Tao Wang, Xiaojin Zhu, Yunhao He, Yongye Huang *, Bing Wang *

College of Life and Health Sciences, Northeastern University, Shenyang, 110169, China;

* To whom correspondence should be addressed: Tel: +86-24-83656116. Fax: +86-24-83656116. E-mail: huangyongye88@163.com (Yongye Huang), wangbing@mail.neu.edu.cn (Bing Wang).

Supplemental Figure

Figure S1. The survival of A549 lung cancer cells with transient transfection of pcDNA3.1(+)-BAP31-Flag plasmid. (A) The cell survival was examined by the CCK-8 assay. (B) Apoptosis was determined by annexin V-FITC/ PI staining using flow cytometry. (C) Cell cycle distribution was examined by PI staining using flow cytometry. (D) Expression of BAP31 and cyclin B1 as evaluated by Western blot analysis.

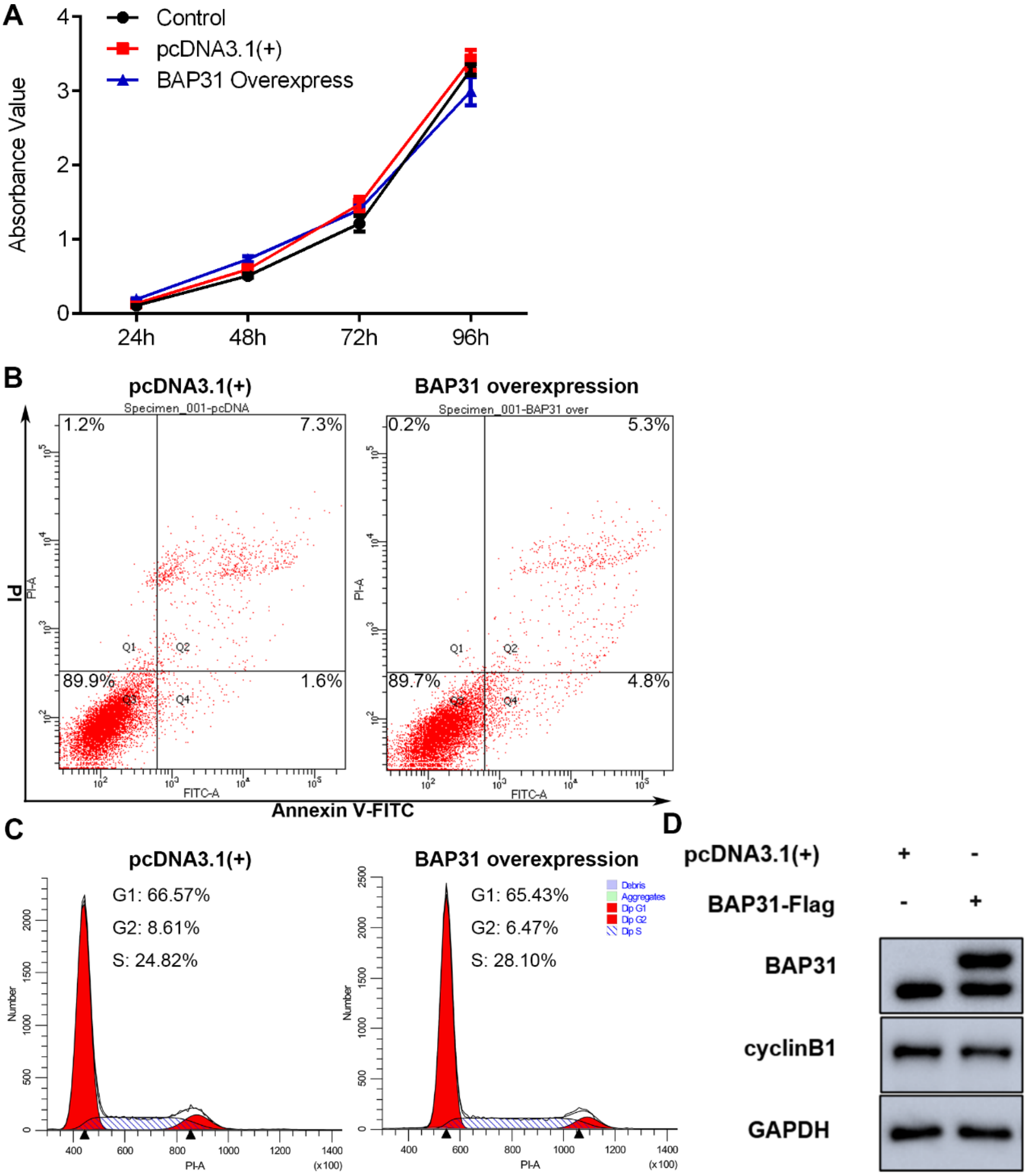

Figure S2. The survival of UC3 bladder cancer cells with BAP31 knockdown. (A) The survival of cells with BAP31 knockdown using shRNA was examined by the MTT assay. shBAP31-4: cells from colony 4# with BAP31 knockdown using shRNA. shBAP31-15: cells from colony 15# with BAP31 knockdown using shRNA. (B) The colony formation of UC3 cells with BAP31 knockdown using shRNA (mean ± SEM of duplicate experiments). (C) Expression of BAP31 in cells with BAP31 knockdown using shRNA as evaluated by Western blot analysis. (D, E) mRNA expression as examined by qPCR in cells with BAP31 knockdown using shRNA (mean ± SEM of duplicate experiments). (F, G) Apoptosis was determined by annexin V-FITC/ PI staining using flow cytometry in cells with BAP31 siRNA knockdown. Expression of BAP31 as evaluated by Western blot analysis. *$p<0.05$ versus control, **$p<0.01$ versus control and ***$p<0.001$ versus control.

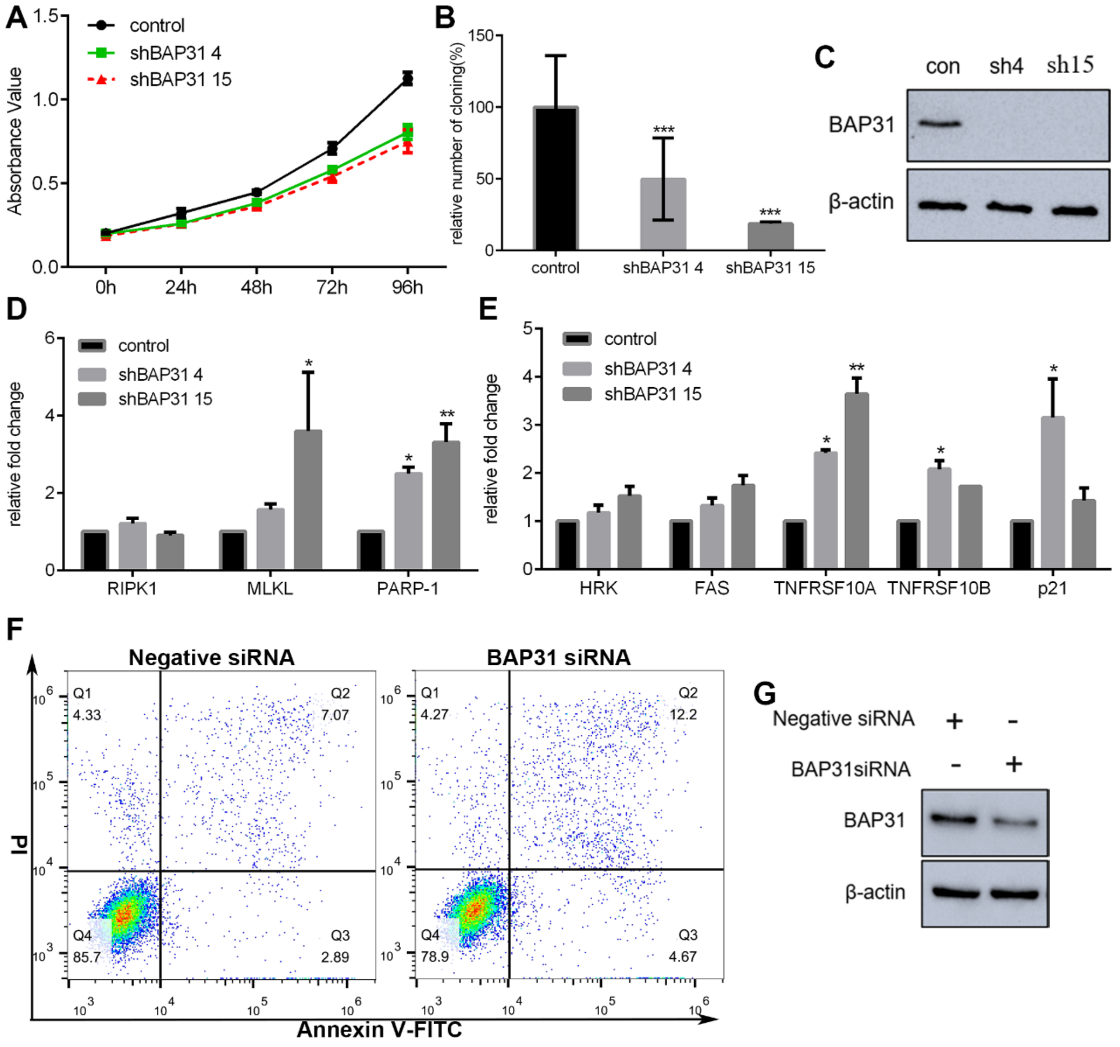

Figure S3. The survival of BAP31 knockdown A549 lung cancer cells treated with $MgCl_2$ or Z-VAD-FMK. (A) Apoptosis was determined by annexin V-FITC/ PI staining using flow cytometry. (B) The cell survival was examined by the CCK-8 assay. ***$p<0.001$ versus negative siRNA in control group.

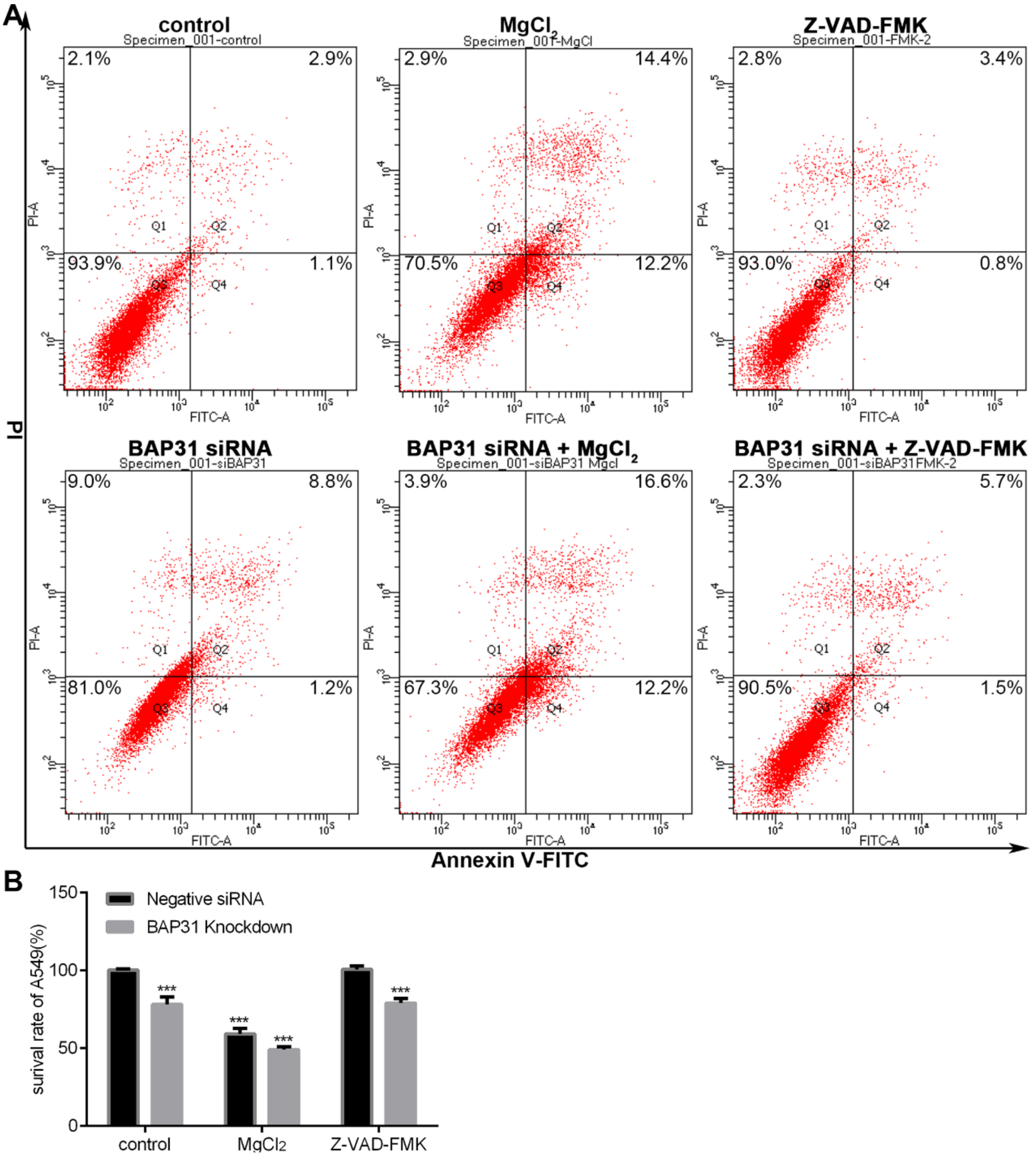

Supplementary Table S1. List of primary antibodies applied in this study

Antibody	brand	dilution	application
Bax	Invitrogen	1:500	Western blotting
Bak	Bimake	1:1000	Western blotting
Bcl2	Bimake	1:1000	Western blotting
Bid	Signalway Antibody	1:1000	Western blotting
MLKL	Bimake	1:1000	Western blotting
CD63	CST	1:1000	Western blotting
Sox2	CST	1:1000	Western blotting
TIF1β	CST	1:1000	Western blotting
Nanog	Invitrogen	1:1000	Western blotting
cyclinB1	Bimake	1:1000	Western blotting
CDK1	proteintech	1:1000	Western blotting
p21	Bimake	1:1000	Western blotting
Bip	proteintech	1:2000	Western blotting
CHOP	proteintech	1:1000	Western blotting
p62	proteintech	1:2000	Western blotting
Beclin1	proteintech	1:2000	Western blotting
VDAC	CST	1:1000	Western blotting
LC3	proteintech	1:1000	Western blotting
E-cadherin	CST	1:1000	Western blotting
Connexin43	Abcam	1:1000	Western blotting
N-cadherin	CST	1:1000	Western blotting
Vimentin	CST	1:1000	Western blotting
α-SMA	proteintech	1:1000	Western blotting
Zeb1	CST	1:1000	Western blotting
β-catenin	CST	1:1000	Western blotting
		1:100	Immunofluorescence
MMP2	proteintech	1:1000	Western blotting

ZO-1	proteintech	1:1000	Western blotting
Claudin1	CST	1:1000	Western blotting
Fibronectin	Abcam	1:1000	Western blotting
MMP9	proteintech	1:1000	Western blotting
HSP90	Bimake	1:1000	Western blotting
Wnt5A/B	CST	1:1000	Western blotting
p-mTOR	CST	1:1000	Western blotting
mTOR	CST	1:1000	Western blotting
p-AKT(473)	CST	1:1000	Western blotting
p-AKT(308)	CST	1:1000	Western blotting
AKT	proteintech	1:2000	Western blotting
p-p38	CST	1:1000	Western blotting
p38	CST	1:1000	Western blotting
Slug	CST	1:1000	Western blotting
BAP31	Abcam	1:1000	Western blotting
		1:100	Immunofluorescence
H3	CST	1:1000	Western blotting
β-actin	CST	1:2000	Western blotting
GAPDH	CST	1:5000	Western blotting
