# Supplementary material for: BAP31 Regulates Wnt Signaling to Modulate Cell Migration in Lung Cancer
Source: Front Oncol. 2022 Mar 10;12:859195. doi: 10.3389/fonc.2022.859195 (PMC8960194; doi:10.3389/fonc.2022.859195)

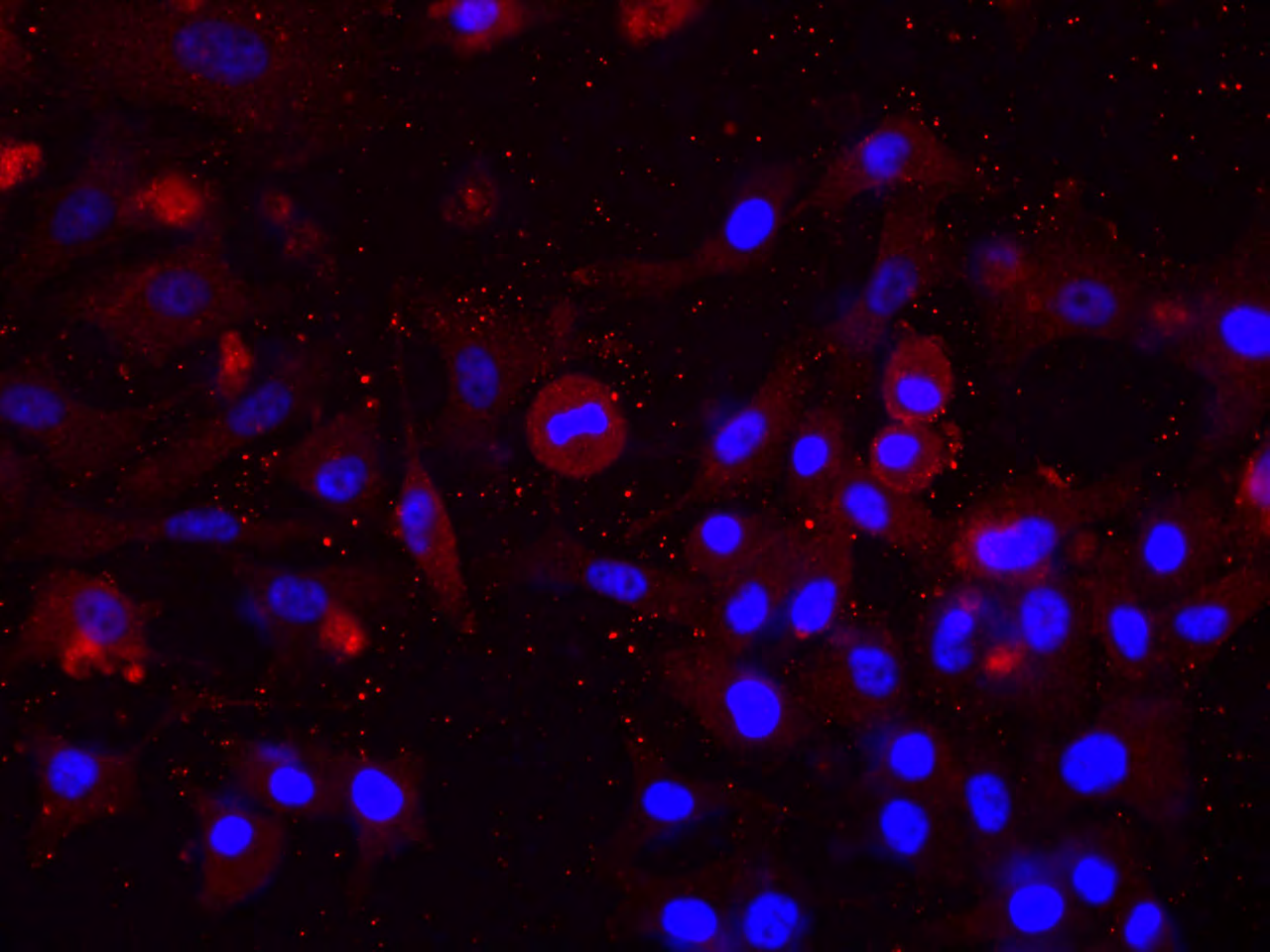

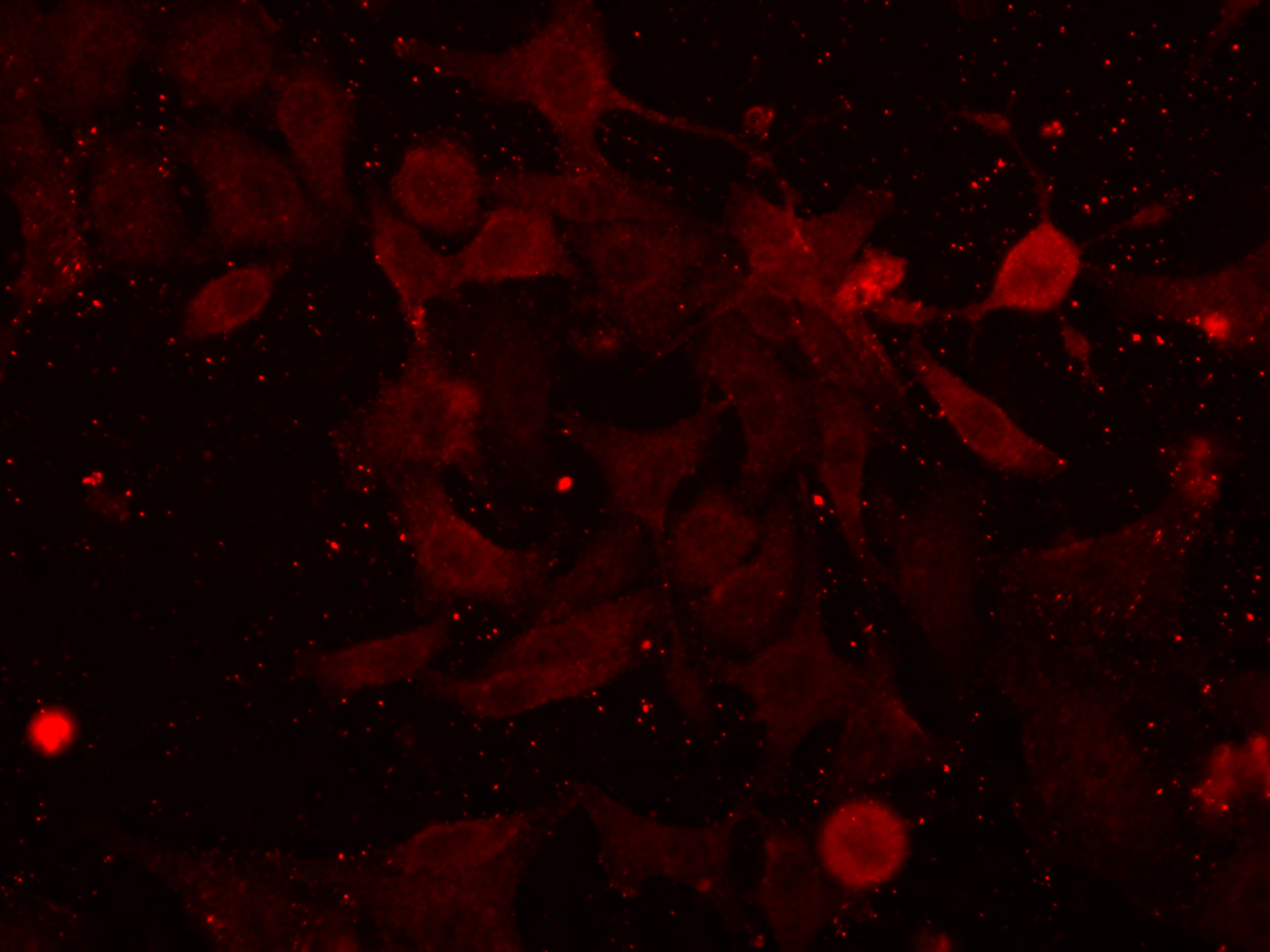

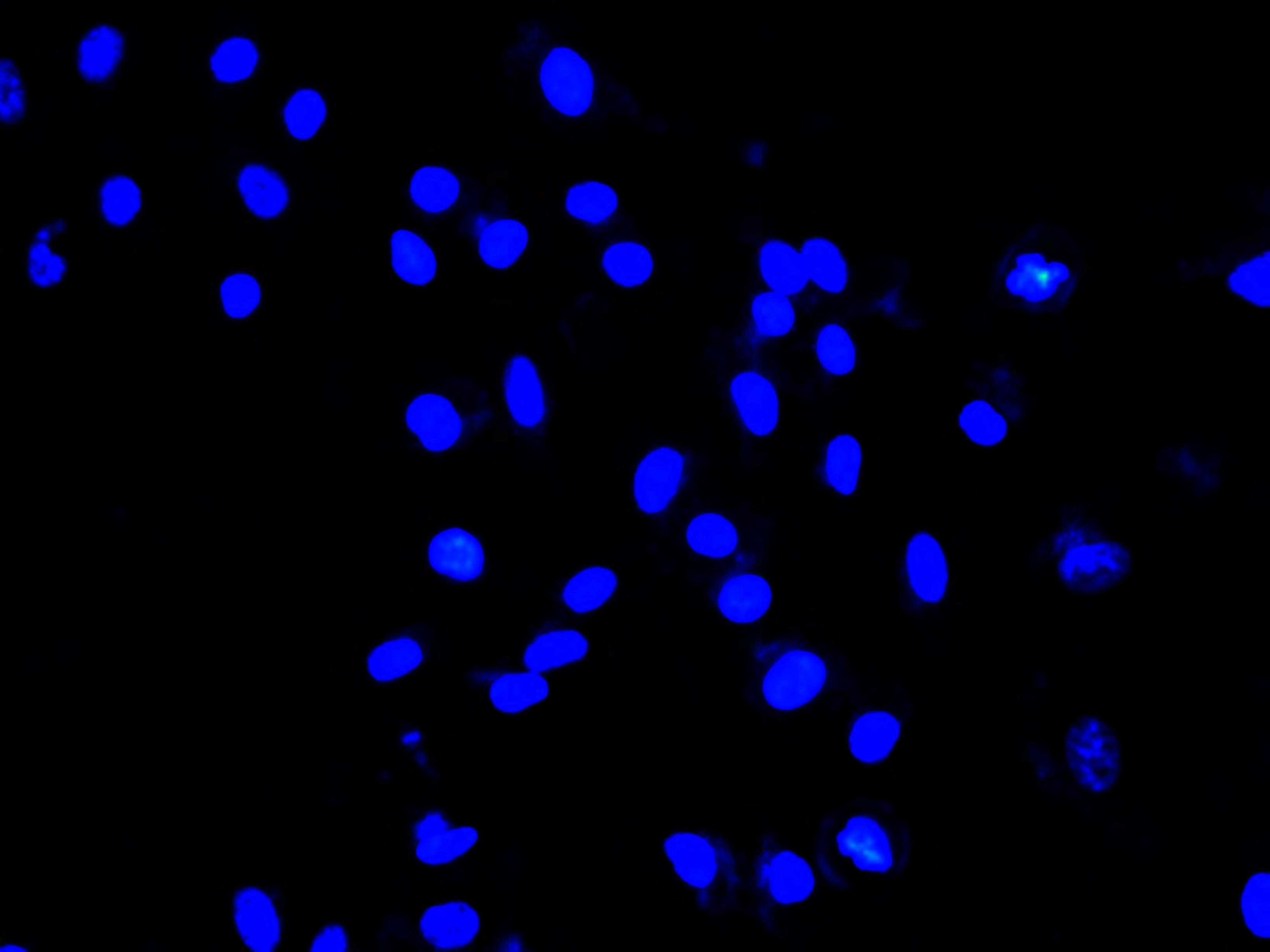

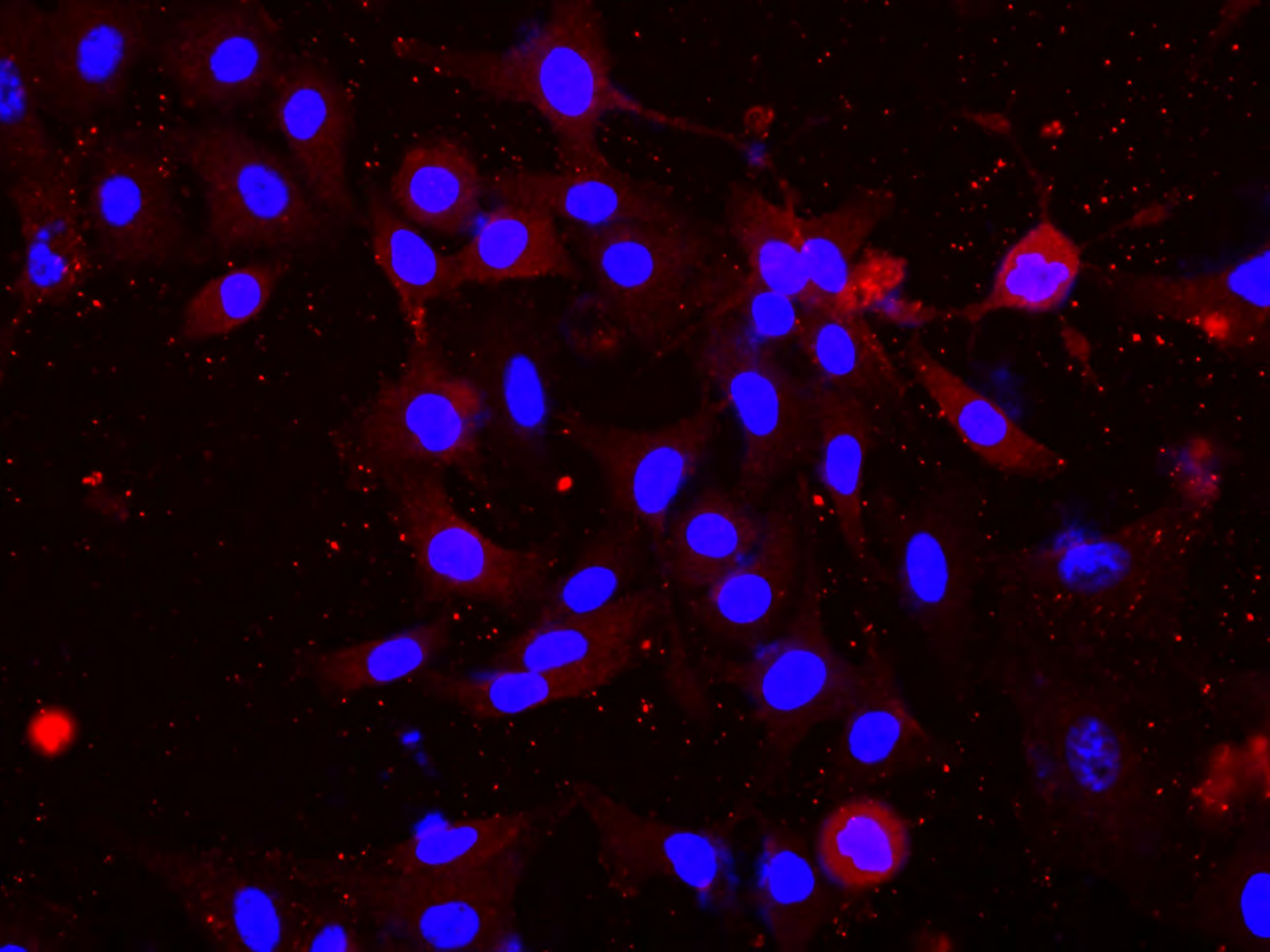

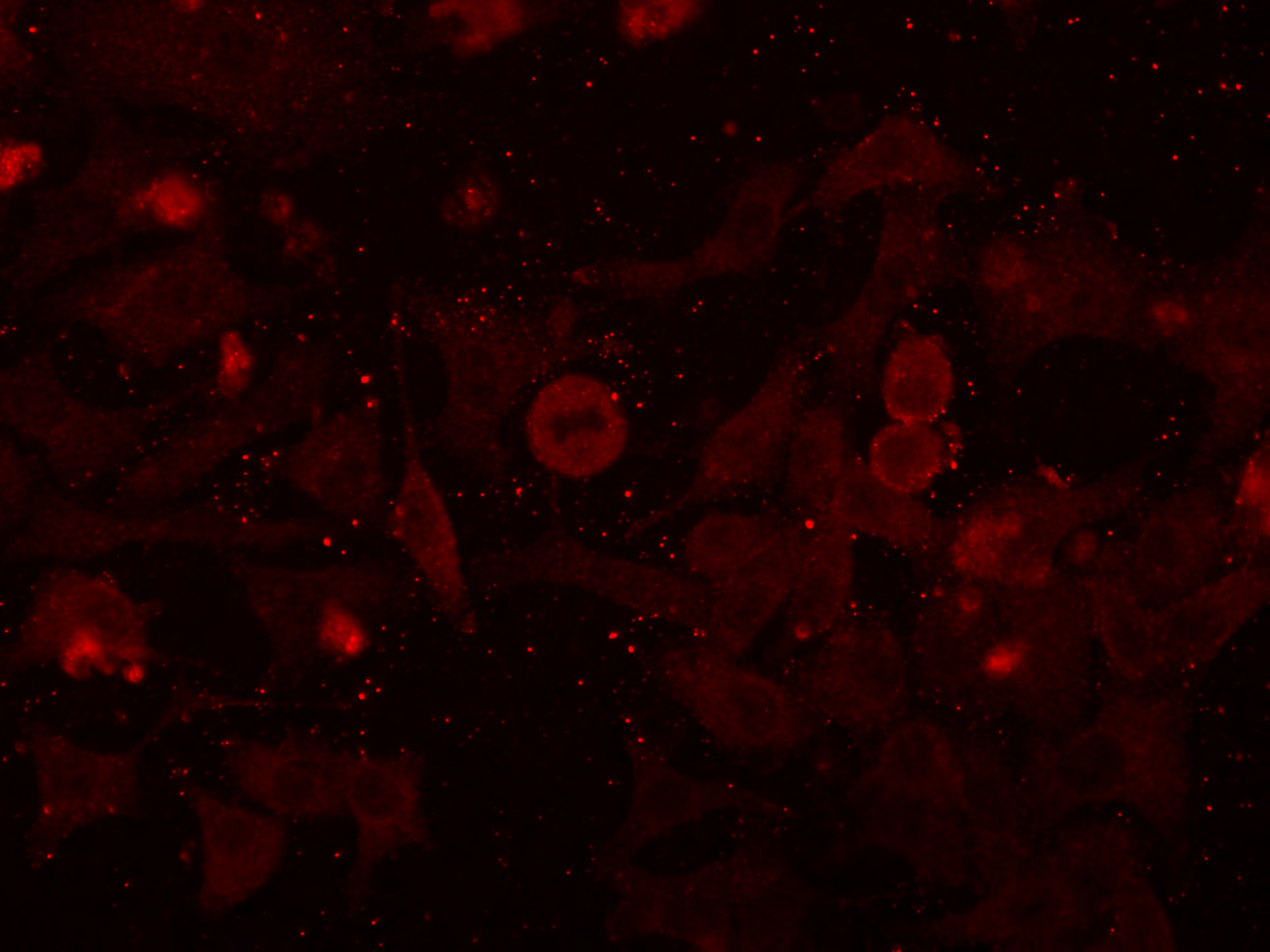

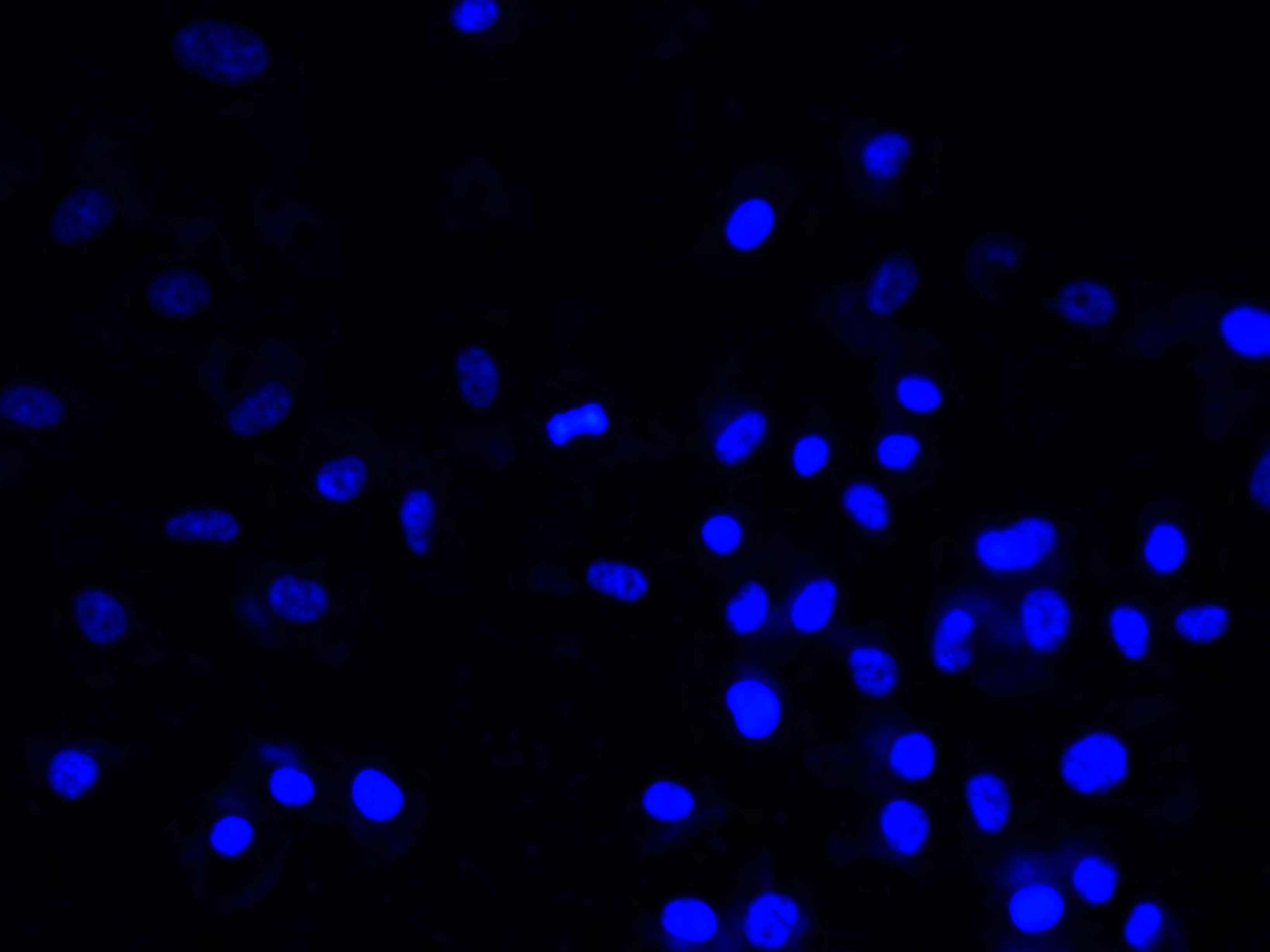

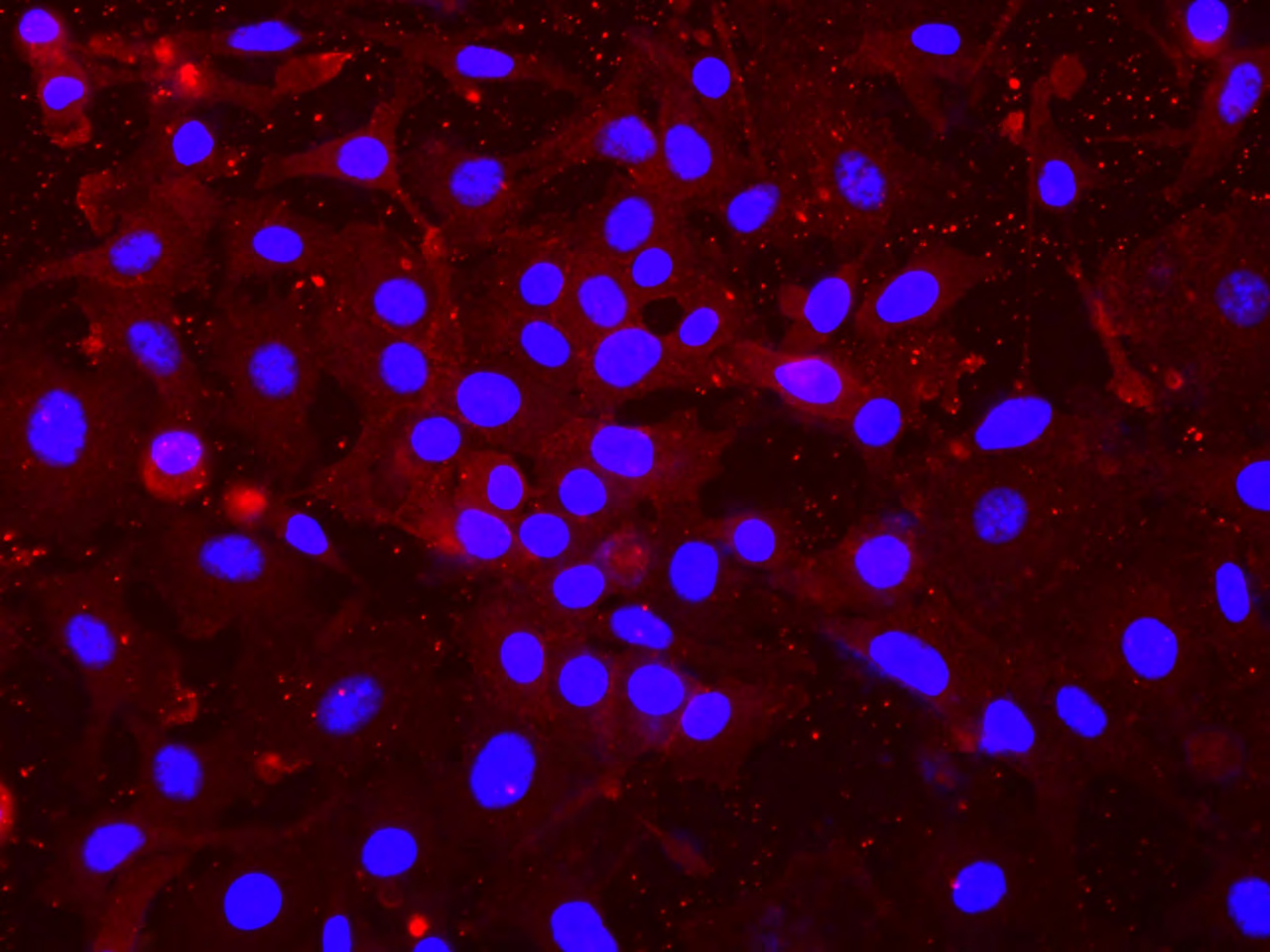

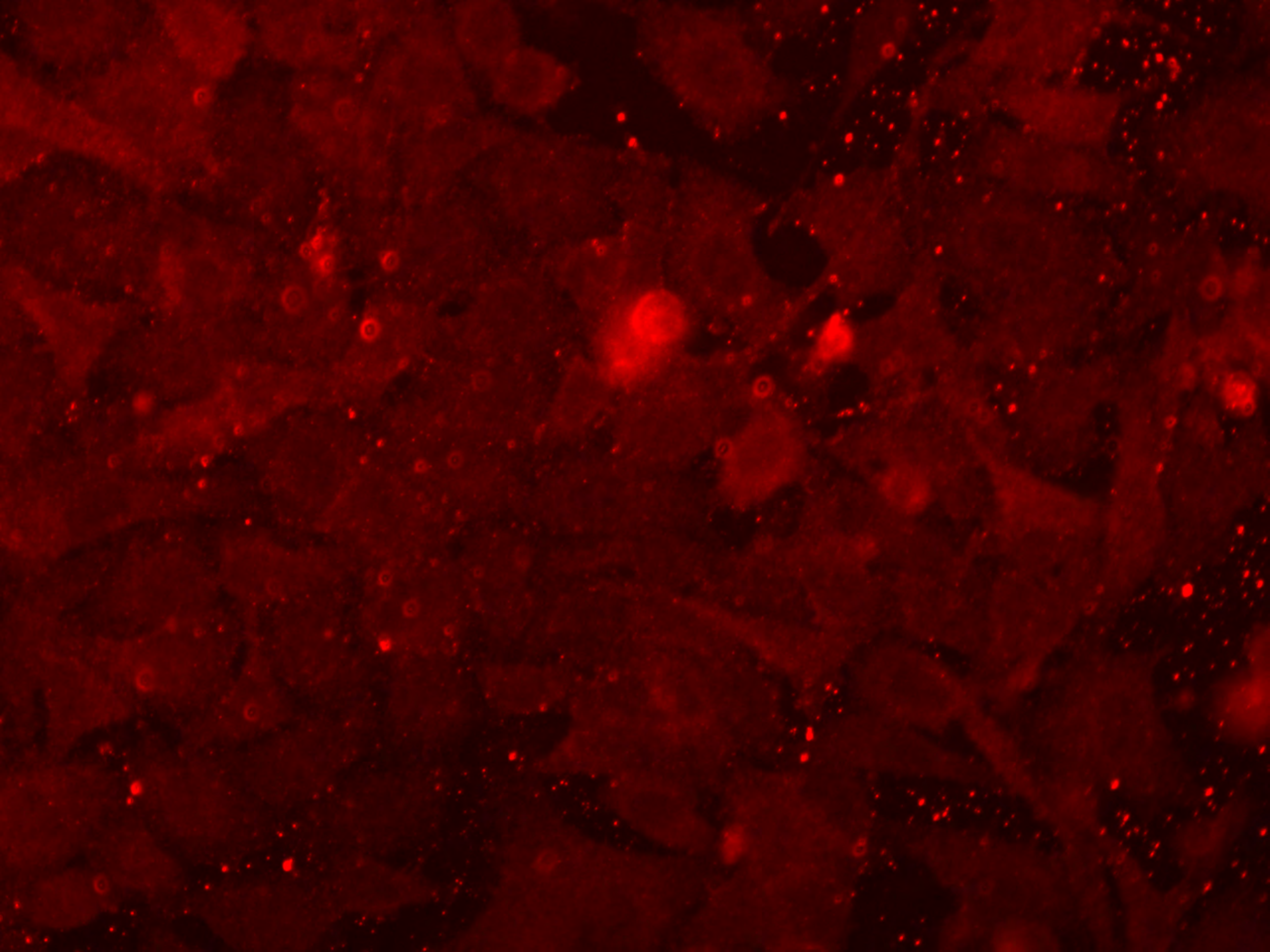

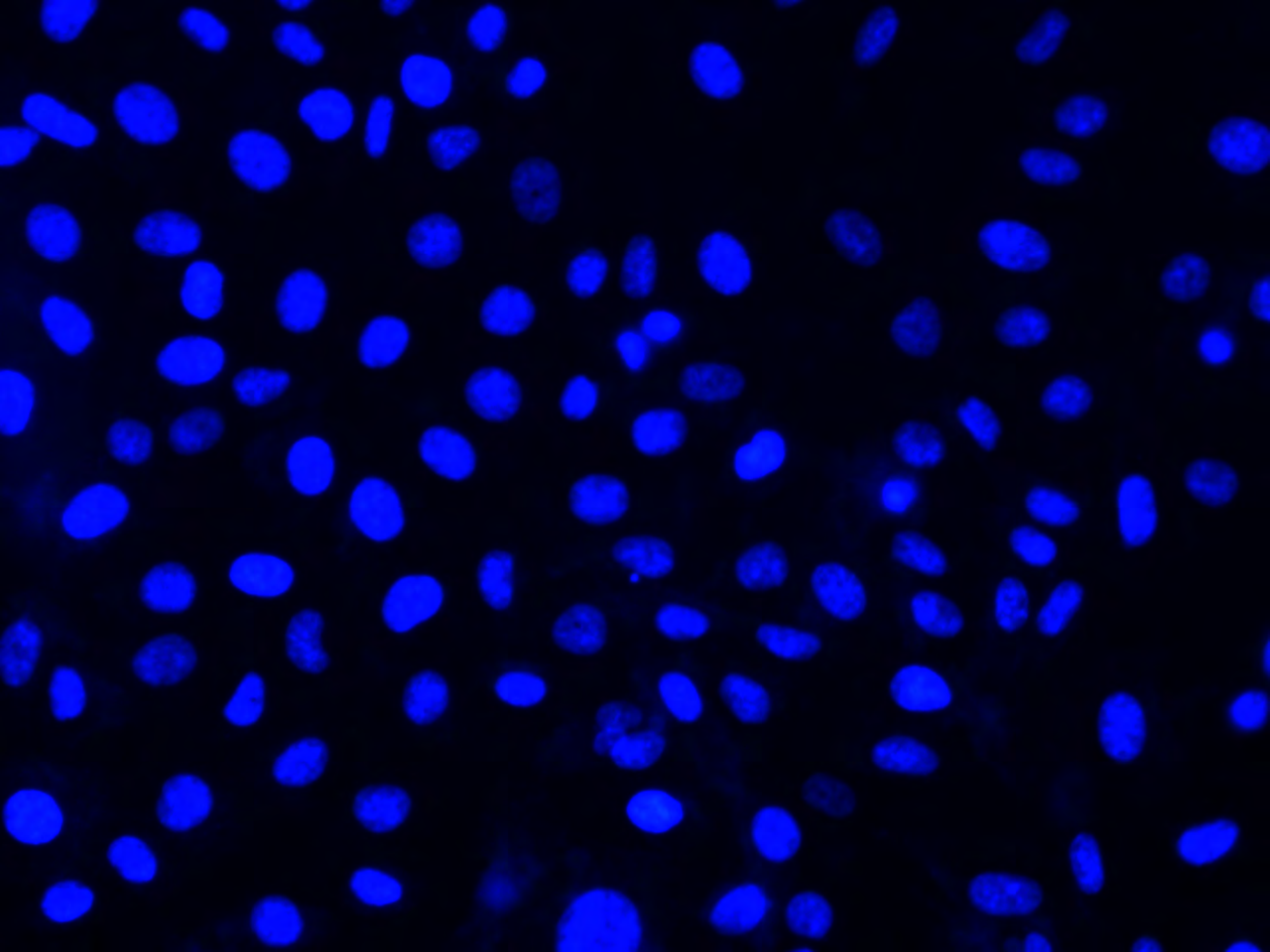

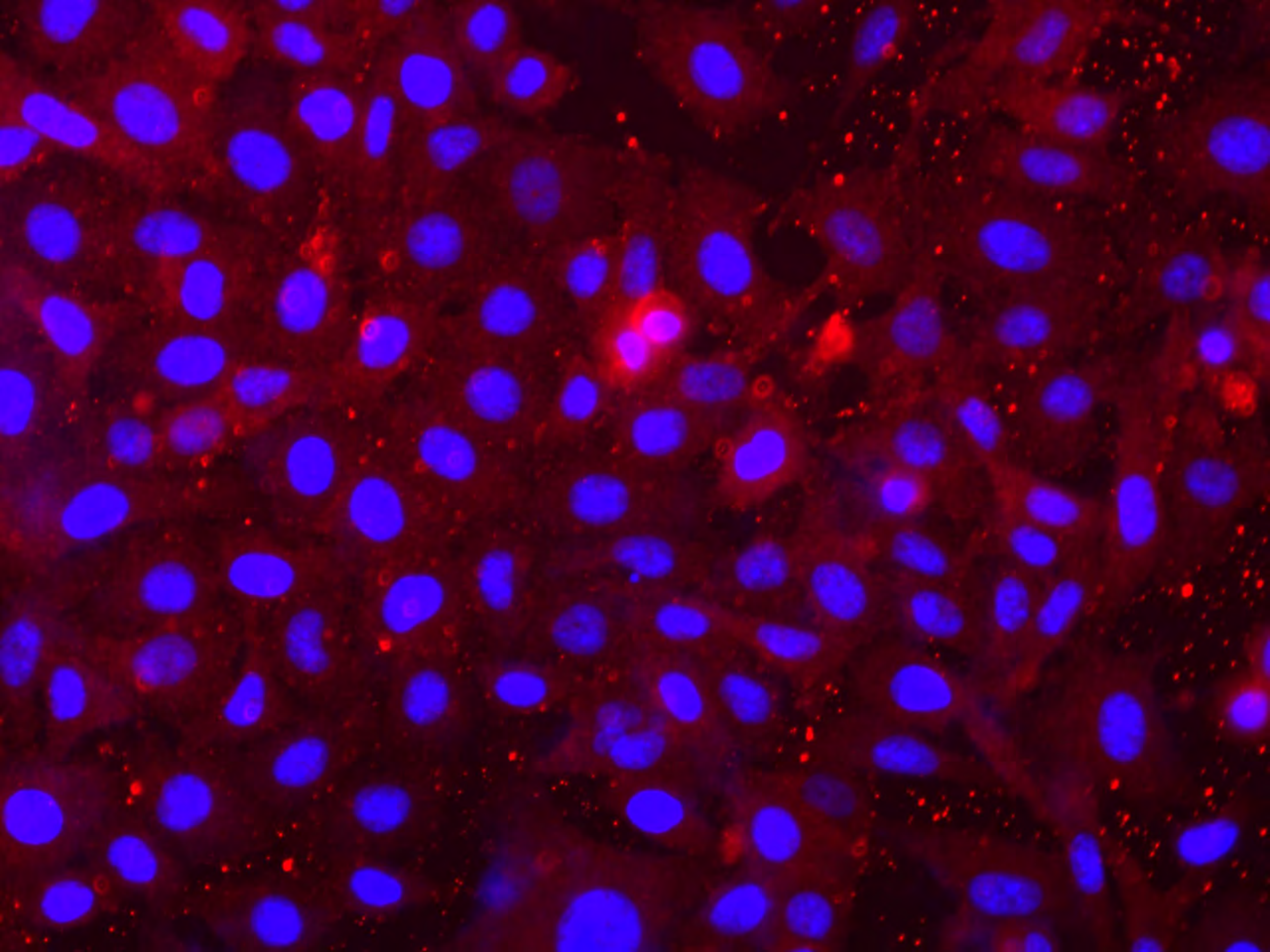

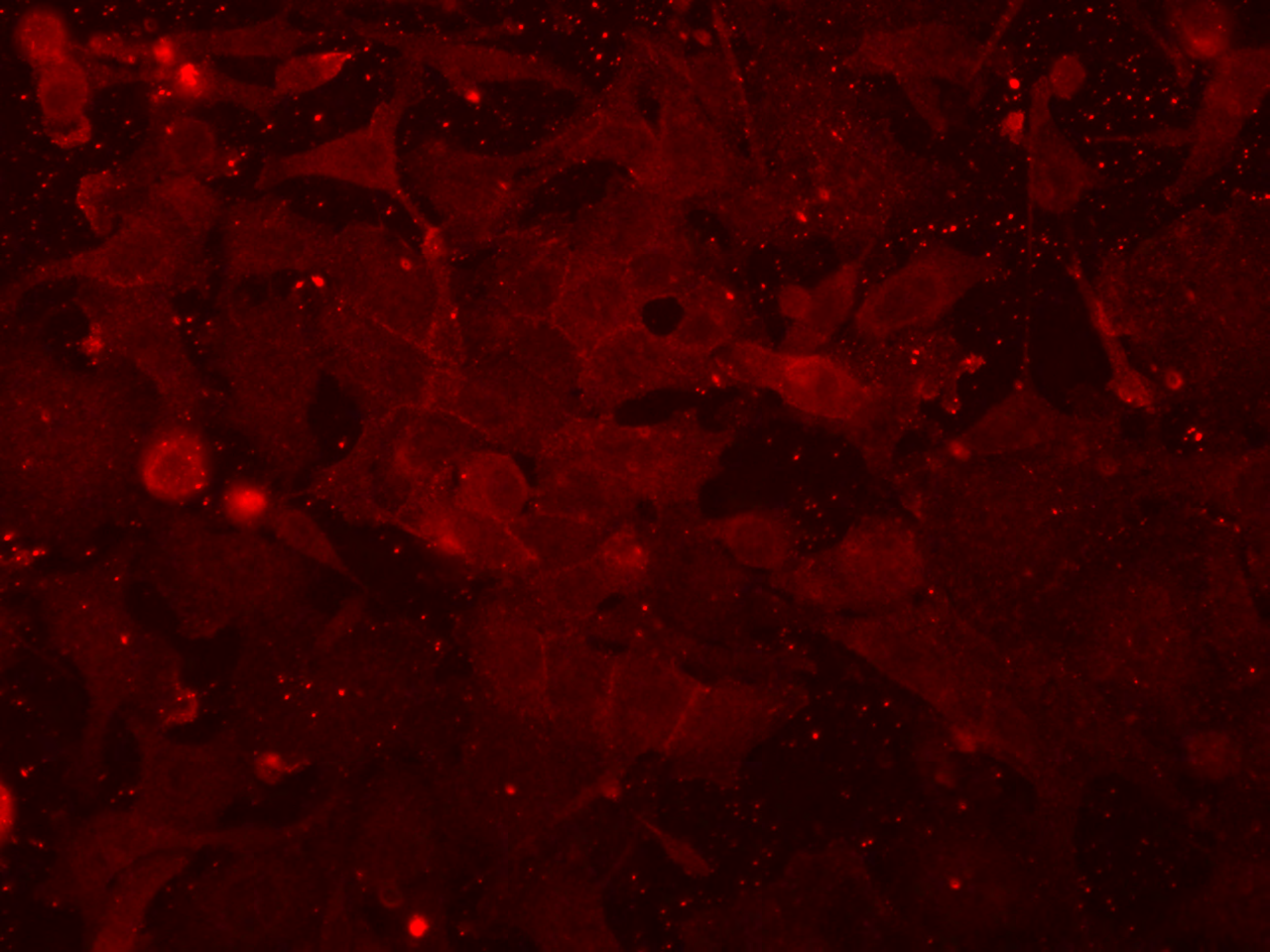

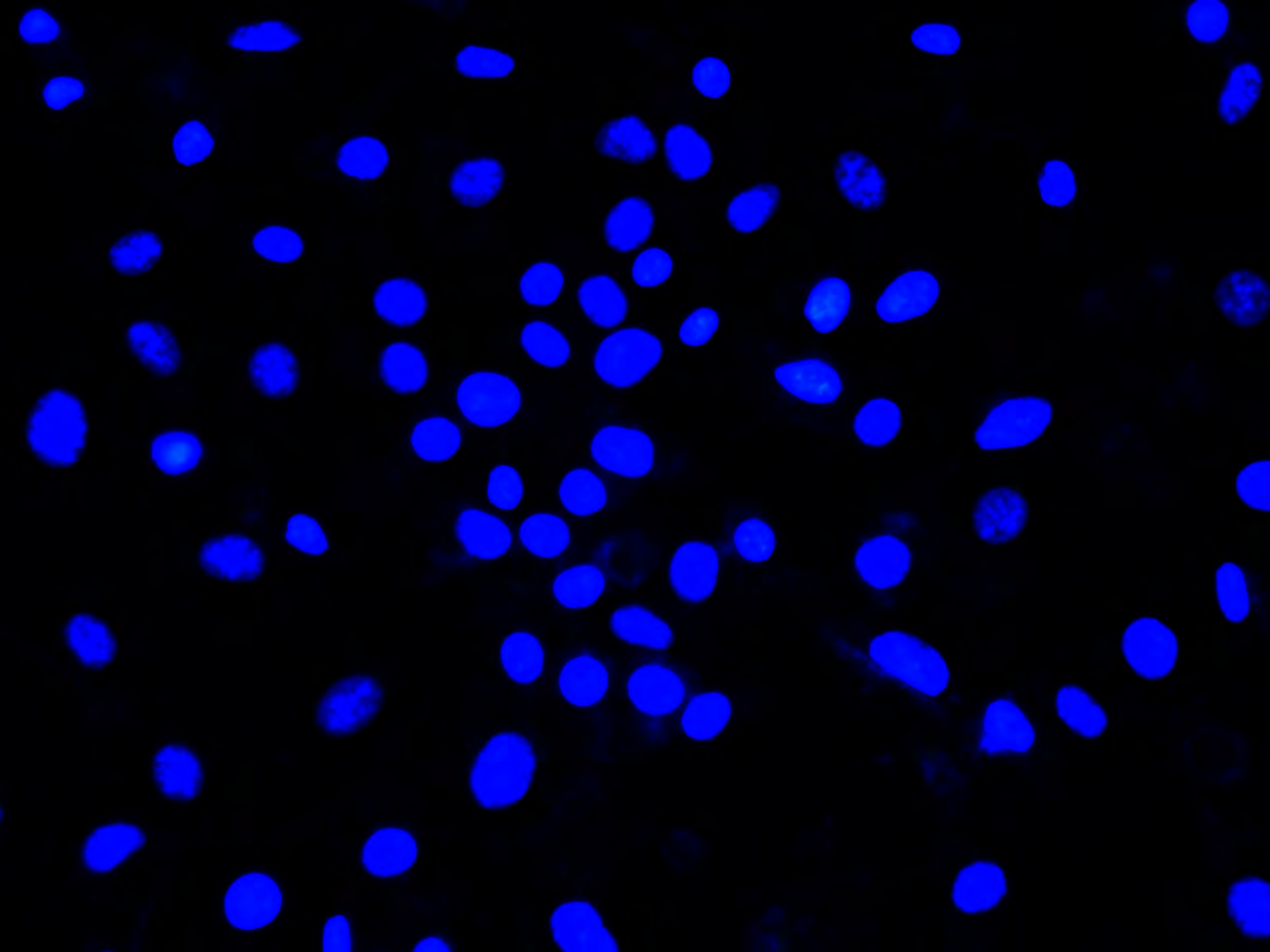

Supplement: Supplementary file 1 [file 4.pdf › raw data/Fig 8/β-catenin BAP31 immunofluorescence/β-catenin/4.pdf]
